# Supplementary material for: The methyltransferase NmbA methylates the low-molecular weight thiol bacillithiol, and displays a specific structural architecture
Source: Redox Biol. 2025 Nov 17;88:103937. doi: 10.1016/j.redox.2025.103937 (PMC12719100; doi:10.1016/j.redox.2025.103937)
Supplement: Multimedia component 1 [file mmc1.pdf]

Supplementary data

The methyltransferase NmbA methylates the low-molecular weight thiol bacillithiol, and displays a specific structural architecture

Marta Hammerstad[a], Erlend Steinvik[b], and Hans-Petter Hersleth[a]

[a]Section for Biochemistry and Molecular Biology, Department of Biosciences, University of Oslo, PO Box 1066, Blindern, 0316 Oslo, Norway

[b]Department of Chemistry, University of Oslo, PO Box 1033, Blindern, 0315 Oslo, Norway

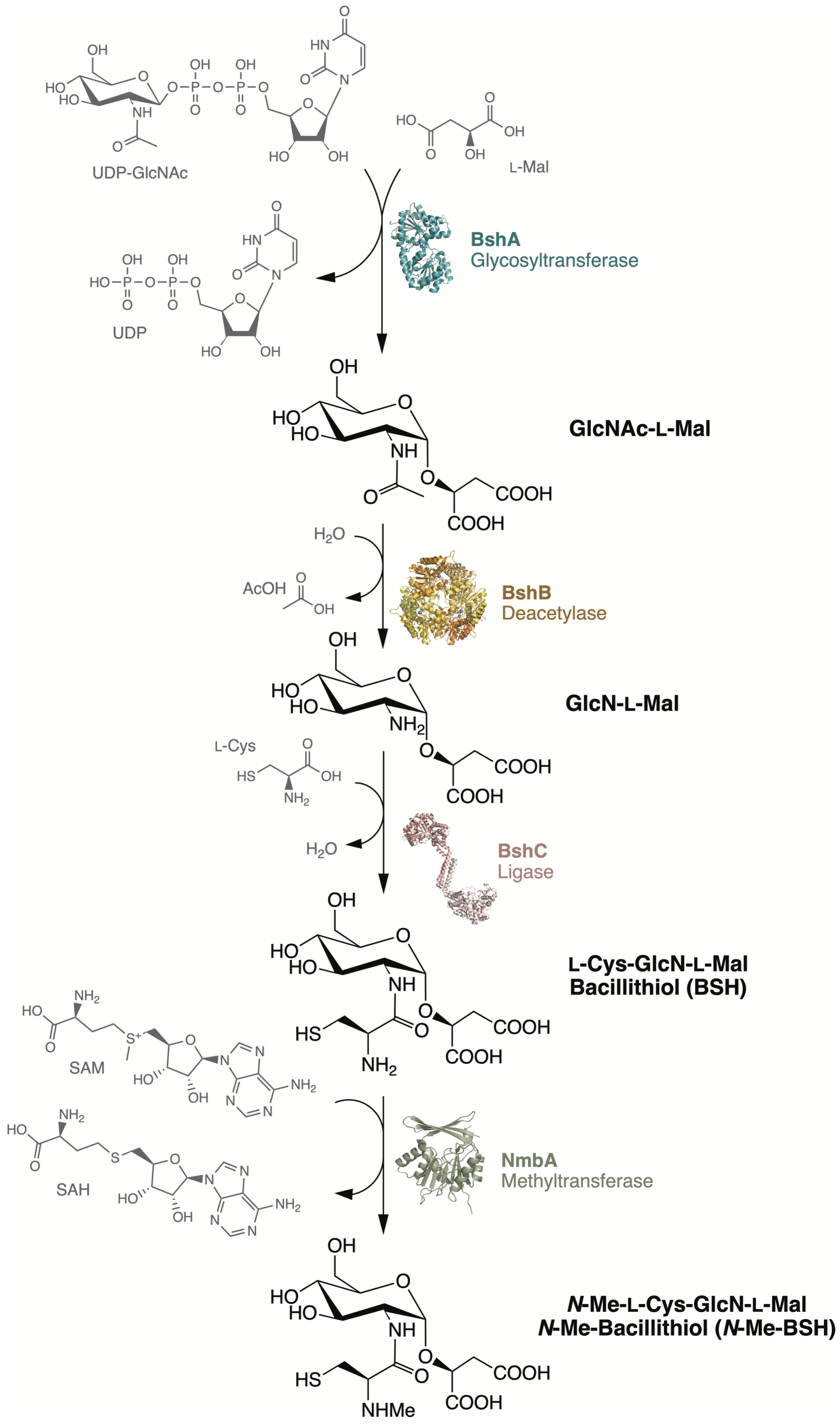

Figure S1. *N*-Me-BSH biosynthesis pathway. The glycosyltransferase BshA (PDBid:6D9T [1]) catalyzes the first step in (*N*-Me)-BSH biosynthesis, utilizing UDP-GlcNAc (uridine 5'-diphosphate-*N*-acetylglucosamine) and L-Mal (L-malate) as substrates, resulting in formation of GlcNAc-L-Mal (malyl-*N*-acetyl-D-glucosamine). GlcNAc-L-Mal is further deacetylated by deacetylase BshB (PDBid:6ULL [2]) to GlcN-L-Mal (malyl-D-glucosamine), which together with L-Cys (L-cysteine) serve as substrates for ligase BshC (PDBid:4WBD [3]) in the formation of BSH. Lastly, in *N*-Me-BSH-producing bacteria, NPMT NmbA catalyzes methylation of the cysteine N-atom of BSH, resulting in *N*-Me-BSH.

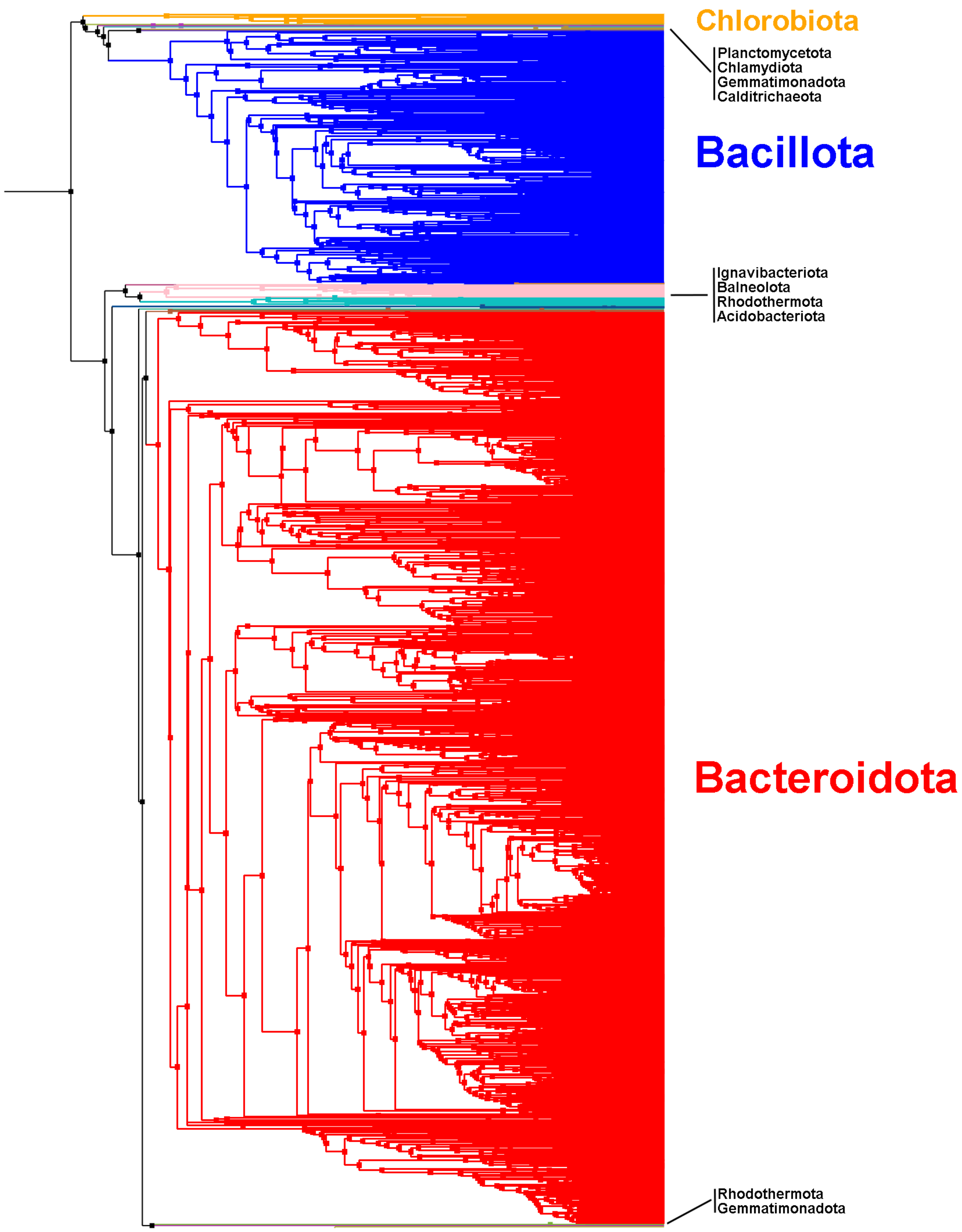

Figure S2. Phylogenetic tree analysis of NmbA homologs. Phylogenetic tree calculated in JalView with average distances using the BLOSUM62 matrix on the CT1040 homologous sequences listed in Supplementary Dataset 1. The clades are colored by bacterial phyla.

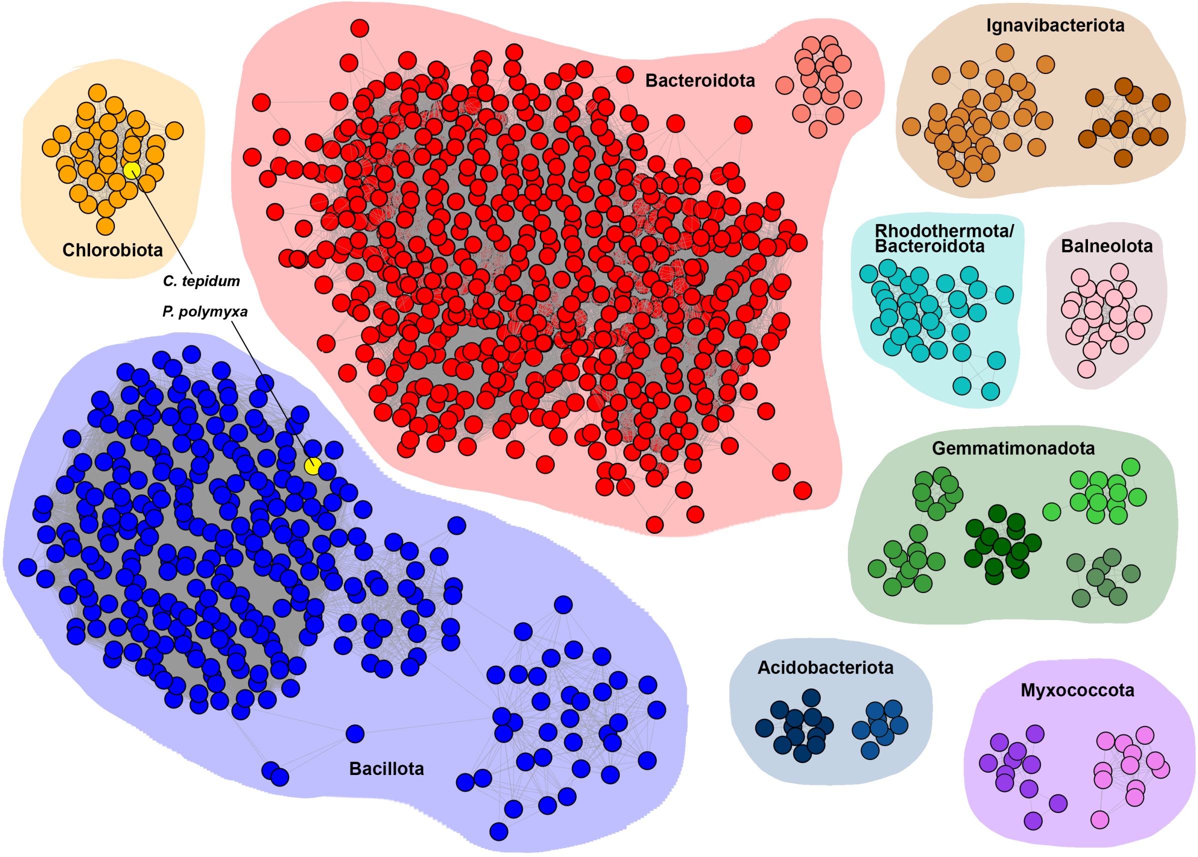

Figure S3. Comparison of *Ct*NmbA with homologous MTs through sequence similarity networks (SSNs). The SSN displays the nine largest clusters. The clusters are individually colored and assembled into groups with respect to bacterial phyla. Selected representative species from Chlorobiota and Bacillota encoding the *N*-Me-BSH biosynthetic genes are indicated. The edges (gray lines) connecting the protein nodes (circles) indicate the level of sequence similarity.

A

	Total % products, including by-products					
	Me-BSSB $C_{27}H_{44}N_4O_{20}S_2$	Me-BSSB-Me $C_{28}H_{46}N_4O_{20}S_2$	Me-BSSB + Me-BSSB-Me $C_{27}H_{44}N_4O_{20}S_2$ + $C_{28}H_{46}N_4O_{20}S_2$	$C_{14}H_{22}N_2O_{10}S$	$C_{15}H_{24}N_2O_{10}S$	$C_{17}H_{30}N_2O_{10}S$
*Ct*NmbA	17 ± 7	57 ± 10	74 ± 7	0.5 ± 0.5	9.8 ± 9.7	10 ± 4.4
*Pp*NmbA	21 ± 5	48 ± 10	69 ± 9	0.7 ± 0.4	13.6 ± 9	10.4 ± 4.1
Ct + *Pp*NmbA	19 ± 6	53 ± 11	72 ± 8	0.6 ± 0.5	11.7 ± 9.2	10.2 ± 4.1

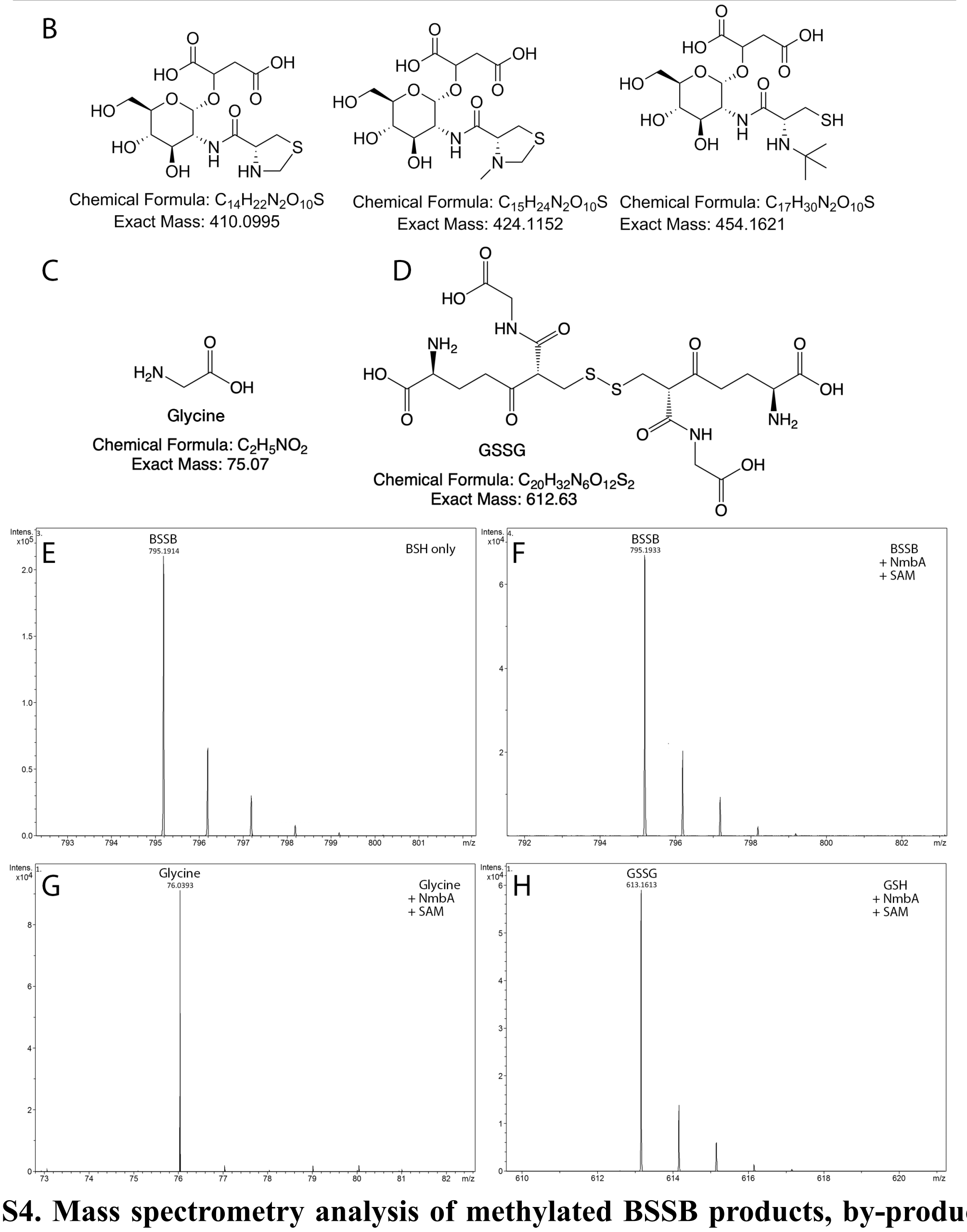

Figure S4. Mass spectrometry analysis of methylated BSSB products, by-products, and alternative substrates. (A) The calculated percentage of methylated BSSB forms from the enzymatic reactions using BSH as a substrate and SAM as co-substrate, catalyzed by *Ct*NmbA or *Pp*NmbA, including three by-products. The numbers are calculated from the total BSSB pool (unmethylated BSSB, Me-BSSB, and Me-BSSB-Me) as well as the by-products from the enzymatic reactions. **(B)** Proposed structures of the three detected by-products and the structures of glycine **(C)** and GSSG **(D). (E) and (F)** show representative mass spectra of unmethylated BSSB ($[M + H]^+$795.1914 or 795.1933) from a sample of BSH only **(E)** and from a reaction catalyzed by *Ct*NmbA using oxidized BSSB as a substrate **(F)**. Characteristic mass spectra from reactions using alternative substrates glycine ($[M + H]^+$76.0393) in **(G)** and using GSH (oxidized GSSG, $[M + H]^+$613.1613) in **(H)** catalyzed by *Pp*NmbA show no methylated products.

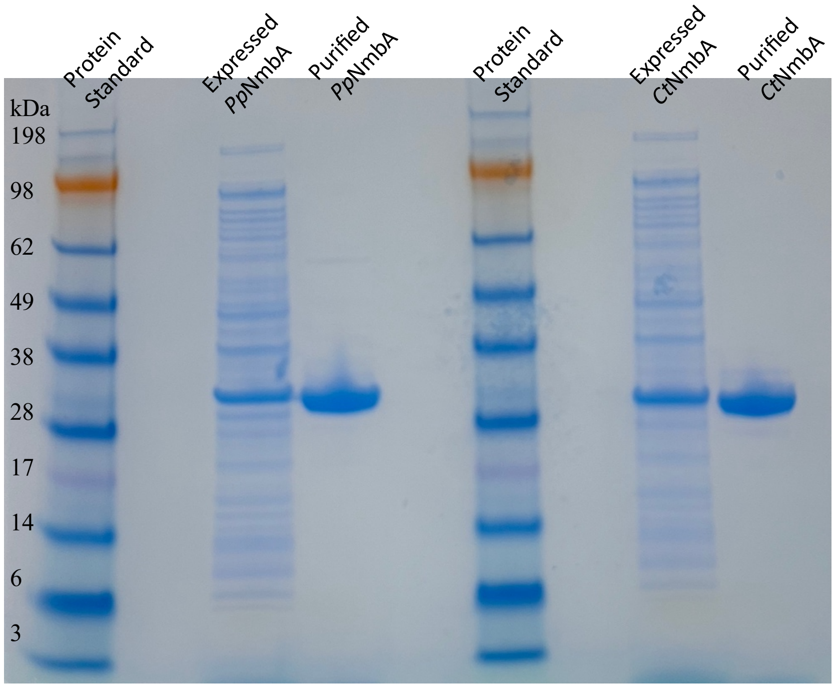

Figure S5. SDS-PAGE analysis. SDS-PAGE showing the overexpression of *Pp*NmbA (29.7 kDa) and *Ct*NmbA (30.5 kDa) in *E. coli* One Shot^{TM} BL21 (DE3) cells (Invitrogen, Thermo Fischer Scientific), as well as the final purified protein samples. The SDS-PAGE was run using a Bolt Bis-Tris Plus Mini Protein Gel, 4-12%, 1.0 mm, WedgeWell format with a 1x Bolt MES SDS Running Buffer, and a SeeBlue Plus2 Pre-stained Protein Standard (all from Thermo Fisher Scientific).

References

[1] C.J. Royer, P.D. Cook, A structural and functional analysis of the glycosyltransferase BshA from Staphylococcus aureus: Insights into the reaction mechanism and regulation of bacillithiol production, Protein Science 28(6) (2019) 1083-1094.

[2] R.L. Woodward, M.M. Castleman, C.E. Meloche, M.E. Karpen, C.G. Carlson, W.H. Yobi, J.C. Jepsen, B.W. Lewis, B.N. Zarnosky, P.D. Cook, X-ray crystallographic structure of BshB, the zinc-dependent deacetylase involved in bacillithiol biosynthesis, Protein Science 29(4) (2020) 1035-1039.

[3] A.J. VanDuinen, K.R. Winchell, M.E. Keithly, P.D. Cook, X-ray Crystallographic Structure of BshC, a Unique Enzyme Involved in Bacillithiol Biosynthesis, Biochemistry 54(2) (2015) 100-103.
