# Supplementary material for: The methyltransferase NmbA methylates the low-molecular weight thiol bacillithiol, and displays a specific structural architecture
Source: Redox Biol. 2025 Nov 17;88:103937. doi: 10.1016/j.redox.2025.103937 (PMC12719100; doi:10.1016/j.redox.2025.103937)
Supplement: Multimedia component 2 [file mmc2.pdf]

Supplementary Dataset Overview - Distribution of unique species among bacterial phyla encoding the *N*-Me-BSH or BSH biosynthetic pathways*†.

Bacterial phylum	# species with homologs of NmbA, BshA, BshB, and BshC	# species with homologs of BshA, BshB, and BshC
Acidobacteriota	4	51
Bacillota	366	1466
Bacteroidota	1329	2111
Balneolota	18	28
Calditrichaeota	1	1
Chlamydiota	3	3
Chlorobiota	15	15
Cyanobacteriota	0	1
Deinococcota	1	107
Gemmatimonadota	8	13
Ignavibacteriota	2	2
Myxococcota	2	42
Planctomycetota	2	2
Rhodothermota	13	15

* Searching remaining bacterial phyla validly published according to the Bacteriological Code gave no hits of members containing the complete BSH nor *N*-Me-BSH biosynthetic pathways. These included: Actinomycetota, Aquificota, Armatimonadota, Atribacterota, Bdellovibrionota, Caldisericota, Campylobacterota, Chloroflexota, Chrysiogenota, Coprothermobacterota, Deferribacterota, Dictyoglomota, Elusimicrobiota, Fibrobacterota, Fusobacteriota, Kiritimatiellota, Lentisphaerota, Mycoplasmatota, Nitrospinota, Nitrospirota, Pseudomonadota, Spirochaetota, Synergistota, Thermodesulfobacteriota, Thermomicrobiota, Thermoproteota, Thermotogota, and Verrucomicrobiota.

† A complete overview of all species in this table, including organism names and accession codes, is listed in Supplementary Dataset 1.

Supplementary Dataset 1A – Unique species containing the complete *N*-Me-BSH biosynthetic pathway. Phylum, species, and NCBI accession codes for NmbA, BshA, BshB, and BshC are listed for each organism.

Phylum	Species	Accession code NmbA	Accession code BshA	Accession code BshB	Accession code BshC
Acidobacteriota	*Chloracidobacterium aggregatum*	WP_211427380.1	WP_211422436.1	WP_211423485.1	WP_211426094.1
Acidobacteriota	*Chloracidobacterium sp. D*	WP_211433143.1	WP_211432135.1	WP_211433022.1	WP_211432012.1
Acidobacteriota	*Chloracidobacterium thermophilum*	WP_014101269.1	WP_014098778.1	WP_058865916.1	WP_014098599.1
Acidobacteriota	*Chloracidobacterium validum*	WP_211430584.1	WP_211428433.1	WP_211430360.1	WP_211428837.1
Bacillota	*Aliibacillus thermotolerans*	WP_270895466.1	WP_270897557.1	WP_270897558.1	WP_270898399.1
Bacillota	*Alkalihalobacillus deserti*	WP_227937551.1	WP_227936340.1	WP_227936216.1	WP_227935718.1
Bacillota	*Alkalihalobacillus sp. LMS39*	WP_243527333.1	WP_243527612.1	WP_243527614.1	WP_243528494.1
Bacillota	*Alkalihalobacterium bogoriense*	WP_026671931.1	WP_035178327.1	WP_035178325.1	WP_026674384.1
Bacillota	*Alkalihalophilus lindianensis*	WP_317123171.1	WP_317120797.1	WP_317120796.1	WP_317122523.1
Bacillota	*Alkalihalophilus marmarensis*	WP_326239376.1	WP_022627180.1	WP_326238214.1	WP_326240074.1
Bacillota	*Alkalihalophilus pseudofirmus*	WP_289235703.1	WP_075682216.1	WP_012958526.1	WP_075682601.1
Bacillota	*Alkalihalophilus sp. As8PL*	WP_368505392.1	WP_368504480.1	WP_368504481.1	WP_368503982.1
Bacillota	*Alteribacillus bidgolensis*	WP_091579508.1	WP_091579714.1	WP_245917746.1	WP_091582817.1
Bacillota	*Alteribacillus iranensis*	WP_091656492.1	WP_091656672.1	WP_091656669.1	WP_091657528.1
Bacillota	*Alteribacillus persepolensis*	WP_091271002.1	WP_091270857.1	WP_245705149.1	WP_091270224.1
Bacillota	*Alteribacillus sp. JSM 102045*	WP_375006769.1	WP_375006684.1	WP_375006685.1	WP_375005851.1
Bacillota	*Ammoniphilus resinae*	WP_209809244.1	WP_209811430.1	WP_209811431.1	WP_209808725.1
Bacillota	*Ammoniphilus sp. YIM 78166*	WP_167577707.1	WP_134699003.1	WP_134699002.1	WP_167577581.1
Bacillota	*Anaerobacillus sp.*	WP_409848335.1	WP_409852530.1	WP_409852635.1	WP_409849617.1
Bacillota	*Aneurinibacillus aneurinilyticus*	WP_276915438.1	WP_370951626.1	WP_021622598.1	WP_021624582.1
Bacillota	*Aneurinibacillus migulanus*	WP_043066703.1	WP_043067898.1	WP_235355982.1	WP_043066087.1
Bacillota	*Aneurinibacillus sp. REN35*	WP_371148367.1	WP_371146670.1	WP_371146669.1	WP_371147049.1
Bacillota	*Aneurinibacillus terranovensis*	WP_027417799.1	WP_027417519.1	WP_035102034.1	WP_027414484.1
Bacillota	*Aneurinibacillus tyrosinisolvens*	WP_047152122.1	WP_047150554.1	WP_047152599.1	WP_052947533.1

Bacillota	*Aneurinibacillus uraniiduransis*	WP_272562244.1	WP_272560005.1	WP_272560006.1	WP_272562398.1
Bacillota	*Anoxybacillus calidus*	WP_181537656.1	WP_181535810.1	WP_181535808.1	WP_181536607.1
Bacillota	*Bacillus alveayuensis*	WP_044894876.1	WP_044748079.1	WP_044748078.1	WP_419151291.1
Bacillota	*Bacillus cereus group*	WP_065212475.1	WP_097817830.1	WP_070172932.1	WP_002165144.1
Bacillota	*Bacillus piscicola*	WP_240377695.1	WP_240375564.1	WP_338021128.1	WP_240375160.1
Bacillota	*Bacillus solitudinis*	WP_100407558.1	WP_100407236.1	WP_100408361.1	WP_100404574.1
Bacillota	*Bacillus sp. FJAT-45037*	WP_100372031.1	WP_100372728.1	WP_100372727.1	WP_157796518.1
Bacillota	*Baia soyae*	WP_243649462.1	WP_131848545.1	WP_131848544.1	WP_131847547.1
Bacillota	*Brevibacillus agri*	WP_290426100.1	WP_327944617.1	WP_005831237.1	WP_005833010.1
Bacillota	*Brevibacillus antibioticus*	WP_137029627.1	WP_137029308.1	WP_137029309.1	WP_137028103.1
Bacillota	*Brevibacillus borstelensis*	WP_340257345.1	WP_171506301.1	WP_340250559.1	WP_024983988.1
Bacillota	*Brevibacillus brevis*	WP_310772139.1	WP_144616023.1	WP_310772836.1	WP_106653219.1
Bacillota	*Brevibacillus centrosporus*	WP_092267967.1	WP_411504249.1	WP_327930207.1	WP_328211152.1
Bacillota	*Brevibacillus choshinensis*	WP_327995948.1	WP_203356916.1	WP_327976965.1	WP_055746698.1
Bacillota	*Brevibacillus composti*	WP_198826645.1	WP_198826360.1	WP_198826361.1	WP_198829395.1
Bacillota	*Brevibacillus daliensis*	WP_232699265.1	WP_232696377.1	WP_232696376.1	WP_232698373.1
Bacillota	*Brevibacillus dissolubilis*	WP_139489532.1	WP_139489136.1	WP_139489137.1	WP_139489967.1
Bacillota	*Brevibacillus fluminis*	WP_122919557.1	WP_122920747.1	WP_122920746.1	WP_122916979.1
Bacillota	*Brevibacillus formosus*	WP_047068682.1	WP_047071010.1	WP_047069015.1	WP_047068378.1
Bacillota	*Brevibacillus fortis*	WP_409178351.1	WP_106841588.1	WP_327950005.1	WP_106840056.1
Bacillota	*Brevibacillus gelatini*	WP_122903973.1	WP_122904259.1	WP_122904258.1	WP_122906096.1
Bacillota	*Brevibacillus humidisoli*	WP_230046769.1	WP_230046195.1	WP_230046959.1	WP_230045536.1
Bacillota	*Brevibacillus invocatus*	WP_122911184.1	WP_122908283.1	WP_122908779.1	WP_251243155.1
Bacillota	*Brevibacillus laterosporus*	WP_121473740.1	WP_031414565.1	WP_003341662.1	WP_104031805.1
Bacillota	*Brevibacillus marinus*	WP_126427700.1	WP_126427242.1	WP_126427244.1	WP_126425988.1
Bacillota	*Brevibacillus massiliensis*	WP_019119801.1	WP_019122419.1	WP_019122420.1	WP_019120713.1
Bacillota	*Brevibacillus migulae*	WP_134682950.1	WP_134684718.1	WP_134686655.1	WP_134687458.1
Bacillota	*Brevibacillus nitrificans*	WP_310234934.1	WP_310227961.1	WP_310227962.1	WP_328207014.1

Bacillota	*Brevibacillus panacihumi*	WP_023554712.1	WP_122912326.1	WP_427329339.1	WP_427329734.1
Bacillota	*Brevibacillus parabrevis*	WP_063227760.1	WP_063226980.1	WP_063227869.1	WP_063230376.1
Bacillota	*Brevibacillus porteri*	WP_376897442.1	WP_106833023.1	WP_106835974.1	WP_106835688.1
Bacillota	*Brevibacillus reuszeri*	WP_103108679.1	WP_049740083.1	WP_049740084.1	WP_213022732.1
Bacillota	*Brevibacillus ruminantium*	WP_251870872.1	WP_251870562.1	WP_251870563.1	WP_251874646.1
Bacillota	*Brevibacillus sp. SKDU10*	WP_064018533.1	WP_064018445.1	WP_064018446.1	WP_064018249.1
Bacillota	*Brevibacillus thermoruber*	WP_029099275.1	WP_029097619.1	WP_029099570.1	WP_271140081.1
Bacillota	*Caldalkalibacillus mannanilyticus*	WP_025026615.1	WP_025026795.1	WP_025026794.1	WP_025028057.1
Bacillota	*Caldalkalibacillus salinus*	WP_202078626.1	WP_272899181.1	WP_202079975.1	WP_202076947.1
Bacillota	*Caldalkalibacillus thermarum*	WP_188622173.1	WP_188623426.1	WP_188623462.1	WP_222822622.1
Bacillota	*Caldalkalibacillus uzonensis*	WP_307340273.1	WP_307334880.1	WP_307334882.1	WP_307338827.1
Bacillota	*Calditerricola satsumensis*	WP_188816489.1	WP_054672757.1	WP_054672754.1	WP_188816587.1
Bacillota	*Chengkuizengella axinellae*	WP_305989955.1	WP_305991132.1	WP_305991131.1	WP_305990434.1
Bacillota	*Chengkuizengella marina*	WP_160645612.1	WP_160647308.1	WP_160647307.1	WP_160646215.1
Bacillota	*Chengkuizengella sediminis*	WP_162038533.1	WP_162035760.1	WP_162035759.1	WP_162035053.1
Bacillota	*Chengkuizengella sp. SCS-71B*	WP_349250319.1	WP_349248815.1	WP_349248814.1	WP_349249855.1
Bacillota	*Cohnella abietis*	WP_130606950.1	WP_130608606.1	WP_157994104.1	WP_157994083.1
Bacillota	*Cohnella boryungensis*	WP_204601817.1	WP_204603271.1	WP_204603272.1	WP_204601225.1
Bacillota	*Cohnella faecalis*	WP_119148594.1	WP_119147875.1	WP_119147874.1	WP_158593973.1
Bacillota	*Cohnella herbarum*	WP_169278050.1	WP_169278750.1	WP_169278749.1	WP_169279893.1
Bacillota	*Cohnella hongkongensis*	WP_378102549.1	WP_378096723.1	WP_378096104.1	WP_378097115.1
Bacillota	*Cohnella luojiensis*	WP_135152141.1	WP_135150944.1	WP_135150943.1	WP_167746931.1
Bacillota	*Cohnella lupini*	WP_115992159.1	WP_115993120.1	WP_115993121.1	WP_115990748.1
Bacillota	*Cohnella mopanensis*	WP_239615160.1	WP_239614212.1	WP_239614211.1	WP_239617380.1
Bacillota	*Cohnella silvisoli*	WP_232187555.1	WP_232183992.1	WP_232186837.1	WP_232187431.1
Bacillota	*Cohnella sp.*	WP_287021570.1	WP_372634402.1	WP_287022233.1	WP_287021775.1
Bacillota	*Croceifilum oryzae*	WP_307251400.1	WP_307254358.1	WP_307254361.1	WP_307250766.1
Bacillota	*Desertibacillus haloalkaliphilus*	WP_217221480.1	WP_217224796.1	WP_217224795.1	WP_217224580.1

Bacillota	*Desmospora activa*	WP_107724566.1	WP_107725504.1	WP_245891082.1	WP_107726050.1
Bacillota	*Desmospora profundinema*	WP_309865860.1	WP_309861822.1	WP_309861824.1	WP_309862629.1
Bacillota	*Fontibacillus panacisegetis*	WP_091228678.1	WP_091229626.1	WP_091229628.1	WP_091226566.1
Bacillota	*Fontibacillus phaseoli*	WP_114497056.1	WP_114495195.1	WP_114495194.1	WP_114494769.1
Bacillota	*Fontibacillus solani*	WP_182534579.1	WP_182535212.1	WP_182535213.1	WP_182535122.1
Bacillota	*Fontibacillus sp. BL9*	WP_410771892.1	WP_410768391.1	WP_410768390.1	WP_410768037.1
Bacillota	*Gordoniibacillus kamchatkensis*	WP_052487363.1	WP_156157817.1	WP_041046861.1	WP_041046255.1
Bacillota	*Gorillibacterium sp. CAU 1737*	WP_347771175.1	WP_347768118.1	WP_347768119.1	WP_347772139.1
Bacillota	*Gorillibacterium timonense*	WP_058302272.1	WP_058303859.1	WP_058303858.1	WP_082651929.1
Bacillota	*Halalkalibacter alkalisediminis*	WP_273839419.1	WP_273840757.1	WP_273840758.1	WP_273839757.1
Bacillota	*Halalkalibacter flavus*	WP_332633351.1	WP_332630148.1	WP_332630147.1	WP_332632203.1
Bacillota	*Halalkalibacter kiskunsagensis*	WP_335963127.1	WP_335958984.1	WP_335958985.1	WP_335960984.1
Bacillota	*Halalkalibacter lacteus*	WP_332696835.1	WP_332693267.1	WP_332693270.1	WP_332690015.1
Bacillota	*Hazenella coriacea*	WP_131923295.1	WP_131925631.1	WP_131925633.1	WP_131924031.1
Bacillota	*Kroppenstedtia eburnea*	WP_380132810.1	WP_076523778.1	WP_040387154.1	WP_380150828.1
Bacillota	*Kroppenstedtia guangzhouensis*	WP_188431726.1	WP_188430415.1	WP_188430417.1	WP_188428771.1
Bacillota	*Kroppenstedtia pulmonis*	WP_173220744.1	WP_173224572.1	WP_173221991.1	WP_173222804.1
Bacillota	*Kroppenstedtia sanguinis*	WP_380162730.1	WP_380163115.1	WP_380163114.1	WP_380164071.1
Bacillota	*Laceyella putida*	WP_379862863.1	WP_379863246.1	WP_379865748.1	WP_379866126.1
Bacillota	*Laceyella sacchari*	WP_132219594.1	WP_259436017.1	WP_259436617.1	WP_022736468.1
Bacillota	*Laceyella tengchongensis*	WP_284723808.1	WP_284724464.1	WP_333639873.1	WP_102992870.1
Bacillota	*Lihuaxuella thermophila*	WP_089971102.1	WP_244527535.1	WP_089969006.1	WP_089966921.1
Bacillota	*Longirhabdus pacifica*	WP_128893698.1	WP_128894412.1	WP_128894413.1	WP_128896012.1
Bacillota	*Marinicrinis sediminis*	WP_379927926.1	WP_379930489.1	WP_379930488.1	WP_379929885.1
Bacillota	*Marininema halotolerans*	WP_091836498.1	WP_245838860.1	WP_091838700.1	WP_176391884.1
Bacillota	*Marininema mesophilum*	WP_091742882.1	WP_091737720.1	WP_091737717.1	WP_091734794.1
Bacillota	*Marinithermofilum abyssi*	WP_188646376.1	WP_188646919.1	WP_188646918.1	WP_188648308.1
Bacillota	*Marinococcus halophilus*	WP_079476624.1	WP_079476570.1	WP_079476571.1	WP_079476090.1

Bacillota	*Marinococcus halotolerans*	WP_022792304.1	WP_022792248.1	WP_022792249.1	WP_022792687.1
Bacillota	*Marinococcus luteus*	WP_322525176.1	WP_322525124.1	WP_322525125.1	WP_091610196.1
Bacillota	*Mechercharimyces sp. CAU 1602*	WP_258839993.1	WP_258839241.1	WP_258839240.1	WP_258838868.1
Bacillota	*Melghirimyces algeriensis*	WP_142506200.1	WP_142504252.1	WP_246064816.1	WP_142504508.1
Bacillota	*Melghirimyces profundicolus*	WP_108023950.1	WP_108023223.1	WP_108023221.1	WP_108026146.1
Bacillota	*Melghirimyces thermohalophilus*	WP_091568263.1	WP_091569222.1	WP_091569219.1	WP_091565521.1
Bacillota	*Metabacillus herbersteinensis*	WP_378939256.1	WP_378932858.1	WP_378933101.1	WP_378931977.1
Bacillota	*Microaerobacter geothermalis*	WP_236404968.1	WP_236405557.1	WP_407942200.1	WP_236405793.1
Bacillota	*Novibacillus thermophilus*	WP_077719752.1	WP_077720594.1	WP_077718877.1	WP_077719370.1
Bacillota	*Paenactinomyces guangxiensis*	WP_181750949.1	WP_181751724.1	WP_309506121.1	WP_181751500.1
Bacillota	*Paenibacillus abyssi*	WP_188533017.1	WP_188528309.1	WP_188528307.1	WP_188531779.1
Bacillota	*Paenibacillus aceris*	WP_167069014.1	WP_167053849.1	WP_167053847.1	WP_167063709.1
Bacillota	*Paenibacillus aceti*	WP_120465243.1	WP_120462256.1	WP_120462257.1	WP_120462650.1
Bacillota	*Paenibacillus agilis*	WP_186786190.1	WP_144988852.1	WP_144988849.1	WP_144991373.1
Bacillota	*Paenibacillus agri*	WP_175370962.1	WP_175373667.1	WP_175373669.1	WP_175372874.1
Bacillota	*Paenibacillus agricola*	WP_166148862.1	WP_166145632.1	WP_166145635.1	WP_311764869.1
Bacillota	*Paenibacillus alba*	WP_173223509.1	WP_326071302.1	WP_326071303.1	WP_326073808.1
Bacillota	*Paenibacillus albicereus*	WP_168908529.1	WP_206110059.1	WP_168907475.1	WP_168908071.1
Bacillota	*Paenibacillus albidus*	WP_189021651.1	WP_215176829.1	WP_189024419.1	WP_215175915.1
Bacillota	*Paenibacillus albus*	WP_126015480.1	WP_126016142.1	WP_126016140.1	WP_126015742.1
Bacillota	*Paenibacillus alginolyticus*	WP_029194617.1	WP_268615698.1	WP_029193045.1	WP_268615491.1
Bacillota	*Paenibacillus algorifonticola*	WP_046229419.1	WP_046232076.1	WP_046232075.1	WP_046230403.1
Bacillota	*Paenibacillus alkaliterrae*	WP_235234645.1	WP_235234721.1	WP_235235115.1	WP_235238666.1
Bacillota	*Paenibacillus alkalitolerans*	WP_199618403.1	WP_199614925.1	WP_199614927.1	WP_199618942.1
Bacillota	*Paenibacillus allorhizoplanae*	WP_236290221.1	WP_236284611.1	WP_236285355.1	WP_236287531.1
Bacillota	*Paenibacillus allorhizosphaerae*	WP_218100378.1	WP_218100601.1	WP_218100602.1	WP_218097063.1
Bacillota	*Paenibacillus alvei*	WP_171419366.1	WP_138186444.1	WP_410453185.1	WP_268632461.1
Bacillota	*Paenibacillus amylolyticus*	WP_353883419.1	WP_123066039.1	WP_133385331.1	WP_347386579.1

Bacillota	*Paenibacillus andongensis*	WP_261304790.1	WP_261306455.1	WP_261301090.1	WP_261305761.1
Bacillota	*Paenibacillus antibioticophila*	WP_101781156.1	WP_212939118.1	WP_212939119.1	WP_044479289.1
Bacillota	*Paenibacillus apiarius*	WP_206097645.1	WP_206098075.1	WP_206098076.1	WP_206096386.1
Bacillota	*Paenibacillus apii*	WP_165099327.1	WP_165094357.1	WP_165094360.1	WP_165094978.1
Bacillota	*Paenibacillus apis*	WP_301628741.1	WP_301624040.1	WP_301627953.1	WP_301624390.1
Bacillota	*Paenibacillus arenilitoris*	WP_190863467.1	WP_190861985.1	WP_190861983.1	WP_190862815.1
Bacillota	*Paenibacillus arenosi*	WP_192026591.1	WP_192024005.1	WP_192024006.1	WP_192023195.1
Bacillota	*Paenibacillus assamensis*	WP_028594151.1	WP_028593560.1	WP_028593559.1	WP_028595832.1
Bacillota	*Paenibacillus athensensis*	WP_134756811.1	WP_134750921.1	WP_134750231.1	WP_134757304.1
Bacillota	*Paenibacillus auburnensis*	WP_236335297.1	WP_236331603.1	WP_236331605.1	WP_236332662.1
Bacillota	*Paenibacillus aurantiacus*	WP_377492888.1	WP_377500029.1	WP_377500027.1	WP_377491245.1
Bacillota	*Paenibacillus aurantius*	WP_315606699.1	WP_315603029.1	WP_315607009.1	WP_315607290.1
Bacillota	*Paenibacillus baekrokdamisoli*	WP_232016416.1	WP_125656665.1	WP_164522785.1	WP_125657417.1
Bacillota	*Paenibacillus barcinonensis*	WP_216519699.1	WP_110894264.1	WP_110894265.1	WP_110898516.1
Bacillota	*Paenibacillus barengoltzii*	WP_085279631.1	WP_016313974.1	WP_016311834.1	WP_085169625.1
Bacillota	*Paenibacillus beijingensis*	WP_045671434.1	WP_045672563.1	WP_045672564.1	WP_045671783.1
Bacillota	*Paenibacillus borealis*	WP_042217159.1	WP_042215472.1	WP_042215475.1	WP_042216727.1
Bacillota	*Paenibacillus bouchesdurhonensis*	WP_110933759.1	WP_110932522.1	WP_110932521.1	WP_110932164.1
Bacillota	*Paenibacillus brevis*	WP_216479681.1	WP_216481004.1	WP_216481005.1	WP_216481043.1
Bacillota	*Paenibacillus camerounensis*	WP_042203043.1	WP_042196114.1	WP_042204296.1	WP_042197491.1
Bacillota	*Paenibacillus castaneae*	WP_208414390.1	WP_102713920.1	WP_102710846.1	WP_102712600.1
Bacillota	*Paenibacillus caui*	WP_223066141.1	WP_223066654.1	WP_223066691.1	WP_223066358.1
Bacillota	*Paenibacillus cellulosilyticus*	WP_174812767.1	WP_110043359.1	WP_110043360.1	WP_110046471.1
Bacillota	*Paenibacillus cellulositrophicus*	WP_152398643.1	WP_251676995.1	WP_251676997.1	WP_152398424.1
Bacillota	*Paenibacillus chibensis*	WP_127606199.1	WP_328277584.1	WP_127607166.1	WP_127601684.1
Bacillota	*Paenibacillus chitinolyticus*	WP_387540779.1	WP_218229832.1	WP_377648432.1	WP_042232440.1
Bacillota	*Paenibacillus chungangensis*	WP_377568795.1	WP_377562651.1	WP_377562179.1	WP_377568441.1
Bacillota	*Paenibacillus cineris*	WP_212971249.1	WP_212984345.1	WP_212967858.1	WP_212984444.1

Bacillota	*Paenibacillus contaminans*	WP_113030359.1	WP_113033634.1	WP_113032777.1	WP_113032225.1
Bacillota	*Paenibacillus cremeus*	WP_144842443.1	WP_144842940.1	WP_261381216.1	WP_144847196.1
Bacillota	*Paenibacillus curdlanolyticus*	WP_006039766.1	WP_006039229.1	WP_006039230.1	WP_006036336.1
Bacillota	*Paenibacillus cymbidii*	WP_135553327.1	WP_135551466.1	WP_135547907.1	WP_135546844.1
Bacillota	*Paenibacillus daejeonensis*	WP_020615915.1	WP_020616645.1	WP_020616646.1	WP_020615593.1
Bacillota	*Paenibacillus dakarensis*	WP_054956193.1	WP_054957615.1	WP_054957616.1	WP_054956622.1
Bacillota	*Paenibacillus dendritiformis*	WP_133382336.1	WP_168181508.1	WP_133381197.1	WP_213469088.1
Bacillota	*Paenibacillus dendrobii*	WP_160500621.1	WP_202128686.1	WP_160498316.1	WP_160498813.1
Bacillota	*Paenibacillus dokdonensis*	WP_326089625.1	WP_136606159.1	WP_136606160.1	WP_326090758.1
Bacillota	*Paenibacillus donghaensis*	WP_087917836.1	WP_087914496.1	WP_087916894.1	WP_087917553.1
Bacillota	*Paenibacillus durus*	WP_042207934.1	WP_042205360.1	WP_042207283.1	WP_042207704.1
Bacillota	*Paenibacillus ehimensis*	WP_326059397.1	WP_127485603.1	WP_025846315.1	WP_326057552.1
Bacillota	*Paenibacillus elgii*	WP_339371850.1	WP_108531728.1	WP_339370912.1	WP_127459765.1
Bacillota	*Paenibacillus endophyticus*	WP_183557533.1	WP_183561091.1	WP_183561093.1	WP_183563669.1
Bacillota	*Paenibacillus etheri*	WP_060621774.1	WP_060622731.1	WP_060622730.1	WP_060622030.1
Bacillota	*Paenibacillus eucommiae*	WP_209975024.1	WP_209972764.1	WP_209973263.1	WP_209974125.1
Bacillota	*Paenibacillus faecalis*	WP_106768550.1	WP_106767939.1	WP_106767940.1	WP_106768318.1
Bacillota	*Paenibacillus faecis*	WP_213508219.1	WP_213504414.1	WP_213504415.1	WP_148451589.1
Bacillota	*Paenibacillus farraposensis*	WP_229523210.1	WP_229525501.1	WP_229525500.1	WP_229524566.1
Bacillota	*Paenibacillus favisporus*	WP_163881487.1	WP_163879295.1	WP_354494891.1	WP_354494692.1
Bacillota	*Paenibacillus ferrarius*	WP_079419186.1	WP_079420736.1	WP_079420735.1	WP_079420014.1
Bacillota	*Paenibacillus filicis*	WP_341418433.1	WP_341414414.1	WP_341414415.1	WP_341416827.1
Bacillota	*Paenibacillus flagellatus*	WP_110842254.1	WP_110839882.1	WP_110839883.1	WP_110840646.1
Bacillota	*Paenibacillus foliorum*	WP_171654195.1	WP_171651155.1	WP_171655783.1	WP_312886573.1
Bacillota	*Paenibacillus fonticola*	WP_019638513.1	WP_019640396.1	WP_019640395.1	WP_019636244.1
Bacillota	*Paenibacillus forsythiae*	WP_025697225.1	WP_025699187.1	WP_025699479.1	WP_025697264.1
Bacillota	*Paenibacillus frigoriresistens*	WP_173186128.1	WP_173184821.1	WP_173184605.1	WP_173186448.1
Bacillota	*Paenibacillus ginsengihumi*	WP_019532785.1	WP_019534029.1	WP_019534030.1	WP_019533053.1

Bacillota	*Paenibacillus graminis*	WP_326082249.1	WP_025707470.1	WP_025707469.1	WP_025708368.1
Bacillota	*Paenibacillus guangzhouensis*	WP_152391647.1	WP_152393925.1	WP_152393926.1	WP_265333443.1
Bacillota	*Paenibacillus gyeongsangnamensis*	WP_269881790.1	WP_269881438.1	WP_269880074.1	WP_269879583.1
Bacillota	*Paenibacillus haidiansis*	WP_331848184.1	WP_331844562.1	WP_331844563.1	WP_331844893.1
Bacillota	*Paenibacillus hamazuiensis*	WP_248927459.1	WP_248925463.1	WP_248925462.1	WP_248926615.1
Bacillota	*Paenibacillus harenae*	WP_029192613.1	WP_307201541.1	WP_028610564.1	WP_028611235.1
Bacillota	*Paenibacillus hemerocallicola*	WP_139601970.1	WP_139604983.1	WP_139604984.1	WP_139602082.1
Bacillota	*Paenibacillus herberti*	WP_342745633.1	WP_176444973.1	WP_089523697.1	WP_176444650.1
Bacillota	*Paenibacillus hexagrammi*	WP_235118391.1	WP_235118659.1	WP_235122911.1	WP_235121698.1
Bacillota	*Paenibacillus ihbetae*	WP_099478905.1	WP_077565455.1	WP_099476792.1	WP_099476993.1
Bacillota	*Paenibacillus ihuae*	WP_054942784.1	WP_054941331.1	WP_054941332.1	WP_054941758.1
Bacillota	*Paenibacillus ihumii*	WP_055109149.1	WP_055107795.1	WP_055110100.1	WP_055107462.1
Bacillota	*Paenibacillus illinoisensis*	WP_221807689.1	WP_336780754.1	WP_402875603.1	WP_337032811.1
Bacillota	*Paenibacillus jilunlii*	WP_062523374.1	WP_062519683.1	WP_062519685.1	WP_062527005.1
Bacillota	*Paenibacillus kobensis*	WP_127529797.1	WP_127530721.1	WP_127530722.1	WP_127533050.1
Bacillota	*Paenibacillus koleovorans*	WP_127578709.1	WP_127588578.1	WP_127588579.1	WP_127582757.1
Bacillota	*Paenibacillus kribbensis*	WP_094154033.1	WP_068499549.1	WP_326109516.1	WP_094154485.1
Bacillota	*Paenibacillus lacisoli*	WP_305024135.1	WP_305022224.1	WP_305022225.1	WP_305022581.1
Bacillota	*Paenibacillus larvae*	WP_077995778.1	WP_268573972.1	WP_023482678.1	WP_079940202.1
Bacillota	*Paenibacillus lautus*	WP_220685281.1	WP_127594050.1	WP_127594126.1	WP_127592034.1
Bacillota	*Paenibacillus lemnae*	WP_169504005.1	WP_169506788.1	WP_169506789.1	WP_169503480.1
Bacillota	*Paenibacillus lentus*	WP_125084497.1	WP_379392406.1	WP_379392405.1	WP_125082923.1
Bacillota	*Paenibacillus lignilyticus*	WP_210656124.1	WP_210659859.1	WP_210659857.1	WP_210659384.1
Bacillota	*Paenibacillus luteus*	WP_141504106.1	WP_141504020.1	WP_141504734.1	WP_141505818.1
Bacillota	*Paenibacillus lutimineralis*	WP_127002345.1	WP_126997891.1	WP_126997889.1	WP_126997225.1
Bacillota	*Paenibacillus lutrae*	WP_235918181.1	WP_157335440.1	WP_157335441.1	WP_157334343.1
Bacillota	*Paenibacillus lycopersici*	WP_162357517.1	WP_162356891.1	WP_162356889.1	WP_162356475.1
Bacillota	*Paenibacillus macerans*	WP_278738955.1	WP_251586340.1	WP_326045291.1	WP_328270886.1

Bacillota	*Paenibacillus marchantiophytorum*	WP_189020885.1	WP_189009728.1	WP_189014345.1	WP_189006243.1
Bacillota	*Paenibacillus massiliensis*	WP_025676521.1	WP_028589105.1	WP_025677800.1	WP_028588777.1
Bacillota	*Paenibacillus maysiensis*	WP_025685211.1	WP_025685875.1	WP_025685876.1	WP_025685742.1
Bacillota	*Paenibacillus mellifer*	WP_248552883.1	WP_248550581.1	WP_248550580.1	WP_248551931.1
Bacillota	*Paenibacillus mendelii*	WP_204817996.1	WP_204817258.1	WP_204818322.1	WP_204816513.1
Bacillota	*Paenibacillus mesophilus*	WP_138877610.1	WP_138878350.1	WP_138878085.1	WP_138882220.1
Bacillota	*Paenibacillus mesotrionivorans*	WP_416215470.1	WP_416214544.1	WP_416214545.1	WP_416219599.1
Bacillota	*Paenibacillus methanolicus*	WP_148931812.1	WP_148929581.1	WP_148928225.1	WP_148928442.1
Bacillota	*Paenibacillus montanisoli*	WP_206098550.1	WP_112882578.1	WP_112882579.1	WP_112882883.1
Bacillota	*Paenibacillus montaniterrae*	WP_213516663.1	WP_213513802.1	WP_213513264.1	WP_246563468.1
Bacillota	*Paenibacillus monticola*	WP_154121649.1	WP_154118393.1	WP_154118394.1	WP_154121344.1
Bacillota	*Paenibacillus motobuensis*	WP_343862506.1	WP_343863633.1	WP_343863631.1	WP_343865702.1
Bacillota	*Paenibacillus mucilaginosus*	WP_013920168.1	WP_013916540.1	WP_013916539.1	WP_014652281.1
Bacillota	*Paenibacillus naphthalenovorans*	WP_074727122.1	WP_175472024.1	WP_074729390.1	WP_062410860.1
Bacillota	*Paenibacillus nasutitermitis*	WP_188993163.1	WP_188992611.1	WP_188992612.1	WP_188992857.1
Bacillota	*Paenibacillus oceani*	WP_190924490.1	WP_190925107.1	WP_190925109.1	WP_190926136.1
Bacillota	*Paenibacillus odorifer*	WP_094902091.1	WP_312151840.1	WP_248548496.1	WP_076304292.1
Bacillota	*Paenibacillus oenotherae*	WP_219874260.1	WP_219870643.1	WP_219870644.1	WP_219870874.1
Bacillota	*Paenibacillus oleatilyticus*	WP_216795578.1	WP_216792181.1	WP_216792182.1	WP_216797443.1
Bacillota	*Paenibacillus oralis*	WP_128634953.1	WP_128634089.1	WP_128634090.1	WP_128634460.1
Bacillota	*Paenibacillus oryzae*	WP_068679112.1	WP_068683840.1	WP_068683842.1	WP_068684506.1
Bacillota	*Paenibacillus oryzisoli*	WP_068665391.1	WP_068670283.1	WP_068670284.1	WP_068670715.1
Bacillota	*Paenibacillus ottowii*	WP_142613378.1	WP_064796416.1	WP_326400406.1	WP_305164649.1
Bacillota	*Paenibacillus pabuli*	WP_062327955.1	WP_426251135.1	WP_347384342.1	WP_247898527.1
Bacillota	*Paenibacillus paeoniae*	WP_116042437.1	WP_116044696.1	WP_116044698.1	WP_240644312.1
Bacillota	*Paenibacillus paridis*	WP_139991027.1	WP_139999520.1	WP_238404104.1	WP_139997909.1
Bacillota	*Paenibacillus pasadenensis*	WP_251559661.1	WP_251560931.1	WP_251560930.1	WP_101809358.1
Bacillota	*Paenibacillus pectinilyticus*	WP_065851700.1	WP_065857228.1	WP_065857226.1	WP_244163060.1

Bacillota	*Paenibacillus pedocola*	WP_310829968.1	WP_310829302.1	WP_310829303.1	WP_310829725.1
Bacillota	*Paenibacillus peoriae*	WP_338532767.1	WP_010345798.1	WP_076293050.1	WP_104497052.1
Bacillota	*Paenibacillus periandrae*	WP_240414419.1	WP_240413846.1	WP_240413847.1	WP_240417094.1
Bacillota	*Paenibacillus phocaensis*	WP_068783155.1	WP_068785288.1	WP_068785287.1	WP_068784959.1
Bacillota	*Paenibacillus phyllosphaerae*	WP_183596122.1	WP_183596763.1	WP_183598251.1	WP_246427471.1
Bacillota	*Paenibacillus physcomitrellae*	WP_094096216.1	WP_094093303.1	WP_094093304.1	WP_094093583.1
Bacillota	*Paenibacillus phytohabitans*	WP_336102449.1	WP_171716664.1	WP_171716665.1	WP_171721012.1
Bacillota	*Paenibacillus pinihumi*	WP_028561893.1	WP_028561258.1	WP_028561259.1	WP_028561628.1
Bacillota	*Paenibacillus pinisoli*	WP_120107911.1	WP_120113277.1	WP_424452460.1	WP_243643891.1
Bacillota	*Paenibacillus piri*	WP_133228799.1	WP_133225913.1	WP_133225912.1	WP_342774573.1
Bacillota	*Paenibacillus piscarius*	WP_238652198.1	WP_238652982.1	WP_238652983.1	WP_238654633.1
Bacillota	*Paenibacillus plantarum*	WP_171630428.1	WP_171628794.1	WP_171629110.1	WP_171635300.1
Bacillota	*Paenibacillus planticolens*	WP_171686840.1	WP_171682055.1	WP_171682056.1	WP_246294449.1
Bacillota	*Paenibacillus plantiphilus*	WP_236346401.1	WP_236344839.1	WP_371877750.1	WP_236344089.1
Bacillota	*Paenibacillus polymyxa*	WP_330706336.1	WP_290402692.1	WP_194843217.1	WP_013311101.1
Bacillota	*Paenibacillus polysaccharolyticus*	WP_251508739.1	WP_251505656.1	WP_251505659.1	WP_253492605.1
Bacillota	*Paenibacillus popilliae*	WP_142545952.1	WP_006285465.1	WP_142543324.1	WP_006286443.1
Bacillota	*Paenibacillus prosopidis*	WP_114382400.1	WP_114382545.1	WP_114378576.1	WP_114381166.1
Bacillota	*Paenibacillus pseudetheri*	WP_234537788.1	WP_234531948.1	WP_234531942.1	WP_234533213.1
Bacillota	*Paenibacillus psychroresistens*	WP_155702999.1	WP_155701927.1	WP_155704866.1	WP_155700801.1
Bacillota	*Paenibacillus puerhi*	WP_159881110.1	WP_159883059.1	WP_159883057.1	WP_235941410.1
Bacillota	*Paenibacillus puldeungensis*	WP_379321167.1	WP_379321196.1	WP_379321195.1	WP_379318034.1
Bacillota	*Paenibacillus qinlingensis*	WP_173115243.1	WP_173110236.1	WP_173110235.1	WP_310226997.1
Bacillota	*Paenibacillus radicibacter*	WP_258208379.1	WP_258206429.1	WP_258206430.1	WP_258206078.1
Bacillota	*Paenibacillus radicis*	WP_258211272.1	WP_258216056.1	WP_258216055.1	WP_258216523.1
Bacillota	*Paenibacillus rhizophilus*	WP_124695119.1	WP_124693619.1	WP_124693618.1	WP_124695680.1
Bacillota	*Paenibacillus rhizoplanae*	WP_209993516.1	WP_379256890.1	WP_209993887.1	WP_209987956.1
Bacillota	*Paenibacillus rhizosphaerae*	WP_183587077.1	WP_183581975.1	WP_183581974.1	WP_183581722.1

Bacillota	*Paenibacillus rigui*	WP_094014449.1	WP_094013285.1	WP_094013286.1	WP_094015209.1
Bacillota	*Paenibacillus riograndensis*	WP_020430154.1	WP_060861783.1	WP_060861782.1	WP_020427852.1
Bacillota	*Paenibacillus sabinae*	WP_025336118.1	WP_025335550.1	WP_025335551.1	WP_025335875.1
Bacillota	*Paenibacillus sabuli*	WP_223869387.1	WP_190916452.1	WP_190916454.1	WP_190915075.1
Bacillota	*Paenibacillus sambharensis*	WP_181438790.1	WP_111149231.1	WP_111149230.1	WP_111148711.1
Bacillota	*Paenibacillus sanguinis*	WP_018752292.1	WP_018751371.1	WP_018751372.1	WP_018753264.1
Bacillota	*Paenibacillus sedimenti*	WP_188175441.1	WP_188177267.1	WP_188174343.1	WP_188172947.1
Bacillota	*Paenibacillus selenitireducens*	WP_078497635.1	WP_078497211.1	WP_078497210.1	WP_078498745.1
Bacillota	*Paenibacillus senegalensis*	WP_010271006.1	WP_026021375.1	WP_010273795.1	WP_010270149.1
Bacillota	*Paenibacillus senegalimassiliensis*	WP_059049591.1	WP_059052566.1	WP_059052564.1	WP_059052170.1
Bacillota	*Paenibacillus septentrionalis*	WP_379231073.1	WP_379233282.1	WP_379232422.1	WP_379230547.1
Bacillota	*Paenibacillus sepulcri*	WP_210046909.1	WP_210041358.1	WP_210045727.1	WP_210037664.1
Bacillota	*Paenibacillus silagei*	WP_209876089.1	WP_209874571.1	WP_209869954.1	WP_209871997.1
Bacillota	*Paenibacillus silvae*	WP_248063624.1	WP_111271384.1	WP_111271385.1	WP_111271909.1
Bacillota	*Paenibacillus silvestris*	WP_235960584.1	WP_161408218.1	WP_161408564.1	WP_235959055.1
Bacillota	*Paenibacillus silvisoli*	WP_308638710.1	WP_308639168.1	WP_308639167.1	WP_308638903.1
Bacillota	*Paenibacillus silviterrae*	WP_284644809.1	WP_284643983.1	WP_284640174.1	WP_284638745.1
Bacillota	*Paenibacillus sinopodophylli*	WP_342778467.1	WP_138750981.1	WP_138750980.1	WP_138751928.1
Bacillota	*Paenibacillus solanacearum*	WP_218092977.1	WP_218091182.1	WP_218091183.1	WP_218090647.1
Bacillota	*Paenibacillus solisilvae*	WP_379190145.1	WP_379189360.1	WP_379191972.1	WP_379190327.1
Bacillota	*Paenibacillus sonchi*	WP_039838104.1	WP_039835063.1	WP_039835064.1	WP_233184314.1
Bacillota	*Paenibacillus sophorae*	WP_036598261.1	WP_036594357.1	WP_036594356.1	WP_036596753.1
Bacillota	*Paenibacillus sp.*	WP_309122791.1	WP_309118627.1	WP_309118628.1	WP_421885775.1
Bacillota	*Paenibacillus spongiae*	WP_258387971.1	WP_258387583.1	WP_258388477.1	WP_258388242.1
Bacillota	*Paenibacillus stellifer*	WP_038698104.1	WP_038697001.1	WP_038697002.1	WP_038697658.1
Bacillota	*Paenibacillus swuensis*	WP_068607342.1	WP_068605796.1	WP_068604930.1	WP_231891263.1
Bacillota	*Paenibacillus taichungensis*	WP_113055901.1	WP_175382996.1	WP_413372315.1	WP_113053843.1
Bacillota	*Paenibacillus taihuensis*	WP_116189947.1	WP_181909822.1	WP_116187052.1	WP_116187460.1

Bacillota	*Paenibacillus taiwanensis*	WP_028547052.1	WP_028544285.1	WP_028544284.1	WP_245596021.1
Bacillota	*Paenibacillus tarimensis*	WP_235287205.1	WP_235285858.1	WP_235285857.1	WP_235285404.1
Bacillota	*Paenibacillus tengchongensis*	WP_151736597.1	WP_151734500.1	WP_151734499.1	WP_151735851.1
Bacillota	*Paenibacillus tepidiphilus*	WP_150269544.1	WP_150273581.1	WP_150273580.1	WP_150266292.1
Bacillota	*Paenibacillus terrae*	WP_145163154.1	WP_014282065.1	WP_014282066.1	WP_149093315.1
Bacillota	*Paenibacillus terricola*	WP_191201982.1	WP_191203123.1	WP_191203122.1	WP_191202242.1
Bacillota	*Paenibacillus terrigena*	WP_314587033.1	WP_018755349.1	WP_314585441.1	WP_018756691.1
Bacillota	*Paenibacillus thalictri*	WP_170318199.1	WP_131012956.1	WP_131012957.1	WP_131014125.1
Bacillota	*Paenibacillus thermoaerophilus*	WP_138788696.1	WP_138787912.1	WP_138787913.1	WP_170209381.1
Bacillota	*Paenibacillus thiaminolyticus*	WP_119796028.1	WP_272045015.1	WP_119792949.1	WP_087442146.1
Bacillota	*Paenibacillus tianjinensis*	WP_206101756.1	WP_206100817.1	WP_206100818.1	WP_206101483.1
Bacillota	*Paenibacillus tianmuensis*	WP_090669484.1	WP_090665888.1	WP_090665886.1	WP_090670061.1
Bacillota	*Paenibacillus timonensis*	WP_240271378.1	WP_240268406.1	WP_240268405.1	WP_240269054.1
Bacillota	*Paenibacillus tritici*	WP_173132030.1	WP_173129013.1	WP_211720201.1	WP_173128240.1
Bacillota	*Paenibacillus tundrae*	WP_307218044.1	WP_307212880.1	WP_307212877.1	WP_338540183.1
Bacillota	*Paenibacillus turicensis*	WP_210088697.1	WP_210091285.1	WP_210091286.1	WP_210088043.1
Bacillota	*Paenibacillus turpanensis*	WP_166240415.1	WP_166239436.1	WP_166239434.1	WP_166243811.1
Bacillota	*Paenibacillus typhae*	WP_090714882.1	WP_090714162.1	WP_221798010.1	WP_090712285.1
Bacillota	*Paenibacillus tyrfis*	WP_281937182.1	WP_036680591.1	WP_253409447.1	WP_253412858.1
Bacillota	*Paenibacillus uliginis*	WP_208915349.1	WP_208914519.1	WP_208914520.1	WP_208915055.1
Bacillota	*Paenibacillus vandeheii*	WP_301248911.1	WP_301246512.1	WP_301246510.1	WP_301243803.1
Bacillota	*Paenibacillus vietnamensis*	WP_224723386.1	WP_224723467.1	WP_224722472.1	WP_224721890.1
Bacillota	*Paenibacillus whitsoniae*	WP_126145122.1	WP_126140510.1	WP_126140511.1	WP_126143135.1
Bacillota	*Paenibacillus woosongensis*	WP_283927468.1	WP_155610100.1	WP_330163346.1	WP_283928465.1
Bacillota	*Paenibacillus wynnii*	WP_307589484.1	WP_036657105.1	WP_307586656.1	WP_036657888.1
Bacillota	*Paenibacillus xanthanilyticus*	WP_377721381.1	WP_377722267.1	WP_377722268.1	WP_377722477.1
Bacillota	*Paenibacillus xerothermodurans*	WP_089200934.1	WP_089199061.1	WP_089199062.1	WP_243633136.1
Bacillota	*Paenibacillus xylanexedens*	WP_315370738.1	WP_145047451.1	WP_145408863.1	WP_124116014.1

Bacillota	*Paenibacillus xylaniclasticus*	WP_127572600.1	WP_127566875.1	WP_127566877.1	WP_127567498.1
Bacillota	*Paenibacillus xylanilyticus*	WP_175394430.1	WP_413035370.1	WP_175397877.1	WP_175397529.1
Bacillota	*Paenibacillus xylanivorans*	WP_053783136.1	WP_053780711.1	WP_053780710.1	WP_053781229.1
Bacillota	*Paenibacillus yonginensis*	WP_068697326.1	WP_068695883.1	WP_068695885.1	WP_068696453.1
Bacillota	*Paenibacillus zanthoxyli*	WP_025693164.1	WP_025692677.1	WP_025692678.1	WP_025691633.1
Bacillota	*Paludifilum halophilum*	WP_169713717.1	WP_094264071.1	WP_094262790.1	WP_240511973.1
Bacillota	*Peribacillus butanolivorans*	WP_340455904.1	WP_379155062.1	WP_379155065.1	WP_387587241.1
Bacillota	*Peribacillus castrilensis*	WP_318291301.1	WP_367409646.1	WP_367407233.1	WP_367407051.1
Bacillota	*Peribacillus frigoritolerans*	WP_367235604.1	WP_349728053.1	WP_286166049.1	WP_342615343.1
Bacillota	*Peribacillus simplex*	WP_137020667.1	WP_061144265.1	WP_061144266.1	WP_061144059.1
Bacillota	*Planifilum fimeticola*	WP_106345446.1	WP_106345631.1	WP_106345581.1	WP_106344368.1
Bacillota	*Planifilum fulgidum*	WP_177199077.1	WP_092035968.1	WP_092035967.1	WP_092041673.1
Bacillota	*Planococcus antarcticus*	WP_006830683.1	WP_040852325.1	WP_006829302.1	WP_006828840.1
Bacillota	*Polycladomyces abyssicola*	WP_212774188.1	WP_212772256.1	WP_212772255.1	WP_212772617.1
Bacillota	*Polycladomyces subterraneus*	WP_301240606.1	WP_301237983.1	WP_301237984.1	WP_301239880.1
Bacillota	*Polycladospora coralii*	WP_191139217.1	WP_347239741.1	WP_191138248.1	WP_191140021.1
Bacillota	*Saccharibacillus alkalitolerans*	WP_166276593.1	WP_166272347.1	WP_166272345.1	WP_166271645.1
Bacillota	*Saccharibacillus deserti*	WP_172254369.1	WP_172250470.1	WP_172250468.1	WP_172249784.1
Bacillota	*Saccharibacillus endophyticus*	WP_172244410.1	WP_172239876.1	WP_172239879.1	WP_172247082.1
Bacillota	*Saccharibacillus kuerlensis*	WP_018978303.1	WP_018976427.1	WP_018976426.1	WP_018975684.1
Bacillota	*Saccharibacillus qingshengii*	WP_172196605.1	WP_172196256.1	WP_172196255.1	WP_172194137.1
Bacillota	*Saccharibacillus sp. O23*	WP_088488869.1	WP_088489527.1	WP_088489528.1	WP_088489347.1
Bacillota	*Salibacterium aidingense*	WP_026700176.1	WP_026700929.1	WP_422787443.1	WP_422785923.1
Bacillota	*Salibacterium halotolerans*	WP_093335510.1	WP_093335998.1	WP_093335997.1	WP_212634930.1
Bacillota	*Salibacterium lacus*	WP_380711669.1	WP_380711740.1	WP_380711739.1	WP_380712172.1
Bacillota	*Salibacterium qingdaonense*	WP_177195506.1	WP_090925891.1	WP_090925890.1	WP_177195403.1
Bacillota	*Salibacterium salarium*	WP_125557516.1	WP_125555844.1	WP_348639443.1	WP_306897393.1
Bacillota	*Salinithrix halophila*	WP_380702635.1	WP_380705887.1	WP_380705888.1	WP_380703580.1

Bacillota	*Salipaludibacillus sp. CUR1*	WP_230896231.1	WP_230896188.1	WP_230896189.1	WP_230898551.1
Bacillota	*Salsuginibacillus halophilus*	WP_106587779.1	WP_106587852.1	WP_106587851.1	WP_106587505.1
Bacillota	*Salsuginibacillus kocurii*	WP_018922183.1	WP_018922278.1	WP_018922277.1	WP_018921352.1
Bacillota	*Sinobaca qinghaiensis*	WP_120193293.1	WP_120193227.1	WP_120193228.1	WP_120192335.1
Bacillota	*Sutcliffiella horikoshii*	WP_148990256.1	WP_226683573.1	WP_404428349.1	WP_187441928.1
Bacillota	*Thermoactinomyces sp. DSM 45891*	WP_072336567.1	WP_072329871.1	WP_072329873.1	WP_072331764.1
Bacillota	*Thermoflavimicrobium daqui*	WP_113657606.1	WP_113660335.1	WP_113660334.1	WP_113658619.1
Bacillota	*Thermoflavimicrobium dichotomicum*	WP_093228129.1	WP_093230852.1	WP_093230851.1	WP_175482318.1
Bacillota	*Virgibacillus tibetensis*	WP_327609447.1	WP_327606045.1	WP_327608938.1	WP_327606340.1
Bacillota	*Xylanibacillus composti*	WP_244865042.1	WP_213410179.1	WP_213410178.1	WP_213410594.1
Bacteroidota	*Acidiluteibacter ferrifornacis*	WP_160633441.1	WP_160634009.1	WP_160633238.1	WP_160632436.1
Bacteroidota	*Adhaeribacter arboris*	WP_106931386.1	WP_106925362.1	WP_106929784.1	WP_106933576.1
Bacteroidota	*Adhaeribacter pallidiroseus*	WP_115374736.1	WP_115373291.1	WP_115373435.1	WP_115375762.1
Bacteroidota	*Adhaeribacter radiodurans*	WP_246343606.1	WP_182414787.1	WP_182412160.1	WP_182414468.1
Bacteroidota	*Adhaeribacter soli*	WP_150903137.1	WP_150904862.1	WP_150905911.1	WP_150903363.1
Bacteroidota	*Adhaeribacter swui*	WP_185273551.1	WP_185270322.1	WP_185274196.1	WP_185272839.1
Bacteroidota	*Adhaeribacter terreus*	WP_378017384.1	WP_378015753.1	WP_378016465.1	WP_378017653.1
Bacteroidota	*Adhaeribacter terrigena*	WP_200506402.1	WP_200504364.1	WP_200505621.1	WP_200506036.1
Bacteroidota	*Aequorivita antarctica*	WP_111843961.1	WP_111843834.1	WP_111845043.1	WP_111844811.1
Bacteroidota	*Aequorivita aquimaris*	WP_062623013.1	WP_062621430.1	WP_062619548.1	WP_062619660.1
Bacteroidota	*Aequorivita aurantiaca*	WP_290253495.1	WP_290253605.1	WP_290255165.1	WP_290255086.1
Bacteroidota	*Aequorivita capsosiphonis*	WP_026451993.1	WP_026449899.1	WP_026451927.1	WP_026450721.1
Bacteroidota	*Aequorivita ciconiae*	WP_128249725.1	WP_128249060.1	WP_128249027.1	WP_128251637.1
Bacteroidota	*Aequorivita lipolytica*	WP_111816085.1	WP_111815969.1	WP_111816248.1	WP_111816891.1
Bacteroidota	*Aequorivita marina*	WP_310993630.1	WP_310991922.1	WP_310992157.1	WP_310993816.1
Bacteroidota	*Aequorivita nionensis*	WP_410005368.1	WP_410005228.1	WP_410004785.1	WP_410007104.1
Bacteroidota	*Aequorivita soesokkakensis*	WP_068761931.1	WP_068760878.1	WP_068761363.1	WP_068760990.1
Bacteroidota	*Aequorivita sp. KMM 9714*	WP_165150480.1	WP_165147784.1	WP_165149664.1	WP_165150353.1

Bacteroidota	*Aequorivita sublithincola*	WP_042492077.1	WP_014783441.1	WP_014781031.1	WP_014780829.1
Bacteroidota	*Aequorivita todarodis*	WP_272854795.1	WP_272854907.1	WP_272856410.1	WP_272856446.1
Bacteroidota	*Aequorivita viscosa*	WP_073214506.1	WP_073219450.1	WP_073221462.1	WP_073214181.1
Bacteroidota	*Aequorivita vitellina*	WP_237602696.1	WP_237602884.1	WP_237604082.1	WP_237604065.1
Bacteroidota	*Aequorivita vladivostokensis*	WP_045081596.1	WP_045080904.1	WP_045080108.1	WP_045081657.1
Bacteroidota	*Aequorivita xiaoshiensis*	WP_237609006.1	WP_237607055.1	WP_237608260.1	WP_237608850.1
Bacteroidota	*Aestuariibaculum lutulentum*	WP_240573145.1	WP_240572916.1	WP_240574573.1	WP_240573340.1
Bacteroidota	*Aestuariibaculum marinum*	WP_188222212.1	WP_188223804.1	WP_188221776.1	WP_188222367.1
Bacteroidota	*Aestuariibaculum sediminum*	WP_188229559.1	WP_188229943.1	WP_188229495.1	WP_188230458.1
Bacteroidota	*Aestuariibaculum sp. M13*	WP_258228506.1	WP_258227523.1	WP_258227337.1	WP_258228345.1
Bacteroidota	*Aestuariibaculum suncheonense*	WP_188216222.1	WP_188215830.1	WP_188215657.1	WP_188216389.1
Bacteroidota	*Aestuariivivens insulae*	WP_242205529.1	WP_242204282.1	WP_242206092.1	WP_242205612.1
Bacteroidota	*Aestuariivivens marinum*	WP_242132968.1	WP_242132290.1	WP_242135394.1	WP_242135503.1
Bacteroidota	*Aestuariivivens sediminicola*	WP_242093504.1	WP_242120336.1	WP_242121371.1	WP_242093034.1
Bacteroidota	*Aestuariivivens sediminis*	WP_242084579.1	WP_242086932.1	WP_242158599.1	WP_242157877.1
Bacteroidota	*Aestuariivivens sp. NBU2969*	WP_223548038.1	WP_223548296.1	WP_223551508.1	WP_223552062.1
Bacteroidota	*Agaribacillus aureus*	WP_346761610.1	WP_346757862.1	WP_346755833.1	WP_346761836.1
Bacteroidota	*Albibacterium bauzanense*	WP_132221933.1	WP_132222972.1	WP_132221473.1	WP_132222344.1
Bacteroidota	*Albibacterium sp.*	WP_325360523.1	WP_325357452.1	WP_325358942.1	WP_325359824.1
Bacteroidota	*Algibacter agarivorans*	WP_345193075.1	WP_345189876.1	WP_345193043.1	WP_345193815.1
Bacteroidota	*Algibacter amylolyticus*	WP_144117106.1	WP_144118081.1	WP_144117049.1	WP_144116980.1
Bacteroidota	*Algibacter aquimarinus*	WP_345169948.1	WP_345169810.1	WP_345170338.1	WP_345169783.1
Bacteroidota	*Algibacter lectus*	WP_074940410.1	WP_042494898.1	WP_042497935.1	WP_074936115.1
Bacteroidota	*Algibacter luteus*	WP_019387202.1	WP_289194008.1	WP_019387259.1	WP_289196303.1
Bacteroidota	*Algibacter marinivivus*	WP_109353024.1	WP_109353276.1	WP_109353074.1	WP_109353815.1
Bacteroidota	*Algibacter mikhailovii*	WP_189361399.1	WP_189358466.1	WP_189362427.1	WP_282124090.1
Bacteroidota	*Algibacter miyuki*	WP_290273055.1	WP_290273454.1	WP_290271960.1	WP_290273464.1
Bacteroidota	*Algibacter pacificus*	WP_147677447.1	WP_147678726.1	WP_147678533.1	WP_147678795.1

Bacteroidota	*Algibacter pectinivorans*	WP_092852296.1	WP_092853154.1	WP_092852392.1	WP_092851984.1
Bacteroidota	*Algibacter sp.*	WP_300249043.1	WP_300252120.1	WP_348451855.1	WP_348451771.1
Bacteroidota	*Algivirga pacifica*	WP_345370542.1	WP_345373764.1	WP_345373213.1	WP_345371319.1
Bacteroidota	*Algoriella sp.*	WP_332546600.1	WP_332544110.1	WP_332546255.1	WP_287443154.1
Bacteroidota	*Allotamlana fucoidanivorans*	WP_139698527.1	WP_139694982.1	WP_139698433.1	WP_139696462.1
Bacteroidota	*Altibacter sp.*	WP_290625980.1	WP_288003297.1	WP_290628138.1	WP_290627392.1
Bacteroidota	*Anditalea andensis*	WP_035075125.1	WP_035075984.1	WP_035072741.1	WP_035075153.1
Bacteroidota	*Apibacter adventoris*	WP_105192749.1	WP_105193923.1	WP_181042806.1	WP_105193663.1
Bacteroidota	*Apibacter mensalis*	WP_303812563.1	WP_303821584.1	WP_055425757.1	WP_303812704.1
Bacteroidota	*Apibacter muscae*	WP_146310081.1	WP_146261981.1	WP_146261740.1	WP_146311829.1
Bacteroidota	*Apibacter sp.*	WP_293918652.1	WP_293917054.1	WP_293918312.1	WP_293918330.1
Bacteroidota	*Aquaticitalea lipolytica*	WP_283635384.1	WP_188605702.1	WP_283635546.1	WP_188606921.1
Bacteroidota	*Aquimarina acroporae*	WP_248333197.1	WP_248325971.1	WP_248327184.1	WP_248329473.1
Bacteroidota	*Aquimarina addita*	WP_344929695.1	WP_344928121.1	WP_344926945.1	WP_344927438.1
Bacteroidota	*Aquimarina agarilytica*	WP_010182218.1	WP_010182235.1	WP_010178350.1	WP_010182594.1
Bacteroidota	*Aquimarina agarivorans*	WP_010520948.1	WP_010521832.1	WP_010522891.1	WP_010520711.1
Bacteroidota	*Aquimarina algicola*	WP_140588301.1	WP_140589169.1	WP_140595569.1	WP_140595083.1
Bacteroidota	*Aquimarina amphilecti*	WP_091403738.1	WP_091404647.1	WP_091403964.1	WP_091410641.1
Bacteroidota	*Aquimarina aquimarini*	WP_234859132.1	WP_108869272.1	WP_108868242.1	WP_108868066.1
Bacteroidota	*Aquimarina atlantica*	WP_051575609.1	WP_034238049.1	WP_034241498.1	WP_034246824.1
Bacteroidota	*Aquimarina brevivitae*	WP_130287912.1	WP_130286860.1	WP_130287960.1	WP_130286092.1
Bacteroidota	*Aquimarina celericrescens*	WP_378321902.1	WP_378318285.1	WP_378318615.1	WP_378321627.1
Bacteroidota	*Aquimarina gracilis*	WP_324181944.1	WP_324180621.1	WP_324180101.1	WP_324178525.1
Bacteroidota	*Aquimarina intermedia*	WP_148783869.1	WP_148782085.1	WP_148782431.1	WP_148783651.1
Bacteroidota	*Aquimarina litoralis*	WP_219009173.1	WP_343912639.1	WP_299600296.1	WP_343913483.1
Bacteroidota	*Aquimarina longa*	WP_062055130.1	WP_062057664.1	WP_062058925.1	WP_062059664.1
Bacteroidota	*Aquimarina macrocephali*	WP_024772579.1	WP_024770031.1	WP_024771131.1	WP_024771276.1
Bacteroidota	*Aquimarina muelleri*	WP_027413955.1	WP_027411125.1	WP_027411711.1	WP_027411300.1

Bacteroidota	*Aquimarina mytili*	WP_201924405.1	WP_201917632.1	WP_201924142.1	WP_201922323.1
Bacteroidota	*Aquimarina pacifica*	WP_025740165.1	WP_025742060.1	WP_025743084.1	WP_025742517.1
Bacteroidota	*Aquimarina rubra*	WP_378288611.1	WP_378290376.1	WP_378288455.1	WP_378295432.1
Bacteroidota	*Aquimarina sediminis*	WP_103067938.1	WP_103068351.1	WP_103070573.1	WP_103070728.1
Bacteroidota	*Aquimarina sp. 2-A2*	WP_407140247.1	WP_407139263.1	WP_407140005.1	WP_407139823.1
Bacteroidota	*Aquimarina spongiae*	WP_073314334.1	WP_073314409.1	WP_073314355.1	WP_073320496.1
Bacteroidota	*Aquirufa antheringensis*	WP_130895386.1	WP_130922775.1	WP_269001602.1	WP_269001114.1
Bacteroidota	*Aquirufa aurantiipilula*	WP_223144145.1	WP_223142915.1	WP_276343803.1	WP_223143335.1
Bacteroidota	*Aquirufa avitistagni*	WP_377983087.1	WP_377983723.1	WP_377982913.1	WP_377983456.1
Bacteroidota	*Aquirufa beregesia*	WP_166229642.1	WP_166230096.1	WP_166228982.1	WP_166229514.1
Bacteroidota	*Aquirufa ecclesiirivi*	WP_166373820.1	WP_166374907.1	WP_166375689.1	WP_422184414.1
Bacteroidota	*Aquirufa echingensis*	WP_377974724.1	WP_377976744.1	WP_377976516.1	WP_377974603.1
Bacteroidota	*Aquirufa lenticrescens*	WP_223131557.1	WP_223131088.1	WP_223130902.1	WP_223129962.1
Bacteroidota	*Aquirufa nivalisilvae*	WP_109324317.1	WP_109322091.1	WP_269024951.1	WP_130924003.1
Bacteroidota	*Aquirufa novilacunae*	WP_406778192.1	WP_406800710.1	WP_406800059.1	WP_406800572.1
Bacteroidota	*Aquirufa originis*	WP_377978463.1	WP_377979066.1	WP_377978324.1	WP_377978916.1
Bacteroidota	*Aquirufa regiilacus*	WP_315574943.1	WP_315576891.1	WP_315575113.1	WP_316070539.1
Bacteroidota	*Aquirufa rosea*	WP_129027395.1	WP_129026517.1	WP_129027631.1	WP_129027468.1
Bacteroidota	*Aquirufa salirivi*	WP_406750953.1	WP_406749496.1	WP_406751867.1	WP_406750838.1
Bacteroidota	*Aquirufa sp.*	WP_395767647.1	WP_395617283.1	WP_395767809.1	WP_395616701.1
Bacteroidota	*Arcticibacter eurypsychrophilus*	WP_069659817.1	WP_069660197.1	WP_069658220.1	WP_069658143.1
Bacteroidota	*Arcticibacter pallidicorallinus*	WP_106290857.1	WP_106295235.1	WP_106293321.1	WP_106293700.1
Bacteroidota	*Arcticibacter sp.*	WP_407430282.1	WP_407426349.1	WP_407428255.1	WP_407430250.1
Bacteroidota	*Arcticibacter svalbardensis*	WP_016196807.1	WP_016197349.1	WP_016193496.1	WP_016195818.1
Bacteroidota	*Arcticibacter tournemirensis*	WP_128768377.1	WP_128770802.1	WP_128769017.1	WP_128771266.1
Bacteroidota	*Aridibaculum aurantiacum*	WP_207493959.1	WP_207496171.1	WP_207493540.1	WP_207493862.1
Bacteroidota	*Ascidiimonas aurantiaca*	WP_340067019.1	WP_340063238.1	WP_340065951.1	WP_340063664.1
Bacteroidota	*Ascidiimonas meishanensis*	WP_340202765.1	WP_340202346.1	WP_340201815.1	WP_340203336.1

Bacteroidota	*Asinibacterium sp. OR53*	WP_026770568.1	WP_026768116.1	WP_026770043.1	WP_026770422.1
Bacteroidota	*Asprobacillus argus*	WP_349241159.1	WP_349240976.1	WP_349241222.1	WP_349242390.1
Bacteroidota	*Aurantibacillus circumpalustris*	WP_317898783.1	WP_317897266.1	WP_317899147.1	WP_317898617.1
Bacteroidota	*Aurantibacter aestuarii*	WP_181256232.1	WP_106463421.1	WP_106463824.1	WP_106463759.1
Bacteroidota	*Aurantibacter sp.*	WP_375238534.1	WP_375239262.1	WP_348149237.1	WP_375239007.1
Bacteroidota	*Aureibacter tunicatorum*	WP_309938190.1	WP_309937555.1	WP_309941955.1	WP_309937614.1
Bacteroidota	*Aureibaculum algae*	WP_138949641.1	WP_138950357.1	WP_138950222.1	WP_138948862.1
Bacteroidota	*Aureibaculum flavum*	WP_198841374.1	WP_198839813.1	WP_198839693.1	WP_198842913.1
Bacteroidota	*Aureibaculum luteum*	WP_117880577.1	WP_117883329.1	WP_117883048.1	WP_117885144.1
Bacteroidota	*Aureibaculum marinum*	WP_123899280.1	WP_123896962.1	WP_123897081.1	WP_123896527.1
Bacteroidota	*Aureibaculum sp. 2308TA14-22*	WP_354204459.1	WP_354205576.1	WP_354205365.1	WP_354206002.1
Bacteroidota	*Aureisphaera galaxeae*	WP_272862643.1	WP_272862417.1	WP_272863078.1	WP_272861625.1
Bacteroidota	*Aureispira anguillae*	WP_264788052.1	WP_264788426.1	WP_264791552.1	WP_264791511.1
Bacteroidota	*Aureitalea sp. L0-47*	WP_265200652.1	WP_265198881.1	WP_265199191.1	WP_265200177.1
Bacteroidota	*Aureivirga marina*	WP_196892920.1	WP_196892692.1	WP_196894274.1	WP_196894243.1
Bacteroidota	*Aureivirga sp. CE67*	WP_196886436.1	WP_196888611.1	WP_196888714.1	WP_196887798.1
Bacteroidota	*Bergeyella cardium*	WP_120489054.1	WP_120489413.1	WP_120488367.1	WP_120488308.1
Bacteroidota	*Bergeyella porcorum*	WP_376834211.1	WP_327985048.1	WP_327984028.1	WP_412677604.1
Bacteroidota	*Bergeyella sp. RCAD1439*	WP_327609825.1	WP_327609899.1	WP_327610773.1	WP_327610132.1
Bacteroidota	*Bergeyella zoohelcum*	WP_002664476.1	WP_394265388.1	WP_002661560.1	WP_002662668.1
Bacteroidota	*Bernardetia litoralis*	WP_014798351.1	WP_014799449.1	WP_014796014.1	WP_014797464.1
Bacteroidota	*Bernardetia sp.*	WP_291723737.1	WP_291727868.1	WP_291727328.1	WP_291721233.1
Bacteroidota	*Bizionia arctica*	WP_188464709.1	WP_188463855.1	WP_188463365.1	WP_188465515.1
Bacteroidota	*Bizionia argentinensis*	WP_008636277.1	WP_008634538.1	WP_008636439.1	WP_008637444.1
Bacteroidota	*Bizionia echini*	WP_092208682.1	WP_092207375.1	WP_092206879.1	WP_092209708.1
Bacteroidota	*Bizionia hallyeonensis*	WP_376860896.1	WP_376859852.1	WP_376859305.1	WP_376860208.1
Bacteroidota	*Bizionia myxarmorum*	WP_148403551.1	WP_148404171.1	WP_148405154.1	WP_148403799.1
Bacteroidota	*Bizionia paragorgiae*	WP_092132826.1	WP_318908187.1	WP_092135828.1	WP_318907152.1

Bacteroidota	*Bizionia sediminis*	WP_376894251.1	WP_376891938.1	WP_376891554.1	WP_376891096.1
Bacteroidota	*Bizionia sp.*	WP_417196395.1	WP_417195014.1	WP_417199749.1	WP_417238594.1
Bacteroidota	*Botryobacter ruber*	WP_114782892.1	WP_114781647.1	WP_114781543.1	WP_240676079.1
Bacteroidota	*Brumimicrobium aurantiacum*	WP_116880677.1	WP_116880684.1	WP_116880515.1	WP_116881141.1
Bacteroidota	*Brumimicrobium glaciale*	WP_130094390.1	WP_130094022.1	WP_242494536.1	WP_165366249.1
Bacteroidota	*Brumimicrobium mesophilum*	WP_107039253.1	WP_107040258.1	WP_107039225.1	WP_107039918.1
Bacteroidota	*Brumimicrobium oceani*	WP_109360571.1	WP_109358790.1	WP_233244208.1	WP_109360517.1
Bacteroidota	*Brumimicrobium salinarum*	WP_101334249.1	WP_101335045.1	WP_101334281.1	WP_101335294.1
Bacteroidota	*Brumimicrobium sp.*	WP_417266258.1	WP_417265319.1	WP_417266280.1	WP_417265785.1
Bacteroidota	*Candidatus Kaistella beijingensis*	WP_224136972.1	WP_224135010.1	WP_224134386.1	WP_224134929.1
Bacteroidota	*Candidatus Ulvibacter alkanivorans*	WP_114490672.1	WP_114491340.1	WP_114491714.1	WP_114491961.1
Bacteroidota	*Cesiribacter sp. SM1*	WP_370634655.1	WP_224999480.1	WP_224998922.1	WP_224999441.1
Bacteroidota	*Changchengzhania lutea*	WP_142785652.1	WP_142785956.1	WP_142783246.1	WP_142783659.1
Bacteroidota	*Chishuiella changwenlii*	WP_072930933.1	WP_072931684.1	WP_072930379.1	WP_143147299.1
Bacteroidota	*Chishuiella sp.*	WP_313376097.1	WP_313385780.1	WP_313386052.1	WP_313374642.1
Bacteroidota	*Chitinophaga agri*	WP_238429994.1	WP_162333742.1	WP_162331142.1	WP_238430125.1
Bacteroidota	*Chitinophaga agrisoli*	WP_149839302.1	WP_149836969.1	WP_149837932.1	WP_149840340.1
Bacteroidota	*Chitinophaga alhagiae*	WP_119078929.1	WP_119077216.1	WP_119079661.1	WP_157986662.1
Bacteroidota	*Chitinophaga arvensicola*	WP_089902623.1	WP_089889289.1	WP_089896020.1	WP_177192308.1
Bacteroidota	*Chitinophaga barathri*	WP_120516762.1	WP_120518200.1	WP_120514463.1	WP_162946241.1
Bacteroidota	*Chitinophaga caeni*	WP_098193960.1	WP_098192515.1	WP_098194845.1	WP_157760992.1
Bacteroidota	*Chitinophaga caseinilytica*	WP_423737646.1	WP_341842633.1	WP_341839141.1	WP_423734991.1
Bacteroidota	*Chitinophaga costaii*	WP_089708494.1	WP_089715512.1	WP_089710726.1	WP_089715615.1
Bacteroidota	*Chitinophaga cymbidii*	WP_146862438.1	WP_146858207.1	WP_146865249.1	WP_186831128.1
Bacteroidota	*Chitinophaga defluvii*	WP_354663379.1	WP_354661261.1	WP_354660547.1	WP_354659270.1
Bacteroidota	*Chitinophaga deserti*	WP_109698414.1	WP_109694755.1	WP_109699103.1	WP_157962811.1
Bacteroidota	*Chitinophaga dinghuensis*	WP_245950740.1	WP_111591532.1	WP_111592684.1	WP_111595453.1
Bacteroidota	*Chitinophaga eiseniae*	WP_168741694.1	WP_078670110.1	WP_078670884.1	WP_168741852.1

Bacteroidota	*Chitinophaga filiformis*	WP_247813539.1	WP_089834396.1	WP_089836010.1	WP_089830986.1
Bacteroidota	*Chitinophaga flava*	WP_113617360.1	WP_113616485.1	WP_113614148.1	WP_113618438.1
Bacteroidota	*Chitinophaga fulva*	WP_169228654.1	WP_169222926.1	WP_169225324.1	WP_169227571.1
Bacteroidota	*Chitinophaga ginsengisoli*	WP_245901671.1	WP_106600515.1	WP_106605529.1	WP_106600880.1
Bacteroidota	*Chitinophaga horti*	WP_264281860.1	WP_244842465.1	WP_264282605.1	WP_264282541.1
Bacteroidota	*Chitinophaga hostae*	WP_211971874.1	WP_211973759.1	WP_211974175.1	WP_211973914.1
Bacteroidota	*Chitinophaga japonensis*	WP_145713553.1	WP_145711536.1	WP_145715336.1	WP_145717070.1
Bacteroidota	*Chitinophaga jiangningensis*	WP_073083571.1	WP_073084114.1	WP_073086198.1	WP_073087053.1
Bacteroidota	*Chitinophaga lutea*	WP_123848482.1	WP_123845536.1	WP_123849538.1	WP_158618180.1
Bacteroidota	*Chitinophaga niabensis*	WP_074239863.1	WP_343304213.1	WP_074238956.1	WP_143197336.1
Bacteroidota	*Chitinophaga niastensis*	WP_106528029.1	WP_106526122.1	WP_106528908.1	WP_106530351.1
Bacteroidota	*Chitinophaga nivalis*	WP_264727449.1	WP_264735150.1	WP_264728856.1	WP_264732223.1
Bacteroidota	*Chitinophaga oryzae*	WP_168806582.1	WP_168807646.1	WP_168859804.1	WP_168804602.1
Bacteroidota	*Chitinophaga oryziterrae*	WP_157299517.1	WP_157302517.1	WP_157302627.1	WP_157300745.1
Bacteroidota	*Chitinophaga parva*	WP_108686952.1	WP_108684680.1	WP_108687857.1	WP_108687548.1
Bacteroidota	*Chitinophaga pinensis*	WP_261387389.1	WP_146305122.1	WP_012790728.1	WP_012792326.1
Bacteroidota	*Chitinophaga pollutisoli*	WP_341835061.1	WP_341838311.1	WP_341836676.1	WP_341835840.1
Bacteroidota	*Chitinophaga polysaccharea*	WP_142686179.1	WP_145660751.1	WP_168763632.1	WP_145670857.1
Bacteroidota	*Chitinophaga qingshengii*	WP_188089689.1	WP_188091016.1	WP_188086724.1	WP_188091470.1
Bacteroidota	*Chitinophaga rhizophila*	WP_317197475.1	WP_220251837.1	WP_220253029.1	WP_220252641.1
Bacteroidota	*Chitinophaga rhizosphaerae*	WP_126246916.1	WP_126245588.1	WP_126249185.1	WP_164714245.1
Bacteroidota	*Chitinophaga rupis*	WP_089913986.1	WP_089920443.1	WP_089919493.1	WP_089909602.1
Bacteroidota	*Chitinophaga sancti*	WP_320580850.1	WP_320580275.1	WP_072357214.1	WP_320573126.1
Bacteroidota	*Chitinophaga silvatica*	WP_116977058.1	WP_116975180.1	WP_116973669.1	WP_116976693.1
Bacteroidota	*Chitinophaga silvisoli*	WP_233525504.1	WP_116853605.1	WP_116854710.1	WP_116854545.1
Bacteroidota	*Chitinophaga skermanii*	WP_111599050.1	WP_111595753.1	WP_111597493.1	WP_111596564.1
Bacteroidota	*Chitinophaga solisilvae*	WP_235938309.1	WP_127037710.1	WP_160712735.1	WP_160716070.1
Bacteroidota	*Chitinophaga sp.*	WP_325645883.1	WP_343669784.1	WP_326464793.1	WP_298707121.1

Bacteroidota	*Chitinophaga terrae (ex Kim and Jung 2007)*	WP_089759984.1	WP_089758629.1	WP_089759461.1	WP_089760460.1
Bacteroidota	*Chitinophaga tropicalis*	WP_232539105.1	WP_157304928.1	WP_157309866.1	WP_157306277.1
Bacteroidota	*Chitinophaga varians*	WP_188099639.1	WP_188096056.1	WP_188101673.1	WP_168873033.1
Bacteroidota	*Chitinophaga vietnamensis*	WP_143307862.1	WP_143310755.1	WP_143310472.1	WP_143306111.1
Bacteroidota	*Chryseobacterium angstadtii*	WP_048507973.1	WP_048504691.1	WP_048508403.1	WP_048504839.1
Bacteroidota	*Chryseobacterium aquaeductus*	WP_162088794.1	WP_162087455.1	WP_162088291.1	WP_162087563.1
Bacteroidota	*Chryseobacterium aquaticum*	WP_059136490.1	WP_056017623.1	WP_056013198.1	WP_059135831.1
Bacteroidota	*Chryseobacterium arachidis*	WP_072952915.1	WP_072953422.1	WP_072962042.1	WP_072953694.1
Bacteroidota	*Chryseobacterium arthrosphaerae*	WP_229047544.1	WP_065401126.1	WP_229045977.1	WP_229046828.1
Bacteroidota	*Chryseobacterium artocarpi*	WP_065393734.1	WP_065393438.1	WP_065395308.1	WP_065395850.1
Bacteroidota	*Chryseobacterium aureum*	WP_126654068.1	WP_126653405.1	WP_126652494.1	WP_126653278.1
Bacteroidota	*Chryseobacterium binzhouense*	WP_143884491.1	WP_160812457.1	WP_143885159.1	WP_143885655.1
Bacteroidota	*Chryseobacterium camelliae*	WP_309869801.1	WP_271148067.1	WP_100075739.1	WP_100076441.1
Bacteroidota	*Chryseobacterium carnipullorum*	WP_276729760.1	WP_073335484.1	WP_073329474.1	WP_123877096.1
Bacteroidota	*Chryseobacterium cheonjiense*	WP_169229803.1	WP_169231258.1	WP_169232082.1	WP_169231368.1
Bacteroidota	*Chryseobacterium contaminans*	WP_336959609.1	WP_066699585.1	WP_336963978.1	WP_066696416.1
Bacteroidota	*Chryseobacterium culicis*	WP_193545624.1	WP_193546784.1	WP_105681681.1	WP_105683270.1
Bacteroidota	*Chryseobacterium daecheongense*	WP_123263217.1	WP_245142642.1	WP_123263453.1	WP_245142849.1
Bacteroidota	*Chryseobacterium daeguense*	WP_027378272.1	WP_027380310.1	WP_027381351.1	WP_027380381.1
Bacteroidota	*Chryseobacterium defluvii*	WP_121461429.1	WP_184192087.1	WP_184190659.1	WP_184188145.1
Bacteroidota	*Chryseobacterium echinoideorum*	WP_144282438.1	WP_144283498.1	WP_144281767.1	WP_144281870.1
Bacteroidota	*Chryseobacterium edaphi*	WP_263001273.1	WP_263004635.1	WP_263003815.1	WP_263003315.1
Bacteroidota	*Chryseobacterium endalhagicum*	WP_202093532.1	WP_202092377.1	WP_202088381.1	WP_202092137.1
Bacteroidota	*Chryseobacterium flavum*	WP_115964410.1	WP_115962526.1	WP_115959980.1	WP_333596648.1
Bacteroidota	*Chryseobacterium fluminis*	WP_266174365.1	WP_266173542.1	WP_266172508.1	WP_266173392.1
Bacteroidota	*Chryseobacterium foetidum*	WP_262152037.1	WP_262150941.1	WP_262149356.1	WP_262150698.1
Bacteroidota	*Chryseobacterium formosense*	WP_034678774.1	WP_034678117.1	WP_034679053.1	WP_034677951.1

Bacteroidota	*Chryseobacterium formosus*	WP_267264994.1	WP_267266397.1	WP_267266997.1	WP_267266551.1
Bacteroidota	*Chryseobacterium gambrini*	WP_333781483.1	WP_076395077.1	WP_421745152.1	WP_338613118.1
Bacteroidota	*Chryseobacterium gilvum*	WP_262989096.1	WP_262989998.1	WP_262991445.1	WP_262990122.1
Bacteroidota	*Chryseobacterium ginsengisoli*	WP_345208418.1	WP_345204417.1	WP_345207700.1	WP_345204007.1
Bacteroidota	*Chryseobacterium ginsenosidimutans*	WP_344832014.1	WP_344828526.1	WP_344823491.1	WP_344828136.1
Bacteroidota	*Chryseobacterium gleum*	WP_002980773.1	WP_343687245.1	WP_131436182.1	WP_002982977.1
Bacteroidota	*Chryseobacterium gotjawalense*	WP_282905641.1	WP_282903957.1	WP_282903963.1	WP_282903911.1
Bacteroidota	*Chryseobacterium gregarium*	WP_027386164.1	WP_027387127.1	WP_027387487.1	WP_027387596.1
Bacteroidota	*Chryseobacterium group*	WP_063968116.1	WP_063971113.1	WP_063971246.1	WP_063971373.1
Bacteroidota	*Chryseobacterium gwangjuense*	WP_233111112.1	WP_233112162.1	WP_233112332.1	WP_233112287.1
Bacteroidota	*Chryseobacterium hagamense*	WP_146941259.1	WP_146939999.1	WP_146942032.1	WP_146939862.1
Bacteroidota	*Chryseobacterium herbae*	WP_259836630.1	WP_259839341.1	WP_259838793.1	WP_259839482.1
Bacteroidota	*Chryseobacterium indologenes*	WP_062696905.1	WP_034737273.1	WP_276283141.1	WP_034734713.1
Bacteroidota	*Chryseobacterium jejuense*	WP_089732913.1	WP_209919674.1	WP_209916422.1	WP_089734761.1
Bacteroidota	*Chryseobacterium koreense*	WP_304343934.1	WP_048499434.1	WP_048499442.1	WP_048498815.1
Bacteroidota	*Chryseobacterium kwangjuense*	WP_062652674.1	WP_062650765.1	WP_062650258.1	WP_062651103.1
Bacteroidota	*Chryseobacterium lactis*	WP_047097582.1	WP_047098204.1	WP_103291818.1	WP_185290056.1
Bacteroidota	*Chryseobacterium lacus*	WP_114303517.1	WP_126730282.1	WP_126728548.1	WP_126731582.1
Bacteroidota	*Chryseobacterium lathyri*	WP_307210283.1	WP_306840365.1	WP_306845405.1	WP_111956929.1
Bacteroidota	*Chryseobacterium limigenitum*	WP_072411935.1	WP_072407513.1	WP_072411150.1	WP_072407276.1
Bacteroidota	*Chryseobacterium luquanense*	WP_267280016.1	WP_267280638.1	WP_267282236.1	WP_267280772.1
Bacteroidota	*Chryseobacterium luteum*	WP_034704854.1	WP_034702980.1	WP_034701920.1	WP_034702712.1
Bacteroidota	*Chryseobacterium manosquense*	WP_188321635.1	WP_123248881.1	WP_188322246.1	WP_188320621.1
Bacteroidota	*Chryseobacterium mucoviscidosis*	WP_087708020.1	WP_288460300.1	WP_336717035.1	WP_343663715.1
Bacteroidota	*Chryseobacterium muglaense*	WP_191179782.1	WP_191178277.1	WP_191179160.1	WP_191178394.1
Bacteroidota	*Chryseobacterium nakagawai*	WP_123859852.1	WP_123858886.1	WP_123857931.1	WP_123858751.1
Bacteroidota	*Chryseobacterium nepalense*	WP_248392104.1	WP_248390388.1	WP_326988922.1	WP_326988498.1
Bacteroidota	*Chryseobacterium oleae*	WP_090024241.1	WP_090025259.1	WP_090025667.1	WP_090025098.1

Bacteroidota	*Chryseobacterium oranimense*	WP_343608012.1	WP_040993637.1	WP_040997539.1	WP_343611841.1
Bacteroidota	*Chryseobacterium oryctis*	WP_264743146.1	WP_264743935.1	WP_264742216.1	WP_264744072.1
Bacteroidota	*Chryseobacterium panacisoli*	WP_149388131.1	WP_047382874.1	WP_149387241.1	WP_149387698.1
Bacteroidota	*Chryseobacterium paridis*	WP_200244889.1	WP_200241446.1	WP_200248911.1	WP_200241814.1
Bacteroidota	*Chryseobacterium pennipullorum*	WP_115928125.1	WP_115929148.1	WP_115928623.1	WP_115927319.1
Bacteroidota	*Chryseobacterium phocaeense*	WP_080778555.1	WP_080777849.1	WP_080776683.1	WP_080777716.1
Bacteroidota	*Chryseobacterium phosphatilyticum*	WP_109712741.1	WP_103248064.1	WP_103247118.1	WP_109713458.1
Bacteroidota	*Chryseobacterium piperi*	WP_034685471.1	WP_034687838.1	WP_034681116.1	WP_034680385.1
Bacteroidota	*Chryseobacterium piscicola*	WP_076452458.1	WP_076449729.1	WP_076452144.1	WP_076449478.1
Bacteroidota	*Chryseobacterium polytrichastri*	WP_073293819.1	WP_073295856.1	WP_073293519.1	WP_073296113.1
Bacteroidota	*Chryseobacterium populi*	WP_007842630.1	WP_007846994.1	WP_034669435.1	WP_007847155.1
Bacteroidota	*Chryseobacterium profundimaris*	WP_283422386.1	WP_283421978.1	WP_283422706.1	WP_283421026.1
Bacteroidota	*Chryseobacterium salviniae*	WP_326322517.1	WP_326320873.1	WP_326319633.1	WP_326321868.1
Bacteroidota	*Chryseobacterium scophthalmum*	WP_210150663.1	WP_411811350.1	WP_074232060.1	WP_210148717.1
Bacteroidota	*Chryseobacterium sediminis*	WP_309875175.1	WP_309876686.1	WP_184557784.1	WP_149833964.1
Bacteroidota	*Chryseobacterium shandongense*	WP_123852139.1	WP_123852675.1	WP_123854981.1	WP_123854495.1
Bacteroidota	*Chryseobacterium shigense*	WP_184161100.1	WP_076506939.1	WP_184167674.1	WP_076507142.1
Bacteroidota	*Chryseobacterium soldanellicola*	WP_089753028.1	WP_089754460.1	WP_089756315.1	WP_089754742.1
Bacteroidota	*Chryseobacterium sp.*	WP_312075764.1	WP_147756884.1	WP_353148905.1	WP_312389382.1
Bacteroidota	*Chryseobacterium suipulveris*	WP_243550869.1	WP_243548920.1	WP_243548777.1	WP_243550068.1
Bacteroidota	*Chryseobacterium taeanense*	WP_374460200.1	WP_089856114.1	WP_374460750.1	WP_374458606.1
Bacteroidota	*Chryseobacterium tagetis*	WP_225685454.1	WP_225686926.1	WP_225690509.1	WP_225687090.1
Bacteroidota	*Chryseobacterium taichungense*	WP_312902571.1	WP_312901422.1	WP_089998393.1	WP_312902982.1
Bacteroidota	*Chryseobacterium taihuense*	WP_089742875.1	WP_089744917.1	WP_089744036.1	WP_089741825.1
Bacteroidota	*Chryseobacterium taiwanense*	WP_039366252.1	WP_039364300.1	WP_039365139.1	WP_039364551.1
Bacteroidota	*Chryseobacterium takakiae*	WP_072884494.1	WP_072883998.1	WP_072883461.1	WP_072883882.1
Bacteroidota	*Chryseobacterium taklimakanense*	WP_095070596.1	WP_124806977.1	WP_124807081.1	WP_124785301.1
Bacteroidota	*Chryseobacterium terrae*	WP_408087995.1	WP_408090487.1	WP_408091166.1	WP_408090916.1

Bacteroidota	*Chryseobacterium tructae*	WP_290300233.1	WP_290301454.1	WP_290295476.1	WP_290301759.1
Bacteroidota	*Chryseobacterium turcicum*	WP_230670334.1	WP_230672020.1	WP_230667182.1	WP_230672276.1
Bacteroidota	*Chryseobacterium ureilyticum*	WP_076552841.1	WP_076553735.1	WP_076551667.1	WP_076552180.1
Bacteroidota	*Chryseobacterium vietnamense*	WP_047432483.1	WP_047429091.1	WP_310405478.1	WP_047427422.1
Bacteroidota	*Chryseobacterium vrystaatense*	WP_034742985.1	WP_034738641.1	WP_073175554.1	WP_034738968.1
Bacteroidota	*Chryseobacterium wanjuense*	WP_089792497.1	WP_089793124.1	WP_089795693.1	WP_089793430.1
Bacteroidota	*Chryseobacterium zhengzhouense*	WP_378173883.1	WP_378177090.1	WP_378181010.1	WP_378176574.1
Bacteroidota	*Chryseolinea serpens*	WP_221408807.1	WP_073133656.1	WP_073130757.1	WP_073134550.1
Bacteroidota	*Chryseolinea soli*	WP_119756236.1	WP_119754613.1	WP_119757032.1	WP_119754129.1
Bacteroidota	*Cloacibacterium caeni*	WP_213195642.1	WP_213190510.1	WP_213196799.1	WP_213196929.1
Bacteroidota	*Cloacibacterium normanense*	WP_104792413.1	WP_104794166.1	WP_374451362.1	WP_069800177.1
Bacteroidota	*Cloacibacterium rupense*	WP_188617936.1	WP_188618261.1	WP_188616825.1	WP_229663381.1
Bacteroidota	*Cloacibacterium sp.*	WP_414984650.1	WP_414983089.1	WP_414982360.1	WP_374362169.1
Bacteroidota	*Cnuella takakiae*	WP_073042383.1	WP_073048635.1	WP_073047240.1	WP_073046215.1
Bacteroidota	*Cochleicola gelatinilyticus*	WP_068593090.1	WP_068592651.1	WP_068591820.1	WP_068588824.1
Bacteroidota	*Cognatitamlana onchidii*	WP_136480838.1	WP_136481225.1	WP_136481392.1	WP_136480656.1
Bacteroidota	*Confluentibacter citreus*	WP_100615263.1	WP_100614548.1	WP_100614611.1	WP_100615531.1
Bacteroidota	*Confluentibacter flavum*	WP_106658209.1	WP_106660758.1	WP_106658464.1	WP_106659928.1
Bacteroidota	*Confluentibacter lentus*	WP_100613067.1	WP_100611433.1	WP_100611373.1	WP_100612254.1
Bacteroidota	*Constantimarinum furrinae*	WP_186988948.1	WP_186988759.1	WP_186989389.1	WP_186987917.1
Bacteroidota	*Corallibacter sp.*	WP_418512035.1	WP_418513145.1	WP_418509762.1	WP_418513932.1
Bacteroidota	*Croceibacter atlanticus*	WP_341199413.1	WP_327018545.1	WP_341200636.1	WP_013185972.1
Bacteroidota	*Croceimicrobium hydrocarbonivorans*	WP_210759163.1	WP_210758119.1	WP_210758057.1	WP_210758765.1
Bacteroidota	*Croceimicrobium sp.*	WP_421754238.1	WP_421753003.1	WP_421752888.1	WP_421753676.1
Bacteroidota	*Crocinitomix algicola*	WP_066759458.1	WP_070138319.1	WP_066755789.1	WP_066757945.1
Bacteroidota	*Crocinitomix catalasitica*	WP_051568430.1	WP_027419381.1	WP_027421155.1	WP_051568672.1
Bacteroidota	*Cryomorpha ignava*	WP_163285727.1	WP_163283374.1	WP_163285353.1	WP_163285151.1
Bacteroidota	*Cytophaga aurantiaca*	WP_018341921.1	WP_018341432.1	WP_018344950.1	WP_018344260.1

Bacteroidota	*Cytophaga hutchinsonii*	WP_011583780.1	WP_011583422.1	WP_011585160.1	WP_011584089.1
Bacteroidota	*Daejeonella lutea*	WP_079701599.1	WP_079702447.1	WP_079700741.1	WP_079703645.1
Bacteroidota	*Daejeonella oryzae*	WP_026898753.1	WP_026896934.1	WP_026898669.1	WP_026896820.1
Bacteroidota	*Daejeonella rubra*	WP_090701791.1	WP_090706014.1	WP_090700380.1	WP_090706315.1
Bacteroidota	*Daejeonella sp.*	WP_305363755.1	WP_340382233.1	WP_395625608.1	WP_424288394.1
Bacteroidota	*Danxiaibacter flavus*	WP_369331907.1	WP_369329328.1	WP_369329440.1	WP_369330972.1
Bacteroidota	*Deminuibacter soli*	WP_116847103.1	WP_116845410.1	WP_116847565.1	WP_116846911.1
Bacteroidota	*Desertivirga arenae*	WP_207532248.1	WP_207535454.1	WP_207532598.1	WP_207534650.1
Bacteroidota	*Desertivirga brevis*	WP_207423621.1	WP_207424218.1	WP_207422904.1	WP_207424873.1
Bacteroidota	*Desertivirga xinjiangensis*	WP_256012415.1	WP_256013323.1	WP_256009515.1	WP_256009196.1
Bacteroidota	*Dinghuibacter silviterrae*	WP_133993125.1	WP_133989948.1	WP_133993427.1	WP_133993337.1
Bacteroidota	*Edaphocola aurantiacus*	WP_222165161.1	WP_222166449.1	WP_222167030.1	WP_222166341.1
Bacteroidota	*Edaphocola flava*	WP_129022374.1	WP_129021451.1	WP_129019585.1	WP_161971320.1
Bacteroidota	*Eisenibacter elegans*	WP_027000827.1	WP_027000537.1	WP_027000503.1	WP_027000333.1
Bacteroidota	*Elizabethkingia anophelis*	WP_035588455.1	WP_407473270.1	WP_021348450.1	WP_289217335.1
Bacteroidota	*Elizabethkingia argenteiflava*	WP_166520106.1	WP_166519254.1	WP_166519561.1	WP_166520285.1
Bacteroidota	*Elizabethkingia meningoseptica*	WP_016199328.1	WP_087138152.1	WP_249063179.1	WP_407482187.1
Bacteroidota	*Elizabethkingia sp. JS20170427COW*	WP_138983013.1	WP_138982481.1	WP_138982284.1	WP_138981844.1
Bacteroidota	*Elizabethkingia ursingii*	WP_249081401.1	WP_059325009.1	WP_249079041.1	WP_249078125.1
Bacteroidota	*Empedobacter brevis*	WP_353164418.1	WP_312557397.1	WP_312557845.1	WP_314061045.1
Bacteroidota	*Empedobacter falsenii*	WP_115002071.1	WP_286478688.1	WP_038336617.1	WP_416403977.1
Bacteroidota	*Empedobacter sedimenti*	WP_282628450.1	WP_282628566.1	WP_282630778.1	WP_282629868.1
Bacteroidota	*Empedobacter tilapiae*	WP_135835889.1	WP_135834963.1	WP_135834524.1	WP_135835429.1
Bacteroidota	*Emticicia aquatilis*	WP_188767157.1	WP_188768899.1	WP_188770815.1	WP_188765468.1
Bacteroidota	*Emticicia oligotrophica*	WP_015028859.1	WP_015029864.1	WP_015027285.1	WP_015028296.1
Bacteroidota	*Emticicia sp.*	WP_421768965.1	WP_421769690.1	WP_394993460.1	WP_394994852.1
Bacteroidota	*Epilithonimonas arachidiradicis*	WP_120213231.1	WP_120213936.1	WP_120213882.1	WP_120212215.1
Bacteroidota	*Epilithonimonas bovis*	WP_076784065.1	WP_076781783.1	WP_076783416.1	WP_076783565.1

Bacteroidota	*Epilithonimonas caeni*	WP_027381826.1	WP_027383614.1	WP_027383664.1	WP_034671664.1
Bacteroidota	*Epilithonimonas hispanica*	WP_116036308.1	WP_116033190.1	WP_116033058.1	WP_116033454.1
Bacteroidota	*Epilithonimonas hominis*	WP_312083011.1	WP_313306334.1	WP_312083915.1	WP_312082323.1
Bacteroidota	*Epilithonimonas hungarica*	WP_089874579.1	WP_089874829.1	WP_307311947.1	WP_089870570.1
Bacteroidota	*Epilithonimonas lactis*	WP_051879890.1	WP_034976505.1	WP_034976395.1	WP_311316404.1
Bacteroidota	*Epilithonimonas mollis*	WP_072995787.1	WP_072997678.1	WP_072996781.1	WP_072997439.1
Bacteroidota	*Epilithonimonas pallida*	WP_283415408.1	WP_283416258.1	WP_283416210.1	WP_283415997.1
Bacteroidota	*Epilithonimonas sp.*	WP_312418479.1	WP_312823661.1	WP_333852588.1	WP_312823036.1
Bacteroidota	*Epilithonimonas tenax*	WP_028123098.1	WP_028121144.1	WP_028121200.1	WP_316928783.1
Bacteroidota	*Epilithonimonas vandammei*	WP_124802385.1	WP_312195794.1	WP_124801530.1	WP_317125027.1
Bacteroidota	*Epilithonimonas xixisoli*	WP_133943661.1	WP_133945463.1	WP_133945376.1	WP_133944863.1
Bacteroidota	*Epilithonimonas zeae*	WP_248876890.1	WP_248875836.1	WP_248875783.1	WP_317041454.1
Bacteroidota	*Faecalibacter bovis*	WP_230475886.1	WP_230477862.1	WP_230476304.1	WP_230477768.1
Bacteroidota	*Faecalibacter macacae*	WP_121934179.1	WP_121935498.1	WP_121934504.1	WP_121933187.1
Bacteroidota	*Faecalibacter rhinopitheci*	WP_194182785.1	WP_194183591.1	WP_194181660.1	WP_194183128.1
Bacteroidota	*Faecalibacter sp. LW9*	WP_322970199.1	WP_323674094.1	WP_322969619.1	WP_322971732.1
Bacteroidota	*Ferruginibacter lapsinanis*	WP_229759503.1	WP_229761199.1	WP_229759758.1	WP_229760830.1
Bacteroidota	*Ferruginibacter sp.*	WP_301931299.1	WP_301933928.1	WP_301929043.1	WP_301932499.1
Bacteroidota	*Ferruginibacter yonginensis*	WP_379707491.1	WP_379710127.1	WP_379706315.1	WP_379706877.1
Bacteroidota	*Filimonas effusa*	WP_129002188.1	WP_129005128.1	WP_246022425.1	WP_129001150.1
Bacteroidota	*Filimonas lacunae*	WP_076381105.1	WP_076379672.1	WP_076378918.1	WP_231940298.1
Bacteroidota	*Filimonas zeae*	WP_188951172.1	WP_188957660.1	WP_188951939.1	WP_188952473.1
Bacteroidota	*Flammeovirga kamogawensis*	WP_144073603.1	WP_144073762.1	WP_144073022.1	WP_144074686.1
Bacteroidota	*Flammeovirga pacifica*	WP_044226230.1	WP_044227781.1	WP_044219798.1	WP_044228724.1
Bacteroidota	*Flammeovirga pectinis*	WP_126617619.1	WP_126616529.1	WP_126610898.1	WP_126614491.1
Bacteroidota	*Flammeovirga sp. EKP202*	WP_188170347.1	WP_188169643.1	WP_188168981.1	WP_188168142.1
Bacteroidota	*Flammeovirga yaeyamensis*	WP_169665818.1	WP_169664813.1	WP_169662846.1	WP_169665136.1
Bacteroidota	*Flaviaesturariibacter amylovorans*	WP_345256172.1	WP_345252897.1	WP_345255691.1	WP_345253761.1

Bacteroidota	*Flaviaesturariibacter aridisoli*	WP_131852011.1	WP_131850298.1	WP_131850986.1	WP_131853005.1
Bacteroidota	*Flaviaesturariibacter flavus*	WP_131450506.1	WP_131449966.1	WP_243647739.1	WP_131449650.1
Bacteroidota	*Flavicella marina*	WP_152287659.1	WP_152286514.1	WP_152286678.1	WP_226913082.1
Bacteroidota	*Flavicella sediminum*	WP_139959642.1	WP_139959103.1	WP_139959517.1	WP_139959645.1
Bacteroidota	*Flavicella sp.*	WP_336892167.1	WP_300324116.1	WP_336893444.1	WP_300321354.1
Bacteroidota	*Flavihumibacter cheonanensis*	WP_239596693.1	WP_239602470.1	WP_239595074.1	WP_239595392.1
Bacteroidota	*Flavihumibacter fluminis*	WP_234863950.1	WP_234867994.1	WP_234865708.1	WP_234865854.1
Bacteroidota	*Flavihumibacter fluvii*	WP_214457096.1	WP_214458631.1	WP_214457879.1	WP_214457758.1
Bacteroidota	*Flavihumibacter profundi*	WP_224115923.1	WP_224117810.1	WP_224115123.1	WP_224115252.1
Bacteroidota	*Flavihumibacter rivuli*	WP_214449044.1	WP_214447400.1	WP_214449400.1	WP_239804314.1
Bacteroidota	*Flavihumibacter solisilvae*	WP_039142204.1	WP_039138289.1	WP_039140031.1	WP_039141623.1
Bacteroidota	*Flavihumibacter sp.*	WP_332737307.1	WP_332732135.1	WP_332734476.1	WP_332734753.1
Bacteroidota	*Flavihumibacter stibioxidans*	WP_187257671.1	WP_187255864.1	WP_187255311.1	WP_187255180.1
Bacteroidota	*Flavipsychrobacter stenotrophus*	WP_105040525.1	WP_105041010.1	WP_105040190.1	WP_105037087.1
Bacteroidota	*Flaviramulus aquimarinus*	WP_345272025.1	WP_345272414.1	WP_345274344.1	WP_345272394.1
Bacteroidota	*Flaviramulus basaltis*	WP_072401373.1	WP_072401320.1	WP_171946619.1	WP_072402259.1
Bacteroidota	*Flaviramulus multivorans*	WP_237231591.1	WP_237230794.1	WP_237232560.1	WP_237230925.1
Bacteroidota	*Flaviramulus sp. BrNp1-15*	WP_238676220.1	WP_238673743.1	WP_238676151.1	WP_238673896.1
Bacteroidota	*Flavisolibacter ginsengisoli*	WP_072833833.1	WP_072835562.1	WP_072835367.1	WP_072835393.1
Bacteroidota	*Flavisolibacter ginsenosidimutans*	WP_146783909.1	WP_146785371.1	WP_146781711.1	WP_146781592.1
Bacteroidota	*Flavisolibacter nicotianae*	WP_121355392.1	WP_121354209.1	WP_121356021.1	WP_121356571.1
Bacteroidota	*Flavisolibacter tropicus*	WP_066409453.1	WP_066404609.1	WP_066401941.1	WP_066402027.1
Bacteroidota	*Flavivirga abyssicola*	WP_303315535.1	WP_303317827.1	WP_303315654.1	WP_303319200.1
Bacteroidota	*Flavivirga algicola*	WP_169669217.1	WP_169673212.1	WP_169669341.1	WP_169676338.1
Bacteroidota	*Flavivirga amylovorans*	WP_303283397.1	WP_303282627.1	WP_303283341.1	WP_303283293.1
Bacteroidota	*Flavivirga aquatica*	WP_069831156.1	WP_069830633.1	WP_069831205.1	WP_069830554.1
Bacteroidota	*Flavivirga aquimarina*	WP_303279537.1	WP_303279803.1	WP_303277252.1	WP_303276492.1
Bacteroidota	*Flavivirga eckloniae*	WP_102757772.1	WP_102758028.1	WP_102754615.1	WP_102754426.1

Bacteroidota	*Flavivirga jejuensis*	WP_303301060.1	WP_303303193.1	WP_303301116.1	WP_303302142.1
Bacteroidota	*Flavivirga rizhaonensis*	WP_135879042.1	WP_135878189.1	WP_135874426.1	WP_135874598.1
Bacteroidota	*Flavivirga sp. 57AJ16*	WP_274185376.1	WP_274184694.1	WP_274186157.1	WP_274185721.1
Bacteroidota	*Flavivirga spongiicola*	WP_303308613.1	WP_303307549.1	WP_303308519.1	WP_303308337.1
Bacteroidota	*Flavobacterium acetivorans*	WP_230062601.1	WP_230062547.1	WP_230061645.1	WP_230061457.1
Bacteroidota	*Flavobacterium aciduliphilum*	WP_112111896.1	WP_112111771.1	WP_112113984.1	WP_112111999.1
Bacteroidota	*Flavobacterium adhaerens*	WP_348799578.1	WP_348800068.1	WP_348799652.1	WP_348800379.1
Bacteroidota	*Flavobacterium aerium*	WP_306352775.1	WP_306350197.1	WP_306352814.1	WP_306352934.1
Bacteroidota	*Flavobacterium aestivum*	WP_268846035.1	WP_268846490.1	WP_268846139.1	WP_281322463.1
Bacteroidota	*Flavobacterium agri*	WP_176004311.1	WP_176004808.1	WP_176004297.1	WP_176006078.1
Bacteroidota	*Flavobacterium agrisoli*	WP_200104785.1	WP_200104139.1	WP_200104943.1	WP_200105035.1
Bacteroidota	*Flavobacterium ajazii*	WP_163411136.1	WP_163410375.1	WP_163408156.1	WP_163408377.1
Bacteroidota	*Flavobacterium akiainvivens*	WP_054406163.1	WP_054406461.1	WP_054409670.1	WP_054409766.1
Bacteroidota	*Flavobacterium album*	WP_108778736.1	WP_108778230.1	WP_108778512.1	WP_108779563.1
Bacteroidota	*Flavobacterium algicola*	WP_239861776.1	WP_239865649.1	WP_239867780.1	WP_239866461.1
Bacteroidota	*Flavobacterium alkalisoli*	WP_417352198.1	WP_147583880.1	WP_147583557.1	WP_147584873.1
Bacteroidota	*Flavobacterium alvei*	WP_103804954.1	WP_304198350.1	WP_103806427.1	WP_103806165.1
Bacteroidota	*Flavobacterium ammonificans*	WP_229323660.1	WP_229329560.1	WP_229329027.1	WP_229329847.1
Bacteroidota	*Flavobacterium ammoniigenes*	WP_229316983.1	WP_229316756.1	WP_229316411.1	WP_229316318.1
Bacteroidota	*Flavobacterium amnicola*	WP_129436564.1	WP_129435686.1	WP_129436520.1	WP_129436066.1
Bacteroidota	*Flavobacterium amniphilum*	WP_250595224.1	WP_250595108.1	WP_250597894.1	WP_250598363.1
Bacteroidota	*Flavobacterium anhuiense*	WP_129748659.1	WP_091133020.1	WP_129745604.1	WP_091133591.1
Bacteroidota	*Flavobacterium antarcticum*	WP_022828777.1	WP_022827233.1	WP_022828706.1	WP_022828531.1
Bacteroidota	*Flavobacterium aquariorum*	WP_111409190.1	WP_111410466.1	WP_111409112.1	WP_111409004.1
Bacteroidota	*Flavobacterium aquaticum*	WP_111565510.1	WP_111567236.1	WP_111565803.1	WP_111565483.1
Bacteroidota	*Flavobacterium aquatile*	WP_035126130.1	WP_035127441.1	WP_035126550.1	WP_035126315.1
Bacteroidota	*Flavobacterium aquicola*	WP_115809568.1	WP_115812379.1	WP_115809349.1	WP_115810936.1
Bacteroidota	*Flavobacterium aquidurense*	WP_310296061.1	WP_310299635.1	WP_310297587.1	WP_395432764.1

Bacteroidota	*Flavobacterium aquiphilum*	WP_281226196.1	WP_281228934.1	WP_281226080.1	WP_281228415.1
Bacteroidota	*Flavobacterium araucananum*	WP_089479424.1	WP_089480192.1	WP_089481795.1	WP_089478665.1
Bacteroidota	*Flavobacterium arcticum*	WP_114678501.1	WP_114678538.1	WP_114678788.1	WP_114676491.1
Bacteroidota	*Flavobacterium ardleyense*	WP_379803746.1	WP_379807201.1	WP_318640057.1	WP_318639709.1
Bacteroidota	*Flavobacterium arsenatis*	WP_310026947.1	WP_310025109.1	WP_310028268.1	WP_310023609.1
Bacteroidota	*Flavobacterium artemisiae*	WP_379813796.1	WP_379816507.1	WP_379817049.1	WP_379816923.1
Bacteroidota	*Flavobacterium arundinis*	WP_341696644.1	WP_341695324.1	WP_341696461.1	WP_341696278.1
Bacteroidota	*Flavobacterium aurantiibacter*	WP_094484980.1	WP_094485081.1	WP_094486620.1	WP_094487510.1
Bacteroidota	*Flavobacterium aureirubrum*	WP_342695876.1	WP_342695397.1	WP_342696707.1	WP_342696298.1
Bacteroidota	*Flavobacterium azizsancarii*	WP_271335569.1	WP_271336029.1	WP_271338417.1	WP_271334397.1
Bacteroidota	*Flavobacterium azooxidireducens*	WP_248433839.1	WP_248434158.1	WP_248433393.1	WP_248433145.1
Bacteroidota	*Flavobacterium beibuense*	WP_129749981.1	WP_129751414.1	WP_035132715.1	WP_129751784.1
Bacteroidota	*Flavobacterium bernardetii*	WP_166130803.1	WP_166125315.1	WP_166126073.1	WP_166126115.1
Bacteroidota	*Flavobacterium bizetiae*	WP_173972779.1	WP_263759732.1	WP_173971207.1	WP_173971103.1
Bacteroidota	*Flavobacterium branchiarum*	WP_290265837.1	WP_290262884.1	WP_290262510.1	WP_290266136.1
Bacteroidota	*Flavobacterium branchiicola*	WP_213255567.1	WP_213258301.1	WP_213258164.1	WP_213257900.1
Bacteroidota	*Flavobacterium branchiophilum*	WP_097553948.1	WP_089081239.1	WP_014083730.1	WP_089081437.1
Bacteroidota	*Flavobacterium buctense*	WP_187660344.1	WP_187660824.1	WP_187660755.1	WP_187660569.1
Bacteroidota	*Flavobacterium caeni*	WP_091144087.1	WP_091141017.1	WP_091140782.1	WP_091139974.1
Bacteroidota	*Flavobacterium calami*	WP_341694229.1	WP_341690908.1	WP_341692856.1	WP_341692452.1
Bacteroidota	*Flavobacterium capsici*	WP_313321799.1	WP_313321563.1	WP_313323035.1	WP_313325783.1
Bacteroidota	*Flavobacterium caseinilyticum*	WP_131909939.1	WP_131908690.1	WP_131910707.1	WP_131909271.1
Bacteroidota	*Flavobacterium cauense*	WP_023571930.1	WP_023571266.1	WP_023571697.1	WP_023570087.1
Bacteroidota	*Flavobacterium celericrescens*	WP_166236013.1	WP_166235729.1	WP_166236799.1	WP_166235918.1
Bacteroidota	*Flavobacterium cellulosilyticum*	WP_132009313.1	WP_132006492.1	WP_132006986.1	WP_132004434.1
Bacteroidota	*Flavobacterium cerinum*	WP_256551113.1	WP_128388995.1	WP_256549717.1	WP_256552875.1
Bacteroidota	*Flavobacterium channae*	WP_231836571.1	WP_231834529.1	WP_231836887.1	WP_231836528.1
Bacteroidota	*Flavobacterium cheongpyeongense*	WP_110306664.1	WP_110307633.1	WP_110304959.1	WP_110304752.1

Bacteroidota	*Flavobacterium chilense*	WP_068843172.1	WP_068842693.1	WP_068842835.1	WP_068844503.1
Bacteroidota	*Flavobacterium chuncheonense*	WP_379811074.1	WP_379810082.1	WP_379811473.1	WP_379811128.1
Bacteroidota	*Flavobacterium chungnamense*	WP_345089596.1	WP_345094576.1	WP_345095656.1	WP_345093744.1
Bacteroidota	*Flavobacterium circumlabens*	WP_132037869.1	WP_132035216.1	WP_348079458.1	WP_132037989.1
Bacteroidota	*Flavobacterium collinsii*	WP_173966751.1	WP_281924965.1	WP_263361526.1	WP_395340080.1
Bacteroidota	*Flavobacterium columnare*	WP_192188487.1	WP_014165519.1	WP_138424961.1	WP_163444573.1
Bacteroidota	*Flavobacterium coralii*	WP_348676667.1	WP_223052048.1	WP_223053230.1	WP_348678002.1
Bacteroidota	*Flavobacterium crassostreae*	WP_066334673.1	WP_066335673.1	WP_066334792.1	WP_066334607.1
Bacteroidota	*Flavobacterium croceum*	WP_245874595.1	WP_282787780.1	WP_103726256.1	WP_103726419.1
Bacteroidota	*Flavobacterium crocinum*	WP_109192690.1	WP_109194175.1	WP_109194355.1	WP_109194604.1
Bacteroidota	*Flavobacterium cucumis*	WP_073583744.1	WP_073584520.1	WP_073582549.1	WP_073580360.1
Bacteroidota	*Flavobacterium cupreum*	WP_127337142.1	WP_127338736.1	WP_127336437.1	WP_127339709.1
Bacteroidota	*Flavobacterium cutihirudinis*	WP_115886603.1	WP_115888499.1	WP_115888668.1	WP_115888903.1
Bacteroidota	*Flavobacterium cyanobacteriorum*	WP_094412888.1	WP_094416373.1	WP_094412463.1	WP_094417039.1
Bacteroidota	*Flavobacterium cyclinae*	WP_231841939.1	WP_231843804.1	WP_231841638.1	WP_231841964.1
Bacteroidota	*Flavobacterium daejeonense*	WP_026715165.1	WP_026714153.1	WP_026715500.1	WP_026714113.1
Bacteroidota	*Flavobacterium daemonense*	WP_144337204.1	WP_144339228.1	WP_144337841.1	WP_144338222.1
Bacteroidota	*Flavobacterium dankookense*	WP_133533032.1	WP_133531808.1	WP_133531489.1	WP_133533978.1
Bacteroidota	*Flavobacterium dauae*	WP_129758356.1	WP_129758015.1	WP_129757852.1	WP_129757042.1
Bacteroidota	*Flavobacterium defluvii*	WP_073413815.1	WP_073416481.1	WP_073416641.1	WP_073417082.1
Bacteroidota	*Flavobacterium degerlachei*	WP_091429533.1	WP_091434817.1	WP_091429243.1	WP_091434165.1
Bacteroidota	*Flavobacterium denitrificans*	WP_026729249.1	WP_026730144.1	WP_026730335.1	WP_026728034.1
Bacteroidota	*Flavobacterium difficile*	WP_166078192.1	WP_166077145.1	WP_166078006.1	WP_166077778.1
Bacteroidota	*Flavobacterium eburneipallidum*	WP_269225030.1	WP_281336077.1	WP_269225176.1	WP_281337225.1
Bacteroidota	*Flavobacterium enshiense*	WP_023574756.1	WP_023572418.1	WP_367230350.1	WP_023574299.1
Bacteroidota	*Flavobacterium erciyesense*	WP_210789057.1	WP_210788498.1	WP_210788461.1	WP_210788629.1
Bacteroidota	*Flavobacterium facile*	WP_329804406.1	WP_329804786.1	WP_329806052.1	WP_329806076.1
Bacteroidota	*Flavobacterium faecale*	WP_108741003.1	WP_108741200.1	WP_108740786.1	WP_418263472.1

Bacteroidota	*Flavobacterium filum*	WP_051220712.1	WP_276979020.1	WP_026709932.1	WP_026711516.1
Bacteroidota	*Flavobacterium flavigenum*	WP_269235311.1	WP_269236635.1	WP_269236927.1	WP_269237110.1
Bacteroidota	*Flavobacterium flavipallidum*	WP_341700562.1	WP_341700249.1	WP_341700978.1	WP_341701690.1
Bacteroidota	*Flavobacterium flevense*	WP_073245238.1	WP_073245585.1	WP_073245546.1	WP_073244749.1
Bacteroidota	*Flavobacterium fluviale*	WP_113678179.1	WP_113679386.1	WP_113680010.1	WP_113679743.1
Bacteroidota	*Flavobacterium fluviatile*	WP_163399140.1	WP_163399805.1	WP_163401290.1	WP_163400519.1
Bacteroidota	*Flavobacterium fluvii*	WP_073372003.1	WP_073370950.1	WP_073371333.1	WP_073370273.1
Bacteroidota	*Flavobacterium foetidum*	WP_135220181.1	WP_135223692.1	WP_135220578.1	WP_135220849.1
Bacteroidota	*Flavobacterium fontis*	WP_073363541.1	WP_073362329.1	WP_073365076.1	WP_073362776.1
Bacteroidota	*Flavobacterium fragile*	WP_250582634.1	WP_250581805.1	WP_250582693.1	WP_250582588.1
Bacteroidota	*Flavobacterium franklandianum*	WP_143389910.1	WP_143391881.1	WP_143391434.1	WP_143391604.1
Bacteroidota	*Flavobacterium frigidarium*	WP_339654646.1	WP_026707310.1	WP_371570404.1	WP_371571863.1
Bacteroidota	*Flavobacterium frigidimaris*	WP_074657062.1	WP_074658449.1	WP_074658704.1	WP_074659117.1
Bacteroidota	*Flavobacterium frigoris*	WP_007138811.1	WP_007139028.1	WP_074723038.1	WP_074723885.1
Bacteroidota	*Flavobacterium frigoritolerans*	WP_264286204.1	WP_264287449.1	WP_264287159.1	WP_264285323.1
Bacteroidota	*Flavobacterium fructosi*	WP_379858511.1	WP_379857536.1	WP_379858922.1	WP_379857307.1
Bacteroidota	*Flavobacterium galactosidilyticum*	WP_229942134.1	WP_229942020.1	WP_229941185.1	WP_229943974.1
Bacteroidota	*Flavobacterium gelatinilyticum*	WP_281234421.1	WP_281232719.1	WP_281232539.1	WP_281232268.1
Bacteroidota	*Flavobacterium gelidilacus*	WP_051191491.1	WP_026715688.1	WP_026716442.1	WP_026716198.1
Bacteroidota	*Flavobacterium gillisiae*	WP_091086804.1	WP_091086427.1	WP_091093031.1	WP_091090389.1
Bacteroidota	*Flavobacterium gilvum*	WP_035634759.1	WP_035637836.1	WP_035638905.1	WP_035640009.1
Bacteroidota	*Flavobacterium ginsengiterrae*	WP_345144824.1	WP_345146172.1	WP_345145973.1	WP_345145719.1
Bacteroidota	*Flavobacterium ginsenosidimutans*	WP_111286560.1	WP_111285252.1	WP_111285576.1	WP_111289076.1
Bacteroidota	*Flavobacterium glaciei*	WP_114754681.1	WP_114755205.1	WP_114755304.1	WP_114753358.1
Bacteroidota	*Flavobacterium glycines*	WP_066330025.1	WP_066329059.1	WP_066329643.1	WP_066324645.1
Bacteroidota	*Flavobacterium granuli*	WP_310003045.1	WP_072937916.1	WP_072941919.1	WP_310008176.1
Bacteroidota	*Flavobacterium gyeonganense*	WP_278009297.1	WP_278010294.1	WP_278010560.1	WP_278010745.1
Bacteroidota	*Flavobacterium haoranii*	WP_072784910.1	WP_072783792.1	WP_072785678.1	WP_072785004.1

Bacteroidota	*Flavobacterium hercynium*	WP_089047846.1	WP_089051503.1	WP_089050641.1	WP_089051567.1
Bacteroidota	*Flavobacterium hibernum*	WP_041518017.1	WP_041518361.1	WP_041515948.1	WP_041517094.1
Bacteroidota	*Flavobacterium hibisci*	WP_223681520.1	WP_223680455.1	WP_223683385.1	WP_223681683.1
Bacteroidota	*Flavobacterium humi*	WP_135524624.1	WP_135524654.1	WP_135526473.1	WP_135526235.1
Bacteroidota	*Flavobacterium humidisoli*	WP_248726213.1	WP_248727515.1	WP_248727640.1	WP_248727894.1
Bacteroidota	*Flavobacterium hungaricum*	WP_193844941.1	WP_194139755.1	WP_194139922.1	WP_194140192.1
Bacteroidota	*Flavobacterium hydatis*	WP_035627509.1	WP_035620475.1	WP_035627907.1	WP_035625949.1
Bacteroidota	*Flavobacterium hydrocarbonoxydans*	WP_160374738.1	WP_160373147.1	WP_160376079.1	WP_160375866.1
Bacteroidota	*Flavobacterium hydrophilum*	WP_110348265.1	WP_110345293.1	WP_110344961.1	WP_110344757.1
Bacteroidota	*Flavobacterium ichthyis*	WP_166537492.1	WP_166536024.1	WP_166535779.1	WP_166537713.1
Bacteroidota	*Flavobacterium jejuense*	WP_140960659.1	WP_140963139.1	WP_140959233.1	WP_140960741.1
Bacteroidota	*Flavobacterium johnsoniae*	WP_149207923.1	WP_071638765.1	WP_289877969.1	WP_354017319.1
Bacteroidota	*Flavobacterium kayseriense*	WP_187010675.1	WP_187011015.1	WP_187011005.1	WP_187010795.1
Bacteroidota	*Flavobacterium kingsejongi*	WP_108738093.1	WP_108737943.1	WP_108737494.1	WP_108738362.1
Bacteroidota	*Flavobacterium lacus*	WP_112085686.1	WP_112085796.1	WP_112085450.1	WP_112086584.1
Bacteroidota	*Flavobacterium lacustre*	WP_269685402.1	WP_269684458.1	WP_269685141.1	WP_269684167.1
Bacteroidota	*Flavobacterium laiguense*	WP_116762616.1	WP_116763267.1	WP_116764434.1	WP_116760445.1
Bacteroidota	*Flavobacterium limi*	WP_163395014.1	WP_163396595.1	WP_163394177.1	WP_163394387.1
Bacteroidota	*Flavobacterium limicola*	WP_121364806.1	WP_121365842.1	WP_121365603.1	WP_121365642.1
Bacteroidota	*Flavobacterium limnophilum*	WP_281299277.1	WP_281299364.1	WP_281299001.1	WP_281297214.1
Bacteroidota	*Flavobacterium limnosediminis*	WP_023580149.1	WP_023579171.1	WP_023579392.1	WP_023580327.1
Bacteroidota	*Flavobacterium litorale*	WP_220640035.1	WP_220639998.1	WP_220640443.1	WP_220640681.1
Bacteroidota	*Flavobacterium longum*	WP_415579833.1	WP_415580331.1	WP_415580030.1	WP_415581162.1
Bacteroidota	*Flavobacterium lotistagni*	WP_166336344.1	WP_166335680.1	WP_166336123.1	WP_166334538.1
Bacteroidota	*Flavobacterium luminosum*	WP_250592192.1	WP_250592150.1	WP_250591819.1	WP_250591576.1
Bacteroidota	*Flavobacterium luteum*	WP_151108361.1	WP_151108338.1	WP_151106222.1	WP_151107915.1
Bacteroidota	*Flavobacterium macacae*	WP_125012638.1	WP_125011741.1	WP_125013480.1	WP_125012357.1
Bacteroidota	*Flavobacterium magnesitis*	WP_373392694.1	WP_373391948.1	WP_373393194.1	WP_373390870.1

Bacteroidota	*Flavobacterium magnum*	WP_108371479.1	WP_108371837.1	WP_108373551.1	WP_108370611.1
Bacteroidota	*Flavobacterium maritimum*	WP_348813958.1	WP_348812361.1	WP_348811647.1	WP_348811765.1
Bacteroidota	*Flavobacterium microcysteis*	WP_140001285.1	WP_140000093.1	WP_140001393.1	WP_140002987.1
Bacteroidota	*Flavobacterium micromati*	WP_073018050.1	WP_073021752.1	WP_073021956.1	WP_073021419.1
Bacteroidota	*Flavobacterium muglaense*	WP_187018573.1	WP_187020500.1	WP_187019474.1	WP_187016867.1
Bacteroidota	*Flavobacterium myungsuense*	WP_379756042.1	WP_379759358.1	WP_379758019.1	WP_379755506.1
Bacteroidota	*Flavobacterium nackdongense*	WP_133275741.1	WP_133277831.1	WP_133275103.1	WP_133275856.1
Bacteroidota	*Flavobacterium nakdongensis*	WP_309531785.1	WP_309533082.1	WP_309532800.1	WP_309531613.1
Bacteroidota	*Flavobacterium nitratireducens*	WP_278035989.1	WP_278035832.1	WP_278035364.1	WP_278035249.1
Bacteroidota	*Flavobacterium noncentrifugens*	WP_091394897.1	WP_091396656.1	WP_091398032.1	WP_091393211.1
Bacteroidota	*Flavobacterium odoriferum*	WP_262319179.1	WP_262318657.1	WP_262318477.1	WP_262317886.1
Bacteroidota	*Flavobacterium okayamense*	WP_221257746.1	WP_221258181.1	WP_221258373.1	WP_221257683.1
Bacteroidota	*Flavobacterium olei*	WP_347051710.1	WP_347049574.1	WP_347049694.1	WP_347049942.1
Bacteroidota	*Flavobacterium oreochromis*	WP_235873409.1	WP_088397939.1	WP_088399325.1	WP_088397908.1
Bacteroidota	*Flavobacterium orientale*	WP_188361824.1	WP_188361792.1	WP_188361733.1	WP_188361861.1
Bacteroidota	*Flavobacterium ovatum*	WP_366187315.1	WP_366183810.1	WP_366183988.1	WP_366184218.1
Bacteroidota	*Flavobacterium pallidum*	WP_108904360.1	WP_108904208.1	WP_108904408.1	WP_108903708.1
Bacteroidota	*Flavobacterium palustre*	WP_188495722.1	WP_188493166.1	WP_188493705.1	WP_188493752.1
Bacteroidota	*Flavobacterium paronense*	WP_290285321.1	WP_290285180.1	WP_290285908.1	WP_290284915.1
Bacteroidota	*Flavobacterium pectinovorum*	WP_302095401.1	WP_302096861.1	WP_140511608.1	WP_302097218.1
Bacteroidota	*Flavobacterium petrolei*	WP_395381288.1	WP_113666649.1	WP_113666264.1	WP_395380779.1
Bacteroidota	*Flavobacterium phragmitis*	WP_091491246.1	WP_091492985.1	WP_091491738.1	WP_091492158.1
Bacteroidota	*Flavobacterium phycosphaerae*	WP_162127828.1	WP_162127750.1	WP_162126086.1	WP_162128342.1
Bacteroidota	*Flavobacterium piscinae*	WP_129464091.1	WP_321538495.1	WP_129464344.1	WP_129464983.1
Bacteroidota	*Flavobacterium pisciphilum*	WP_229990811.1	WP_229987522.1	WP_229987734.1	WP_229990525.1
Bacteroidota	*Flavobacterium piscis*	WP_310282236.1	WP_310284124.1	WP_065450509.1	WP_310281901.1
Bacteroidota	*Flavobacterium piscisymbiosum*	WP_230036937.1	WP_230039643.1	WP_230039970.1	WP_230040703.1
Bacteroidota	*Flavobacterium plantiphilum*	WP_408081925.1	WP_408078841.1	WP_408078801.1	WP_408081465.1

Bacteroidota	*Flavobacterium pokkalii*	WP_188221639.1	WP_188220226.1	WP_055092636.1	WP_188221459.1
Bacteroidota	*Flavobacterium polysaccharolyticum*	WP_342692751.1	WP_342692940.1	WP_342690826.1	WP_342691523.1
Bacteroidota	*Flavobacterium ponti*	WP_379742522.1	WP_379740641.1	WP_379741904.1	WP_379743091.1
Bacteroidota	*Flavobacterium potami*	WP_223705374.1	WP_223704457.1	WP_223704287.1	WP_223704074.1
Bacteroidota	*Flavobacterium poyangense*	WP_166924366.1	WP_166923811.1	WP_166925746.1	WP_166922132.1
Bacteroidota	*Flavobacterium praedii*	WP_281240167.1	WP_281239829.1	WP_281240117.1	WP_281239177.1
Bacteroidota	*Flavobacterium procerum*	WP_379686047.1	WP_379682565.1	WP_379682729.1	WP_379683027.1
Bacteroidota	*Flavobacterium profundi*	WP_140998711.1	WP_140996821.1	WP_140999128.1	WP_140998869.1
Bacteroidota	*Flavobacterium proteolyticum*	WP_194096385.1	WP_194095444.1	WP_194095749.1	WP_194094760.1
Bacteroidota	*Flavobacterium psychraquaticum*	WP_322550477.1	WP_322549922.1	WP_322549451.1	WP_322550351.1
Bacteroidota	*Flavobacterium psychrophilum*	WP_341966078.1	WP_011962921.1	WP_046205375.1	WP_247440291.1
Bacteroidota	*Flavobacterium psychroterrae*	WP_213304194.1	WP_213298471.1	WP_213303007.1	WP_213305312.1
Bacteroidota	*Flavobacterium psychrotolerans*	WP_116724932.1	WP_116725783.1	WP_116723503.1	WP_116723560.1
Bacteroidota	*Flavobacterium psychrotrophum*	WP_116789094.1	WP_116788075.1	WP_116788784.1	WP_116789352.1
Bacteroidota	*Flavobacterium pygoscelis*	WP_248427670.1	WP_248427562.1	WP_248429168.1	WP_248427783.1
Bacteroidota	*Flavobacterium qiangtangense*	WP_379789644.1	WP_379790684.1	WP_379791238.1	WP_379792899.1
Bacteroidota	*Flavobacterium rakeshii*	WP_157481558.1	WP_157483142.1	WP_330122173.1	WP_330122791.1
Bacteroidota	*Flavobacterium reichenbachii*	WP_035683762.1	WP_035683155.1	WP_035682855.1	WP_035690269.1
Bacteroidota	*Flavobacterium restrictum*	WP_144255659.1	WP_144255860.1	WP_144254725.1	WP_144257666.1
Bacteroidota	*Flavobacterium rhizophilum*	WP_408072782.1	WP_408075709.1	WP_408073956.1	WP_408074961.1
Bacteroidota	*Flavobacterium rhizosphaerae*	WP_408083500.1	WP_408083731.1	WP_408083897.1	WP_408083921.1
Bacteroidota	*Flavobacterium rivuli*	WP_020211280.1	WP_020214032.1	WP_020212488.1	WP_020212097.1
Bacteroidota	*Flavobacterium rivulicola*	WP_171223067.1	WP_171222509.1	WP_171221989.1	WP_171223169.1
Bacteroidota	*Flavobacterium saccharophilum*	WP_072971291.1	WP_072972051.1	WP_072972649.1	WP_072973028.1
Bacteroidota	*Flavobacterium saliperosum*	WP_023575827.1	WP_023576809.1	WP_023576527.1	WP_023577062.1
Bacteroidota	*Flavobacterium sandaracinum*	WP_132065033.1	WP_132066739.1	WP_132064968.1	WP_132064488.1
Bacteroidota	*Flavobacterium sangjuense*	WP_136152239.1	WP_136152510.1	WP_136152911.1	WP_136152372.1
Bacteroidota	*Flavobacterium sasangense*	WP_026726258.1	WP_026725803.1	WP_026726094.1	WP_026724613.1

Bacteroidota	*Flavobacterium sedimenticola*	WP_283240149.1	WP_283238790.1	WP_283238321.1	WP_283238626.1
Bacteroidota	*Flavobacterium segetis*	WP_072991066.1	WP_072992850.1	WP_072993824.1	WP_072987071.1
Bacteroidota	*Flavobacterium selenitireducens*	WP_191019087.1	WP_191018474.1	WP_191018872.1	WP_191020079.1
Bacteroidota	*Flavobacterium seoulense*	WP_035658105.1	WP_035660257.1	WP_035661625.1	WP_035662614.1
Bacteroidota	*Flavobacterium sharifuzzamanii*	WP_111424150.1	WP_111426281.1	WP_111426401.1	WP_111426645.1
Bacteroidota	*Flavobacterium shii*	WP_264205074.1	WP_264206984.1	WP_264204354.1	WP_264206383.1
Bacteroidota	*Flavobacterium silvaticum*	WP_169525831.1	WP_169528127.1	WP_169526441.1	WP_169528369.1
Bacteroidota	*Flavobacterium silvisoli*	WP_131476404.1	WP_131476214.1	WP_131476810.1	WP_131475440.1
Bacteroidota	*Flavobacterium sinopsychrotolerans*	WP_091172020.1	WP_091168489.1	WP_091166664.1	WP_091166756.1
Bacteroidota	*Flavobacterium soli*	WP_026705388.1	WP_026705573.1	WP_026705749.1	WP_026704342.1
Bacteroidota	*Flavobacterium solisilvae*	WP_169523010.1	WP_169523750.1	WP_169524792.1	WP_169524984.1
Bacteroidota	*Flavobacterium soyae*	WP_232682820.1	WP_406844002.1	WP_406844131.1	WP_232678917.1
Bacteroidota	*Flavobacterium soyangense*	WP_194312891.1	WP_194311707.1	WP_194311327.1	WP_194310813.1
Bacteroidota	*Flavobacterium sp.*	WP_324218812.1	WP_310560047.1	WP_298143336.1	WP_326401577.1
Bacteroidota	*Flavobacterium stagni*	WP_129461543.1	WP_129461143.1	WP_129462148.1	WP_129460802.1
Bacteroidota	*Flavobacterium suaedae*	WP_188619340.1	WP_188621315.1	WP_188620323.1	WP_188620523.1
Bacteroidota	*Flavobacterium subsaxonicum*	WP_026992171.1	WP_026991950.1	WP_026990105.1	WP_026993033.1
Bacteroidota	*Flavobacterium succinicans*	WP_024980019.1	WP_064714826.1	WP_064715172.1	WP_064715246.1
Bacteroidota	*Flavobacterium sufflavum*	WP_128197814.1	WP_128196693.1	WP_128194842.1	WP_128194048.1
Bacteroidota	*Flavobacterium suncheonense*	WP_026979736.1	WP_026981611.1	WP_026979946.1	WP_026981151.1
Bacteroidota	*Flavobacterium supellecticarium*	WP_136402496.1	WP_136404178.1	WP_136401869.1	WP_136403284.1
Bacteroidota	*Flavobacterium swingsii*	WP_091474450.1	WP_091474524.1	WP_091474723.1	WP_091472898.1
Bacteroidota	*Flavobacterium tagetis*	WP_202001004.1	WP_202003963.1	WP_202003572.1	WP_202006294.1
Bacteroidota	*Flavobacterium taihuense*	WP_219316622.1	WP_219318647.1	WP_219318958.1	WP_219319062.1
Bacteroidota	*Flavobacterium tegetincola*	WP_026977841.1	WP_026978617.1	WP_026977492.1	WP_026978195.1
Bacteroidota	*Flavobacterium terrae*	WP_073310989.1	WP_073310928.1	WP_073311178.1	WP_073309335.1
Bacteroidota	*Flavobacterium terrigena*	WP_091315599.1	WP_091313686.1	WP_091315883.1	WP_091311805.1
Bacteroidota	*Flavobacterium terrisoli*	WP_284652054.1	WP_284652565.1	WP_284653435.1	WP_284652285.1

Bacteroidota	*Flavobacterium tibetense*	WP_113988678.1	WP_113988761.1	WP_113989048.1	WP_113988628.1
Bacteroidota	*Flavobacterium tistrianum*	WP_111366714.1	WP_111365996.1	WP_111366193.1	WP_111364009.1
Bacteroidota	*Flavobacterium turcicum*	WP_166137451.1	WP_166135359.1	WP_166135473.1	WP_166134952.1
Bacteroidota	*Flavobacterium undicola*	WP_161643988.1	WP_161640825.1	WP_161643606.1	WP_161643714.1
Bacteroidota	*Flavobacterium urocaniciphilum*	WP_091470295.1	WP_091465144.1	WP_091468925.1	WP_091468962.1
Bacteroidota	*Flavobacterium urumqiense*	WP_103999328.1	WP_104000151.1	WP_104000107.1	WP_103999846.1
Bacteroidota	*Flavobacterium ustbae*	WP_125719466.1	WP_125722837.1	WP_125720253.1	WP_125723234.1
Bacteroidota	*Flavobacterium xinjiangense*	WP_073207932.1	WP_073211262.1	WP_073208242.1	WP_073207460.1
Bacteroidota	*Flavobacterium xueshanense*	WP_091206530.1	WP_091206099.1	WP_091207614.1	WP_091203754.1
Bacteroidota	*Flavobacterium xylosi*	WP_379853312.1	WP_379854913.1	WP_379853196.1	WP_379854551.1
Bacteroidota	*Flavobacterium yafengii*	WP_282727115.1	WP_282716422.1	WP_282715470.1	WP_282727969.1
Bacteroidota	*Flavobacterium zepuense*	WP_143373431.1	WP_143372536.1	WP_143375269.1	WP_143373232.1
Bacteroidota	*Flavobacterium zhairuonense*	WP_132990185.1	WP_132988062.1	WP_132989944.1	WP_132989696.1
Bacteroidota	*Flavobacterium zhouii*	WP_379851541.1	WP_379852713.1	WP_379851381.1	WP_379849947.1
Bacteroidota	*Flavobacterium zubiriense*	WP_373405786.1	WP_373406039.1	WP_373406299.1	WP_373406703.1
Bacteroidota	*Flectobacillus longus*	WP_283326372.1	WP_283325519.1	WP_283325847.1	WP_283328046.1
Bacteroidota	*Flectobacillus major*	WP_026996775.1	WP_026997808.1	WP_026994392.1	WP_026997855.1
Bacteroidota	*Flectobacillus roseus*	WP_283344929.1	WP_283345382.1	WP_283343837.1	WP_283374206.1
Bacteroidota	*Flectobacillus sp.*	WP_421821822.1	WP_421820662.1	WP_421819229.1	WP_421818880.1
Bacteroidota	*Flexibacter flexilis*	WP_091511107.1	WP_091508130.1	WP_091505765.1	WP_091516603.1
Bacteroidota	*Flexibacter sp. ATCC 35103*	WP_083691640.1	WP_083692816.1	WP_083692959.1	WP_083693241.1
Bacteroidota	*Fluviicola chungangensis*	WP_186279992.1	WP_144331325.1	WP_186280074.1	WP_186280185.1
Bacteroidota	*Fluviicola sp.*	WP_300358642.1	WP_300663211.1	WP_343636787.1	WP_300659961.1
Bacteroidota	*Fluviicola taffensis*	WP_013687018.1	WP_341901437.1	WP_013687603.1	WP_013687422.1
Bacteroidota	*Foetidibacter luteolus*	WP_235920994.1	WP_153796776.1	WP_153800762.1	WP_153795974.1
Bacteroidota	*Formosa agariphila*	WP_038528683.1	WP_038529567.1	WP_038528827.1	WP_038527824.1
Bacteroidota	*Formosa algae*	WP_057779463.1	WP_057779342.1	WP_057778720.1	WP_103191423.1
Bacteroidota	*Formosa maritima*	WP_148454452.1	WP_148452936.1	WP_148454901.1	WP_148455345.1

Bacteroidota	*Formosa sediminum*	WP_143381647.1	WP_143381701.1	WP_143382029.1	WP_143380981.1
Bacteroidota	*Formosa sp. Hel1_33_131*	WP_069677819.1	WP_069677461.1	WP_069675596.1	WP_069675818.1
Bacteroidota	*Formosa undariae*	WP_382381504.1	WP_382381391.1	WP_382381605.1	WP_382382124.1
Bacteroidota	*Frigoriflavimonas asaccharolytica*	WP_173778168.1	WP_173780209.1	WP_173780018.1	WP_173779288.1
Bacteroidota	*Fulvitalea axinellae*	WP_338393436.1	WP_338392299.1	WP_338391720.1	WP_338391676.1
Bacteroidota	*Fulvivirga imtechensis*	WP_009580067.1	WP_009583631.1	WP_009577485.1	WP_009579199.1
Bacteroidota	*Fulvivirga kasyanovii*	WP_155168941.1	WP_155174132.1	WP_155171568.1	WP_343855452.1
Bacteroidota	*Fulvivirga ligni*	WP_233772449.1	WP_233771349.1	WP_233770555.1	WP_233771846.1
Bacteroidota	*Fulvivirga lutea*	WP_205722999.1	WP_205721864.1	WP_205721098.1	WP_205722411.1
Bacteroidota	*Fulvivirga lutimaris*	WP_155186002.1	WP_155187492.1	WP_155185506.1	WP_155188308.1
Bacteroidota	*Fulvivirga marina*	WP_202859358.1	WP_202854470.1	WP_202855140.1	WP_202854505.1
Bacteroidota	*Fulvivirga maritima*	WP_233779464.1	WP_233778822.1	WP_233780993.1	WP_233780020.1
Bacteroidota	*Fulvivirga sedimenti*	WP_225699088.1	WP_225698760.1	WP_225696347.1	WP_225699844.1
Bacteroidota	*Fulvivirga sediminis*	WP_202241851.1	WP_202244114.1	WP_202243775.1	WP_202244855.1
Bacteroidota	*Fulvivirga sp.*	WP_350112549.1	WP_350112158.1	WP_350116307.1	WP_350111703.1
Bacteroidota	*Fulvivirga ulvae*	WP_233766756.1	WP_233767761.1	WP_233762065.1	WP_233765613.1
Bacteroidota	*Gaetbulibacter aquiaggeris*	WP_395438225.1	WP_395438398.1	WP_395438696.1	WP_395437480.1
Bacteroidota	*Gaetbulibacter saemankumensis*	WP_027137536.1	WP_027136548.1	WP_027136618.1	WP_027136785.1
Bacteroidota	*Gaetbulibacter sp. M240*	WP_394875070.1	WP_394875138.1	WP_394875025.1	WP_394875879.1
Bacteroidota	*Gangjinia marincola*	WP_343765107.1	WP_343762526.1	WP_343764592.1	WP_343764901.1
Bacteroidota	*Gelatiniphilus marinus*	WP_388016973.1	WP_388014987.1	WP_388013439.1	WP_388016878.1
Bacteroidota	*Gelidibacter algens*	WP_066430462.1	WP_066431358.1	WP_066437546.1	WP_111625946.1
Bacteroidota	*Gelidibacter gilvus*	WP_129016401.1	WP_129016144.1	WP_129017242.1	WP_129017912.1
Bacteroidota	*Gelidibacter japonicus*	WP_323026877.1	WP_163515830.1	WP_250066750.1	WP_417368856.1
Bacteroidota	*Gelidibacter maritimus*	WP_182204285.1	WP_182206455.1	WP_182204067.1	WP_182205246.1
Bacteroidota	*Gelidibacter mesophilus*	WP_027125198.1	WP_027126355.1	WP_027127133.1	WP_027125360.1
Bacteroidota	*Gelidibacter pelagius*	WP_208232877.1	WP_208233111.1	WP_208233378.1	WP_208232401.1
Bacteroidota	*Gelidibacter salicanalis*	WP_146893854.1	WP_199602477.1	WP_146889517.1	WP_146888335.1

Bacteroidota	*Gelidibacter sediminis*	WP_133758292.1	WP_133758180.1	WP_133757911.1	WP_133757493.1
Bacteroidota	*Gelidibacter sp.*	WP_325578379.1	WP_348037356.1	WP_325448570.1	WP_348035391.1
Bacteroidota	*Geojedonia litorea*	WP_387961785.1	WP_387961949.1	WP_387961441.1	WP_387960537.1
Bacteroidota	*Gilvibacter sediminis*	WP_272831957.1	WP_272832536.1	WP_272832132.1	WP_272833033.1
Bacteroidota	*Gilvibacter sp.*	WP_291187662.1	WP_291185014.1	WP_374960310.1	WP_420379054.1
Bacteroidota	*Gilvirhabdus luticola*	WP_316663263.1	WP_316663118.1	WP_316662346.1	WP_316661412.1
Bacteroidota	*Ginsengibacter hankyongi*	WP_150412771.1	WP_150413724.1	WP_150415216.1	WP_150413131.1
Bacteroidota	*Haloflavibacter putidus*	WP_141421426.1	WP_141420749.1	WP_141421610.1	WP_141422424.1
Bacteroidota	*Halpernia frigidisoli*	WP_090082314.1	WP_090079850.1	WP_090080935.1	WP_177205450.1
Bacteroidota	*Halpernia humi*	WP_103913750.1	WP_103913058.1	WP_103913318.1	WP_103913510.1
Bacteroidota	*Halpernia sp.*	WP_417430268.1	WP_417428735.1	WP_417428411.1	WP_417427724.1
Bacteroidota	*Hanstruepera flava*	WP_250433844.1	WP_250436433.1	WP_250432405.1	WP_250434084.1
Bacteroidota	*Hanstruepera marina*	WP_223033503.1	WP_223034528.1	WP_223033481.1	WP_223033900.1
Bacteroidota	*Hanstruepera neustonica*	WP_103052605.1	WP_103052034.1	WP_103052338.1	WP_103051335.1
Bacteroidota	*Hanstruepera ponticola*	WP_191859824.1	WP_191859655.1	WP_191860971.1	WP_104734393.1
Bacteroidota	*Hufsiella arboris*	WP_160845686.1	WP_160844963.1	WP_160844516.1	WP_160845831.1
Bacteroidota	*Hugenholtzia roseola*	WP_027003369.1	WP_027001197.1	WP_027003762.1	WP_051203851.1
Bacteroidota	*Hwangdonia lutea*	WP_316983296.1	WP_316983175.1	WP_316983375.1	WP_316982178.1
Bacteroidota	*Hwangdonia seohaensis*	WP_311938766.1	WP_311938626.1	WP_311938917.1	WP_311939926.1
Bacteroidota	*Hwangdonia sp.*	WP_418604510.1	WP_418604453.1	WP_418604389.1	WP_418603464.1
Bacteroidota	*Hydrobacter penzbergensis*	WP_092726552.1	WP_092724081.1	WP_092725408.1	WP_092722670.1
Bacteroidota	*Hymenobacter actinosclerus*	WP_092773040.1	WP_092772166.1	WP_092770383.1	WP_092773236.1
Bacteroidota	*Hymenobacter aerophilus*	WP_019949270.1	WP_019947749.1	WP_019946669.1	WP_019946883.1
Bacteroidota	*Hymenobacter algoricola*	WP_345113875.1	WP_345113237.1	WP_345114690.1	WP_345109111.1
Bacteroidota	*Hymenobacter amundsenii*	WP_088462812.1	WP_088464214.1	WP_088464474.1	WP_088462720.1
Bacteroidota	*Hymenobacter aquaticus*	WP_135463777.1	WP_210114293.1	WP_135461986.1	WP_135463988.1
Bacteroidota	*Hymenobacter busanensis*	WP_151078495.1	WP_151079239.1	WP_338074383.1	WP_151078604.1
Bacteroidota	*Hymenobacter canadensis*	WP_269559131.1	WP_269560067.1	WP_269561050.1	WP_269558985.1

Bacteroidota	*Hymenobacter chitinivorans*	WP_100337718.1	WP_100334769.1	WP_100335002.1	WP_100337998.1
Bacteroidota	*Hymenobacter daecheongensis*	WP_073105469.1	WP_073108984.1	WP_073111368.1	WP_073105191.1
Bacteroidota	*Hymenobacter duratus*	WP_211539194.1	WP_190784662.1	WP_190785735.1	WP_190783158.1
Bacteroidota	*Hymenobacter edaphi*	WP_111480507.1	WP_111478724.1	WP_111476768.1	WP_111477643.1
Bacteroidota	*Hymenobacter elongatus*	WP_135498979.1	WP_135499093.1	WP_135499484.1	WP_135498247.1
Bacteroidota	*Hymenobacter endophyticus*	WP_315998093.1	WP_315999127.1	WP_315997629.1	WP_315997288.1
Bacteroidota	*Hymenobacter fastidiosus*	WP_345074911.1	WP_345074853.1	WP_345072700.1	WP_345071857.1
Bacteroidota	*Hymenobacter fodinae*	WP_135430104.1	WP_135433580.1	WP_135436110.1	WP_135430550.1
Bacteroidota	*Hymenobacter gelipurpurascens*	WP_088844785.1	WP_088843741.1	WP_088842140.1	WP_088844610.1
Bacteroidota	*Hymenobacter glacieicola*	WP_188557127.1	WP_188559509.1	WP_188558562.1	WP_188556977.1
Bacteroidota	*Hymenobacter gummosus*	WP_126694282.1	WP_126696445.1	WP_126694959.1	WP_126694911.1
Bacteroidota	*Hymenobacter guriensis*	WP_317191392.1	WP_196953312.1	WP_231403234.1	WP_196956148.1
Bacteroidota	*Hymenobacter jeollabukensis*	WP_138082352.1	WP_138077952.1	WP_138074696.1	WP_138076488.1
Bacteroidota	*Hymenobacter koreensis*	WP_345227100.1	WP_345220804.1	WP_345225765.1	WP_345227550.1
Bacteroidota	*Hymenobacter lapidiphilus*	WP_176907463.1	WP_176907383.1	WP_176906325.1	WP_176910239.1
Bacteroidota	*Hymenobacter lucidus*	WP_226170117.1	WP_226175553.1	WP_226177909.1	WP_226170797.1
Bacteroidota	*Hymenobacter metallicola*	WP_135396638.1	WP_135394722.1	WP_135394102.1	WP_135395606.1
Bacteroidota	*Hymenobacter metallilatus*	WP_125431828.1	WP_125427055.1	WP_125432977.1	WP_125426004.1
Bacteroidota	*Hymenobacter nitidus*	WP_226186498.1	WP_226189119.1	WP_226183341.1	WP_226186023.1
Bacteroidota	*Hymenobacter oligotrophus*	WP_119445448.1	WP_119444743.1	WP_119443979.1	WP_119445303.1
Bacteroidota	*Hymenobacter perfusus*	WP_125438156.1	WP_125439651.1	WP_125440281.1	WP_125435945.1
Bacteroidota	*Hymenobacter persicinus*	WP_129920492.1	WP_129922767.1	WP_129923205.1	WP_129923055.1
Bacteroidota	*Hymenobacter pini*	WP_226270965.1	WP_226265934.1	WP_226269985.1	WP_226271625.1
Bacteroidota	*Hymenobacter piscis*	WP_215593080.1	WP_215594052.1	WP_215592429.1	WP_215591981.1
Bacteroidota	*Hymenobacter psychrophilus*	WP_092740064.1	WP_092739454.1	WP_092741564.1	WP_092739666.1
Bacteroidota	*Hymenobacter psychrotolerans*	WP_073287058.1	WP_073286580.1	WP_073287465.1	WP_073285851.1
Bacteroidota	*Hymenobacter qilianensis*	WP_187733482.1	WP_187732856.1	WP_187732044.1	WP_187733782.1
Bacteroidota	*Hymenobacter rigui*	WP_125424374.1	WP_125420006.1	WP_125423685.1	WP_125417329.1

Bacteroidota	*Hymenobacter roseosalivarius*	WP_084443675.1	WP_084444424.1	WP_084447454.1	WP_084445205.1
Bacteroidota	*Hymenobacter rubripertinctus*	WP_119655126.1	WP_119656221.1	WP_119654072.1	WP_119654547.1
Bacteroidota	*Hymenobacter sediminicola*	WP_260625764.1	WP_185887369.1	WP_185886273.1	WP_185888447.1
Bacteroidota	*Hymenobacter sediminis*	WP_110976193.1	WP_202910460.1	WP_110979241.1	WP_110976343.1
Bacteroidota	*Hymenobacter sp.*	WP_331147061.1	WP_331059006.1	WP_331059402.1	WP_325135416.1
Bacteroidota	*Hymenobacter sublimis*	WP_247975281.1	WP_247975982.1	WP_247976812.1	WP_247975169.1
Bacteroidota	*Hymenobacter swuensis*	WP_044000558.1	WP_044001162.1	WP_044002076.1	WP_044003628.1
Bacteroidota	*Hymenobacter terrestris*	WP_176898131.1	WP_176900196.1	WP_176898993.1	WP_176901120.1
Bacteroidota	*Hymenobacter tibetensis*	WP_243798354.1	WP_243800611.1	WP_243802657.1	WP_243799028.1
Bacteroidota	*Hymenobacter translucens*	WP_227606985.1	WP_227609277.1	WP_227606247.1	WP_227610336.1
Bacteroidota	*Hymenobacter volaticus*	WP_245120172.1	WP_245122496.1	WP_245125362.1	WP_245120586.1
Bacteroidota	*Hymenobacter wooponensis*	WP_135532540.1	WP_135529106.1	WP_135530529.1	WP_135530138.1
Bacteroidota	*Hymenobacter yonginensis*	WP_270126485.1	WP_270127571.1	WP_270128601.1	WP_270126306.1
Bacteroidota	*Hyunsoonleella aestuarii*	WP_139002422.1	WP_139002770.1	WP_139000583.1	WP_139000362.1
Bacteroidota	*Hyunsoonleella aquatilis*	WP_186562002.1	WP_186563531.1	WP_186563450.1	WP_186559073.1
Bacteroidota	*Hyunsoonleella flava*	WP_130962751.1	WP_130962624.1	WP_130962429.1	WP_130963622.1
Bacteroidota	*Hyunsoonleella jejuensis*	WP_092578310.1	WP_092578219.1	WP_092578669.1	WP_092579037.1
Bacteroidota	*Hyunsoonleella pacifica*	WP_130935561.1	WP_130935536.1	WP_130935871.1	WP_130938023.1
Bacteroidota	*Hyunsoonleella rubra*	WP_380293872.1	WP_380288079.1	WP_380289797.1	WP_380292807.1
Bacteroidota	*Hyunsoonleella sp. 2307UL5-6*	WP_413972126.1	WP_413972052.1	WP_413973366.1	WP_413973261.1
Bacteroidota	*Hyunsoonleella ulvae*	WP_203256791.1	WP_203256821.1	WP_203258597.1	WP_203257890.1
Bacteroidota	*Ichthyenterobacterium magnum*	WP_120201057.1	WP_120200732.1	WP_120201318.1	WP_120199279.1
Bacteroidota	*Ichthyobacterium seriolicida*	WP_096686620.1	WP_096686479.1	WP_096686568.1	WP_096685584.1
Bacteroidota	*Imtechella halotolerans*	WP_008239022.1	WP_008240408.1	WP_008238821.1	WP_008238112.1
Bacteroidota	*Jejudonia soesokkakensis*	WP_380217216.1	WP_380216994.1	WP_380217385.1	WP_380218067.1
Bacteroidota	*Jejuia pallidilutea*	WP_042244445.1	WP_105474530.1	WP_105474849.1	WP_105474438.1
Bacteroidota	*Jejuia sp. DST062*	WP_406972028.1	WP_406971608.1	WP_406969494.1	WP_406968753.1
Bacteroidota	*Jejuia spongiicola*	WP_249973538.1	WP_249972989.1	WP_249973577.1	WP_249972470.1

Bacteroidota	*Kaistella antarctica*	WP_034716011.1	WP_034718549.1	WP_034718534.1	WP_051803858.1
Bacteroidota	*Kaistella carnis*	WP_125022700.1	WP_313717127.1	WP_312818541.1	WP_125024677.1
Bacteroidota	*Kaistella chaponensis*	WP_076388336.1	WP_076386389.1	WP_076386374.1	WP_076387281.1
Bacteroidota	*Kaistella daneshvariae*	WP_124758469.1	WP_124757691.1	WP_124757689.1	WP_123266721.1
Bacteroidota	*Kaistella faecalis*	WP_218249738.1	WP_218248450.1	WP_218248442.1	WP_218250327.1
Bacteroidota	*Kaistella flava (ex Peng et al. 2021)*	WP_193812873.1	WP_193811537.1	WP_193811529.1	WP_193811028.1
Bacteroidota	*Kaistella gelatinilytica*	WP_196079166.1	WP_196079956.1	WP_196079963.1	WP_196080199.1
Bacteroidota	*Kaistella haifensis*	WP_031501825.1	WP_031501350.1	WP_031501345.1	WP_031504621.1
Bacteroidota	*Kaistella jeonii*	WP_039352692.1	WP_039351184.1	WP_039351160.1	WP_039350477.1
Bacteroidota	*Kaistella montana*	WP_255927104.1	WP_255929243.1	WP_255929267.1	WP_255930280.1
Bacteroidota	*Kaistella palustris*	WP_027376429.1	WP_027376032.1	WP_027376024.1	WP_027377070.1
Bacteroidota	*Kaistella polysaccharea*	WP_226064635.1	WP_226063347.1	WP_226063355.1	WP_226063808.1
Bacteroidota	*Kaistella soli*	WP_217199978.1	WP_088358885.1	WP_217201330.1	WP_217201368.1
Bacteroidota	*Kaistella solincola*	WP_039345848.1	WP_039342678.1	WP_039341850.1	WP_039341001.1
Bacteroidota	*Kaistella sp.*	WP_332032649.1	WP_373708837.1	WP_423975190.1	WP_373707369.1
Bacteroidota	*Kaistella treverensis*	WP_089819399.1	WP_089819909.1	WP_089819511.1	WP_089819931.1
Bacteroidota	*Kaistella yananensis*	WP_265144198.1	WP_265142890.1	WP_265142884.1	WP_265144070.1
Bacteroidota	*Kaistella yonginensis*	WP_290220396.1	WP_290216955.1	WP_290216940.1	WP_290217812.1
Bacteroidota	*Kordia aestuariivivens*	WP_187561876.1	WP_187563470.1	WP_187561095.1	WP_187564525.1
Bacteroidota	*Kordia algicida*	WP_007093217.1	WP_007093984.1	WP_040559864.1	WP_007094736.1
Bacteroidota	*Kordia antarctica*	WP_160128858.1	WP_160128089.1	WP_160128402.1	WP_160131354.1
Bacteroidota	*Kordia jejudonensis*	WP_046757610.1	WP_046758503.1	WP_046758012.1	WP_046757471.1
Bacteroidota	*Kordia periserrulae*	WP_108113611.1	WP_108114659.1	WP_108114435.1	WP_108116523.1
Bacteroidota	*Kordia sp.*	WP_420574344.1	WP_420573743.1	WP_420572871.1	WP_290671858.1
Bacteroidota	*Kordia zhangzhouensis*	WP_082106780.1	WP_046744617.1	WP_046744409.1	WP_046745889.1
Bacteroidota	*Lacibacter cauensis*	WP_144884070.1	WP_144886451.1	WP_144884137.1	WP_144886127.1
Bacteroidota	*Lacibacter luteus*	WP_129132569.1	WP_129131595.1	WP_129128817.1	WP_129132186.1
Bacteroidota	*Lacibacter sediminis*	WP_182801160.1	WP_182803974.1	WP_182801129.1	WP_182801935.1

Bacteroidota	*Lacibacter sp.*	WP_324232101.1	WP_324231404.1	WP_324232070.1	WP_324230093.1
Bacteroidota	*Lacihabitans lacunae*	WP_379838358.1	WP_379835146.1	WP_379834768.1	WP_379838877.1
Bacteroidota	*Lacihabitans soyangensis*	WP_255038614.1	WP_255038281.1	WP_255039128.1	WP_255037505.1
Bacteroidota	*Lacihabitans sp. CS3-21*	WP_255050028.1	WP_255050370.1	WP_255047189.1	WP_255048821.1
Bacteroidota	*Lacinutrix algicola*	WP_055436825.1	WP_055437101.1	WP_055437076.1	WP_055435440.1
Bacteroidota	*Lacinutrix gracilariae*	WP_379900224.1	WP_379901067.1	WP_379899746.1	WP_379902270.1
Bacteroidota	*Lacinutrix himadriensis*	WP_055443177.1	WP_055444954.1	WP_055443481.1	WP_055445236.1
Bacteroidota	*Lacinutrix iliipiscaria*	WP_183489379.1	WP_183487450.1	WP_183488070.1	WP_183486898.1
Bacteroidota	*Lacinutrix jangbogonensis*	WP_034058149.1	WP_034058557.1	WP_034060840.1	WP_034057457.1
Bacteroidota	*Lacinutrix mariniflava*	WP_055445619.1	WP_055446055.1	WP_055448589.1	WP_055448788.1
Bacteroidota	*Lacinutrix sp.*	WP_290696520.1	WP_340169257.1	WP_300207521.1	WP_290698518.1
Bacteroidota	*Lacinutrix venerupis*	WP_121049029.1	WP_121048868.1	WP_121048213.1	WP_076732624.1
Bacteroidota	*Leptobacterium meishanense*	WP_340074781.1	WP_340077561.1	WP_340073967.1	WP_340074705.1
Bacteroidota	*Limibacter armeniacum*	WP_334246972.1	WP_334246576.1	WP_334246496.1	WP_334246523.1
Bacteroidota	*Limnovirga soli*	WP_171607574.1	WP_171609007.1	WP_171609473.1	WP_171609767.1
Bacteroidota	*Lishizhenia sp.*	WP_318959256.1	WP_318958285.1	WP_318958486.1	WP_318958685.1
Bacteroidota	*Lishizhenia tianjinensis*	WP_090247069.1	WP_090246723.1	WP_090247519.1	WP_090245381.1
Bacteroidota	*Litoribacter populi*	WP_143959586.1	WP_143962451.1	WP_143960986.1	WP_143959597.1
Bacteroidota	*Litoribacter ruber*	WP_213944562.1	WP_213943340.1	WP_213945420.1	WP_213944573.1
Bacteroidota	*Litoribaculum gwangyangense*	WP_345276546.1	WP_345276104.1	WP_345276075.1	WP_345276767.1
Bacteroidota	*Longitalea arenae*	WP_205512728.1	WP_205508771.1	WP_205512896.1	WP_205509770.1
Bacteroidota	*Longitalea luteola*	WP_207513380.1	WP_207511955.1	WP_207513435.1	WP_207513616.1
Bacteroidota	*Luteibaculum oceani*	WP_147014622.1	WP_147014709.1	WP_147014190.1	WP_147013057.1
Bacteroidota	*Luteirhabdus pelagi*	WP_203295889.1	WP_203295780.1	WP_203296683.1	WP_203296609.1
Bacteroidota	*Lutibacter agarilyticus*	WP_089379847.1	WP_089380012.1	WP_089380156.1	WP_089381860.1
Bacteroidota	*Lutibacter citreus*	WP_111708422.1	WP_111708460.1	WP_111707821.1	WP_111708609.1
Bacteroidota	*Lutibacter flavus*	WP_089377158.1	WP_089377124.1	WP_089376832.1	WP_089377379.1
Bacteroidota	*Lutibacter holmesii*	WP_386809318.1	WP_386809209.1	WP_386809111.1	WP_386807184.1

Bacteroidota	*Lutibacter maritimus*	WP_090223066.1	WP_090222967.1	WP_090222428.1	WP_090221845.1
Bacteroidota	*Lutibacter oceani*	WP_115879669.1	WP_115879279.1	WP_115879655.1	WP_115882099.1
Bacteroidota	*Lutibacter oricola*	WP_090121788.1	WP_090121462.1	WP_090120722.1	WP_090123020.1
Bacteroidota	*Lutibacter profundi*	WP_068211365.1	WP_068205635.1	WP_068205690.1	WP_068206420.1
Bacteroidota	*Lutibacter sp.*	WP_291974348.1	WP_368051282.1	WP_305600318.1	WP_372794956.1
Bacteroidota	*Lutimonas halocynthiae*	WP_290292455.1	WP_290293069.1	WP_290293732.1	WP_290289555.1
Bacteroidota	*Lutimonas saemankumensis*	WP_224927108.1	WP_224927446.1	WP_224927476.1	WP_224932014.1
Bacteroidota	*Lutimonas sp.*	WP_424352615.1	WP_424350756.1	WP_424350450.1	WP_424352018.1
Bacteroidota	*Lutimonas vermicola*	WP_342158652.1	WP_342158194.1	WP_342159627.1	WP_342159791.1
Bacteroidota	*Lutimonas zeaxanthinifaciens*	WP_302045662.1	WP_302043014.1	WP_302043042.1	WP_302043417.1
Bacteroidota	*Mangrovimonas aestuarii*	WP_274475618.1	WP_274475567.1	WP_274475634.1	WP_274475942.1
Bacteroidota	*Mangrovimonas cancribranchiae*	WP_338733612.1	WP_338733894.1	WP_338733664.1	WP_338733268.1
Bacteroidota	*Mangrovimonas futianensis*	WP_232395652.1	WP_232634821.1	WP_232395550.1	WP_232634478.1
Bacteroidota	*Mangrovimonas sp. YM274*	WP_307899412.1	WP_307899351.1	WP_307902005.1	WP_307901681.1
Bacteroidota	*Mangrovimonas spongiae*	WP_125467229.1	WP_125468490.1	WP_125467189.1	WP_125467469.1
Bacteroidota	*Mangrovimonas xylaniphaga*	WP_053977740.1	WP_053977790.1	WP_053977261.1	WP_053977459.1
Bacteroidota	*Mangrovimonas yunxiaonensis*	WP_036122858.1	WP_036123792.1	WP_036123026.1	WP_036122376.1
Bacteroidota	*Mangrovivirga halotolerans*	WP_266056956.1	WP_266058647.1	WP_266056794.1	WP_266057182.1
Bacteroidota	*Mariniflexile aquimaris*	WP_379942699.1	WP_379942415.1	WP_379942788.1	WP_379941225.1
Bacteroidota	*Mariniflexile fucanivorans*	WP_132216225.1	WP_132219156.1	WP_132215582.1	WP_132216338.1
Bacteroidota	*Mariniflexile gromovii*	WP_209654374.1	WP_209656289.1	WP_209657102.1	WP_209654042.1
Bacteroidota	*Mariniflexile jejuense*	WP_379926659.1	WP_379924078.1	WP_379926576.1	WP_379926525.1
Bacteroidota	*Mariniflexile litorale*	WP_308992018.1	WP_308992851.1	WP_308992244.1	WP_308992065.1
Bacteroidota	*Mariniflexile maritimum*	WP_157209100.1	WP_157206636.1	WP_157208718.1	WP_157206179.1
Bacteroidota	*Mariniflexile ostreae*	WP_379861387.1	WP_379860781.1	WP_379861361.1	WP_379860627.1
Bacteroidota	*Mariniflexile soesokkakense*	WP_346242364.1	WP_346242016.1	WP_346239891.1	WP_346242332.1
Bacteroidota	*Mariniflexile sp.*	WP_372754610.1	WP_372755176.1	WP_372754476.1	WP_372754686.1
Bacteroidota	*Marinigracilibium pacificum*	WP_169683144.1	WP_169684667.1	WP_169684819.1	WP_169677532.1

Bacteroidota	*Marinirhabdus gelatinilytica*	WP_115124603.1	WP_115124645.1	WP_115123303.1	WP_115123992.1
Bacteroidota	*Marinoscillum furvescens*	WP_115866421.1	WP_115866794.1	WP_245986162.1	WP_115868181.1
Bacteroidota	*Marinoscillum sp. MHG1-6*	WP_258102520.1	WP_258103226.1	WP_258104476.1	WP_258102800.1
Bacteroidota	*Marivirga arenosa*	WP_308355675.1	WP_308356037.1	WP_302125633.1	WP_322347713.1
Bacteroidota	*Marivirga atlantica*	WP_201918422.1	WP_201918138.1	WP_201924100.1	WP_201916581.1
Bacteroidota	*Marivirga aurantiaca*	WP_201430705.1	WP_201432316.1	WP_201429891.1	WP_201432555.1
Bacteroidota	*Marivirga harenae*	WP_303270671.1	WP_303267928.1	WP_303272113.1	WP_303270879.1
Bacteroidota	*Marivirga lumbricoides*	WP_188464610.1	WP_188460934.1	WP_188462202.1	WP_188465104.1
Bacteroidota	*Marivirga salinae*	WP_308349634.1	WP_308351288.1	WP_308347826.1	WP_308349786.1
Bacteroidota	*Marivirga sericea*	WP_085519183.1	WP_085517122.1	WP_085515726.1	WP_085518009.1
Bacteroidota	*Marivirga sp.*	WP_296620148.1	WP_296618831.1	WP_296622034.1	WP_325121820.1
Bacteroidota	*Marivirga tractuosa*	WP_375579149.1	WP_013455427.1	WP_013452380.1	WP_375579341.1
Bacteroidota	*Marixanthomonas ophiurae*	WP_117160414.1	WP_117160044.1	WP_117160475.1	WP_117159718.1
Bacteroidota	*Marixanthomonas sp. SCSIO 43207*	WP_223108255.1	WP_223107920.1	WP_223107512.1	WP_223109995.1
Bacteroidota	*Marixanthomonas spongiae*	WP_116695087.1	WP_116693667.1	WP_116693396.1	WP_116694569.1
Bacteroidota	*Marnyiella aurantia*	WP_209815840.1	WP_181886854.1	WP_181887822.1	WP_181886945.1
Bacteroidota	*Mesoflavibacter sp.*	WP_370225504.1	WP_370225638.1	WP_370228053.1	WP_370225799.1
Bacteroidota	*Mesoflavibacter zeaxanthinifaciens*	WP_304142908.1	WP_304144207.1	WP_106678698.1	WP_106679414.1
Bacteroidota	*Mesohalobacter halotolerans*	WP_138931837.1	WP_138931676.1	WP_138932211.1	WP_138932670.1
Bacteroidota	*Mesonia aestuariivivens*	WP_219039632.1	WP_219039071.1	WP_219040755.1	WP_219041146.1
Bacteroidota	*Mesonia algae*	WP_111541660.1	WP_111540188.1	WP_111541586.1	WP_111541566.1
Bacteroidota	*Mesonia aquimarina*	WP_121665614.1	WP_121665309.1	WP_121665496.1	WP_121666282.1
Bacteroidota	*Mesonia hippocampi*	WP_183477565.1	WP_183477614.1	WP_183475703.1	WP_183477477.1
Bacteroidota	*Mesonia maritima*	WP_309727274.1	WP_309729227.1	WP_309728181.1	WP_309728893.1
Bacteroidota	*Mesonia mobilis*	WP_304133107.1	WP_027883935.1	WP_304157464.1	WP_304156199.1
Bacteroidota	*Mesonia ostreae*	WP_311400708.1	WP_311401302.1	WP_311400781.1	WP_311400862.1
Bacteroidota	*Mesonia profundi*	WP_308864155.1	WP_308863989.1	WP_308864680.1	WP_308863498.1
Bacteroidota	*Mesonia sediminis*	WP_379044607.1	WP_379044484.1	WP_379046647.1	WP_379045708.1

Bacteroidota	*Mesonia sp.*	WP_347420137.1	WP_347419748.1	WP_292246739.1	WP_347418592.1
Bacteroidota	*Microcosmobacter mediterraneus*	WP_311426187.1	WP_311427926.1	WP_311426391.1	WP_311425829.1
Bacteroidota	*Microscilla marina*	WP_002705669.1	WP_002698220.1	WP_002693779.1	WP_002700262.1
Bacteroidota	*Mongoliitalea daihaiensis*	WP_236139018.1	WP_236139112.1	WP_236136828.1	WP_236139309.1
Bacteroidota	*Mongoliitalea lutea*	WP_189579359.1	WP_189579276.1	WP_189585048.1	WP_189579197.1
Bacteroidota	*Mucilaginibacter gracilis*	WP_121200601.1	WP_121201357.1	WP_121198186.1	WP_121197576.1
Bacteroidota	*Mucilaginibacter paludis*	WP_008510050.1	WP_040627802.1	WP_008511304.1	WP_008505046.1
Bacteroidota	*Mucilaginibacter sp.*	WP_348053712.1	WP_348084747.1	WP_369592252.1	WP_348082828.1
Bacteroidota	*Myroides fluvii*	WP_158963029.1	WP_158960886.1	WP_158963905.1	WP_158962616.1
Bacteroidota	*Myroides guanonis*	WP_090677389.1	WP_090677506.1	WP_090677655.1	WP_090680943.1
Bacteroidota	*Myroides injenensis*	WP_010256440.1	WP_010254937.1	WP_010256156.1	WP_010255812.1
Bacteroidota	*Myroides marinus*	WP_038986398.1	WP_038988375.1	WP_286425173.1	WP_038985991.1
Bacteroidota	*Myroides odoratimimus*	WP_286415851.1	WP_025125853.1	WP_286386351.1	WP_286436824.1
Bacteroidota	*Myroides odoratus*	WP_060873289.1	WP_353101116.1	WP_353101147.1	WP_353118317.1
Bacteroidota	*Myroides oncorhynchi*	WP_229946658.1	WP_229948831.1	WP_229948027.1	WP_229946215.1
Bacteroidota	*Myroides pelagicus*	WP_155036017.1	WP_155035200.1	WP_155036246.1	WP_155035270.1
Bacteroidota	*Myroides phaeus*	WP_090406831.1	WP_090406041.1	WP_090408418.1	WP_090409129.1
Bacteroidota	*Myroides sp. DW712*	WP_410877564.1	WP_410879920.1	WP_410877788.1	WP_410881116.1
Bacteroidota	*Nafulsella turpanensis*	WP_017730111.1	WP_017731237.1	WP_017729874.1	WP_017731273.1
Bacteroidota	*Namhaeicola litoreus*	WP_377179375.1	WP_377178661.1	WP_377178571.1	WP_377178062.1
Bacteroidota	*Nemorincola caseinilytica*	WP_345083699.1	WP_345077030.1	WP_345084461.1	WP_345085155.1
Bacteroidota	*Neotamlana laminarinivorans*	WP_226539851.1	WP_226540228.1	WP_226540714.1	WP_226544469.1
Bacteroidota	*Neotamlana nanhaiensis*	WP_044624813.1	WP_044626297.1	WP_044624653.1	WP_044627344.1
Bacteroidota	*Neotamlana sargassicola*	WP_226696502.1	WP_226695741.1	WP_226695825.1	WP_226695554.1
Bacteroidota	*Neotamlana sedimentorum*	WP_044633376.1	WP_044632100.1	WP_044632017.1	WP_044633752.1
Bacteroidota	*Neptunitalea chrysea*	WP_281755024.1	WP_281752677.1	WP_281751884.1	WP_281754931.1
Bacteroidota	*Neptunitalea lumnitzerae*	WP_281764548.1	WP_281765818.1	WP_281765697.1	WP_281766268.1
Bacteroidota	*Niastella caeni*	WP_243751633.1	WP_243751588.1	WP_136576828.1	WP_136578394.1

Bacteroidota	*Niastella koreensis*	WP_014220614.1	WP_014223133.1	WP_014220554.1	WP_014218826.1
Bacteroidota	*Niastella populi*	WP_081163821.1	WP_081170820.1	WP_081163880.1	WP_081168632.1
Bacteroidota	*Niastella sp.*	WP_407746754.1	WP_407743925.1	WP_407744206.1	WP_407743428.1
Bacteroidota	*Niastella vici*	WP_081147921.1	WP_081151070.1	WP_081147991.1	WP_081155202.1
Bacteroidota	*Niastella yeongjuensis*	WP_081203523.1	WP_081199676.1	WP_081203469.1	WP_081202772.1
Bacteroidota	*Nibribacter koreensis*	WP_345167043.1	WP_345169332.1	WP_345162107.1	WP_345165865.1
Bacteroidota	*Nibribacter ruber*	WP_160692106.1	WP_160690431.1	WP_160694538.1	WP_160692977.1
Bacteroidota	*Nonlabens agnitus*	WP_105981819.1	WP_105982533.1	WP_105981802.1	WP_105981856.1
Bacteroidota	*Nonlabens antarcticus*	WP_194852021.1	WP_194850349.1	WP_194852000.1	WP_194850045.1
Bacteroidota	*Nonlabens arenilitoris*	WP_170061736.1	WP_105071680.1	WP_245910827.1	WP_105072145.1
Bacteroidota	*Nonlabens dokdonensis*	WP_303686156.1	WP_015363711.1	WP_015360760.1	WP_304017239.1
Bacteroidota	*Nonlabens marinus*	WP_041497320.1	WP_041497035.1	WP_041494828.1	WP_041497313.1
Bacteroidota	*Nonlabens ponticola*	WP_241234638.1	WP_126448044.1	WP_126448506.1	WP_126448453.1
Bacteroidota	*Nonlabens sp.*	WP_292901267.1	WP_213523056.1	WP_415185567.1	WP_348116523.1
Bacteroidota	*Nonlabens spongiae*	WP_085765855.1	WP_085766589.1	WP_085765672.1	WP_085766476.1
Bacteroidota	*Nonlabens tegetincola*	WP_105018485.1	WP_338350316.1	WP_042278472.1	WP_105018588.1
Bacteroidota	*Nonlabens ulvanivorans*	WP_348378735.1	WP_348376625.1	WP_036585357.1	WP_397300323.1
Bacteroidota	*Nonlabens xiamenensis*	WP_124979799.1	WP_124981425.1	WP_124979846.1	WP_240642324.1
Bacteroidota	*Nubsella zeaxanthinifaciens*	WP_113639010.1	WP_113637362.1	WP_113635575.1	WP_427531266.1
Bacteroidota	*Oceanihabitans sediminis*	WP_113966091.1	WP_072349045.1	WP_072351629.1	WP_318933800.1
Bacteroidota	*Oceanihabitans sp. 2_MG-2023*	WP_303424503.1	WP_303425751.1	WP_303424129.1	WP_303424880.1
Bacteroidota	*Ochrovirga pacifica*	WP_010136408.1	WP_010136703.1	WP_010135397.1	WP_010134725.1
Bacteroidota	*Olivibacter sp. SDN3*	WP_187340080.1	WP_187341938.1	WP_187339661.1	WP_187340539.1
Bacteroidota	*Olleya aquimaris*	WP_111659532.1	WP_111659383.1	WP_111659072.1	WP_111660549.1
Bacteroidota	*Olleya marilimosa*	WP_339875171.1	WP_028282215.1	WP_099569000.1	WP_191100015.1
Bacteroidota	*Olleya namhaensis*	WP_090838608.1	WP_281848004.1	WP_272022623.1	WP_281847549.1
Bacteroidota	*Olleya sp. UBA1516*	WP_292949009.1	WP_292945675.1	WP_292949436.1	WP_292943930.1
Bacteroidota	*Owenweeksia hongkongensis*	WP_417592092.1	WP_014201498.1	WP_014201423.1	WP_417601339.1

Bacteroidota	*Paenimyroides aestuarii*	WP_257498959.1	WP_257498498.1	WP_257498436.1	WP_257498655.1
Bacteroidota	*Paenimyroides baculatum*	WP_150010046.1	WP_150014147.1	WP_150014239.1	WP_150011125.1
Bacteroidota	*Paenimyroides ceti*	WP_290363757.1	WP_290362695.1	WP_290363368.1	WP_290363635.1
Bacteroidota	*Paenimyroides marinum*	WP_091095447.1	WP_091095377.1	WP_091095373.1	WP_091095038.1
Bacteroidota	*Paenimyroides tangerinum*	WP_125019654.1	WP_125018708.1	WP_125017044.1	WP_125017160.1
Bacteroidota	*Paenimyroides ummariense*	WP_091519820.1	WP_091522971.1	WP_091526027.1	WP_091521721.1
Bacteroidota	*Paenimyroides viscosum*	WP_124898542.1	WP_124899808.1	WP_124898830.1	WP_124898761.1
Bacteroidota	*Panacibacter ginsenosidivorans*	WP_147187667.1	WP_147189023.1	WP_147192662.1	WP_147188223.1
Bacteroidota	*Panacibacter microcysteis*	WP_196991769.1	WP_196990551.1	WP_196991319.1	WP_196991932.1
Bacteroidota	*Paracrocinitomix mangrovi*	WP_221836281.1	WP_221834572.1	WP_221834850.1	WP_221836018.1
Bacteroidota	*Paradesertivirga mongoliensis*	WP_255903222.1	WP_255897988.1	WP_255902703.1	WP_255902177.1
Bacteroidota	*Parafilimonas terrae*	WP_090656488.1	WP_090655776.1	WP_245751513.1	WP_090658732.1
Bacteroidota	*Paraflavisolibacter caeni*	WP_279295253.1	WP_279295855.1	WP_279299632.1	WP_279297706.1
Bacteroidota	*Paraflavisolibacter sp. H34*	WP_336719945.1	WP_336722650.1	WP_336725373.1	WP_336725333.1
Bacteroidota	*Pararcticibacter amylolyticus*	WP_109416670.1	WP_109416979.1	WP_109414587.1	WP_109416868.1
Bacteroidota	*Parasediminibacterium paludis*	WP_379011984.1	WP_379013407.1	WP_379014846.1	WP_379012157.1
Bacteroidota	*Parasediminibacterium sp. JCM 36343*	WP_410846444.1	WP_410844105.1	WP_410844722.1	WP_410847215.1
Bacteroidota	*Parasegetibacter sp. NRK P23*	WP_251757843.1	WP_251761400.1	WP_251758786.1	WP_251759009.1
Bacteroidota	*Parvicella tangerina*	WP_258540467.1	WP_258541382.1	WP_258543270.1	WP_258541479.1
Bacteroidota	*Patiriisocius hiemis*	WP_311333773.1	WP_311331756.1	WP_311331402.1	WP_311331573.1
Bacteroidota	*Patiriisocius marinistellae*	WP_151894487.1	WP_151894385.1	WP_151894039.1	WP_151894628.1
Bacteroidota	*Patiriisocius marinus*	WP_151675022.1	WP_151673679.1	WP_151673105.1	WP_151673038.1
Bacteroidota	*Patiriisocius sp. Uisw_047*	WP_415371134.1	WP_415371185.1	WP_415370858.1	WP_415370868.1
Bacteroidota	*Paucihalobacter ruber*	WP_140990968.1	WP_140990425.1	WP_140990909.1	WP_140990640.1
Bacteroidota	*Paucihalobacter sp.*	WP_334111456.1	WP_334112096.1	WP_334113206.1	WP_334111572.1
Bacteroidota	*Pedobacter africanus*	WP_310140594.1	WP_310142609.1	WP_310141383.1	WP_084237520.1
Bacteroidota	*Pedobacter agri*	WP_116167521.1	WP_316805750.1	WP_316806296.1	WP_316808301.1
Bacteroidota	*Pedobacter albus*	WP_330106040.1	WP_330109376.1	WP_330105924.1	WP_330107668.1

Bacteroidota	*Pedobacter alluvionis*	WP_121286791.1	WP_121287746.1	WP_121287178.1	WP_121283637.1
Bacteroidota	*Pedobacter alpinus*	WP_379040248.1	WP_379048161.1	WP_379040262.1	WP_379047338.1
Bacteroidota	*Pedobacter antarcticus*	WP_037439044.1	WP_219224099.1	WP_037442773.1	WP_316744585.1
Bacteroidota	*Pedobacter aquae*	WP_149073860.1	WP_149074866.1	WP_149073748.1	WP_149074685.1
Bacteroidota	*Pedobacter aquatilis*	WP_316736527.1	WP_316734886.1	WP_316738179.1	WP_316756619.1
Bacteroidota	*Pedobacter arcticus*	WP_017258694.1	WP_017256750.1	WP_017259123.1	WP_040539760.1
Bacteroidota	*Pedobacter borealis*	WP_029275995.1	WP_029280013.1	WP_029278913.1	WP_029276083.1
Bacteroidota	*Pedobacter boryungensis*	WP_173270125.1	WP_173272792.1	WP_173273727.1	WP_173273906.1
Bacteroidota	*Pedobacter caeni*	WP_073233220.1	WP_073232208.1	WP_073234076.1	WP_073229707.1
Bacteroidota	*Pedobacter changchengzhani*	WP_133262608.1	WP_133263516.1	WP_133262326.1	WP_133261648.1
Bacteroidota	*Pedobacter chinensis*	WP_115403602.1	WP_115402615.1	WP_115404077.1	WP_115404238.1
Bacteroidota	*Pedobacter chitinilyticus*	WP_113648656.1	WP_113648051.1	WP_113645262.1	WP_113647052.1
Bacteroidota	*Pedobacter cryoconitis*	WP_111632184.1	WP_184624986.1	WP_068402913.1	WP_183885582.1
Bacteroidota	*Pedobacter cryophilus*	WP_136827635.1	WP_136825249.1	WP_136826977.1	WP_317130548.1
Bacteroidota	*Pedobacter cryotolerans*	WP_136877078.1	WP_136874354.1	WP_136876996.1	WP_136878258.1
Bacteroidota	*Pedobacter duraquae*	WP_133553010.1	WP_133556412.1	WP_133553364.1	WP_133555384.1
Bacteroidota	*Pedobacter endophyticus*	WP_196098957.1	WP_196100950.1	WP_196099093.1	WP_196100596.1
Bacteroidota	*Pedobacter fastidiosus*	WP_187071800.1	WP_187069658.1	WP_187071857.1	WP_187070164.1
Bacteroidota	*Pedobacter flavus*	WP_330146138.1	WP_330144997.1	WP_330145864.1	WP_330145931.1
Bacteroidota	*Pedobacter foliorum*	WP_173091633.1	WP_173090079.1	WP_173093305.1	WP_173091865.1
Bacteroidota	*Pedobacter frigidisoli*	WP_131557085.1	WP_316798605.1	WP_131561206.1	WP_316800050.1
Bacteroidota	*Pedobacter frigiditerrae*	WP_131554366.1	WP_131552253.1	WP_316768507.1	WP_131555756.1
Bacteroidota	*Pedobacter frigoris*	WP_316794001.1	WP_316793455.1	WP_316793880.1	WP_136837743.1
Bacteroidota	*Pedobacter gandavensis*	WP_281029962.1	WP_316746284.1	WP_316747424.1	WP_316840536.1
Bacteroidota	*Pedobacter ghigonis*	WP_175634016.1	WP_175636134.1	WP_175633854.1	WP_175636440.1
Bacteroidota	*Pedobacter ginsengisoli*	WP_099440970.1	WP_285059228.1	WP_285056991.1	WP_285054377.1
Bacteroidota	*Pedobacter glucosidilyticus*	WP_026904798.1	WP_026905129.1	WP_304064693.1	WP_026902661.1
Bacteroidota	*Pedobacter hartonius*	WP_090554705.1	WP_090555335.1	WP_090555089.1	WP_090556570.1

Bacteroidota	*Pedobacter helvus*	WP_138731280.1	WP_138729967.1	WP_138730109.1	WP_138728912.1
Bacteroidota	*Pedobacter heparinus*	WP_316810839.1	WP_316813681.1	WP_012781370.1	WP_015809326.1
Bacteroidota	*Pedobacter hiemivivus*	WP_131611192.1	WP_136880229.1	WP_136879996.1	WP_131607297.1
Bacteroidota	*Pedobacter insulae*	WP_090992148.1	WP_090996882.1	WP_090992110.1	WP_090996648.1
Bacteroidota	*Pedobacter jamesrossensis*	WP_378960642.1	WP_378962165.1	WP_378959945.1	WP_378962518.1
Bacteroidota	*Pedobacter jejuensis*	WP_123205183.1	WP_123204999.1	WP_123205102.1	WP_123206726.1
Bacteroidota	*Pedobacter jeongneungensis*	WP_025143861.1	WP_025144181.1	WP_025143672.1	WP_025145791.1
Bacteroidota	*Pedobacter kyungheensis*	WP_039480112.1	WP_039479543.1	WP_039479903.1	WP_039479944.1
Bacteroidota	*Pedobacter lithocola*	WP_378981640.1	WP_378987507.1	WP_378981496.1	WP_378984310.1
Bacteroidota	*Pedobacter lusitanus*	WP_041885766.1	WP_041883719.1	WP_041885191.1	WP_041887252.1
Bacteroidota	*Pedobacter mendelii*	WP_188412606.1	WP_188411375.1	WP_378957843.1	WP_188415496.1
Bacteroidota	*Pedobacter metabolipauper*	WP_133577838.1	WP_133577613.1	WP_133577989.1	WP_133577171.1
Bacteroidota	*Pedobacter miscanthi*	WP_113947079.1	WP_316824526.1	WP_113952201.1	WP_113950981.1
Bacteroidota	*Pedobacter montanisoli*	WP_243361101.1	WP_243360292.1	WP_243363041.1	WP_243360692.1
Bacteroidota	*Pedobacter mucosus*	WP_238416438.1	WP_238414581.1	WP_238414089.1	WP_238414324.1
Bacteroidota	*Pedobacter namyangjuensis*	WP_113654792.1	WP_113654293.1	WP_113653838.1	WP_113654129.1
Bacteroidota	*Pedobacter nanyangensis*	WP_113663099.1	WP_113662147.1	WP_113662675.1	WP_113661786.1
Bacteroidota	*Pedobacter nototheniae*	WP_316802806.1	WP_131539222.1	WP_316805533.1	WP_316804932.1
Bacteroidota	*Pedobacter nutrimenti*	WP_316833367.1	WP_110833957.1	WP_110834448.1	WP_316836260.1
Bacteroidota	*Pedobacter nyackensis*	WP_084287073.1	WP_316816358.1	WP_316815893.1	WP_316818807.1
Bacteroidota	*Pedobacter paludis*	WP_109930234.1	WP_109930788.1	WP_109932520.1	WP_109931200.1
Bacteroidota	*Pedobacter petrophilus*	WP_154279375.1	WP_154281502.1	WP_154282467.1	WP_154278704.1
Bacteroidota	*Pedobacter planticolens*	WP_182922054.1	WP_182922361.1	WP_182922156.1	WP_182920612.1
Bacteroidota	*Pedobacter polaris*	WP_136841322.1	WP_136838023.1	WP_136844270.1	WP_136841447.1
Bacteroidota	*Pedobacter psychrodurus*	WP_131532023.1	WP_131533002.1	WP_316846007.1	WP_131530597.1
Bacteroidota	*Pedobacter psychrophilus*	WP_068821378.1	WP_068822602.1	WP_068823604.1	WP_068822891.1
Bacteroidota	*Pedobacter psychroterrae*	WP_131596710.1	WP_131594357.1	WP_131597463.1	WP_131597727.1
Bacteroidota	*Pedobacter psychrotolerans*	WP_132535531.1	WP_132530194.1	WP_132536433.1	WP_132529721.1

Bacteroidota	*Pedobacter punctiformis*	WP_269427941.1	WP_269428406.1	WP_269427832.1	WP_269428613.1
Bacteroidota	*Pedobacter puniceum*	WP_154286206.1	WP_154288091.1	WP_154286091.1	WP_154288349.1
Bacteroidota	*Pedobacter quisquiliarum*	WP_188627291.1	WP_188626080.1	WP_188627188.1	WP_188624811.1
Bacteroidota	*Pedobacter rhizosphaerae*	WP_090880663.1	WP_090884952.1	WP_090881040.1	WP_090883636.1
Bacteroidota	*Pedobacter rhodius*	WP_269414017.1	WP_269417121.1	WP_269413907.1	WP_269416549.1
Bacteroidota	*Pedobacter roseus*	WP_187591070.1	WP_187592982.1	WP_187591282.1	WP_187591487.1
Bacteroidota	*Pedobacter sandarakinus*	WP_265856552.1	WP_265854285.1	WP_265855752.1	WP_265854120.1
Bacteroidota	*Pedobacter schmidteae*	WP_121273509.1	WP_121269434.1	WP_121273731.1	WP_121271022.1
Bacteroidota	*Pedobacter segetis*	WP_200584548.1	WP_200587972.1	WP_200585255.1	WP_200586207.1
Bacteroidota	*Pedobacter soli*	WP_090771268.1	WP_090772814.1	WP_090772601.1	WP_090766432.1
Bacteroidota	*Pedobacter sp.*	WP_353903522.1	WP_324370905.1	WP_421942790.1	WP_424057527.1
Bacteroidota	*Pedobacter steynii*	WP_069382250.1	WP_069379562.1	WP_074606995.1	WP_074611952.1
Bacteroidota	*Pedobacter suwonensis*	WP_090987543.1	WP_293742548.1	WP_293741410.1	WP_090984736.1
Bacteroidota	*Pedobacter terrae*	WP_090499916.1	WP_335317504.1	WP_335320483.1	WP_090498603.1
Bacteroidota	*Pedobacter ureilyticus*	WP_138724127.1	WP_138722568.1	WP_138723946.1	WP_138721851.1
Bacteroidota	*Pedobacter vanadiisoli*	WP_379080426.1	WP_379081851.1	WP_379080222.1	WP_379081366.1
Bacteroidota	*Pedobacter westerhofensis*	WP_142529649.1	WP_142528417.1	WP_142529467.1	WP_142530983.1
Bacteroidota	*Pedobacter xixiisoli*	WP_097131290.1	WP_097132640.1	WP_097127343.1	WP_097132993.1
Bacteroidota	*Pedobacter yonginense*	WP_109927202.1	WP_109925110.1	WP_109927344.1	WP_109926179.1
Bacteroidota	*Pedobacter yulinensis*	WP_107217006.1	WP_107214288.1	WP_107216326.1	WP_107216633.1
Bacteroidota	*Pedobacter zeae*	WP_183759580.1	WP_183765082.1	WP_183759401.1	WP_183768407.1
Bacteroidota	*Pelobium manganitolerans*	WP_120181569.1	WP_120181251.1	WP_120181798.1	WP_120181363.1
Bacteroidota	*Penaeicola halotolerans*	WP_226390012.1	WP_226390126.1	WP_226389474.1	WP_226390042.1
Bacteroidota	*Persicobacter diffluens*	WP_338235790.1	WP_338237395.1	WP_338236170.1	WP_338235858.1
Bacteroidota	*Persicobacter psychrovividus*	WP_332920859.1	WP_332922396.1	WP_338397812.1	WP_338397565.1
Bacteroidota	*Persicobacter sp. CCB-QB2*	WP_053406022.1	WP_053406831.1	WP_053404325.1	WP_053405934.1
Bacteroidota	*Phaeodactylibacter luteus*	WP_147165650.1	WP_147168864.1	WP_147166612.1	WP_147167063.1
Bacteroidota	*Phaeodactylibacter sp.*	WP_350178551.1	WP_293565027.1	WP_293566465.1	WP_350177613.1

Bacteroidota	*Phaeodactylibacter xiamenensis*	WP_044226137.1	WP_044220771.1	WP_425423020.1	WP_044226419.1
Bacteroidota	*Phnomibacter sp.*	WP_333798807.1	WP_333801136.1	WP_333798932.1	WP_333799269.1
Bacteroidota	*Pinibacter aurantiacus*	WP_217793815.1	WP_217789871.1	WP_217794396.1	WP_217791097.1
Bacteroidota	*Pinibacter soli*	WP_282335857.1	WP_282334310.1	WP_282334518.1	WP_282335433.1
Bacteroidota	*Planktosalinus lacus*	WP_188441290.1	WP_188441418.1	WP_188443026.1	WP_188439440.1
Bacteroidota	*Planobacterium oryzisoli*	WP_194738628.1	WP_194739118.1	WP_194739626.1	WP_194738760.1
Bacteroidota	*Polaribacter aestuariivivens*	WP_138535047.1	WP_138534867.1	WP_138535111.1	WP_138537235.1
Bacteroidota	*Polaribacter aquimarinus*	WP_235869146.1	WP_109404621.1	WP_109405612.1	WP_109404950.1
Bacteroidota	*Polaribacter atrinae*	WP_282073704.1	WP_341220606.1	WP_341222179.1	WP_282073884.1
Bacteroidota	*Polaribacter batillariae*	WP_207971760.1	WP_207972030.1	WP_207972378.1	WP_207971661.1
Bacteroidota	*Polaribacter butkevichii*	WP_105048181.1	WP_105048550.1	WP_105048114.1	WP_105048036.1
Bacteroidota	*Polaribacter cellanae*	WP_208079091.1	WP_208079180.1	WP_208079597.1	WP_208079626.1
Bacteroidota	*Polaribacter dokdonensis*	WP_053974795.1	WP_053974708.1	WP_053974658.1	WP_053975070.1
Bacteroidota	*Polaribacter filamentus*	WP_104808833.1	WP_104809256.1	WP_104808805.1	WP_104808646.1
Bacteroidota	*Polaribacter gangjinensis*	WP_105045698.1	WP_105045548.1	WP_105047271.1	WP_105047223.1
Bacteroidota	*Polaribacter glomeratus*	WP_105021496.1	WP_105020554.1	WP_105020177.1	WP_105020891.1
Bacteroidota	*Polaribacter haliotis*	WP_088353370.1	WP_088353541.1	WP_088353291.1	WP_088352880.1
Bacteroidota	*Polaribacter huanghezhanensis*	WP_301400178.1	WP_301400243.1	WP_301400130.1	WP_301400796.1
Bacteroidota	*Polaribacter irgensii*	WP_004570937.1	WP_026288371.1	WP_018942509.1	WP_004570425.1
Bacteroidota	*Polaribacter litorisediminis*	WP_223447258.1	WP_223446044.1	WP_223446128.1	WP_223439388.1
Bacteroidota	*Polaribacter marinivivus*	WP_377410462.1	WP_377410213.1	WP_377410502.1	WP_377409873.1
Bacteroidota	*Polaribacter marinus*	WP_242177528.1	WP_242177238.1	WP_242177468.1	WP_242176929.1
Bacteroidota	*Polaribacter pacificus*	WP_229664930.1	WP_188598849.1	WP_188598986.1	WP_188599336.1
Bacteroidota	*Polaribacter pectinis*	WP_187483182.1	WP_187483341.1	WP_187483380.1	WP_187482972.1
Bacteroidota	*Polaribacter ponticola*	WP_265724611.1	WP_265724554.1	WP_265724156.1	WP_274270124.1
Bacteroidota	*Polaribacter porphyrae*	WP_105014403.1	WP_105014864.1	WP_105014974.1	WP_105015495.1
Bacteroidota	*Polaribacter reichenbachii*	WP_068360296.1	WP_068360224.1	WP_068360040.1	WP_068364870.1
Bacteroidota	*Polaribacter sejongensis*	WP_261974070.1	WP_208889400.1	WP_261973620.1	WP_373940948.1

Bacteroidota	*Polaribacter septentrionalilitoris*	WP_159947717.1	WP_159947352.1	WP_159947839.1	WP_159948279.1
Bacteroidota	*Polaribacter sp.*	WP_348024623.1	WP_296637153.1	WP_348023849.1	WP_404486996.1
Bacteroidota	*Polaribacter staleyi*	WP_343329531.1	WP_343329764.1	WP_343329481.1	WP_343329414.1
Bacteroidota	*Polaribacter tangerinus*	WP_088322715.1	WP_088322902.1	WP_088322791.1	WP_088322699.1
Bacteroidota	*Polaribacter vadi*	WP_065319297.1	WP_065318254.1	WP_065318000.1	WP_065320679.1
Bacteroidota	*Polluticaenibacter yanchengensis*	WP_407031991.1	WP_407031566.1	WP_407030962.1	WP_407032955.1
Bacteroidota	*Polluticoccus soli*	WP_276134737.1	WP_276134619.1	WP_276134772.1	WP_276132203.1
Bacteroidota	*Pontibacter actiniarum*	WP_025608587.1	WP_025607855.1	WP_025605102.1	WP_084196269.1
Bacteroidota	*Pontibacter akesuensis*	WP_068837678.1	WP_068837038.1	WP_068839774.1	WP_082815155.1
Bacteroidota	*Pontibacter amylolyticus*	WP_188500845.1	WP_188502506.1	WP_188499622.1	WP_188500582.1
Bacteroidota	*Pontibacter anaerobius*	WP_266052702.1	WP_266051738.1	WP_266054022.1	WP_266054192.1
Bacteroidota	*Pontibacter aquaedesilientis*	WP_191183522.1	WP_191182936.1	WP_191182164.1	WP_224744371.1
Bacteroidota	*Pontibacter arcticus*	WP_112304594.1	WP_112306243.1	WP_112305500.1	WP_239020921.1
Bacteroidota	*Pontibacter aydingkolensis*	WP_219876992.1	WP_219876295.1	WP_219878462.1	WP_246596896.1
Bacteroidota	*Pontibacter beigongshangensis*	WP_187262260.1	WP_187260967.1	WP_187260582.1	WP_187264052.1
Bacteroidota	*Pontibacter burrus*	WP_163911654.1	WP_163915162.1	WP_163914727.1	WP_163910788.1
Bacteroidota	*Pontibacter cellulosilyticus*	WP_187068082.1	WP_187067611.1	WP_187065817.1	WP_230407259.1
Bacteroidota	*Pontibacter chinhatensis*	WP_092100996.1	WP_092099925.1	WP_092098427.1	WP_092101233.1
Bacteroidota	*Pontibacter chitinilyticus*	WP_347157579.1	WP_347159781.1	WP_347157039.1	WP_347157394.1
Bacteroidota	*Pontibacter diazotrophicus*	WP_115566407.1	WP_115563627.1	WP_115565944.1	WP_246000815.1
Bacteroidota	*Pontibacter fetidus*	WP_162344978.1	WP_162345166.1	WP_162344390.1	WP_162346564.1
Bacteroidota	*Pontibacter flavimaris*	WP_073851502.1	WP_170866119.1	WP_073851109.1	WP_073851885.1
Bacteroidota	*Pontibacter harenae*	WP_229967464.1	WP_229972779.1	WP_229969948.1	WP_229968909.1
Bacteroidota	*Pontibacter indicus*	WP_076668258.1	WP_170871914.1	WP_076666330.1	WP_244554682.1
Bacteroidota	*Pontibacter kalidii*	WP_266203549.1	WP_266202819.1	WP_266205387.1	WP_266203411.1
Bacteroidota	*Pontibacter korlensis*	WP_046309691.1	WP_046308896.1	WP_046312400.1	WP_084694729.1
Bacteroidota	*Pontibacter liquoris*	WP_367615719.1	WP_242916368.1	WP_242918685.1	WP_242921285.1
Bacteroidota	*Pontibacter litorisediminis*	WP_276498473.1	WP_276499133.1	WP_276495720.1	WP_276498631.1

Bacteroidota	*Pontibacter locisalis*	WP_377506203.1	WP_377507272.1	WP_377512209.1	WP_377503573.1
Bacteroidota	*Pontibacter mangrovi*	WP_140620258.1	WP_140621972.1	WP_140618665.1	WP_239022830.1
Bacteroidota	*Pontibacter mucosus*	WP_108210053.1	WP_108212430.1	WP_108213444.1	WP_108210166.1
Bacteroidota	*Pontibacter oryzae*	WP_119432228.1	WP_119430834.1	WP_119431269.1	WP_119432100.1
Bacteroidota	*Pontibacter pamirensis*	WP_162054115.1	WP_162053720.1	WP_162052484.1	WP_237144869.1
Bacteroidota	*Pontibacter populi*	WP_350413596.1	WP_350410730.1	WP_350412632.1	WP_350411634.1
Bacteroidota	*Pontibacter pudoricolor*	WP_162428158.1	WP_162428816.1	WP_162426266.1	WP_162427917.1
Bacteroidota	*Pontibacter qinzhouensis*	WP_147920392.1	WP_147922611.1	WP_147923662.1	WP_255474196.1
Bacteroidota	*Pontibacter ramchanderi*	WP_101444762.1	WP_101446093.1	WP_101442876.1	WP_180336380.1
Bacteroidota	*Pontibacter roseus*	WP_018476290.1	WP_211214358.1	WP_018478075.1	WP_018476588.1
Bacteroidota	*Pontibacter ruber*	WP_250428584.1	WP_250431823.1	WP_250429605.1	WP_250428291.1
Bacteroidota	*Pontibacter rugosus*	WP_377521968.1	WP_377526565.1	WP_377529224.1	WP_377526710.1
Bacteroidota	*Pontibacter saemangeumensis*	WP_345160897.1	WP_345161860.1	WP_345157442.1	WP_345159741.1
Bacteroidota	*Pontibacter sp. H259*	WP_340327220.1	WP_340325105.1	WP_340324301.1	WP_340326631.1
Bacteroidota	*Pontibacter toksunensis*	WP_377482369.1	WP_377487128.1	WP_377479545.1	WP_377485187.1
Bacteroidota	*Pontibacter ummariensis*	WP_089318033.1	WP_089319534.1	WP_089317724.1	WP_089319831.1
Bacteroidota	*Pontibacter virosus*	WP_116543722.1	WP_207774867.1	WP_116543526.1	WP_116541785.1
Bacteroidota	*Pontibacter vulgaris*	WP_242926206.1	WP_242927759.1	WP_242928228.1	WP_242926450.1
Bacteroidota	*Pontimicrobium aquaticum*	WP_136844426.1	WP_136844924.1	WP_136842894.1	WP_136842336.1
Bacteroidota	*Pontimicrobium sp. IMCC45349*	WP_412984746.1	WP_412984909.1	WP_412985655.1	WP_412984315.1
Bacteroidota	*Porifericola rhodea*	WP_302236952.1	WP_302241969.1	WP_302238587.1	WP_302241977.1
Bacteroidota	*Postechiella marina*	WP_344788461.1	WP_344786197.1	WP_344786381.1	WP_344789558.1
Bacteroidota	*Pseudalgibacter alginicilyticus*	WP_054723694.1	WP_054724859.1	WP_054724689.1	WP_054725144.1
Bacteroidota	*Pseudarcicella hirudinis*	WP_092019125.1	WP_092018740.1	WP_092016199.1	WP_092018609.1
Bacteroidota	*Pseudobacter ginsenosidimutans*	WP_130543858.1	WP_130542873.1	WP_130543804.1	WP_130540330.1
Bacteroidota	*Pseudoflavitalea rhizosphaerae*	WP_127132024.1	WP_127128830.1	WP_127132125.1	WP_127125031.1
Bacteroidota	*Pseudofulvibacter geojedonensis*	WP_377714822.1	WP_377715660.1	WP_377712568.1	WP_377713360.1
Bacteroidota	*Pseudopedobacter beijingensis*	WP_379663189.1	WP_379661639.1	WP_379663639.1	WP_379660747.1

Bacteroidota	*Pseudopedobacter saltans*	WP_013633114.1	WP_013634471.1	WP_013633057.1	WP_013631349.1
Bacteroidota	*Pseudopedobacter sp.*	WP_353133445.1	WP_353133078.1	WP_353136078.1	WP_353135823.1
Bacteroidota	*Pseudotamlana agarivorans*	WP_216068594.1	WP_067147999.1	WP_216068537.1	WP_067145892.1
Bacteroidota	*Pseudotamlana carrageenivorans*	WP_102996449.1	WP_102996535.1	WP_102996350.1	WP_102996863.1
Bacteroidota	*Pseudotamlana haliotis*	WP_150935729.1	WP_150935676.1	WP_150937802.1	WP_150940490.1
Bacteroidota	*Pseudotenacibaculum haliotis*	WP_379666458.1	WP_379666268.1	WP_379666551.1	WP_379664548.1
Bacteroidota	*Pseudotenacibaculum sp. MALMAid0570*	WP_349553689.1	WP_349553908.1	WP_349553590.1	WP_349554108.1
Bacteroidota	*Psychroflexus maritimus*	WP_166400925.1	WP_166400947.1	WP_166400890.1	WP_166399151.1
Bacteroidota	*Psychroflexus salarius*	WP_073193621.1	WP_073191291.1	WP_073190977.1	WP_073193286.1
Bacteroidota	*Psychroflexus sp. ALD_RP9*	WP_207038279.1	WP_207038686.1	WP_207038581.1	WP_207037867.1
Bacteroidota	*Psychroserpens algicola*	WP_204345880.1	WP_248413321.1	WP_248411954.1	WP_204346159.1
Bacteroidota	*Psychroserpens burtonensis*	WP_147231107.1	WP_028870830.1	WP_028872699.1	WP_028872803.1
Bacteroidota	*Psychroserpens damuponensis*	WP_040282237.1	WP_040279120.1	WP_040280953.1	WP_040281111.1
Bacteroidota	*Psychroserpens jangbogonensis*	WP_052172542.1	WP_033959147.1	WP_033959080.1	WP_033956243.1
Bacteroidota	*Psychroserpens luteolus*	WP_230935730.1	WP_230935135.1	WP_230935982.1	WP_230936893.1
Bacteroidota	*Psychroserpens luteus*	WP_194507887.1	WP_194507318.1	WP_194507631.1	WP_194508324.1
Bacteroidota	*Psychroserpens mesophilus*	WP_040250502.1	WP_040251637.1	WP_174435320.1	WP_040250904.1
Bacteroidota	*Psychroserpens ponticola*	WP_249995719.1	WP_249995923.1	WP_249995559.1	WP_249995438.1
Bacteroidota	*Psychroserpens sp.*	WP_425223521.1	WP_323789418.1	WP_335728066.1	WP_415224132.1
Bacteroidota	*Puia dinghuensis*	WP_188929662.1	WP_188937963.1	WP_188929879.1	WP_188927805.1
Bacteroidota	*Puia sp.*	WP_325593104.1	WP_331581891.1	WP_325587851.1	WP_325588515.1
Bacteroidota	*Pustulibacterium marinum*	WP_093023466.1	WP_093026409.1	WP_093025214.1	WP_093023162.1
Bacteroidota	*Putridiphycobacter roseus*	WP_111063168.1	WP_111062192.1	WP_111062136.1	WP_111061356.1
Bacteroidota	*Raineya orbicola*	WP_101358011.1	WP_101359701.1	WP_101357744.1	WP_165778063.1
Bacteroidota	*Rapidithrix thailandica*	WP_346820272.1	WP_346820230.1	WP_346819169.1	WP_346819304.1
Bacteroidota	*Rasiella rasia*	WP_164678329.1	WP_164678373.1	WP_164678848.1	WP_164679013.1
Bacteroidota	*Reichenbachiella agariperforans*	WP_216011801.1	WP_073124535.1	WP_073123459.1	WP_073122811.1
Bacteroidota	*Reichenbachiella agarivorans*	WP_262310620.1	WP_262309824.1	WP_262308525.1	WP_262310351.1

Bacteroidota	*Reichenbachiella carrageenanivorans*	WP_263052273.1	WP_263050107.1	WP_263049587.1	WP_263051374.1
Bacteroidota	*Reichenbachiella faecimaris*	WP_084372697.1	WP_084371036.1	WP_084371183.1	WP_084371882.1
Bacteroidota	*Reichenbachiella sp.*	WP_318169581.1	WP_348120269.1	WP_348463629.1	WP_422361535.1
Bacteroidota	*Reichenbachiella ulvae*	WP_264138846.1	WP_264137898.1	WP_264137298.1	WP_264138500.1
Bacteroidota	*Reichenbachiella versicolor*	WP_109832983.1	WP_109829597.1	WP_109831061.1	WP_109832016.1
Bacteroidota	*Rhodoflexus caldus*	WP_250632542.1	WP_250631486.1	WP_250632666.1	WP_250629984.1
Bacteroidota	*Robertkochia marina*	WP_136336582.1	WP_136336830.1	WP_136335742.1	WP_136336997.1
Bacteroidota	*Robertkochia sediminum*	WP_203058860.1	WP_203061522.1	WP_203060466.1	WP_203059512.1
Bacteroidota	*Robertkochia sp. 3YJGBD-33*	WP_224484802.1	WP_224483128.1	WP_224484353.1	WP_224484607.1
Bacteroidota	*Roseivirga sp. BDSF3-8*	WP_371908952.1	WP_371908042.1	WP_371910304.1	WP_371908816.1
Bacteroidota	*Rufibacter aurantiacus*	WP_246853757.1	WP_210487332.1	WP_210488992.1	WP_210488416.1
Bacteroidota	*Rufibacter glacialis*	WP_149096627.1	WP_149097938.1	WP_149098143.1	WP_149098844.1
Bacteroidota	*Rufibacter hautae*	WP_317132021.1	WP_149088950.1	WP_149091153.1	WP_149089626.1
Bacteroidota	*Rufibacter immobilis*	WP_377060888.1	WP_377062404.1	WP_123134153.1	WP_123133193.1
Bacteroidota	*Rufibacter latericius*	WP_123128600.1	WP_123125038.1	WP_123125940.1	WP_123126617.1
Bacteroidota	*Rufibacter psychrotolerans*	WP_205501396.1	WP_205502872.1	WP_205500681.1	WP_205500297.1
Bacteroidota	*Rufibacter radiotolerans*	WP_048922194.1	WP_048921188.1	WP_048920482.1	WP_048921794.1
Bacteroidota	*Rufibacter roseolus*	WP_246849904.1	WP_210463819.1	WP_210464973.1	WP_210463158.1
Bacteroidota	*Rufibacter roseus*	WP_066620381.1	WP_066618735.1	WP_066621324.1	WP_066623342.1
Bacteroidota	*Rufibacter sediminis*	WP_186635039.1	WP_186640101.1	WP_186636070.1	WP_186639587.1
Bacteroidota	*Rufibacter sp. LB8*	WP_192822645.1	WP_192820106.1	WP_192820940.1	WP_192822191.1
Bacteroidota	*Rufibacter tibetensis*	WP_062543512.1	WP_062542449.1	WP_062545229.1	WP_062543027.1
Bacteroidota	*Rurimicrobium arvi*	WP_344824269.1	WP_344826516.1	WP_344828551.1	WP_344822986.1
Bacteroidota	*Sabulibacter ruber*	WP_207433634.1	WP_207435692.1	WP_207430476.1	WP_207434099.1
Bacteroidota	*Salibacter sp.*	WP_310682266.1	WP_310667847.1	WP_310667026.1	WP_310667442.1
Bacteroidota	*Salinimicrobium catena*	WP_093112977.1	WP_093112685.1	WP_334213357.1	WP_334214643.1
Bacteroidota	*Salinimicrobium flavum*	WP_380753729.1	WP_380752956.1	WP_380747343.1	WP_380748127.1
Bacteroidota	*Salinimicrobium gaetbulicola*	WP_380736476.1	WP_380736752.1	WP_380736910.1	WP_380737270.1

Bacteroidota	*Salinimicrobium marinum*	WP_189606297.1	WP_189603621.1	WP_189604486.1	WP_189604708.1
Bacteroidota	*Salinimicrobium oceani*	WP_168139201.1	WP_168138491.1	WP_168136558.1	WP_168136841.1
Bacteroidota	*Salinimicrobium profundisediminis*	WP_266068498.1	WP_266068280.1	WP_266070952.1	WP_266070336.1
Bacteroidota	*Salinimicrobium sediminis*	WP_097056427.1	WP_097056067.1	WP_097056276.1	WP_097057124.1
Bacteroidota	*Salinimicrobium soli*	WP_418336149.1	WP_418335852.1	WP_418335645.1	WP_418334907.1
Bacteroidota	*Salinimicrobium sp. 3283s*	WP_345598467.1	WP_345598592.1	WP_345598933.1	WP_345598169.1
Bacteroidota	*Salinimicrobium terrae*	WP_029034933.1	WP_029033011.1	WP_029033552.1	WP_029034990.1
Bacteroidota	*Salinimicrobium tongyeongense*	WP_265163318.1	WP_265163551.1	WP_265163274.1	WP_265164049.1
Bacteroidota	*Salinimicrobium xinjiangense*	WP_029036764.1	WP_029036068.1	WP_029037520.1	WP_029038605.1
Bacteroidota	*Sandaracinomonas limnophila*	WP_127803033.1	WP_127804355.1	WP_127802513.1	WP_127803171.1
Bacteroidota	*Sanyastnella coralliicola*	WP_306644150.1	WP_306643031.1	WP_306639898.1	WP_306643246.1
Bacteroidota	*Saprospira grandis*	WP_002661067.1	WP_270100066.1	WP_015691616.1	WP_002660161.1
Bacteroidota	*Saprospira sp. CCB-QB6*	WP_272619536.1	WP_272620540.1	WP_272620609.1	WP_272620088.1
Bacteroidota	*Schleiferia thermophila*	WP_037356799.1	WP_037355887.1	WP_037358008.1	WP_160171979.1
Bacteroidota	*Sediminibacter sp. Hel_I_10*	WP_026755642.1	WP_026755702.1	WP_026755964.1	WP_026753280.1
Bacteroidota	*Sediminibacterium ginsengisoli*	WP_078832212.1	WP_078831188.1	WP_078832335.1	WP_245825700.1
Bacteroidota	*Sediminibacterium goheungense*	WP_133475208.1	WP_133474678.1	WP_133475808.1	WP_133475323.1
Bacteroidota	*Sediminibacterium roseum*	WP_161819353.1	WP_161817057.1	WP_161818326.1	WP_161817922.1
Bacteroidota	*Sediminibacterium salmoneum*	WP_026763428.1	WP_026764238.1	WP_026763456.1	WP_026763181.1
Bacteroidota	*Sediminibacterium soli*	WP_161834845.1	WP_161835386.1	WP_161834296.1	WP_161834578.1
Bacteroidota	*Sediminibacterium sp.*	WP_324483938.1	WP_324486156.1	WP_305536514.1	WP_305534927.1
Bacteroidota	*Sediminitomix flava*	WP_109622381.1	WP_109616372.1	WP_109618873.1	WP_245935572.1
Bacteroidota	*Segetibacter sp. 3557_3*	WP_133268512.1	WP_133268197.1	WP_133266548.1	WP_133267570.1
Bacteroidota	*Seonamhaeicola algicola*	WP_147133561.1	WP_147130549.1	WP_147133745.1	WP_147133851.1
Bacteroidota	*Seonamhaeicola aphaedonensis*	WP_116524709.1	WP_116039943.1	WP_116523873.1	WP_116524046.1
Bacteroidota	*Seonamhaeicola marinus*	WP_148544647.1	WP_148541872.1	WP_148544579.1	WP_262713610.1
Bacteroidota	*Seonamhaeicola maritimus*	WP_147766866.1	WP_147767208.1	WP_147769343.1	WP_282136242.1
Bacteroidota	*Seonamhaeicola sediminis*	WP_133356969.1	WP_133356602.1	WP_133357173.1	WP_133354879.1

Bacteroidota	*Seonamhaeicola sp.*	WP_299549661.1	WP_299547549.1	WP_299550404.1	WP_372938276.1
Bacteroidota	*Shiella aurantiaca*	WP_320004588.1	WP_320003240.1	WP_320003921.1	WP_320004771.1
Bacteroidota	*Siansivirga zeaxanthinifaciens*	WP_044638673.1	WP_044638880.1	WP_044638641.1	WP_044638743.1
Bacteroidota	*Snuella lapsa*	WP_345003992.1	WP_345003601.1	WP_345003664.1	WP_345007529.1
Bacteroidota	*Snuella sedimenti*	WP_199115323.1	WP_199115617.1	WP_199116703.1	WP_199114629.1
Bacteroidota	*Solirubrum puertoriconensis*	WP_059071577.1	WP_059067657.1	WP_059071390.1	WP_059068878.1
Bacteroidota	*Solitalea canadensis*	WP_014680599.1	WP_014682578.1	WP_014680712.1	WP_014681131.1
Bacteroidota	*Solitalea koreensis*	WP_142603940.1	WP_142603364.1	WP_142604438.1	WP_142600541.1
Bacteroidota	*Solitalea lacus*	WP_237846350.1	WP_237848968.1	WP_237846591.1	WP_237847143.1
Bacteroidota	*Solitalea longa*	WP_103789570.1	WP_103787391.1	WP_103790159.1	WP_103788875.1
Bacteroidota	*Soonwooa buanensis*	WP_079665548.1	WP_079668157.1	WP_079667385.1	WP_079666718.1
Bacteroidota	*Soonwooa sp.*	WP_312324244.1	WP_312322246.1	WP_300673687.1	WP_313027157.1
Bacteroidota	*Spongiivirga citrea*	WP_164029590.1	WP_164029170.1	WP_164028867.1	WP_164032428.1
Bacteroidota	*Spongiivirga sp. MCCC 1A20706*	WP_410484143.1	WP_410484950.1	WP_410483661.1	WP_410485712.1
Bacteroidota	*Sporocytophaga myxococcoides*	WP_045468567.1	WP_045459397.1	WP_045464077.1	WP_045459954.1
Bacteroidota	*Sporocytophaga sp.*	WP_293893448.1	WP_293897300.1	WP_293894613.1	WP_293892478.1
Bacteroidota	*Subsaxibacter sp. CAU 1640*	WP_248024861.1	WP_248025836.1	WP_248025383.1	WP_248024187.1
Bacteroidota	*Subsaximicrobium wynnwilliamsii*	WP_147085143.1	WP_147084534.1	WP_147085199.1	WP_317128650.1
Bacteroidota	*Sungkyunkwania multivorans*	WP_386403032.1	WP_386407340.1	WP_386405566.1	WP_386407050.1
Bacteroidota	*Taibaiella chishuiensis*	WP_106521218.1	WP_106525076.1	WP_106521535.1	WP_181358454.1
Bacteroidota	*Taibaiella helva*	WP_118952493.1	WP_118953545.1	WP_118949944.1	WP_157976869.1
Bacteroidota	*Taibaiella koreensis*	WP_118973686.1	WP_118972826.1	WP_118974487.1	WP_162903180.1
Bacteroidota	*Taibaiella lutea*	WP_150032017.1	WP_150031081.1	WP_150033014.1	WP_190277335.1
Bacteroidota	*Taibaiella soli*	WP_243630355.1	WP_110999133.1	WP_110996983.1	WP_110999509.1
Bacteroidota	*Taibaiella sp. KBW10*	WP_124634811.1	WP_124636061.1	WP_124635204.1	WP_124636155.1
Bacteroidota	*Taishania pollutisoli*	WP_163493010.1	WP_163492192.1	WP_163490697.1	WP_216714193.1
Bacteroidota	*Tamlana crocina*	WP_167917346.1	WP_167917677.1	WP_167919677.1	WP_167918689.1
Bacteroidota	*Tamlana flava*	WP_370480120.1	WP_370479806.1	WP_370476504.1	WP_370476851.1

Bacteroidota	*Tamlana sp. 2201CG12-4*	WP_326284301.1	WP_326283243.1	WP_326284009.1	WP_326282716.1
Bacteroidota	*Tenacibaculum adriaticum*	WP_148869522.1	WP_148869874.1	WP_148871029.1	WP_148871047.1
Bacteroidota	*Tenacibaculum aestuarii*	WP_408036025.1	WP_408035614.1	WP_408036213.1	WP_408036178.1
Bacteroidota	*Tenacibaculum aestuariivivum*	WP_418650629.1	WP_418650557.1	WP_418649929.1	WP_418649959.1
Bacteroidota	*Tenacibaculum agarivorans*	WP_075340842.1	WP_075342671.1	WP_075343661.1	WP_075343920.1
Bacteroidota	*Tenacibaculum aiptasiae*	WP_150901146.1	WP_150899186.1	WP_150899780.1	WP_272151765.1
Bacteroidota	*Tenacibaculum amylolyticum*	WP_408038028.1	WP_408039680.1	WP_408039792.1	WP_408037409.1
Bacteroidota	*Tenacibaculum ascidiaceicola*	WP_415268602.1	WP_425658519.1	WP_415268493.1	WP_425659246.1
Bacteroidota	*Tenacibaculum caenipelagi*	WP_133534370.1	WP_133536489.1	WP_133534486.1	WP_133534460.1
Bacteroidota	*Tenacibaculum crassostreae*	WP_408046608.1	WP_408046929.1	WP_408046466.1	WP_408046492.1
Bacteroidota	*Tenacibaculum dicentrarchi*	WP_214985934.1	WP_370407873.1	WP_101902640.1	WP_101902347.1
Bacteroidota	*Tenacibaculum discolor*	WP_121147477.1	WP_121146781.1	WP_124590251.1	WP_121148738.1
Bacteroidota	*Tenacibaculum finnmarkense*	WP_239780004.1	WP_232122331.1	WP_101915365.1	WP_232128703.1
Bacteroidota	*Tenacibaculum gallaicum*	WP_115900783.1	WP_115901618.1	WP_115900961.1	WP_115900927.1
Bacteroidota	*Tenacibaculum geojense*	WP_386105148.1	WP_386104704.1	WP_386104940.1	WP_386104979.1
Bacteroidota	*Tenacibaculum holothuriorum*	WP_198938355.1	WP_086029382.1	WP_086030732.1	WP_086030767.1
Bacteroidota	*Tenacibaculum insulae*	WP_418644920.1	WP_418644914.1	WP_418644674.1	WP_418644704.1
Bacteroidota	*Tenacibaculum jejuense*	WP_095071561.1	WP_095072327.1	WP_095073068.1	WP_095072980.1
Bacteroidota	*Tenacibaculum larymnensis*	WP_274641245.1	WP_274639816.1	WP_274640215.1	WP_274640185.1
Bacteroidota	*Tenacibaculum litopenaei*	WP_408044355.1	WP_408044075.1	WP_408043301.1	WP_408044502.1
Bacteroidota	*Tenacibaculum lutimaris*	WP_120187015.1	WP_120186681.1	WP_120187165.1	WP_120187140.1
Bacteroidota	*Tenacibaculum maritimum*	WP_024741845.1	WP_159246260.1	WP_159320935.1	WP_406755780.1
Bacteroidota	*Tenacibaculum mesophilum*	WP_394421808.1	WP_047788878.1	WP_253679894.1	WP_047789209.1
Bacteroidota	*Tenacibaculum ovolyticum*	WP_237275937.1	WP_064966370.1	WP_271241654.1	WP_064968091.1
Bacteroidota	*Tenacibaculum pelagium*	WP_182123875.1	WP_182123523.1	WP_182123957.1	WP_182123931.1
Bacteroidota	*Tenacibaculum piscium*	WP_101917121.1	WP_233900280.1	WP_233898313.1	WP_233901351.1
Bacteroidota	*Tenacibaculum platacis*	WP_348725701.1	WP_348712205.1	WP_348742779.1	WP_348742864.1
Bacteroidota	*Tenacibaculum polynesiense*	WP_348718339.1	WP_348716780.1	WP_348715293.1	WP_348718874.1

Bacteroidota	*Tenacibaculum sediminilitoris*	WP_408024488.1	WP_408024139.1	WP_408023732.1	WP_408025091.1
Bacteroidota	*Tenacibaculum singaporense*	WP_125067213.1	WP_125345071.1	WP_125068073.1	WP_125344444.1
Bacteroidota	*Tenacibaculum skagerrakense*	WP_132794118.1	WP_132794789.1	WP_132795009.1	WP_132795025.1
Bacteroidota	*Tenacibaculum soleae*	WP_271405274.1	WP_068705066.1	WP_303558687.1	WP_303529621.1
Bacteroidota	*Tenacibaculum sp.*	WP_417785267.1	WP_273694360.1	WP_417786261.1	WP_273694783.1
Bacteroidota	*Tenacibaculum tangerinum*	WP_279652504.1	WP_279652411.1	WP_279651372.1	WP_279651401.1
Bacteroidota	*Tenacibaculum todarodis*	WP_072554693.1	WP_072554883.1	WP_072555161.1	WP_072555142.1
Bacteroidota	*Tenacibaculum vairaonense*	WP_348747241.1	WP_348740343.1	WP_348746997.1	WP_348702758.1
Bacteroidota	*Tenacibaculum xiamenense*	WP_408032529.1	WP_408031608.1	WP_408030665.1	WP_408032154.1
Bacteroidota	*Terrimonas pollutisoli*	WP_276503523.1	WP_276500859.1	WP_276504086.1	WP_276502652.1
Bacteroidota	*Terrimonas sp. R1*	WP_385886284.1	WP_385894225.1	WP_385890983.1	WP_385885141.1
Bacteroidota	*Thalassobellus suaedae*	WP_415866307.1	WP_415866465.1	WP_415861774.1	WP_415866469.1
Bacteroidota	*Thermaurantimonas aggregans*	WP_124396926.1	WP_124396826.1	WP_124397494.1	WP_160160588.1
Bacteroidota	*Thermaurantimonas sp.*	WP_409769666.1	WP_409769462.1	WP_409771203.1	WP_409771132.1
Bacteroidota	*Thermonema lapsum*	WP_166921076.1	WP_166919763.1	WP_166920515.1	WP_166918106.1
Bacteroidota	*Thermonema rossianum*	WP_038032522.1	WP_051632972.1	WP_038030079.1	WP_084147099.1
Bacteroidota	*Thermonema sp.*	WP_288006170.1	WP_288005729.1	WP_288007082.1	WP_288004089.1
Bacteroidota	*Ulvibacter antarcticus*	WP_121906617.1	WP_121906710.1	WP_121907624.1	WP_121907465.1
Bacteroidota	*Ulvibacter litoralis*	WP_093143850.1	WP_093143547.1	WP_093144987.1	WP_093145223.1
Bacteroidota	*Ulvibacter sp. MAR_2010_11*	WP_100804002.1	WP_100803909.1	WP_100804196.1	WP_100803434.1
Bacteroidota	*Urechidicola croceus*	WP_070235653.1	WP_070235832.1	WP_070235596.1	WP_070236178.1
Bacteroidota	*Urechidicola vernalis*	WP_311592092.1	WP_311591836.1	WP_311592154.1	WP_311594012.1
Bacteroidota	*Vaginella massiliensis*	WP_395092052.1	WP_068598256.1	WP_395093015.1	WP_395090947.1
Bacteroidota	*Vicingus serpentipes*	WP_147099218.1	WP_147101768.1	WP_147097973.1	WP_147100110.1
Bacteroidota	*Wandonia haliotis*	WP_343786300.1	WP_343788488.1	WP_343787892.1	WP_343785738.1
Bacteroidota	*Wenyingzhuangia aestuarii*	WP_167898478.1	WP_167898441.1	WP_167897819.1	WP_167896793.1
Bacteroidota	*Wenyingzhuangia fucanilytica*	WP_068825163.1	WP_068826438.1	WP_068825555.1	WP_068826364.1
Bacteroidota	*Wenyingzhuangia gilva*	WP_302884102.1	WP_302883866.1	WP_302885059.1	WP_302883913.1

Bacteroidota	*Wenyingzhuangia heitensis*	WP_167186003.1	WP_167185859.1	WP_167187886.1	WP_167187308.1
Bacteroidota	*Wenyingzhuangia marina*	WP_073120729.1	WP_073119375.1	WP_073118897.1	WP_073119261.1
Bacteroidota	*Wenyingzhuangia sp.*	WP_347182171.1	WP_347180341.1	WP_347180434.1	WP_347180213.1
Bacteroidota	*Winogradskyella alexanderae*	WP_224526353.1	WP_224530615.1	WP_224526489.1	WP_224526926.1
Bacteroidota	*Winogradskyella algicola*	WP_138434410.1	WP_138433396.1	WP_138434160.1	WP_138434612.1
Bacteroidota	*Winogradskyella aquimaris*	WP_320555137.1	WP_320556630.1	WP_320556356.1	WP_320555584.1
Bacteroidota	*Winogradskyella arenosi*	WP_114309688.1	WP_114309489.1	WP_114309772.1	WP_114310057.1
Bacteroidota	*Winogradskyella aurantia*	WP_094967441.1	WP_094968397.1	WP_094967377.1	WP_094968831.1
Bacteroidota	*Winogradskyella aurantiaca*	WP_115463024.1	WP_115462847.1	WP_115463094.1	WP_317047361.1
Bacteroidota	*Winogradskyella bathintestinalis*	WP_290206016.1	WP_290206804.1	WP_290206994.1	WP_290206180.1
Bacteroidota	*Winogradskyella costae*	WP_179336134.1	WP_179335048.1	WP_179334623.1	WP_179335300.1
Bacteroidota	*Winogradskyella echinorum*	WP_186846218.1	WP_186845557.1	WP_186845790.1	WP_186845877.1
Bacteroidota	*Winogradskyella eckloniae*	WP_173281221.1	WP_173281061.1	WP_173280570.1	WP_173281378.1
Bacteroidota	*Winogradskyella endarachnes*	WP_157362260.1	WP_157361762.1	WP_157363281.1	WP_157362488.1
Bacteroidota	*Winogradskyella epiphytica*	WP_110475366.1	WP_110475170.1	WP_110476132.1	WP_110476237.1
Bacteroidota	*Winogradskyella eximia*	WP_115818299.1	WP_115817543.1	WP_115818051.1	WP_115817452.1
Bacteroidota	*Winogradskyella flava*	WP_282043432.1	WP_185788902.1	WP_282043289.1	WP_282043124.1
Bacteroidota	*Winogradskyella forsetii*	WP_179008373.1	WP_179020256.1	WP_179006261.1	WP_179018484.1
Bacteroidota	*Winogradskyella haliclonae*	WP_188374535.1	WP_188374348.1	WP_188374596.1	WP_188375109.1
Bacteroidota	*Winogradskyella helgolandensis*	WP_179319568.1	WP_178985666.1	WP_317167555.1	WP_179319418.1
Bacteroidota	*Winogradskyella immobilis*	WP_227477884.1	WP_227477848.1	WP_227476698.1	WP_227477668.1
Bacteroidota	*Winogradskyella jejuensis*	WP_073087005.1	WP_073086886.1	WP_073087204.1	WP_073085722.1
Bacteroidota	*Winogradskyella litorisediminis*	WP_386127345.1	WP_386127183.1	WP_386132737.1	WP_386132081.1
Bacteroidota	*Winogradskyella litoriviva*	WP_173300366.1	WP_173300105.1	WP_173301905.1	WP_173300484.1
Bacteroidota	*Winogradskyella ludwigii*	WP_179337864.1	WP_179338973.1	WP_179339464.1	WP_179338155.1
Bacteroidota	*Winogradskyella luteola*	WP_218544163.1	WP_218544848.1	WP_218544234.1	WP_218544490.1
Bacteroidota	*Winogradskyella maritima*	WP_386095730.1	WP_386101441.1	WP_386095869.1	WP_386099159.1
Bacteroidota	*Winogradskyella pacifica*	WP_115808434.1	WP_115808652.1	WP_115809554.1	WP_179349795.1

Bacteroidota	*Winogradskyella pelagia*	WP_208153639.1	WP_208154871.1	WP_208153767.1	WP_208154100.1
Bacteroidota	*Winogradskyella poriferorum*	WP_331810318.1	WP_331809773.1	WP_331810245.1	WP_331809409.1
Bacteroidota	*Winogradskyella psychrotolerans*	WP_020896912.1	WP_215925849.1	WP_215936346.1	WP_020896739.1
Bacteroidota	*Winogradskyella pulchriflava*	WP_386063557.1	WP_386061894.1	WP_386061462.1	WP_386064116.1
Bacteroidota	*Winogradskyella rapida*	WP_386115939.1	WP_386114648.1	WP_386113114.1	WP_386117398.1
Bacteroidota	*Winogradskyella schleiferi*	WP_178988959.1	WP_178988830.1	WP_178988226.1	WP_178987854.1
Bacteroidota	*Winogradskyella sediminis*	WP_115839258.1	WP_092443319.1	WP_417876348.1	WP_115839130.1
Bacteroidota	*Winogradskyella sp.*	WP_296384836.1	WP_296352858.1	WP_391593001.1	WP_370103802.1
Bacteroidota	*Winogradskyella tangerina*	WP_111684169.1	WP_111682739.1	WP_111684095.1	WP_111683804.1
Bacteroidota	*Winogradskyella thalassocola*	WP_092468787.1	WP_092469884.1	WP_092467141.1	WP_092469187.1
Bacteroidota	*Winogradskyella undariae*	WP_173588526.1	WP_179315410.1	WP_179317035.1	WP_173588138.1
Bacteroidota	*Winogradskyella ursingii*	WP_179346562.1	WP_179344862.1	WP_179346499.1	WP_179346073.1
Bacteroidota	*Winogradskyella vidalii*	WP_179352015.1	WP_179353704.1	WP_179352429.1	WP_179351812.1
Bacteroidota	*Winogradskyella vincentii*	WP_224478894.1	WP_224478105.1	WP_224479179.1	WP_224478740.1
Bacteroidota	*Winogradskyella wandonensis*	WP_132705735.1	WP_132703720.1	WP_132705881.1	WP_132702815.1
Bacteroidota	*Winogradskyella wichelsiae*	WP_179376080.1	WP_179376959.1	WP_179376871.1	WP_179376516.1
Bacteroidota	*Wocania arenilitoris*	WP_237240727.1	WP_237240459.1	WP_237239480.1	WP_237240506.1
Bacteroidota	*Wocania ichthyoenteri*	WP_034044808.1	WP_034044660.1	WP_034040352.1	WP_034041167.1
Bacteroidota	*Xanthocytophaga flavus*	WP_314000089.1	WP_313978959.1	WP_313986097.1	WP_313978846.1
Bacteroidota	*Xanthomarina gelatinilytica*	WP_417873413.1	WP_417857232.1	WP_370100095.1	WP_417882250.1
Bacteroidota	*Xanthomarina sp.*	WP_324312250.1	WP_324313051.1	WP_286853545.1	WP_286854418.1
Bacteroidota	*Xanthomarina spongicola*	WP_109683326.1	WP_109681702.1	WP_109681507.1	WP_109682487.1
Bacteroidota	*Yeosuana aromativorans*	WP_188654342.1	WP_188653250.1	WP_188654997.1	WP_188652436.1
Bacteroidota	*Yeosuana marina*	WP_166961327.1	WP_166965288.1	WP_166963373.1	WP_166965638.1
Balneolota	*Aliifodinibius salipaludis*	WP_095607415.1	WP_095605785.1	WP_095607636.1	WP_255233382.1
Balneolota	*Aliifodinibius sp. S!AR15-10*	WP_310686561.1	WP_310684926.1	WP_310682093.1	WP_310685409.1
Balneolota	*Balneola sp. EhC07*	WP_066223311.1	WP_066222314.1	WP_066220100.1	WP_066217088.1
Balneolota	*Cyclonatronum proteinivorum*	WP_164682415.1	WP_114985548.1	WP_114983795.1	WP_164682663.1

Balneolota	*Cyclonatronum sp.*	WP_291482411.1	WP_291482712.1	WP_291482818.1	WP_291482004.1
Balneolota	*Fodinibius roseus*	WP_073063831.1	WP_073061524.1	WP_073063141.1	WP_073059135.1
Balneolota	*Fodinibius salicampi*	WP_265791860.1	WP_265790485.1	WP_265788309.1	WP_265787781.1
Balneolota	*Fodinibius salinus*	WP_148899828.1	WP_148898505.1	WP_246138157.1	WP_170245550.1
Balneolota	*Fodinibius saliphilus*	WP_138432167.1	WP_138430825.1	WP_138430056.1	WP_138429484.1
Balneolota	*Fodinibius salsisoli*	WP_265767352.1	WP_265764074.1	WP_265766003.1	WP_265765528.1
Balneolota	*Fodinibius sediminis*	WP_142714531.1	WP_142712524.1	WP_142714645.1	WP_185958215.1
Balneolota	*Fodinibius sp.*	WP_372634043.1	WP_322574622.1	WP_322575165.1	WP_322575107.1
Balneolota	*Halalkalibaculum roseum*	WP_165143800.1	WP_165138370.1	WP_165140079.1	WP_165140357.1
Balneolota	*Halalkalibaculum sp. DA3122*	WP_395257904.1	WP_395259678.1	WP_395259273.1	WP_395261429.1
Balneolota	*Natronogracilivirga saccharolytica*	WP_210509762.1	WP_210513032.1	WP_210509515.1	WP_210511334.1
Balneolota	*Rhodohalobacter barkolensis*	WP_101073171.1	WP_101073066.1	WP_101071599.1	WP_101071812.1
Balneolota	*Rhodohalobacter mucosus*	WP_109647948.1	WP_109647779.1	WP_109646781.1	WP_109645422.1
Balneolota	*Rhodohalobacter sp.*	WP_372902497.1	WP_322570300.1	WP_372905583.1	WP_372904606.1
Balneolota	*Rhodohalobacter sulfatireducens*	WP_237852421.1	WP_237852468.1	WP_237853641.1	WP_237855134.1
Calditrichaeota	*Caldithrix abyssi*	WP_006931011.1	WP_006929106.1	WP_044281486.1	WP_006931009.1
Chlamydiota	*Criblamydia sequanensis*	WP_041017343.1	WP_041016448.1	WP_053331647.1	WP_041017369.1
Chlamydiota	*Estrella lausannensis*	WP_098038228.1	WP_239414312.1	WP_098037590.1	WP_098038239.1
Chlamydiota	*Waddlia chondrophila*	WP_013182233.1	WP_041941457.1	WP_013181378.1	WP_013181809.1
Chlorobiota	*Candidatus Chlorobium masyuteum*	WP_166808611.1	WP_166808429.1	WP_166808443.1	WP_166807795.1
Chlorobiota	*Chlorobaculum limnaeum*	WP_069809554.1	WP_069810356.1	WP_069809037.1	WP_069808910.1
Chlorobiota	*Chlorobaculum parvum*	WP_012502214.1	WP_012502674.1	WP_012501996.1	WP_012501656.1
Chlorobiota	*Chlorobaculum sp. MV4-Y*	WP_260633512.1	WP_260533492.1	WP_260633235.1	WP_260533288.1
Chlorobiota	*Chlorobaculum tepidum*	WP_010932718.1	WP_164926877.1	WP_010933086.1	WP_010933222.1
Chlorobiota	*Chlorobaculum thiosulfatiphilum*	WP_139457526.1	WP_139456871.1	WP_139457025.1	WP_139457127.1
Chlorobiota	*Chlorobium ferrooxidans*	WP_006366651.1	WP_006366427.1	WP_006367401.1	WP_006365343.1
Chlorobiota	*Chlorobium limicola*	WP_012466208.1	WP_041465847.1	WP_012465860.1	WP_276672508.1
Chlorobiota	*Chlorobium phaeobacteroides*	WP_011745192.1	WP_011744678.1	WP_011744703.1	WP_011745851.1

Chlorobiota	*Chlorobium phaeovibrioides*	WP_151419445.1	WP_126384076.1	WP_126342229.1	WP_126384756.1
Chlorobiota	*Chlorobium sp.*	WP_423931470.1	WP_331561158.1	WP_292002077.1	WP_331561788.1
Chlorobiota	*Chloroherpeton thalassium*	WP_012500949.1	WP_012499088.1	WP_012500566.1	WP_012499687.1
Chlorobiota	*Pelodictyon luteolum*	WP_011357747.1	WP_303680767.1	WP_011358150.1	WP_303682490.1
Chlorobiota	*Pelodictyon phaeoclathratiforme*	WP_012508109.1	WP_041526422.1	WP_012508493.1	WP_012509052.1
Chlorobiota	*Prosthecochloris sp.*	WP_294347425.1	WP_288031379.1	WP_294346988.1	WP_287220752.1
Deinococcota	*Thermus sp. PS18*	WP_219760243.1	WP_267260854.1	WP_267260744.1	WP_267261639.1
Gemmatimonadota	*Candidatus Palauibacter irciniicola*	WP_419858893.1	WP_419859026.1	WP_419856829.1	WP_419858894.1
Gemmatimonadota	*Candidatus Palauibacter polyketidifaciens*	WP_310785101.1	WP_310783687.1	WP_310785236.1	WP_310785103.1
Gemmatimonadota	*Candidatus Palauibacter scopulicola*	WP_310778325.1	WP_310778401.1	WP_310775338.1	WP_310778328.1
Gemmatimonadota	*Candidatus Palauibacter soopunensis*	WP_310759046.1	WP_310758907.1	WP_310757617.1	WP_310759045.1
Gemmatimonadota	*Gemmatimonas sp.*	WP_331050747.1	WP_396219785.1	WP_409947910.1	WP_396206035.1
Gemmatimonadota	*Longimicrobium sp.*	WP_329686406.1	WP_331128124.1	WP_331876780.1	WP_422655644.1
Gemmatimonadota	*Longimicrobium terrae*	WP_170036276.1	WP_170038756.1	WP_170035611.1	WP_170036976.1
Gemmatimonadota	*Pseudogemmatithrix spongiicola*	WP_367885176.1	WP_367887761.1	WP_367887364.1	WP_367887824.1
Ignavibacteriota	*Ignavibacterium album*	WP_014560944.1	WP_014561555.1	WP_014560830.1	WP_014560661.1
Ignavibacteriota	*Ignavibacterium sp.*	WP_297843333.1	WP_337866183.1	WP_337872606.1	WP_297842622.1
Myxococcota	*Stigmatella aurantiaca*	WP_002618112.1	WP_013374927.1	WP_075007943.1	WP_075007944.1
Myxococcota	*Vulgatibacter sp.*	WP_373046082.1	WP_373045413.1	WP_373045414.1	WP_373045415.1
Planctomycetota	*Engelhardtia mirabilis*	WP_145069642.1	WP_145070674.1	WP_419191964.1	WP_419191963.1
Planctomycetota	*Saltatorellus ferox*	WP_419191093.1	WP_145205850.1	WP_419190757.1	WP_145205827.1
Rhodothermota	*Longibacter salinarum*	WP_098076119.1	WP_098074559.1	WP_098074888.1	WP_098074867.1
Rhodothermota	*Longimonas halophila*	WP_098061634.1	WP_098061945.1	WP_098063067.1	WP_098060759.1
Rhodothermota	*Longimonas sp.*	WP_353562514.1	WP_410338332.1	WP_410338071.1	WP_353562900.1
Rhodothermota	*Rhodocaloribacter litoris*	WP_166978522.1	WP_166974234.1	WP_228350645.1	WP_166978060.1
Rhodothermota	*Rhodothermus bifroesti*	WP_210376068.1	WP_210373907.1	WP_210375124.1	WP_210375121.1
Rhodothermota	*Rhodothermus marinus*	WP_161540542.1	WP_012842763.1	WP_012843182.1	WP_014067708.1

Rhodothermota	*Rhodothermus profundi*	WP_072714519.1	WP_072715349.1	WP_072715210.1	WP_072715214.1
Rhodothermota	*Rubrivirga sp.*	WP_420456952.1	WP_420455377.1	WP_424521619.1	WP_420454540.1
Rhodothermota	*Salinibacter altiplanensis*	WP_103021536.1	WP_103019979.1	WP_103027427.1	WP_103029837.1
Rhodothermota	*Salinibacter grassmerensis*	WP_263787278.1	WP_263786151.1	WP_263785401.1	WP_263786104.1
Rhodothermota	*Salinibacter ruber*	WP_251931083.1	WP_341481556.1	WP_112903583.1	WP_259085182.1
Rhodothermota	Salinibacter sp.	WP_375802491.1	WP_375802762.1	WP_263834908.1	WP_375801842.1
Rhodothermota	Salisaeta longa	WP_022835959.1	WP_022835967.1	WP_022836238.1	WP_028567198.1

Supplementary Dataset 1B – Unique species containing the complete BSH biosynthetic pathway. Phylum, species, and NCBI accession codes for BshA, BshB, and BshC are listed for each organism.

Phylum	Organism	Accession code BshA	Accession code BshB	Accession code BshC
Acidobacteriota	*Acidicapsa acidisoli*	WP_263355290.1	WP_263352547.1	WP_263353948.1
Acidobacteriota	*Acidicapsa dinghuensis*	WP_263332892.1	WP_263339261.1	WP_263342256.1
Acidobacteriota	*Acidicapsa ligni*	WP_263357988.1	WP_317890557.1	WP_263356903.1
Acidobacteriota	*Acidipila rosea*	WP_131991325.1	WP_131994826.1	WP_131994166.1
Acidobacteriota	*Acidisarcina polymorpha*	WP_114206173.1	WP_338026767.1	WP_114205520.1
Acidobacteriota	*Acidobacterium sp. S8*	WP_158748945.1	WP_158749030.1	WP_158751191.1
Acidobacteriota	*Alloacidobacterium dinghuense*	WP_186747285.1	WP_186745795.1	WP_186744667.1
Acidobacteriota	*Alloacidobacterium sp.*	WP_324830802.1	WP_324832157.1	WP_324831241.1
Acidobacteriota	*Bryocella elongata*	WP_103931846.1	WP_103931120.1	WP_103933629.1
Acidobacteriota	*Candidatus Korobacter versatilis*	WP_041857031.1	WP_011524233.1	WP_011522983.1
Acidobacteriota	*Chloracidobacterium aggregatum*	WP_211423704.1	WP_211423485.1	WP_211426094.1
Acidobacteriota	*Chloracidobacterium sp. D*	WP_211432787.1	WP_211433022.1	WP_211432012.1
Acidobacteriota	*Chloracidobacterium thermophilum*	WP_058866111.1	WP_058865916.1	WP_014098599.1
Acidobacteriota	*Chloracidobacterium validum*	WP_211428433.1	WP_211430360.1	WP_211428837.1
Acidobacteriota	*Edaphobacter aggregans*	WP_035355527.1	WP_125484521.1	WP_125484223.1
Acidobacteriota	*Edaphobacter bradus*	WP_263366346.1	WP_263365139.1	WP_263368437.1

Acidobacteriota	*Edaphobacter dinghuensis*	WP_188552996.1	WP_188553926.1	WP_188554963.1
Acidobacteriota	*Edaphobacter flagellatus*	WP_260705587.1	WP_260706006.1	WP_260704451.1
Acidobacteriota	*Edaphobacter modestus*	WP_130418350.1	WP_130421490.1	WP_130418550.1
Acidobacteriota	*Edaphobacter paludis*	WP_348268774.1	WP_348266716.1	WP_348269426.1
Acidobacteriota	*Edaphobacter sp.*	WP_325386905.1	WP_299377192.1	WP_326445757.1
Acidobacteriota	*Granulicella aggregans*	WP_184213714.1	WP_184217056.1	WP_184217029.1
Acidobacteriota	*Granulicella arctica*	WP_179492651.1	WP_179490700.1	WP_263381590.1
Acidobacteriota	*Granulicella cerasi*	WP_390236537.1	WP_263370744.1	WP_263371471.1
Acidobacteriota	*Granulicella mallensis*	WP_014264654.1	WP_184252288.1	WP_184255776.1
Acidobacteriota	*Granulicella paludicola*	WP_263378267.1	WP_263379900.1	WP_263379336.1
Acidobacteriota	*Granulicella pectinivorans*	WP_089840539.1	WP_089838949.1	WP_089840336.1
Acidobacteriota	*Granulicella rosea*	WP_089407803.1	WP_089408523.1	WP_089407018.1
Acidobacteriota	*Granulicella sibirica*	WP_128913431.1	WP_128911222.1	WP_128913874.1
Acidobacteriota	*Granulicella sp. 5B5*	WP_182276896.1	WP_182277462.1	WP_182276653.1
Acidobacteriota	*Granulicella tundricola*	WP_013579839.1	WP_013580942.1	WP_013580988.1
Acidobacteriota	*Luteitalea pratensis*	WP_110169869.1	WP_110169868.1	WP_157899524.1
Acidobacteriota	*Occallatibacter riparius*	WP_260791635.1	WP_260792614.1	WP_260793218.1
Acidobacteriota	*Occallatibacter savannae*	WP_109484365.1	WP_204101321.1	WP_109487914.1
Acidobacteriota	*Paludibaculum fermentans*	WP_194446804.1	WP_194448789.1	WP_194448933.1
Acidobacteriota	*Paracidobacterium acidisoli*	WP_117298274.1	WP_117297597.1	WP_117297892.1
Acidobacteriota	*Pseudacidobacterium ailaaui*	WP_044933726.1	WP_026443242.1	WP_026443683.1
Acidobacteriota	*Pyrinomonas methylaliphatogenes*	WP_041975312.1	WP_041975311.1	WP_157770925.1
Acidobacteriota	*Pyrinomonas sp.*	WP_352430502.1	WP_352430500.1	WP_352432353.1
Acidobacteriota	*Silvibacterium bohemicum*	WP_050061700.1	WP_231581164.1	WP_050057645.1
Acidobacteriota	*Silvibacterium dinghuense*	WP_129208410.1	WP_129208146.1	WP_129209111.1
Acidobacteriota	*Silvibacterium sp.*	WP_415197828.1	WP_415197928.1	WP_415199972.1
Acidobacteriota	*Telmatobacter bradus*	WP_420236317.1	WP_420239077.1	WP_420236529.1
Acidobacteriota	*Telmatobacter sp. DSM 110680*	WP_348261856.1	WP_348261883.1	WP_348262743.1

Acidobacteriota	*Terracidiphilus gabretensis*	WP_058189573.1	WP_231738106.1	WP_231738037.1
Acidobacteriota	*Terracidiphilus sp.*	WP_424170589.1	WP_424165284.1	WP_424166473.1
Acidobacteriota	*Thermoanaerobaculum aquaticum*	WP_038046342.1	WP_053334713.1	WP_038046340.1
Acidobacteriota	*Tunturiibacter empetritectus*	WP_183760154.1	WP_179639254.1	WP_353069306.1
Acidobacteriota	*Tunturiibacter gelidoferens*	WP_353073468.1	WP_183980995.1	WP_183976422.1
Acidobacteriota	*Tunturiibacter lichenicola*	WP_260737915.1	WP_260734640.1	WP_260738339.1
Acidobacteriota	*Tunturiibacter psychrotolerans*	WP_429633432.1	WP_353063468.1	WP_429629698.1
Bacillota	*Abyssicoccus albus*	WP_123807515.1	WP_123808477.1	WP_077140460.1
Bacillota	*Acetohalobium arabaticum*	WP_013277297.1	WP_013277296.1	WP_013277295.1
Bacillota	*Acidaminobacter sp.*	WP_291400587.1	WP_291404499.1	WP_300687168.1
Bacillota	*Aciduricibacillus chroicocephali*	WP_348029743.1	WP_348026512.1	WP_348029467.1
Bacillota	*Alicyclobacillus acidiphilus*	WP_067618599.1	WP_067618720.1	WP_067620725.1
Bacillota	*Alicyclobacillus acidocaldarius*	WP_012810972.1	WP_012811345.1	WP_012810636.1
Bacillota	*Alicyclobacillus acidoterrestris*	WP_021296764.1	WP_021297575.1	WP_021297415.1
Bacillota	*Alicyclobacillus cellulosilyticus*	WP_188882258.1	WP_188883153.1	WP_188880771.1
Bacillota	*Alicyclobacillus contaminans*	WP_026974667.1	WP_026975382.1	WP_156900185.1
Bacillota	*Alicyclobacillus cycloheptanicus*	WP_274455965.1	WP_274454683.1	WP_274456251.1
Bacillota	*Alicyclobacillus dauci*	WP_268042068.1	WP_268043292.1	WP_268042825.1
Bacillota	*Alicyclobacillus fastidiosus*	WP_275476750.1	WP_275473714.1	WP_268004344.1
Bacillota	*Alicyclobacillus ferrooxydans*	WP_054968087.1	WP_054968204.1	WP_054967649.1
Bacillota	*Alicyclobacillus fodiniaquatilis*	WP_377942297.1	WP_377940631.1	WP_377945233.1
Bacillota	*Alicyclobacillus fructus*	WP_206830432.1	WP_206832021.1	WP_206831247.1
Bacillota	*Alicyclobacillus herbarius*	WP_026962492.1	WP_026961893.1	WP_026961655.1
Bacillota	*Alicyclobacillus hesperidum*	WP_281758772.1	WP_100218590.1	WP_058094035.1
Bacillota	*Alicyclobacillus kakegawensis*	WP_067935298.1	WP_067931723.1	WP_067931593.1
Bacillota	*Alicyclobacillus macrosporangiidus*	WP_303798311.1	WP_074948571.1	WP_074950252.1
Bacillota	*Alicyclobacillus mali (ex Roth et al. 2021)*	WP_195867399.1	WP_067847766.1	WP_067850001.1
Bacillota	*Alicyclobacillus mengziensis*	WP_206655069.1	WP_206658284.1	WP_206658773.1

Bacillota	*Alicyclobacillus pomorum*	WP_026965004.1	WP_035466845.1	WP_051375186.1
Bacillota	*Alicyclobacillus sacchari*	WP_134158628.1	WP_134159108.1	WP_134159532.1
Bacillota	*Alicyclobacillus sendaiensis*	WP_062305587.1	WP_283202282.1	WP_062307820.1
Bacillota	*Alicyclobacillus shizuokensis*	WP_067929041.1	WP_067927753.1	WP_067922161.1
Bacillota	*Alicyclobacillus suci*	WP_146824268.1	WP_146822294.1	WP_236018018.1
Bacillota	*Alicyclobacillus tolerans*	WP_236811744.1	WP_236812631.1	WP_236813424.1
Bacillota	*Alicyclobacillus vulcanalis*	WP_076347670.1	WP_076346339.1	WP_076347978.1
Bacillota	*Aliibacillus thermotolerans*	WP_270897557.1	WP_270897558.1	WP_270898399.1
Bacillota	*Aliicoccus persicus*	WP_091473304.1	WP_091475302.1	WP_091472699.1
Bacillota	*Alkalibacillus aidingensis*	WP_188206003.1	WP_188206685.1	WP_188205716.1
Bacillota	*Alkalibacillus almallahensis*	WP_167262394.1	WP_167262627.1	WP_167259867.1
Bacillota	*Alkalibacillus filiformis*	WP_307066048.1	WP_307069212.1	WP_307065437.1
Bacillota	*Alkalibacillus flavidus*	WP_354218829.1	WP_354219555.1	WP_354218417.1
Bacillota	*Alkalibacillus haloalkaliphilus*	WP_146814819.1	WP_146817884.1	WP_317093364.1
Bacillota	*Alkalibacillus salilacus*	WP_306978150.1	WP_306976254.1	WP_306975461.1
Bacillota	*Alkalibacillus silvisoli*	WP_343782538.1	WP_343785100.1	WP_343782819.1
Bacillota	*Alkalibacillus sp. S2W*	WP_411953315.1	WP_411953871.1	WP_411955377.1
Bacillota	*Alkalicella caledoniensis*	WP_213168081.1	WP_213168082.1	WP_213168084.1
Bacillota	*Alkalicoccobacillus gibsonii*	WP_369875514.1	WP_343130382.1	WP_203087334.1
Bacillota	*Alkalicoccobacillus murimartini*	WP_306979994.1	WP_306979993.1	WP_306980876.1
Bacillota	*Alkalicoccobacillus plakortidis*	WP_251603919.1	WP_251603916.1	WP_251604935.1
Bacillota	*Alkalicoccobacillus porphyridii*	WP_143848168.1	WP_143848167.1	WP_143848764.1
Bacillota	*Alkalicoccus chagannorensis*	WP_026696511.1	WP_026696512.1	WP_026698190.1
Bacillota	*Alkalicoccus daliensis*	WP_090840435.1	WP_090840437.1	WP_090839767.1
Bacillota	*Alkalicoccus halolimnae*	WP_147804768.1	WP_147804769.1	WP_147802416.1
Bacillota	*Alkalicoccus luteus*	WP_168008843.1	WP_168008845.1	WP_168004737.1
Bacillota	*Alkalicoccus saliphilus*	WP_107582712.1	WP_107582714.1	WP_107583975.1
Bacillota	*Alkalicoccus urumqiensis*	WP_105957872.1	WP_105957871.1	WP_105959832.1

Bacillota	*Alkalihalobacillus alcalophilus*	WP_003322410.1	WP_003322411.1	WP_003324267.1
Bacillota	*Alkalihalobacillus algicola*	WP_224880004.1	WP_224880007.1	WP_224881858.1
Bacillota	*Alkalihalobacillus deserti*	WP_227936217.1	WP_227936216.1	WP_227935718.1
Bacillota	*Alkalihalobacillus hemicentroti*	WP_301550589.1	WP_301551104.1	WP_301552758.1
Bacillota	*Alkalihalobacillus macyae*	WP_048309264.1	WP_305911367.1	WP_305913697.1
Bacillota	*Alkalihalobacillus pseudalcaliphilus*	WP_047989292.1	WP_047989293.1	WP_047987769.1
Bacillota	*Alkalihalobacillus sp. AL-G*	WP_304977622.1	WP_304977624.1	WP_304980912.1
Bacillota	*Alkalihalobacillus trypoxylicola*	WP_045484026.1	WP_061948118.1	WP_061947378.1
Bacillota	*Alkalihalobacterium alkalinitrilicum*	WP_078428479.1	WP_078429650.1	WP_158211770.1
Bacillota	*Alkalihalobacterium bogoriense*	WP_035178327.1	WP_035178325.1	WP_026674384.1
Bacillota	*Alkalihalobacterium chitinilyticum*	WP_275116532.1	WP_275119241.1	WP_275117687.1
Bacillota	*Alkalihalobacterium elongatum*	WP_216829224.1	WP_216830119.1	WP_216830881.1
Bacillota	*Alkalihalobacterium sp. APHAB7*	WP_418199901.1	WP_418197894.1	WP_418198652.1
Bacillota	*Alkalihalophilus lindianensis*	WP_317120797.1	WP_317120796.1	WP_317122523.1
Bacillota	*Alkalihalophilus marmarensis*	WP_326238215.1	WP_022627179.1	WP_022628259.1
Bacillota	*Alkalihalophilus pseudofirmus*	WP_323465924.1	WP_012958526.1	WP_012959498.1
Bacillota	*Alkalihalophilus sp. As8PL*	WP_368504480.1	WP_368504481.1	WP_368503982.1
Bacillota	*Alteribacillus bidgolensis*	WP_091579714.1	WP_245917746.1	WP_091582817.1
Bacillota	*Alteribacillus iranensis*	WP_091656672.1	WP_091656669.1	WP_091657528.1
Bacillota	*Alteribacillus persepolensis*	WP_091270857.1	WP_245705149.1	WP_091270224.1
Bacillota	*Alteribacillus sp. HJP-4*	WP_375534816.1	WP_375534815.1	WP_375533795.1
Bacillota	*Alteribacter aurantiacus*	WP_026689511.1	WP_026689510.1	WP_026690006.1
Bacillota	*Alteribacter keqinensis*	WP_122896690.1	WP_122896689.1	WP_122897211.1
Bacillota	*Alteribacter lacisalsi*	WP_110518270.1	WP_110518271.1	WP_110517475.1
Bacillota	*Alteribacter natronophilus*	WP_138809359.1	WP_138809360.1	WP_138808907.1
Bacillota	*Alteribacter populi*	WP_096434897.1	WP_096434896.1	WP_157811962.1
Bacillota	*Alteribacter salitolerans*	WP_205165103.1	WP_205165104.1	WP_205166118.1
Bacillota	*Ammoniphilus oxalaticus*	WP_120188418.1	WP_120188417.1	WP_120189348.1

Bacillota	*Ammoniphilus resinae*	WP_209811430.1	WP_209811431.1	WP_209808725.1
Bacillota	*Ammoniphilus sp. CFH 90114*	WP_129198961.1	WP_129198958.1	WP_164984851.1
Bacillota	*Amphibacillus cookii*	WP_204439232.1	WP_204437696.1	WP_204435918.1
Bacillota	*Amphibacillus jilinensis*	WP_017472183.1	WP_017472445.1	WP_017471791.1
Bacillota	*Amphibacillus marinus*	WP_091493446.1	WP_091499335.1	WP_091494518.1
Bacillota	*Amphibacillus sediminis*	WP_067836485.1	WP_067840260.1	WP_067838861.1
Bacillota	*Anaerobacillus alkalilacustris*	WP_071309040.1	WP_071308954.1	WP_071309864.1
Bacillota	*Anaerobacillus alkaliphilus*	WP_129076319.1	WP_129076318.1	WP_129079471.1
Bacillota	*Anaerobacillus arseniciselenatis*	WP_071311749.1	WP_071311750.1	WP_071314187.1
Bacillota	*Anaerobacillus isosaccharinicus*	WP_071319369.1	WP_108721444.1	WP_182081065.1
Bacillota	*Anaerobacillus sp.*	WP_409852530.1	WP_409852635.1	WP_409849617.1
Bacillota	*Anaerobranca californiensis*	WP_072908573.1	WP_084672538.1	WP_072908575.1
Bacillota	*Anaerobranca gottschalkii*	WP_091349737.1	WP_091349735.1	WP_091349733.1
Bacillota	*Aneurinibacillus aneurinilyticus*	WP_276912594.1	WP_168974337.1	WP_021624582.1
Bacillota	*Aneurinibacillus migulanus*	WP_043067898.1	WP_235355982.1	WP_043066087.1
Bacillota	*Aneurinibacillus soli*	WP_096466561.1	WP_096466562.1	WP_096464848.1
Bacillota	*Aneurinibacillus sp. Ricciae_BoGa-3*	WP_272442497.1	WP_272442496.1	WP_272439498.1
Bacillota	*Aneurinibacillus terranovensis*	WP_027417519.1	WP_035102034.1	WP_027414484.1
Bacillota	*Aneurinibacillus tyrosinisolvens*	WP_047152598.1	WP_047152599.1	WP_052947533.1
Bacillota	*Aneurinibacillus uraniidurans*	WP_272560005.1	WP_272560006.1	WP_272562398.1
Bacillota	*Anoxybacillus ayderensis*	WP_085788008.1	WP_327867808.1	WP_181520507.1
Bacillota	*Anoxybacillus calidus*	WP_181535810.1	WP_181535808.1	WP_181536607.1
Bacillota	*Anoxybacillus flavithermus*	WP_006319484.1	WP_049754019.1	WP_003395102.1
Bacillota	*Anoxybacillus mongoliensis*	WP_183243126.1	WP_246346286.1	WP_183241713.1
Bacillota	*Anoxybacillus pushchinoensis*	WP_091702736.1	WP_091702733.1	WP_091703699.1
Bacillota	*Anoxybacillus sp.*	WP_297990735.1	WP_297990736.1	WP_297992505.1
Bacillota	*Anoxybacillus suryakundensis*	WP_032099862.1	WP_055440630.1	WP_055441894.1
Bacillota	*Anoxybacillus tengchongensis*	WP_183246662.1	WP_246348803.1	WP_183247171.1

Bacillota	*Anoxybacillus thermarum*	WP_043968235.1	WP_235341152.1	WP_043963574.1
Bacillota	*Anoxybacter fermentans*	WP_127015566.1	WP_205665684.1	WP_127016546.1
Bacillota	*Anoxybacteroides amylolyticum*	WP_066325763.1	WP_066325769.1	WP_066323566.1
Bacillota	*Anoxybacteroides rupiense*	WP_212387093.1	WP_183186419.1	WP_183186293.1
Bacillota	*Anoxybacteroides tepidamans*	WP_027408554.1	WP_183251100.1	WP_183252222.1
Bacillota	*Anoxybacteroides voinovskiense*	WP_183183362.1	WP_183183363.1	WP_183182980.1
Bacillota	*Aquibacillus albus*	WP_204497294.1	WP_204502172.1	WP_239584314.1
Bacillota	*Aquibacillus halophilus*	WP_153736072.1	WP_338079246.1	WP_153735819.1
Bacillota	*Aquibacillus kalidii*	WP_186577852.1	WP_186580881.1	WP_186577561.1
Bacillota	*Aquibacillus koreensis*	WP_259871040.1	WP_259870913.1	WP_259872153.1
Bacillota	*Aquibacillus rhizosphaerae*	WP_285931966.1	WP_285930127.1	WP_285932499.1
Bacillota	*Aquibacillus saliphilus*	WP_226034707.1	WP_226036032.1	WP_226037929.1
Bacillota	*Aquibacillus salsiterrae*	WP_272444767.1	WP_272445133.1	WP_272445492.1
Bacillota	*Aquibacillus sediminis*	WP_138417006.1	WP_138418335.1	WP_138415026.1
Bacillota	*Aquisalibacillus elongatus*	WP_124220368.1	WP_124221327.1	WP_124220187.1
Bacillota	*Aureibacillus halotolerans*	WP_133579378.1	WP_133579379.1	WP_133579192.1
Bacillota	*Bacillus aerius*	WP_309172889.1	WP_343489429.1	WP_343489326.1
Bacillota	*Bacillus aerolatus*	WP_191991728.1	WP_152149454.1	WP_152149950.1
Bacillota	*Bacillus albus*	WP_166703425.1	WP_130067555.1	WP_166701986.1
Bacillota	*Bacillus alkalicellulosilyticus*	WP_078553300.1	WP_078556248.1	WP_078554865.1
Bacillota	*Bacillus altitudinis*	WP_212047044.1	WP_377863049.1	WP_268449497.1
Bacillota	*Bacillus alveayuensis*	WP_044749095.1	WP_044748078.1	WP_044893864.1
Bacillota	*Bacillus amyloliquefaciens*	WP_013352659.1	WP_132105281.1	WP_416522088.1
Bacillota	*Bacillus andreraoultii*	WP_033828563.1	WP_033827693.1	WP_033829314.1
Bacillota	*Bacillus anthracis*	WP_160832241.1	WP_042511954.1	WP_420952557.1
Bacillota	*Bacillus aquiflavi*	WP_163239921.1	WP_163239918.1	WP_163242269.1
Bacillota	*Bacillus arachidis*	WP_208016830.1	WP_208016829.1	WP_286015891.1
Bacillota	*Bacillus atrophaeus*	WP_219946263.1	WP_268524735.1	WP_268512546.1

Bacillota	*Bacillus australimaris*	WP_413597544.1	WP_413597545.1	WP_060699163.1
Bacillota	*Bacillus badius*	WP_223661648.1	WP_063385664.1	WP_223663117.1
Bacillota	*Bacillus benzoevorans*	WP_184523980.1	WP_184525618.1	WP_184521970.1
Bacillota	*Bacillus bingmayongensis*	WP_017152494.1	WP_017152495.1	WP_221783100.1
Bacillota	*Bacillus cabrialesii*	WP_103746458.1	WP_103746457.1	WP_129505784.1
Bacillota	*Bacillus canaveralius*	WP_101575838.1	WP_180956474.1	WP_101576518.1
Bacillota	*Bacillus carboniphilus*	WP_343803993.1	WP_343804291.1	WP_343799035.1
Bacillota	*Bacillus cereus*	WP_098540396.1	WP_336451787.1	WP_098491221.1
Bacillota	*Bacillus changyiensis*	WP_270798899.1	WP_271339824.1	WP_270799347.1
Bacillota	*Bacillus chungangensis*	WP_307229127.1	WP_370875661.1	WP_307231862.1
Bacillota	*Bacillus cihuensis*	WP_028391531.1	WP_028391532.1	WP_028392954.1
Bacillota	*Bacillus clarus*	WP_042982873.1	WP_042982872.1	WP_042984148.1
Bacillota	*Bacillus coahuilensis*	WP_059282924.1	WP_059351084.1	WP_059350700.1
Bacillota	*Bacillus cytotoxicus*	WP_251226243.1	WP_251226244.1	WP_087095433.1
Bacillota	*Bacillus dakarensis*	WP_077213779.1	WP_077215132.1	WP_077211426.1
Bacillota	*Bacillus daqingensis*	WP_377908507.1	WP_377908508.1	WP_377908126.1
Bacillota	*Bacillus dicomae*	WP_140969083.1	WP_140969082.1	WP_140970586.1
Bacillota	*Bacillus ectoiniformans*	WP_204553449.1	WP_204553281.1	WP_204552057.1
Bacillota	*Bacillus enclensis*	WP_058297630.1	WP_336511350.1	WP_058298361.1
Bacillota	*Bacillus fonticola*	WP_170007451.1	WP_170007452.1	WP_170006931.1
Bacillota	*Bacillus fungorum*	WP_353767081.1	WP_098759501.1	WP_353768741.1
Bacillota	*Bacillus gaemokensis*	WP_033676929.1	WP_033676931.1	WP_033672194.1
Bacillota	*Bacillus glycinifermentans*	WP_057957431.1	WP_232517718.1	WP_048353108.1
Bacillota	*Bacillus haikouensis*	WP_172252472.1	WP_172252470.1	WP_172248231.1
Bacillota	*Bacillus halotolerans*	WP_105954959.1	WP_105954958.1	WP_101861185.1
Bacillota	*Bacillus haynesii*	WP_268344774.1	WP_411809870.1	WP_268307532.1
Bacillota	*Bacillus hominis*	WP_289358627.1	WP_002117713.1	WP_289360060.1
Bacillota	*Bacillus horti*	WP_307392687.1	WP_307392685.1	WP_307390445.1

Bacillota	*Bacillus inaquosorum*	WP_327810411.1	WP_019258742.1	WP_268279082.1
Bacillota	*Bacillus infantis*	WP_257991664.1	WP_426878124.1	WP_224904057.1
Bacillota	*Bacillus kandeliae*	WP_338749638.1	WP_338749640.1	WP_338753894.1
Bacillota	*Bacillus kexueae*	WP_243386618.1	WP_243386617.1	WP_243386721.1
Bacillota	*Bacillus licheniformis*	WP_145614123.1	WP_422110169.1	WP_420951217.1
Bacillota	*Bacillus litorisediminis*	WP_243296802.1	WP_313897641.1	WP_243298167.1
Bacillota	*Bacillus lumedeiriae*	WP_404313695.1	WP_404313696.1	WP_404314937.1
Bacillota	*Bacillus luti*	WP_156576229.1	WP_312502853.1	WP_427229069.1
Bacillota	*Bacillus manliponensis*	WP_034636717.1	WP_369900347.1	WP_034635795.1
Bacillota	*Bacillus marasmi*	WP_147533767.1	WP_147533768.1	WP_147531919.1
Bacillota	*Bacillus marinisedimentorum*	WP_070121118.1	WP_070121796.1	WP_169823916.1
Bacillota	*Bacillus massiliglaciei*	WP_110927635.1	WP_110927634.1	WP_110929323.1
Bacillota	*Bacillus massiliigorillae*	WP_042346443.1	WP_042346440.1	WP_042349036.1
Bacillota	*Bacillus massilionigeriensis*	WP_075982109.1	WP_075982110.1	WP_075981027.1
Bacillota	*Bacillus mediterraneensis*	WP_071459948.1	WP_071459949.1	WP_071459349.1
Bacillota	*Bacillus mesophilum*	WP_151573066.1	WP_151573671.1	WP_151571977.1
Bacillota	*Bacillus mesophilus*	WP_163178608.1	WP_163178610.1	WP_163176861.1
Bacillota	*Bacillus methanolicus*	WP_274854886.1	WP_004435240.1	WP_003348869.1
Bacillota	*Bacillus mobilis*	WP_426833822.1	WP_426833821.1	WP_427265026.1
Bacillota	*Bacillus mojavensis*	WP_326192655.1	WP_326334022.1	WP_326223470.1
Bacillota	*Bacillus mycoides*	WP_240380238.1	WP_256484637.1	WP_215551778.1
Bacillota	*Bacillus nakamurai*	WP_229976555.1	WP_229976556.1	WP_229977878.1
Bacillota	*Bacillus ndiopicus*	WP_042478014.1	WP_042470698.1	WP_042471675.1
Bacillota	*Bacillus niameyensis*	WP_062104823.1	WP_062104822.1	WP_062105168.1
Bacillota	*Bacillus nitratireducens*	WP_001237358.1	WP_000015677.1	WP_097809294.1
Bacillota	*Bacillus norwichensis*	WP_191811341.1	WP_191811340.1	WP_191809666.1
Bacillota	*Bacillus oleivorans*	WP_097157564.1	WP_097157563.1	WP_097159486.1
Bacillota	*Bacillus pacificus*	WP_208754974.1	WP_228920269.1	WP_173601325.1

Bacillota	*Bacillus pakistanensis*	WP_205168494.1	WP_205168495.1	WP_205173913.1
Bacillota	*Bacillus paralicheniformis*	WP_143260978.1	WP_020451935.1	WP_205802241.1
Bacillota	*Bacillus paramycoides*	WP_098472977.1	WP_328064577.1	WP_342715544.1
Bacillota	*Bacillus paranthracis*	WP_412838602.1	WP_229136520.1	WP_270363186.1
Bacillota	*Bacillus pinisoli*	WP_246942140.1	WP_246942143.1	WP_246940210.1
Bacillota	*Bacillus piscicola*	WP_240375564.1	WP_338021128.1	WP_240375160.1
Bacillota	*Bacillus proteolyticus*	WP_071743869.1	WP_340506901.1	WP_327817159.1
Bacillota	*Bacillus pseudomycoides*	WP_098644666.1	WP_098165788.1	WP_243523993.1
Bacillota	*Bacillus pumilus*	WP_226509125.1	WP_224925693.1	WP_409481788.1
Bacillota	*Bacillus rubiinfantis*	WP_042355288.1	WP_042355287.1	WP_042356345.1
Bacillota	*Bacillus rugosus*	WP_166851399.1	WP_166851395.1	WP_326220590.1
Bacillota	*Bacillus safensis*	WP_212043074.1	WP_224426689.1	WP_377970795.1
Bacillota	*Bacillus salacetis*	WP_119547185.1	WP_421379192.1	WP_421384454.1
Bacillota	*Bacillus salipaludis*	WP_406581955.1	WP_133335929.1	WP_406580921.1
Bacillota	*Bacillus salitolerans*	WP_377929730.1	WP_377929731.1	WP_377926817.1
Bacillota	*Bacillus seohaeanensis*	WP_377932177.1	WP_377932179.1	WP_377937664.1
Bacillota	*Bacillus shivajii*	WP_226517787.1	WP_226518265.1	WP_226514851.1
Bacillota	*Bacillus siamensis*	WP_415273134.1	WP_326352018.1	WP_269775133.1
Bacillota	*Bacillus sinesaloumensis*	WP_077621011.1	WP_077618088.1	WP_077617725.1
Bacillota	*Bacillus solimangrovi*	WP_069716330.1	WP_083249078.1	WP_069716086.1
Bacillota	*Bacillus solitudinis*	WP_100407236.1	WP_100408361.1	WP_100404574.1
Bacillota	*Bacillus songklensis*	WP_377915469.1	WP_377915472.1	WP_377912371.1
Bacillota	*Bacillus sonorensis*	WP_326187081.1	WP_276561419.1	WP_077736879.1
Bacillota	*Bacillus sp. 03113*	WP_141433313.1	WP_141433314.1	WP_141431292.1
Bacillota	*Bacillus spizizenii*	WP_014114192.1	WP_019714760.1	WP_268470174.1
Bacillota	*Bacillus spongiae*	WP_336586651.1	WP_336586785.1	WP_336585491.1
Bacillota	*Bacillus stercoris*	WP_071580476.1	WP_136654146.1	WP_326274014.1
Bacillota	*Bacillus suaedae*	WP_210596305.1	WP_210596304.1	WP_210597829.1

Bacillota	*Bacillus suaedaesalsae*	WP_204205316.1	WP_204205317.1	WP_204203888.1
Bacillota	*Bacillus subtilis*	WP_041336364.1	WP_336805235.1	WP_032725648.1
Bacillota	*Bacillus swezeyi*	WP_414555380.1	WP_239693662.1	WP_148955705.1
Bacillota	*Bacillus taeanensis*	WP_113805161.1	WP_113805162.1	WP_181833047.1
Bacillota	*Bacillus tequilensis*	WP_174227222.1	WP_167872758.1	WP_174228065.1
Bacillota	*Bacillus testis*	WP_050615402.1	WP_050615403.1	WP_050614681.1
Bacillota	*Bacillus thermotolerans*	WP_175286644.1	WP_040037628.1	WP_039231947.1
Bacillota	*Bacillus thuringiensis*	WP_263701529.1	WP_131934799.1	WP_197230479.1
Bacillota	*Bacillus tianshenii*	WP_204414591.1	WP_224840203.1	WP_224840737.1
Bacillota	*Bacillus timonensis*	WP_136377963.1	WP_010281490.1	WP_136378255.1
Bacillota	*Bacillus toyonensis*	WP_098637734.1	WP_390190602.1	WP_098837687.1
Bacillota	*Bacillus tropicus*	WP_340031223.1	WP_337679143.1	WP_227571775.1
Bacillota	*Bacillus tuaregi*	WP_071395570.1	WP_071396756.1	WP_071395437.1
Bacillota	*Bacillus vallismortis*	WP_252685135.1	WP_394716094.1	WP_367387382.1
Bacillota	*Bacillus velezensis*	WP_326155360.1	WP_138092526.1	WP_041481861.1
Bacillota	*Bacillus weihaiensis*	WP_273125988.1	WP_273125986.1	WP_273125371.1
Bacillota	*Bacillus wiedmannii*	WP_098047379.1	WP_262745050.1	WP_098707497.1
Bacillota	*Bacillus wudalianchiensis*	WP_065409237.1	WP_065409236.1	WP_065411549.1
Bacillota	*Bacillus xiapuensis*	WP_198508479.1	WP_100332054.1	WP_100330817.1
Bacillota	*Bacillus yunxiaonensis*	WP_336482823.1	WP_336482822.1	WP_336483199.1
Bacillota	*Baia soyae*	WP_131848545.1	WP_131848544.1	WP_131847547.1
Bacillota	*Bhargavaea beijingensis*	WP_092095105.1	WP_092097408.1	WP_264556923.1
Bacillota	*Bhargavaea cecembensis*	WP_063179153.1	WP_040227486.1	WP_197462205.1
Bacillota	*Bhargavaea ginsengi*	WP_251239798.1	WP_251238947.1	WP_177168219.1
Bacillota	*Bhargavaea massiliensis*	WP_213423159.1	WP_213422174.1	WP_213423353.1
Bacillota	*Bhargavaea ullalensis*	WP_354196044.1	WP_354197139.1	WP_354197405.1
Bacillota	Brevibacillus agri	WP_327944617.1	WP_197187672.1	WP_005833010.1
Bacillota	Brevibacillus antibioticus	WP_137029308.1	WP_137029309.1	WP_137028103.1

Bacillota	*Brevibacillus borstelensis*	WP_171506301.1	WP_311067562.1	WP_327925907.1
Bacillota	*Brevibacillus brevis*	WP_048032664.1	WP_289677705.1	WP_310764353.1
Bacillota	*Brevibacillus centrosporus*	WP_400582021.1	WP_122961388.1	WP_327930787.1
Bacillota	*Brevibacillus choshinensis*	WP_055743623.1	WP_055743622.1	WP_203255281.1
Bacillota	*Brevibacillus composti*	WP_198826360.1	WP_198826361.1	WP_198829395.1
Bacillota	*Brevibacillus daliensis*	WP_232696377.1	WP_232696376.1	WP_232698373.1
Bacillota	*Brevibacillus dissolubilis*	WP_139489136.1	WP_139489137.1	WP_139489967.1
Bacillota	*Brevibacillus fluminis*	WP_122920747.1	WP_122920746.1	WP_122916979.1
Bacillota	*Brevibacillus formosus*	WP_197245195.1	WP_047069015.1	WP_197244069.1
Bacillota	*Brevibacillus fortis*	WP_327950004.1	WP_409178656.1	WP_409174669.1
Bacillota	*Brevibacillus fulvus*	WP_204516473.1	WP_204516472.1	WP_204517035.1
Bacillota	*Brevibacillus gelatini*	WP_122904259.1	WP_122904258.1	WP_122906096.1
Bacillota	*Brevibacillus ginsengisoli*	WP_426927693.1	WP_426927692.1	WP_426928466.1
Bacillota	*Brevibacillus humidisoli*	WP_230046444.1	WP_230046959.1	WP_230045536.1
Bacillota	*Brevibacillus invocatus*	WP_122908283.1	WP_122908779.1	WP_251243155.1
Bacillota	*Brevibacillus laterosporus*	WP_301802748.1	WP_096887578.1	WP_277545643.1
Bacillota	*Brevibacillus marinus*	WP_126427242.1	WP_126427244.1	WP_126425988.1
Bacillota	*Brevibacillus massiliensis*	WP_019122419.1	WP_019122420.1	WP_019120713.1
Bacillota	*Brevibacillus migulae*	WP_134686654.1	WP_134686655.1	WP_134687458.1
Bacillota	*Brevibacillus nitrificans*	WP_328204720.1	WP_310227962.1	WP_310236248.1
Bacillota	*Brevibacillus panacihumi*	WP_427329340.1	WP_122912325.1	WP_122912984.1
Bacillota	*Brevibacillus parabrevis*	WP_328147717.1	WP_063227869.1	WP_122964991.1
Bacillota	*Brevibacillus porteri*	WP_106835973.1	WP_376898458.1	WP_106835688.1
Bacillota	*Brevibacillus reuszeri*	WP_376908201.1	WP_312108564.1	WP_312114022.1
Bacillota	*Brevibacillus ruminantium*	WP_251870562.1	WP_251870563.1	WP_251874646.1
Bacillota	*Brevibacillus sp. B_LB10_24*	WP_408108360.1	WP_408108361.1	WP_408106202.1
Bacillota	*Brevibacillus thermoruber*	WP_271139233.1	WP_029099570.1	WP_035295256.1
Bacillota	*Caenibacillus caldisaponilyticus*	WP_077616334.1	WP_077616392.1	WP_077614480.1

Bacillota	*Caldalkalibacillus mannanilyticus*	WP_025026795.1	WP_025026794.1	WP_025028057.1
Bacillota	*Caldalkalibacillus salinus*	WP_202079974.1	WP_202079975.1	WP_202076947.1
Bacillota	*Caldalkalibacillus thermarum*	WP_007505592.1	WP_042685701.1	WP_188622502.1
Bacillota	*Caldalkalibacillus uzonensis*	WP_307334880.1	WP_307334882.1	WP_307338827.1
Bacillota	*Calderihabitans maritimus*	WP_088553107.1	WP_088554238.1	WP_088554237.1
Bacillota	*Caldibacillus debilis*	WP_276674373.1	WP_333524186.1	WP_061569350.1
Bacillota	*Caldibacillus thermoamylovorans*	WP_227091017.1	WP_251250642.1	WP_251245618.1
Bacillota	*Caldifermentibacillus hisashii*	WP_341288069.1	WP_404997150.1	WP_368997560.1
Bacillota	*Caldinitratiruptor microaerophilus*	WP_264843609.1	WP_264843608.1	WP_264841576.1
Bacillota	*Calditerricola satsumensis*	WP_054672757.1	WP_188816842.1	WP_188816587.1
Bacillota	*Calidifontibacillus erzurumensis*	WP_173731473.1	WP_173730150.1	WP_173729957.1
Bacillota	*Calidifontibacillus oryziterrae*	WP_017755555.1	WP_017755556.1	WP_017753621.1
Bacillota	*Camelliibacillus cellulosilyticus*	WP_376844808.1	WP_376844809.1	WP_376844302.1
Bacillota	*Candidatus Contubernalis alkalaceticus*	WP_241420565.1	WP_241420566.1	WP_241420567.1
Bacillota	*Carboxydothermus ferrireducens*	WP_028052299.1	WP_028052298.1	WP_028052058.1
Bacillota	*Carboxydothermus hydrogenoformans*	WP_011344056.1	WP_011344055.1	WP_011344794.1
Bacillota	*Carboxydothermus islandicus*	WP_075866452.1	WP_075866451.1	WP_075865582.1
Bacillota	*Carboxydothermus pertinax*	WP_075859531.1	WP_075859530.1	WP_075858767.1
Bacillota	*Caryophanon latum*	WP_066465105.1	WP_066466106.1	WP_066463252.1
Bacillota	*Caryophanon tenue*	WP_066548704.1	WP_066547131.1	WP_066543228.1
Bacillota	*Cerasibacillus quisquiliarum*	WP_146938177.1	WP_146934397.1	WP_146935506.1
Bacillota	*Cerasibacillus sp.*	WP_331643333.1	WP_331642528.1	WP_331639209.1
Bacillota	*Cerasibacillus terrae*	WP_147665579.1	WP_147666711.1	WP_147665147.1
Bacillota	*Chengkuizengella axinellae*	WP_305991132.1	WP_305991131.1	WP_305990434.1
Bacillota	*Chengkuizengella marina*	WP_160647308.1	WP_160647307.1	WP_160646215.1
Bacillota	*Chengkuizengella sediminis*	WP_162035760.1	WP_162035759.1	WP_162035053.1
Bacillota	*Chengkuizengella sp. SCS-71B*	WP_349248815.1	WP_349248814.1	WP_349249855.1
Bacillota	*Chryseomicrobium aureum*	WP_204589171.1	WP_204589836.1	WP_392456146.1

Bacillota	*Chryseomicrobium excrementi*	WP_245858730.1	WP_100353346.1	WP_100352793.1
Bacillota	*Chryseomicrobium palamuruense*	WP_378140865.1	WP_378140089.1	WP_378140646.1
Bacillota	*Chryseomicrobium sp. FSL W7-1435*	WP_342525698.1	WP_342527989.1	WP_342525912.1
Bacillota	*Chungangia koreensis*	WP_378155353.1	WP_378154905.1	WP_378151913.1
Bacillota	*Cohnella abietis*	WP_130608606.1	WP_130608603.1	WP_157994083.1
Bacillota	*Cohnella boryungensis*	WP_204603271.1	WP_204603272.1	WP_204601225.1
Bacillota	*Cohnella caldifontis*	WP_276351556.1	WP_276351558.1	WP_276355939.1
Bacillota	*Cohnella candidum*	WP_123040770.1	WP_123040771.1	WP_164472625.1
Bacillota	*Cohnella cellulosilytica*	WP_378047127.1	WP_378047129.1	WP_378054137.1
Bacillota	*Cohnella endophytica*	WP_120974641.1	WP_120974642.1	WP_158602250.1
Bacillota	*Cohnella faecalis*	WP_119147875.1	WP_119147874.1	WP_158593973.1
Bacillota	*Cohnella fermenti*	WP_136370498.1	WP_136370497.1	WP_168735792.1
Bacillota	*Cohnella ginsengisoli*	WP_277563559.1	WP_277563560.1	WP_277567073.1
Bacillota	*Cohnella hashimotonis*	WP_282908436.1	WP_282908435.1	WP_282909408.1
Bacillota	*Cohnella herbarum*	WP_169278750.1	WP_169278749.1	WP_169279893.1
Bacillota	*Cohnella hongkongensis*	WP_378096723.1	WP_378096104.1	WP_378097115.1
Bacillota	*Cohnella kolymensis*	WP_041065952.1	WP_041065955.1	WP_161793416.1
Bacillota	*Cohnella lubricantis*	WP_185180865.1	WP_185180864.1	WP_185178599.1
Bacillota	*Cohnella luojiensis*	WP_135150944.1	WP_135150943.1	WP_167746931.1
Bacillota	*Cohnella lupini*	WP_115993120.1	WP_115993121.1	WP_115990748.1
Bacillota	*Cohnella mopanensis*	WP_239614212.1	WP_239614211.1	WP_239617380.1
Bacillota	*Cohnella nanjingensis*	WP_185671970.1	WP_185671971.1	WP_185671088.1
Bacillota	*Cohnella panacarvi*	WP_027084921.1	WP_084760938.1	WP_027088337.1
Bacillota	*Cohnella pontilimi*	WP_136776128.1	WP_136776129.1	WP_246042130.1
Bacillota	*Cohnella silvisoli*	WP_232183992.1	WP_232183994.1	WP_232187431.1
Bacillota	*Cohnella soli*	WP_378133467.1	WP_378133469.1	WP_378132313.1
Bacillota	*Cohnella sp.*	WP_372634402.1	WP_391571901.1	WP_391570815.1
Bacillota	*Cohnella suwonensis*	WP_209743106.1	WP_209743108.1	WP_378081019.1

Bacillota	*Cohnella terricola*	WP_144698270.1	WP_144698268.1	WP_186438347.1
Bacillota	*Cohnella thailandensis*	WP_185120590.1	WP_185120591.1	WP_185121488.1
Bacillota	*Cohnella thermotolerans*	WP_027093617.1	WP_027093618.1	WP_027091085.1
Bacillota	*Cohnella xylanilytica*	WP_185134228.1	WP_212959663.1	WP_185139369.1
Bacillota	*Cohnella yongneupensis*	WP_378113744.1	WP_378113743.1	WP_378114142.1
Bacillota	*Cohnella zeiphila*	WP_185133116.1	WP_185133115.1	WP_185127330.1
Bacillota	*Compostibacillus humi*	WP_188392063.1	WP_188391814.1	WP_188391400.1
Bacillota	*Corticicoccus populi*	WP_377771250.1	WP_377773644.1	WP_377772180.1
Bacillota	*Croceifilum oryzae*	WP_307254358.1	WP_307254361.1	WP_307250766.1
Bacillota	*Cytobacillus citreus*	WP_213102480.1	WP_420828161.1	WP_213100596.1
Bacillota	*Cytobacillus dafuensis*	WP_057771339.1	WP_407643411.1	WP_057769642.1
Bacillota	*Cytobacillus depressus*	WP_151533025.1	WP_425481971.1	WP_151534101.1
Bacillota	*Cytobacillus eiseniae*	WP_066395413.1	WP_066395414.1	WP_066393352.1
Bacillota	*Cytobacillus firmus*	WP_258758697.1	WP_404355338.1	WP_335525087.1
Bacillota	*Cytobacillus gottheilii*	WP_080845475.1	WP_214478849.1	WP_426860528.1
Bacillota	*Cytobacillus horneckiae*	WP_066200529.1	WP_412860526.1	WP_412859853.1
Bacillota	*Cytobacillus kochii*	WP_281202324.1	WP_404292258.1	WP_328199021.1
Bacillota	*Cytobacillus luteolus*	WP_193539513.1	WP_193539514.1	WP_193536846.1
Bacillota	*Cytobacillus massiliigabonensis*	WP_102274775.1	WP_102274774.1	WP_102271555.1
Bacillota	*Cytobacillus oceanisediminis*	WP_144541601.1	WP_110064210.1	WP_144542403.1
Bacillota	*Cytobacillus praedii*	WP_328165307.1	WP_242691024.1	WP_232426301.1
Bacillota	*Cytobacillus pseudoceanisediminis*	WP_423375193.1	WP_258743562.1	WP_423373599.1
Bacillota	*Cytobacillus purgationiresistens*	WP_307475962.1	WP_307475964.1	WP_307480611.1
Bacillota	*Cytobacillus solani*	WP_053476782.1	WP_056687224.1	WP_231690049.1
Bacillota	*Cytobacillus sp.*	WP_313798514.1	WP_313798513.1	WP_313798771.1
Bacillota	*Cytobacillus spongiae*	WP_233806574.1	WP_233806576.1	WP_233811026.1
Bacillota	*Desertibacillus haloalkaliphilus*	WP_217224796.1	WP_217224795.1	WP_217224580.1
Bacillota	*Desmospora activa*	WP_107725504.1	WP_245891082.1	WP_107726050.1

Bacillota	*Desmospora profundinema*	WP_309861822.1	WP_309861824.1	WP_309862629.1
Bacillota	*Desulforamulus aeronauticus*	WP_072912166.1	WP_238456746.1	WP_072913709.1
Bacillota	*Desulforamulus aquiferis*	WP_304544453.1	WP_304544451.1	WP_304541554.1
Bacillota	*Desulforamulus ferrireducens*	WP_077714789.1	WP_077714790.1	WP_077713567.1
Bacillota	*Desulforamulus hydrothermalis*	WP_008413214.1	WP_235695578.1	WP_008412797.1
Bacillota	*Desulforamulus putei*	WP_073238853.1	WP_073238856.1	WP_073238516.1
Bacillota	*Desulforamulus reducens*	WP_011878631.1	WP_011878632.1	WP_011877534.1
Bacillota	*Desulforamulus ruminis*	WP_013841388.1	WP_013841387.1	WP_013842872.1
Bacillota	*Desulfosporosinus acididurans*	WP_047810799.1	WP_047808470.1	WP_047810809.1
Bacillota	*Desulfosporosinus acidiphilus*	WP_014828645.1	WP_041276197.1	WP_014828658.1
Bacillota	*Desulfosporosinus fructosivorans*	WP_135544966.1	WP_135544965.1	WP_243454315.1
Bacillota	*Desulfosporosinus hippei*	WP_092328753.1	WP_092331157.1	WP_092328781.1
Bacillota	*Desulfosporosinus lacus*	WP_073028355.1	WP_073031454.1	WP_073028368.1
Bacillota	*Desulfosporosinus meridiei*	WP_014904613.1	WP_014904331.1	WP_014904626.1
Bacillota	*Desulfosporosinus metallidurans*	WP_075363497.1	WP_075365157.1	WP_075363507.1
Bacillota	*Desulfosporosinus nitroreducens*	WP_301997921.1	WP_252470087.1	WP_301997935.1
Bacillota	*Desulfosporosinus orientis*	WP_014187046.1	WP_014186735.1	WP_014187063.1
Bacillota	*Desulfosporosinus paludis*	WP_407307408.1	WP_407305579.1	WP_407307383.1
Bacillota	*Desulfosporosinus sp.*	WP_291352846.1	WP_291349225.1	WP_298201967.1
Bacillota	*Desulfosporosinus youngiae*	WP_007786549.1	WP_007786032.1	WP_007786564.1
Bacillota	*Desulfotomaculum nigrificans*	WP_013810337.1	WP_003543278.1	WP_234702063.1
Bacillota	*Desulfotomaculum sp. 1211_IL3151*	WP_333530773.1	WP_333530162.1	WP_333529912.1
Bacillota	*Dethiobacter alkaliphilus*	WP_008516141.1	WP_008516142.1	WP_008516143.1
Bacillota	*Domibacillus aminovorans*	WP_063966893.1	WP_063975283.1	WP_063975316.1
Bacillota	*Domibacillus antri*	WP_075397513.1	WP_425429437.1	WP_075398213.1
Bacillota	*Domibacillus enclensis*	WP_045851444.1	WP_045852543.1	WP_045851456.1
Bacillota	*Domibacillus epiphyticus*	WP_076767700.1	WP_083711496.1	WP_076762848.1
Bacillota	*Domibacillus indicus*	WP_251270905.1	WP_197076570.1	WP_046175139.1

Bacillota	*Domibacillus iocasae*	WP_069939096.1	WP_069938662.1	WP_069939483.1
Bacillota	*Domibacillus mangrovi*	WP_073709983.1	WP_073710831.1	WP_073711090.1
Bacillota	*Domibacillus robiginosus*	WP_050180706.1	WP_050180456.1	WP_050181536.1
Bacillota	*Domibacillus sp.*	WP_309087748.1	WP_309089351.1	WP_309087016.1
Bacillota	*Domibacillus tundrae*	WP_353913610.1	WP_245618035.1	WP_046179655.1
Bacillota	*Ectobacillus antri*	WP_124563901.1	WP_124563900.1	WP_124563576.1
Bacillota	*Ectobacillus funiculus*	WP_379947984.1	WP_379947985.1	WP_410775892.1
Bacillota	*Ectobacillus panaciterrae*	WP_028402725.1	WP_028402724.1	WP_028398909.1
Bacillota	*Ectobacillus polymachus*	WP_416827846.1	WP_416827847.1	WP_416826947.1
Bacillota	*Ectobacillus ponti*	WP_254758693.1	WP_254758692.1	WP_254760171.1
Bacillota	*Ectobacillus sp. sgz5001026*	WP_379970274.1	WP_379970275.1	WP_379967020.1
Bacillota	*Edaphobacillus lindanitolerans*	WP_076756388.1	WP_076756839.1	WP_076756591.1
Bacillota	*Evansella cellulosilytica*	WP_013488498.1	WP_013488497.1	WP_013489193.1
Bacillota	*Evansella clarkii*	WP_088035635.1	WP_078596894.1	WP_078595466.1
Bacillota	*Evansella halocellulosilytica*	WP_096188656.1	WP_096188655.1	WP_096189230.1
Bacillota	*Evansella sp. LMS18*	WP_255242124.1	WP_255242123.1	WP_255243417.1
Bacillota	*Evansella tamaricis*	WP_217069329.1	WP_217069385.1	WP_217066593.1
Bacillota	*Evansella vedderi*	WP_307331180.1	WP_307331183.1	WP_307322987.1
Bacillota	*Exiguobacterium algae*	WP_214826552.1	WP_240544489.1	WP_338082714.1
Bacillota	*Exiguobacterium alkaliphilum*	WP_367660416.1	WP_034815198.1	WP_246514194.1
Bacillota	*Exiguobacterium aurantiacum*	WP_070327419.1	WP_255176439.1	WP_029335464.1
Bacillota	*Exiguobacterium chiriqhucha*	WP_276693499.1	WP_276693498.1	WP_031200163.1
Bacillota	*Exiguobacterium flavidum*	WP_114570975.1	WP_114572129.1	WP_114571240.1
Bacillota	*Exiguobacterium indicum*	WP_058713743.1	WP_411212175.1	WP_075642557.1
Bacillota	*Exiguobacterium oxidotolerans*	WP_029330621.1	WP_029332029.1	WP_029330917.1
Bacillota	*Exiguobacterium qingdaonense*	WP_215141475.1	WP_215141476.1	WP_338082249.1
Bacillota	*Falsibacillus albus*	WP_121680777.1	WP_121680776.1	WP_121680364.1
Bacillota	*Falsibacillus pallidus*	WP_114745338.1	WP_114745337.1	WP_114743777.1

Bacillota	*Ferdinandcohnia quinoae*	WP_240253376.1	WP_240253378.1	WP_240251968.1
Bacillota	*Ferroacidibacillus organovorans*	WP_067564900.1	WP_067560444.1	WP_079290518.1
Bacillota	*Fervidibacillus albus*	WP_420842691.1	WP_275417391.1	WP_275418420.1
Bacillota	*Fervidibacillus halotolerans*	WP_275421935.1	WP_275420367.1	WP_275421882.1
Bacillota	*Ferviditalea candida*	WP_371753413.1	WP_371753412.1	WP_371752757.1
Bacillota	*Fictibacillus aquaticus*	WP_205727361.1	WP_094250508.1	WP_094250912.1
Bacillota	*Fictibacillus arsenicus*	WP_077364071.1	WP_077364069.1	WP_066289143.1
Bacillota	*Fictibacillus barbaricus*	WP_188402068.1	WP_188402067.1	WP_310261459.1
Bacillota	*Fictibacillus enclensis*	WP_283889052.1	WP_289485947.1	WP_289485337.1
Bacillota	*Fictibacillus fluitans*	WP_301163916.1	WP_301164878.1	WP_301164551.1
Bacillota	*Fictibacillus gelatini*	WP_026676973.1	WP_026676972.1	WP_026675974.1
Bacillota	*Fictibacillus halophilus*	WP_379834442.1	WP_226534979.1	WP_379833681.1
Bacillota	*Fictibacillus iocasae*	WP_379746025.1	WP_379746023.1	WP_379749286.1
Bacillota	*Fictibacillus macauensis*	WP_007200809.1	WP_007200808.1	WP_007202444.1
Bacillota	*Fictibacillus marinisediminis*	WP_248252980.1	WP_248252981.1	WP_248252504.1
Bacillota	*Fictibacillus nanhaiensis*	WP_251320614.1	WP_328084593.1	WP_251322428.1
Bacillota	*Fictibacillus norfolkensis*	WP_191753992.1	WP_191753991.1	WP_191753699.1
Bacillota	*Fictibacillus phosphorivorans*	WP_251312548.1	WP_251312549.1	WP_251310970.1
Bacillota	*Fictibacillus solisalsi*	WP_090234070.1	WP_090234071.1	WP_090233142.1
Bacillota	*Fictibacillus sp. FJAT-27399*	WP_062235415.1	WP_062238660.1	WP_062231614.1
Bacillota	*Fictibacillus terranigra*	WP_290400662.1	WP_290400663.1	WP_290399658.1
Bacillota	*Filibacter tadaridae*	WP_124070649.1	WP_203229615.1	WP_124071008.1
Bacillota	*Filobacillus milosensis*	WP_134339040.1	WP_134338713.1	WP_134339379.1
Bacillota	*Fodinisporobacter ferrooxydans*	WP_347436385.1	WP_347436386.1	WP_347435826.1
Bacillota	*Fontibacillus panacisegetis*	WP_091229626.1	WP_091229628.1	WP_091226566.1
Bacillota	*Fontibacillus phaseoli*	WP_114495195.1	WP_114495194.1	WP_114494769.1
Bacillota	*Fontibacillus solani*	WP_182535212.1	WP_182535213.1	WP_182535122.1
Bacillota	*Fontibacillus sp. BL9*	WP_410768391.1	WP_410768390.1	WP_410768037.1

Bacillota	*Fredinandcohnia onubensis*	WP_099362744.1	WP_099363396.1	WP_099363096.1
Bacillota	*Fredinandcohnia salidurans*	WP_388034606.1	WP_304214230.1	WP_388035390.1
Bacillota	*Fredinandcohnia sp. QZ13*	WP_309536700.1	WP_309534390.1	WP_309534811.1
Bacillota	*Fuchsiella alkaliacetigena*	WP_248662616.1	WP_248662617.1	WP_248662618.1
Bacillota	*Geobacillus genomosp. 3*	WP_020960296.1	WP_041267944.1	WP_020959237.1
Bacillota	*Geobacillus jurassicus*	WP_066229184.1	WP_066229281.1	WP_066233987.1
Bacillota	*Geobacillus kaustophilus*	WP_020278320.1	WP_044731029.1	WP_044736192.1
Bacillota	*Geobacillus sp. JS12*	WP_063192935.1	WP_063192936.1	WP_063192325.1
Bacillota	*Geobacillus stearothermophilus*	WP_277391634.1	WP_063210378.1	WP_237420055.1
Bacillota	*Geobacillus subterraneus*	WP_184318169.1	WP_168369042.1	WP_184317307.1
Bacillota	*Geobacillus thermodenitrificans*	WP_327986912.1	WP_328191681.1	WP_100660330.1
Bacillota	*Geomicrobium halophilum*	WP_184403564.1	WP_184404824.1	WP_184403282.1
Bacillota	*Geomicrobium sediminis*	WP_204698194.1	WP_204696653.1	WP_204696779.1
Bacillota	*Gordoniibacillus kamchatkensis*	WP_041046862.1	WP_041046861.1	WP_041046255.1
Bacillota	*Gorillibacterium sp. CAU 1737*	WP_347768118.1	WP_347768119.1	WP_347772139.1
Bacillota	*Gorillibacterium timonense*	WP_058303859.1	WP_058303858.1	WP_082651929.1
Bacillota	*Gottfriedia acidiceleris*	WP_335486042.1	WP_088010671.1	WP_088013047.1
Bacillota	*Gottfriedia endophytica*	WP_209402472.1	WP_209402474.1	WP_209403689.1
Bacillota	*Gottfriedia luciferensis*	WP_088069167.1	WP_088069169.1	WP_088067724.1
Bacillota	*Gottfriedia solisilvae*	WP_087999426.1	WP_087999425.1	WP_088000236.1
Bacillota	*Gottfriedia sp. NPDC056225*	WP_172444130.1	WP_172444023.1	WP_375949226.1
Bacillota	*Gracilibacillus alcaliphilus*	WP_204667049.1	WP_204667842.1	WP_204670532.1
Bacillota	*Gracilibacillus boraciitolerans*	WP_035721112.1	WP_035722829.1	WP_084040548.1
Bacillota	*Gracilibacillus caseinilyticus*	WP_244716889.1	WP_244718528.1	WP_244717793.1
Bacillota	*Gracilibacillus halophilus*	WP_003466106.1	WP_003462699.1	WP_003463201.1
Bacillota	*Gracilibacillus halotolerans*	WP_184244035.1	WP_184249427.1	WP_184243471.1
Bacillota	*Gracilibacillus kekensis*	WP_073202086.1	WP_073201074.1	WP_073201781.1
Bacillota	*Gracilibacillus lacisalsi*	WP_018933731.1	WP_018930976.1	WP_018932777.1

Bacillota	*Gracilibacillus marinus*	WP_390197772.1	WP_390196412.1	WP_390196950.1
Bacillota	*Gracilibacillus massiliensis*	WP_058307601.1	WP_058306746.1	WP_058305611.1
Bacillota	*Gracilibacillus orientalis*	WP_091483469.1	WP_091484803.1	WP_091483945.1
Bacillota	*Gracilibacillus oryzae*	WP_153401852.1	WP_153403976.1	WP_153401574.1
Bacillota	*Gracilibacillus salinarum*	WP_244746134.1	WP_244743910.1	WP_244744755.1
Bacillota	*Gracilibacillus saliphilus*	WP_163581994.1	WP_163579037.1	WP_163579670.1
Bacillota	*Gracilibacillus salitolerans*	WP_100360378.1	WP_153791010.1	WP_153791192.1
Bacillota	*Gracilibacillus sp. S3-1-1*	WP_319957970.1	WP_319956460.1	WP_319956720.1
Bacillota	*Gracilibacillus suaedae*	WP_208591048.1	WP_208587972.1	WP_208589337.1
Bacillota	*Gracilibacillus thailandensis*	WP_153836484.1	WP_163578820.1	WP_153835392.1
Bacillota	*Gracilibacillus ureilyticus*	WP_089738304.1	WP_089741081.1	WP_089741886.1
Bacillota	*Gracilibacillus xinjiangensis*	WP_390252955.1	WP_390254003.1	WP_390253558.1
Bacillota	*Halalkalibacillus halophilus*	WP_245570102.1	WP_027965402.1	WP_035512343.1
Bacillota	*Halalkalibacillus sediminis*	WP_101330576.1	WP_101331615.1	WP_101330273.1
Bacillota	*Halalkalibacter akibai*	WP_035663521.1	WP_035663519.1	WP_148296803.1
Bacillota	*Halalkalibacter alkaliphilus*	WP_250094869.1	WP_250094868.1	WP_250095283.1
Bacillota	*Halalkalibacter alkalisediminis*	WP_273840757.1	WP_273840758.1	WP_273839757.1
Bacillota	*Halalkalibacter flavus*	WP_332630148.1	WP_332630147.1	WP_332632203.1
Bacillota	*Halalkalibacter hemicellulosilyticus*	WP_035340208.1	WP_369384506.1	WP_035340416.1
Bacillota	*Halalkalibacter kiskunsagensis*	WP_335958984.1	WP_335958985.1	WP_335960984.1
Bacillota	*Halalkalibacter krulwichiae*	WP_066151466.1	WP_066151468.1	WP_066150491.1
Bacillota	*Halalkalibacter lacteus*	WP_332693267.1	WP_332693270.1	WP_332690015.1
Bacillota	*Halalkalibacter nanhaiisediminis*	WP_144448998.1	WP_144448997.1	WP_158640037.1
Bacillota	*Halalkalibacter oceani*	WP_354571001.1	WP_251193962.1	WP_251192048.1
Bacillota	*Halalkalibacter okhensis*	WP_034628459.1	WP_052144674.1	WP_034627251.1
Bacillota	*Halalkalibacter urbisdiaboli*	WP_088102184.1	WP_088102185.1	WP_088104994.1
Bacillota	*Halalkalibacter wakoensis*	WP_034742903.1	WP_034742929.1	WP_052001971.1
Bacillota	*Halalkalibacterium halodurans*	WP_328033658.1	WP_233444599.1	WP_010898728.1

Bacillota	*Halalkalibacterium ligniniphilum*	WP_017728002.1	WP_017728003.1	WP_017727198.1
Bacillota	*Halobacillus aidingensis*	WP_089651930.1	WP_089651334.1	WP_089652803.1
Bacillota	*Halobacillus alkaliphilus*	WP_089750055.1	WP_089751867.1	WP_089751895.1
Bacillota	*Halobacillus amylolyticus*	WP_245033281.1	WP_245035014.1	WP_245034112.1
Bacillota	*Halobacillus andaensis*	WP_188375880.1	WP_188377783.1	WP_188376172.1
Bacillota	*Halobacillus campisalis*	WP_289214293.1	WP_289215238.1	WP_289214028.1
Bacillota	*Halobacillus dabanensis*	WP_075036701.1	WP_075037406.1	WP_075036544.1
Bacillota	*Halobacillus faecis*	WP_371860329.1	WP_146815254.1	WP_146818693.1
Bacillota	*Halobacillus halophilus*	WP_224892577.1	WP_224893246.1	WP_160840210.1
Bacillota	*Halobacillus hunanensis*	WP_079530109.1	WP_079526548.1	WP_079529747.1
Bacillota	*Halobacillus ihumii*	WP_163526213.1	WP_163529223.1	WP_163530396.1
Bacillota	*Halobacillus karajensis*	WP_035504949.1	WP_035508604.1	WP_074733901.1
Bacillota	*Halobacillus kuroshimensis*	WP_206932799.1	WP_206934211.1	WP_027954104.1
Bacillota	*Halobacillus litoralis*	WP_226579282.1	WP_225196267.1	WP_128522867.1
Bacillota	*Halobacillus locisalis*	WP_181471713.1	WP_181473500.1	WP_181471280.1
Bacillota	*Halobacillus mangrovi*	WP_085029742.1	WP_085030800.1	WP_085030042.1
Bacillota	*Halobacillus massiliensis*	WP_082234011.1	WP_082235033.1	WP_082234268.1
Bacillota	*Halobacillus naozhouensis*	WP_283075109.1	WP_283077805.1	WP_283078586.1
Bacillota	*Halobacillus rhizosphaerae*	WP_411787596.1	WP_411790746.1	WP_411790457.1
Bacillota	*Halobacillus salinarum*	WP_244708082.1	WP_244709258.1	WP_244708500.1
Bacillota	*Halobacillus salinus*	WP_079479978.1	WP_079479320.1	WP_135327270.1
Bacillota	*Halobacillus seohaensis*	WP_204706383.1	WP_204710739.1	WP_204707910.1
Bacillota	*Halobacillus shinanisalinarum*	WP_244753174.1	WP_244752164.1	WP_244752680.1
Bacillota	*Halobacillus sp.*	WP_422165301.1	WP_422167379.1	WP_422168518.1
Bacillota	*Halobacillus trueperi*	WP_394217372.1	WP_394219676.1	WP_115824858.1
Bacillota	*Halobacillus yeomjeoni*	WP_197316167.1	WP_197317823.1	WP_197315676.1
Bacillota	*Hazenella coriacea*	WP_131925631.1	WP_131925633.1	WP_131924031.1
Bacillota	*Heliobacterium chlorum*	WP_188038396.1	WP_188038395.1	WP_188041012.1

Bacillota	*Heliobacterium mobile*	WP_170291528.1	WP_155474654.1	WP_155477797.1
Bacillota	*Heliomicrobium modesticaldum*	WP_012283098.1	WP_012283097.1	WP_012283553.1
Bacillota	*Heliomicrobium undosum*	WP_161255425.1	WP_161255422.1	WP_161254746.1
Bacillota	*Heliorestis acidaminivorans*	WP_151618080.1	WP_170270103.1	WP_170270043.1
Bacillota	*Heliorestis convoluta*	WP_153725135.1	WP_153725134.1	WP_153724431.1
Bacillota	*Heyndrickxia acidicola*	WP_066262737.1	WP_066262736.1	WP_066264835.1
Bacillota	*Heyndrickxia acidiproducens*	WP_018660367.1	WP_018660369.1	WP_018664087.1
Bacillota	*Heyndrickxia camelliae*	WP_101353257.1	WP_101353258.1	WP_101352608.1
Bacillota	*Heyndrickxia coagulans*	WP_308928048.1	WP_328004612.1	WP_046721295.1
Bacillota	*Heyndrickxia faecalis*	WP_350346116.1	WP_350346115.1	WP_211062182.1
Bacillota	*Heyndrickxia ginsengihumi*	WP_025729133.1	WP_231473063.1	WP_025730116.1
Bacillota	*Heyndrickxia oleronia*	WP_280615767.1	WP_251345137.1	WP_212942048.1
Bacillota	*Heyndrickxia sp. FSL K6-6286*	WP_342541230.1	WP_342541229.1	WP_342539788.1
Bacillota	*Heyndrickxia sporothermodurans*	WP_066228585.1	WP_066228588.1	WP_066230041.1
Bacillota	*Heyndrickxia vini*	WP_202780324.1	WP_202780323.1	WP_202776866.1
Bacillota	*Hydrogenibacillus schlegelii*	WP_066203583.1	WP_169816914.1	WP_082718315.1
Bacillota	*Indiicoccus explosivorum*	WP_088007583.1	WP_088008730.1	WP_241535823.1
Bacillota	*Insulibacter thermoxylanivorax*	WP_200965202.1	WP_200965920.1	WP_200965739.1
Bacillota	*Jeotgalibacillus alimentarius*	WP_200889064.1	WP_041122687.1	WP_041122314.1
Bacillota	*Jeotgalibacillus aurantiacus*	WP_227395293.1	WP_227395292.1	WP_227395612.1
Bacillota	*Jeotgalibacillus campisalis*	WP_041057981.1	WP_041057983.1	WP_041057289.1
Bacillota	*Jeotgalibacillus haloalkalitolerans*	WP_322420988.1	WP_322420989.1	WP_322420580.1
Bacillota	*Jeotgalibacillus malaysiensis*	WP_404403476.1	WP_404403478.1	WP_404409007.1
Bacillota	*Jeotgalibacillus marinus*	WP_367777475.1	WP_367777476.1	WP_367778874.1
Bacillota	*Jeotgalibacillus proteolyticus*	WP_104056735.1	WP_104056736.1	WP_104056138.1
Bacillota	*Jeotgalibacillus salarius*	WP_134380087.1	WP_134380089.1	WP_134382059.1
Bacillota	*Jeotgalibacillus soli*	WP_041090089.1	WP_041090090.1	WP_041088882.1
Bacillota	*Jeotgalibacillus sp. S-D1*	WP_133375592.1	WP_133376314.1	WP_133375963.1

Bacillota	*Jeotgalibacillus terrae*	WP_204727679.1	WP_204727680.1	WP_204728610.1
Bacillota	*Jeotgalicoccus aerolatus*	WP_186090071.1	WP_092597945.1	WP_186090400.1
Bacillota	*Jeotgalicoccus coquinae*	WP_184282109.1	WP_184283941.1	WP_184281317.1
Bacillota	*Jeotgalicoccus halotolerans*	WP_369890706.1	WP_115885244.1	WP_115885716.1
Bacillota	*Jeotgalicoccus meleagridis*	WP_185125737.1	WP_185125207.1	WP_185126131.1
Bacillota	*Jeotgalicoccus psychrophilus*	WP_026858060.1	WP_026858943.1	WP_026858474.1
Bacillota	*Jeotgalicoccus saudimassiliensis*	WP_035808832.1	WP_035810548.1	WP_035807980.1
Bacillota	*Jeotgalicoccus sp. ATCC 8456*	WP_198687648.1	WP_198687319.1	WP_198687862.1
Bacillota	*Kroppenstedtia eburnea*	WP_076522904.1	WP_040387154.1	WP_076523359.1
Bacillota	*Kroppenstedtia guangzhouensis*	WP_188430415.1	WP_188430417.1	WP_188428771.1
Bacillota	*Kroppenstedtia pulmonis*	WP_173221993.1	WP_173221991.1	WP_173222804.1
Bacillota	*Kroppenstedtia sanguinis*	WP_380163115.1	WP_380163114.1	WP_380164071.1
Bacillota	*Kurthia gibsonii*	WP_325942863.1	WP_368634274.1	WP_325921672.1
Bacillota	*Kurthia huakuii*	WP_029498452.1	WP_029497763.1	WP_029498146.1
Bacillota	*Kurthia massiliensis*	WP_026021961.1	WP_010287167.1	WP_010286585.1
Bacillota	*Kurthia senegalensis*	WP_026022481.1	WP_010302634.1	WP_010301772.1
Bacillota	*Kurthia sibirica*	WP_109307152.1	WP_109307299.1	WP_109304434.1
Bacillota	*Kurthia sp. FSL E2-0154*	WP_340995847.1	WP_340998718.1	WP_340995481.1
Bacillota	*Kurthia zopfii*	WP_109350159.1	WP_109350915.1	WP_109349916.1
Bacillota	*Laceyella putida*	WP_379865747.1	WP_379865748.1	WP_379866126.1
Bacillota	*Laceyella sacchari*	WP_054096534.1	WP_259436617.1	WP_259435664.1
Bacillota	*Laceyella sediminis*	WP_022737412.1	WP_106342438.1	WP_106341380.1
Bacillota	*Laceyella tengchongensis*	WP_102992298.1	WP_333639873.1	WP_102992870.1
Bacillota	*Lacicoccus alkaliphilus*	WP_072707734.1	WP_072710414.1	WP_072708462.1
Bacillota	*Lacicoccus qingdaonensis*	WP_092984866.1	WP_092985141.1	WP_092983623.1
Bacillota	*Lederbergia citrea*	WP_213097146.1	WP_213106924.1	WP_213107223.1
Bacillota	*Lederbergia citri*	WP_213123659.1	WP_213123660.1	WP_213123119.1
Bacillota	*Lederbergia citrisecunda*	WP_213110163.1	WP_213110164.1	WP_213109577.1

Bacillota	*Lederbergia galactosidilytica*	WP_057988245.1	WP_057988247.1	WP_057985812.1
Bacillota	*Lederbergia graminis*	WP_382346605.1	WP_144919802.1	WP_382347844.1
Bacillota	*Lederbergia lenta*	WP_066137836.1	WP_251519880.1	WP_066139938.1
Bacillota	*Lederbergia panacisoli*	WP_257579653.1	WP_257579654.1	WP_257580260.1
Bacillota	*Lederbergia ruris*	WP_158322329.1	WP_212965587.1	WP_212966198.1
Bacillota	*Lederbergia sp. NSJ-179*	WP_244699947.1	WP_244699945.1	WP_244700086.1
Bacillota	*Lederbergia wuyishanensis*	WP_244680233.1	WP_244680234.1	WP_244679783.1
Bacillota	*Lentibacillus amyloliquefaciens*	WP_068442481.1	WP_068447796.1	WP_068440804.1
Bacillota	*Lentibacillus cibarius*	WP_142791518.1	WP_142792004.1	WP_142791314.1
Bacillota	*Lentibacillus halodurans*	WP_090232615.1	WP_090234410.1	WP_090238274.1
Bacillota	*Lentibacillus halophilus*	WP_343753122.1	WP_343754212.1	WP_343753559.1
Bacillota	*Lentibacillus jeotgali*	WP_010530800.1	WP_029329174.1	WP_010530442.1
Bacillota	*Lentibacillus juripiscarius*	WP_382392739.1	WP_382393513.1	WP_382392120.1
Bacillota	*Lentibacillus kapialis*	WP_188631484.1	WP_188634106.1	WP_188631645.1
Bacillota	*Lentibacillus kimchii*	WP_382361417.1	WP_382360135.1	WP_382357189.1
Bacillota	*Lentibacillus persicus*	WP_090087577.1	WP_090084942.1	WP_090083743.1
Bacillota	*Lentibacillus salicampi*	WP_135109086.1	WP_135109587.1	WP_135108198.1
Bacillota	*Lentibacillus salinarum*	WP_382397247.1	WP_382399436.1	WP_382396958.1
Bacillota	*Lentibacillus saliphilus*	WP_217585454.1	WP_217588552.1	WP_217585692.1
Bacillota	*Lentibacillus sediminis*	WP_100010811.1	WP_100010446.1	WP_100011055.1
Bacillota	*Lentibacillus sp.*	WP_324223546.1	WP_324224588.1	WP_324224008.1
Bacillota	*Lihuaxuella thermophila*	WP_244527535.1	WP_089969006.1	WP_089966921.1
Bacillota	*Litchfieldia alkalitelluris*	WP_078546123.1	WP_078546122.1	WP_078547319.1
Bacillota	*Litchfieldia salsa*	WP_090852835.1	WP_090852838.1	WP_090850473.1
Bacillota	*Litoribacterium kuwaitense*	WP_165199021.1	WP_165199020.1	WP_165199860.1
Bacillota	*Longirhabdus pacifica*	WP_128894412.1	WP_128894413.1	WP_128896012.1
Bacillota	*Lottiidibacillus patelloidae*	WP_094923848.1	WP_094923846.1	WP_094921476.1
Bacillota	*Lysinibacillus alkalisoli*	WP_188613934.1	WP_188613427.1	WP_188614344.1

Bacillota	*Lysinibacillus antri*	WP_126657732.1	WP_126658849.1	WP_126657660.1
Bacillota	*Lysinibacillus capsici*	WP_320940576.1	WP_333880215.1	WP_333880887.1
Bacillota	*Lysinibacillus cavernae*	WP_155590643.1	WP_155592437.1	WP_155590130.1
Bacillota	*Lysinibacillus composti*	WP_124764968.1	WP_124763490.1	WP_124764103.1
Bacillota	*Lysinibacillus contaminans*	WP_053584004.1	WP_053585202.1	WP_053584453.1
Bacillota	*Lysinibacillus endophyticus*	WP_341966957.1	WP_253423194.1	WP_253423882.1
Bacillota	*Lysinibacillus fusiformis*	WP_025114698.1	WP_330493602.1	WP_143997544.1
Bacillota	*Lysinibacillus louembei*	WP_319836654.1	WP_319837291.1	WP_319836882.1
Bacillota	*Lysinibacillus macroides*	WP_053994351.1	WP_053993183.1	WP_053993826.1
Bacillota	*Lysinibacillus odysseyi*	WP_036154513.1	WP_036153162.1	WP_036156049.1
Bacillota	*Lysinibacillus pakistanensis*	WP_054770305.1	WP_283870672.1	WP_283871065.1
Bacillota	*Lysinibacillus parviboronicapiens*	WP_354471761.1	WP_107947582.1	WP_107947661.1
Bacillota	*Lysinibacillus piscis*	WP_264986785.1	WP_264987881.1	WP_264987283.1
Bacillota	*Lysinibacillus sp. NPDC093210*	WP_401008257.1	WP_401010871.1	WP_401009567.1
Bacillota	*Lysinibacillus sphaericus*	WP_197141462.1	WP_142509402.1	WP_024362149.1
Bacillota	*Lysinibacillus telephonicus*	WP_126294168.1	WP_126293219.1	WP_346235537.1
Bacillota	*Lysinibacillus timonensis*	WP_106784717.1	WP_106783408.1	WP_106784036.1
Bacillota	*Lysinibacillus xylanilyticus*	WP_049666747.1	WP_402983101.1	WP_400959240.1
Bacillota	*Lysinibacillus yapensis*	WP_118874311.1	WP_118875069.1	WP_118874754.1
Bacillota	*Macrococcoides bohemicum*	WP_419743646.1	WP_199798155.1	WP_312039520.1
Bacillota	*Macrococcoides canis*	WP_254253336.1	WP_138072676.1	WP_086042282.1
Bacillota	*Macrococcoides caseolyticum*	WP_233681270.1	WP_233681943.1	WP_157820940.1
Bacillota	*Macrococcoides goetzii*	WP_099578185.1	WP_205757173.1	WP_099580771.1
Bacillota	*Macrococcus armenti*	WP_224186387.1	WP_224187339.1	WP_224184470.1
Bacillota	*Macrococcus bovicus*	WP_289649503.1	WP_289650147.1	WP_289650867.1
Bacillota	*Macrococcus brunensis*	WP_239046908.1	WP_133431868.1	WP_239035515.1
Bacillota	*Macrococcus carouselicus*	WP_133417120.1	WP_133418089.1	WP_133416794.1
Bacillota	*Macrococcus epidermidis*	WP_254256476.1	WP_254256905.1	WP_254257470.1

Bacillota	*Macrococcus equipercicus*	WP_149458850.1	WP_149459263.1	WP_254250417.1
Bacillota	*Macrococcus hajekii*	WP_133429085.1	WP_133430543.1	WP_133429380.1
Bacillota	*Macrococcus lamae*	WP_133442833.1	WP_133443643.1	WP_133444362.1
Bacillota	*Macrococcus sp. DPC7161*	WP_129064231.1	WP_129065470.1	WP_164993313.1
Bacillota	*Mammaliicoccus fleurettii*	WP_115337434.1	WP_208144256.1	WP_218702747.1
Bacillota	*Mammaliicoccus lentus*	WP_210618937.1	WP_210617024.1	WP_416349754.1
Bacillota	*Mammaliicoccus sciuri*	WP_204187928.1	WP_206165146.1	WP_107617817.1
Bacillota	*Mammaliicoccus sp. Dog046*	WP_323702337.1	WP_323703356.1	WP_323702670.1
Bacillota	*Mammaliicoccus stepanovicii*	WP_095087978.1	WP_095089715.1	WP_095088520.1
Bacillota	*Mammaliicoccus vitulinus*	WP_421040920.1	WP_103322196.1	WP_107536473.1
Bacillota	*Mangrovibacillus sp. Mu-81*	WP_334218689.1	WP_334218688.1	WP_334217387.1
Bacillota	*Marinicrinis lubricantis*	WP_379893406.1	WP_379893408.1	WP_379895403.1
Bacillota	*Marinicrinis sediminis*	WP_379930489.1	WP_379930488.1	WP_379929885.1
Bacillota	*Marininema halotolerans*	WP_245838860.1	WP_091838700.1	WP_176391884.1
Bacillota	*Marininema mesophilum*	WP_091737720.1	WP_091737717.1	WP_091734794.1
Bacillota	*Marinithermofilum abyssi*	WP_188646919.1	WP_188646918.1	WP_188648308.1
Bacillota	*Marinococcus halophilus*	WP_079476570.1	WP_079476571.1	WP_079476090.1
Bacillota	*Marinococcus halotolerans*	WP_022792248.1	WP_022792249.1	WP_022792687.1
Bacillota	*Marinococcus luteus*	WP_091611148.1	WP_091611152.1	WP_091610196.1
Bacillota	*Massilibacterium senegalense*	WP_062198225.1	WP_062198226.1	WP_062197761.1
Bacillota	*Mechercharimyces sp. CAU 1602*	WP_258839241.1	WP_258839240.1	WP_258838868.1
Bacillota	*Melghiribacillus thermohalophilus*	WP_132370988.1	WP_132372634.1	WP_132370283.1
Bacillota	*Melghirimyces algeriensis*	WP_142504252.1	WP_246064816.1	WP_142504508.1
Bacillota	*Melghirimyces profundicolus*	WP_108023223.1	WP_108023221.1	WP_108026146.1
Bacillota	*Melghirimyces thermohalophilus*	WP_091569222.1	WP_091569219.1	WP_091565521.1
Bacillota	*Mesobacillus boroniphilus*	WP_213368229.1	WP_425374501.1	WP_213366746.1
Bacillota	*Mesobacillus campisalis*	WP_173427554.1	WP_046524176.1	WP_046522237.1
Bacillota	*Mesobacillus foraminis*	WP_412919659.1	WP_412919660.1	WP_132005738.1

Bacillota	*Mesobacillus harenae*	WP_174733990.1	WP_174733991.1	WP_174733830.1
Bacillota	*Mesobacillus jeotgali*	WP_311071330.1	WP_079509534.1	WP_192470257.1
Bacillota	*Mesobacillus maritimus*	WP_221872545.1	WP_404329097.1	WP_251425668.1
Bacillota	*Mesobacillus persicus*	WP_090744525.1	WP_090744528.1	WP_090747490.1
Bacillota	*Mesobacillus selenatarsenatis*	WP_167831352.1	WP_084135369.1	WP_041965511.1
Bacillota	*Mesobacillus sp.*	WP_423991807.1	WP_423991813.1	WP_423990406.1
Bacillota	*Mesobacillus subterraneus*	WP_226642288.1	WP_246017800.1	WP_125479208.1
Bacillota	*Mesobacillus thioparans*	WP_415817540.1	WP_415817665.1	WP_415812484.1
Bacillota	*Mesobacillus zeae*	WP_181213417.1	WP_119114370.1	WP_119111472.1
Bacillota	*Metabacillus arenae*	WP_191159270.1	WP_191159271.1	WP_191157009.1
Bacillota	*Metabacillus bambusae*	WP_207980778.1	WP_207980779.1	WP_207975146.1
Bacillota	*Metabacillus crassostreae*	WP_204954254.1	WP_204954255.1	WP_204953265.1
Bacillota	*Metabacillus dongyingensis*	WP_421175340.1	WP_421175339.1	WP_421176627.1
Bacillota	*Metabacillus endolithicus*	WP_247344015.1	WP_247344017.1	WP_247341532.1
Bacillota	*Metabacillus fastidiosus*	WP_328027515.1	WP_412758905.1	WP_326173759.1
Bacillota	*Metabacillus flavus*	WP_211558719.1	WP_211558721.1	WP_211557961.1
Bacillota	*Metabacillus halosaccharovorans*	WP_264144479.1	WP_216774187.1	WP_216773076.1
Bacillota	*Metabacillus herbersteinensis*	WP_378932858.1	WP_378933101.1	WP_378931977.1
Bacillota	*Metabacillus idriensis*	WP_154319159.1	WP_224773259.1	WP_309191454.1
Bacillota	*Metabacillus indicus*	WP_411333257.1	WP_342822342.1	WP_282033509.1
Bacillota	*Metabacillus iocasae*	WP_205186994.1	WP_205187002.1	WP_205183511.1
Bacillota	*Metabacillus kandeliae*	WP_231787999.1	WP_231788118.1	WP_231788484.1
Bacillota	*Metabacillus lacus*	WP_154306098.1	WP_154308074.1	WP_154305956.1
Bacillota	*Metabacillus litoralis*	WP_226666844.1	WP_226666846.1	WP_162987286.1
Bacillota	*Metabacillus malikii*	WP_307335414.1	WP_307335411.1	WP_307341933.1
Bacillota	*Metabacillus mangrovi*	WP_155110674.1	WP_155110675.1	WP_155111176.1
Bacillota	*Metabacillus niabensis*	WP_226526663.1	WP_095302204.1	WP_174879360.1
Bacillota	*Metabacillus rhizosphaerae*	WP_338789327.1	WP_338789326.1	WP_338786668.1

Bacillota	*Metabacillus schmidteae*	WP_102229189.1	WP_175639695.1	WP_102229602.1
Bacillota	*Metabacillus sediminilitoris*	WP_136351060.1	WP_136351061.1	WP_168733743.1
Bacillota	*Metabacillus sediminis*	WP_338776765.1	WP_338776767.1	WP_338781799.1
Bacillota	*Metabacillus sp. cB07*	WP_203287618.1	WP_242524605.1	WP_203290116.1
Bacillota	*Metalysinibacillus jejuensis*	WP_108306670.1	WP_108306092.1	WP_108305698.1
Bacillota	*Metaplanococcus flavidus*	WP_144836964.1	WP_144841296.1	WP_144837543.1
Bacillota	*Metasolibacillus fluoroglycofenilyticus*	WP_107942412.1	WP_066168100.1	WP_107942236.1
Bacillota	*Metasolibacillus meyeri*	WP_326123580.1	WP_326121111.1	WP_326123935.1
Bacillota	*Metasolibacillus sp.*	WP_293921022.1	WP_293921394.1	WP_293922100.1
Bacillota	*Microaerobacter geothermalis*	WP_236405557.1	WP_407942200.1	WP_236405793.1
Bacillota	*Natranaerobius thermophilus*	WP_012447734.1	WP_012447733.1	WP_012447732.1
Bacillota	*Natranaerobius trueperi*	WP_276207008.1	WP_089023721.1	WP_089023720.1
Bacillota	*Natranaerofaba carboxydovora*	WP_241080178.1	WP_241080177.1	WP_241080176.1
Bacillota	*Natribacillus halophilus*	WP_090395622.1	WP_425433695.1	WP_090397283.1
Bacillota	*Natronobacillus azotifigens*	WP_268778642.1	WP_268780107.1	WP_268781301.1
Bacillota	*Neobacillus bataviensis*	WP_223593452.1	WP_223593454.1	WP_223595997.1
Bacillota	*Neobacillus citreus*	WP_213146114.1	WP_213145044.1	WP_213140845.1
Bacillota	*Neobacillus cucumis*	WP_328052667.1	WP_309527024.1	WP_101647346.1
Bacillota	*Neobacillus dielmonensis*	WP_042457792.1	WP_042457922.1	WP_042456158.1
Bacillota	*Neobacillus drentensis*	WP_335441998.1	WP_335441999.1	WP_335465705.1
Bacillota	*Neobacillus driksii*	WP_374931224.1	WP_307310470.1	WP_179597349.1
Bacillota	*Neobacillus endophyticus*	WP_173070301.1	WP_173070303.1	WP_173065939.1
Bacillota	*Neobacillus fumarioli*	WP_066369844.1	WP_066369846.1	WP_066368341.1
Bacillota	*Neobacillus ginsengisoli*	WP_307404320.1	WP_307404318.1	WP_307406315.1
Bacillota	*Neobacillus jeddahensis*	WP_040208386.1	WP_040208383.1	WP_040204841.1
Bacillota	*Neobacillus kokaensis*	WP_191271830.1	WP_191271828.1	WP_191276915.1
Bacillota	*Neobacillus massiliamazoniensis*	WP_090637063.1	WP_090637065.1	WP_090630295.1
Bacillota	*Neobacillus mesonae*	WP_251465883.1	WP_286232340.1	WP_066395689.1

Bacillota	*Neobacillus muris*	WP_251554148.1	WP_251554150.1	WP_251549056.1
Bacillota	*Neobacillus niacini*	WP_063251212.1	WP_335528719.1	WP_262307682.1
Bacillota	*Neobacillus notoginsengisoli*	WP_118920643.1	WP_118920642.1	WP_118922791.1
Bacillota	*Neobacillus novalis*	WP_066089240.1	WP_066089243.1	WP_066085070.1
Bacillota	*Neobacillus piezotolerans*	WP_115452360.1	WP_115452359.1	WP_115450397.1
Bacillota	*Neobacillus rhizophilus*	WP_213120381.1	WP_213120380.1	WP_213116428.1
Bacillota	*Neobacillus rhizosphaerae*	WP_248736036.1	WP_248736035.1	WP_248733686.1
Bacillota	*Neobacillus sedimentimangrovi*	WP_163183094.1	WP_038535832.1	WP_231314560.1
Bacillota	*Neobacillus soli*	WP_066061022.1	WP_066061019.1	WP_066066500.1
Bacillota	*Neobacillus sp.*	WP_312475799.1	WP_374718208.1	WP_312469188.1
Bacillota	*Neobacillus terrae*	WP_166022683.1	WP_166022681.1	WP_166023729.1
Bacillota	*Neobacillus thermocopriae*	WP_163252363.1	WP_163252362.1	WP_163250174.1
Bacillota	*Neobacillus vireti*	WP_024030504.1	WP_335567265.1	WP_335497781.1
Bacillota	*Niallia circulans*	WP_047940384.1	WP_095322317.1	WP_340461021.1
Bacillota	*Niallia endozanthoxylica*	WP_150439756.1	WP_150442538.1	WP_150439624.1
Bacillota	*Niallia nealsonii*	WP_101176769.1	WP_101176770.1	WP_101178689.1
Bacillota	*Niallia oryzisoli*	WP_338451815.1	WP_338453405.1	WP_394232620.1
Bacillota	*Niallia sp.*	WP_312096806.1	WP_312096808.1	WP_312095343.1
Bacillota	*Niallia taxi*	WP_127737632.1	WP_182104199.1	WP_182102957.1
Bacillota	*Nosocomiicoccus ampullae*	WP_219522448.1	WP_068129475.1	WP_183674126.1
Bacillota	*Nosocomiicoccus massiliensis*	WP_102167308.1	WP_040928630.1	WP_052327350.1
Bacillota	*Nosocomiicoccus sp. HMSC09A07*	WP_070456533.1	WP_070457890.1	WP_070457575.1
Bacillota	*Novibacillus thermophilus*	WP_077720594.1	WP_077718877.1	WP_077719370.1
Bacillota	*Numidum massiliense*	WP_054949644.1	WP_054949643.1	WP_054949462.1
Bacillota	*Oceanobacillus alkalisoli*	WP_235760767.1	WP_235761286.1	WP_238471747.1
Bacillota	*Oceanobacillus arenosus*	WP_115772703.1	WP_115774338.1	WP_115773638.1
Bacillota	*Oceanobacillus bengalensis*	WP_121133105.1	WP_121128214.1	WP_121130240.1
Bacillota	*Oceanobacillus chungangensis*	WP_115747896.1	WP_115751250.1	WP_115749999.1

Bacillota	*Oceanobacillus damuensis*	WP_067725496.1	WP_067727112.1	WP_067728293.1
Bacillota	*Oceanobacillus halophilus*	WP_121202721.1	WP_121203191.1	WP_121202470.1
Bacillota	*Oceanobacillus halotolerans*	WP_163969839.1	WP_163970678.1	WP_163970754.1
Bacillota	*Oceanobacillus iheyensis*	WP_011066165.1	WP_011066878.1	WP_011065861.1
Bacillota	*Oceanobacillus indicireducens*	WP_188856749.1	WP_188859558.1	WP_188855640.1
Bacillota	*Oceanobacillus jeddahense*	WP_040981361.1	WP_052400892.1	WP_256706976.1
Bacillota	*Oceanobacillus jordanicus*	WP_238018995.1	WP_106897347.1	WP_238020858.1
Bacillota	*Oceanobacillus kapialis*	WP_379564138.1	WP_404454280.1	WP_379560979.1
Bacillota	*Oceanobacillus kimchii*	WP_260048388.1	WP_272031485.1	WP_317957949.1
Bacillota	*Oceanobacillus limi*	WP_090866871.1	WP_090868480.1	WP_090869609.1
Bacillota	*Oceanobacillus locisalsi*	WP_379590821.1	WP_379593821.1	WP_379593309.1
Bacillota	*Oceanobacillus longus*	WP_379495450.1	WP_379496333.1	WP_379495717.1
Bacillota	*Oceanobacillus luteolus*	WP_251510379.1	WP_251511152.1	WP_379596990.1
Bacillota	*Oceanobacillus manasiensis*	WP_042223609.1	WP_042222495.1	WP_042223865.1
Bacillota	*Oceanobacillus massiliensis*	WP_010650627.1	WP_010647567.1	WP_337019585.1
Bacillota	*Oceanobacillus neutriphilus*	WP_188734211.1	WP_188732546.1	WP_188737378.1
Bacillota	*Oceanobacillus oncorhynchi*	WP_343768320.1	WP_193064296.1	WP_343769320.1
Bacillota	*Oceanobacillus picturae*	WP_119574346.1	WP_058950952.1	WP_379550491.1
Bacillota	*Oceanobacillus piezotolerans*	WP_121521206.1	WP_121521890.1	WP_246001176.1
Bacillota	*Oceanobacillus polygoni*	WP_149474075.1	WP_149473332.1	WP_149476200.1
Bacillota	*Oceanobacillus profundus*	WP_379601181.1	WP_251659207.1	WP_118888966.1
Bacillota	*Oceanobacillus rekensis*	WP_087971512.1	WP_087972585.1	WP_087973019.1
Bacillota	*Oceanobacillus salinisoli*	WP_156289216.1	WP_156288339.1	WP_156290435.1
Bacillota	*Oceanobacillus saliphilus*	WP_249871848.1	WP_249871298.1	WP_249869876.1
Bacillota	*Oceanobacillus senegalensis*	WP_085994422.1	WP_085991367.1	WP_085992481.1
Bacillota	*Oceanobacillus sojae*	WP_077603012.1	WP_077601946.1	WP_379545238.1
Bacillota	*Oceanobacillus sp. FSL K6-2867*	WP_339227992.1	WP_339227347.1	WP_339227598.1
Bacillota	*Oceanobacillus timonensis*	WP_080874393.1	WP_080873302.1	WP_080874844.1

Bacillota	*Oceanobacillus zhaokaii*	WP_114916620.1	WP_114917281.1	WP_114916282.1
Bacillota	*Oikeobacillus pervagus*	WP_307255661.1	WP_307255787.1	WP_307257227.1
Bacillota	*Ornithinibacillus bavariensis*	WP_212921903.1	WP_212919871.1	WP_212919548.1
Bacillota	*Ornithinibacillus californiensis*	WP_047985292.1	WP_047984527.1	WP_047984563.1
Bacillota	*Ornithinibacillus caprae*	WP_155666829.1	WP_155669075.1	WP_155669501.1
Bacillota	*Ornithinibacillus contaminans*	WP_047980060.1	WP_047980813.1	WP_047981359.1
Bacillota	*Ornithinibacillus gellani*	WP_123316431.1	WP_123316031.1	WP_123315413.1
Bacillota	*Ornithinibacillus halophilus*	WP_072887307.1	WP_072892005.1	WP_072886773.1
Bacillota	*Ornithinibacillus halotolerans*	WP_188382795.1	WP_188383526.1	WP_188385364.1
Bacillota	*Ornithinibacillus hominis*	WP_186868106.1	WP_186870584.1	WP_186868582.1
Bacillota	*Ornithinibacillus massiliensis*	WP_211741036.1	WP_211742459.1	WP_211741530.1
Bacillota	*Ornithinibacillus salinisoli*	WP_377557228.1	WP_377557623.1	WP_377555581.1
Bacillota	*Ornithinibacillus scapharcae*	WP_033445626.1	WP_010098482.1	WP_010096796.1
Bacillota	*Ornithinibacillus sp. 179-J 7C1 HS*	WP_412975632.1	WP_412977190.1	WP_412975348.1
Bacillota	*Ornithinibacillus xuwenensis*	WP_345823651.1	WP_345826023.1	WP_345823993.1
Bacillota	*Paenactinomyces guangxiensis*	WP_181751724.1	WP_309506121.1	WP_181751500.1
Bacillota	*Paenalkalicoccus suaedae*	WP_176009154.1	WP_176009153.1	WP_176009567.1
Bacillota	*Paenibacillus abyssi*	WP_188528309.1	WP_188528307.1	WP_188531779.1
Bacillota	*Paenibacillus aceris*	WP_167053849.1	WP_167053847.1	WP_167063709.1
Bacillota	*Paenibacillus aceti*	WP_120462256.1	WP_120462257.1	WP_120462650.1
Bacillota	*Paenibacillus aestuarii*	WP_270881318.1	WP_270881317.1	WP_270885492.1
Bacillota	*Paenibacillus agaridevorans*	WP_108991958.1	WP_214631096.1	WP_214629140.1
Bacillota	*Paenibacillus agilis*	WP_144988852.1	WP_144988849.1	WP_144991373.1
Bacillota	*Paenibacillus agri*	WP_175373667.1	WP_175373669.1	WP_175372874.1
Bacillota	*Paenibacillus agricola*	WP_166145632.1	WP_166145635.1	WP_311764869.1
Bacillota	*Paenibacillus alba*	WP_173215900.1	WP_173215902.1	WP_173216716.1
Bacillota	*Paenibacillus albicereus*	WP_206110059.1	WP_168907475.1	WP_168908071.1
Bacillota	*Paenibacillus albidus*	WP_215176829.1	WP_215176831.1	WP_189025022.1

Bacillota	*Paenibacillus albiflavus*	WP_132416234.1	WP_132416236.1	WP_132419223.1
Bacillota	*Paenibacillus albus*	WP_126016142.1	WP_126016140.1	WP_126015742.1
Bacillota	*Paenibacillus alginolyticus*	WP_029193046.1	WP_268615699.1	WP_029196527.1
Bacillota	*Paenibacillus algorifonticola*	WP_046232076.1	WP_046232075.1	WP_046230403.1
Bacillota	*Paenibacillus alkaliterrae*	WP_235235122.1	WP_235235115.1	WP_235238666.1
Bacillota	*Paenibacillus alkalitolerans*	WP_199614925.1	WP_199614927.1	WP_199618942.1
Bacillota	*Paenibacillus allorhizoplanae*	WP_236284611.1	WP_236285355.1	WP_236287531.1
Bacillota	*Paenibacillus allorhizosphaerae*	WP_218100601.1	WP_218100602.1	WP_218097063.1
Bacillota	*Paenibacillus alvei*	WP_197266707.1	WP_420029255.1	WP_138184889.1
Bacillota	*Paenibacillus amylolyticus*	WP_123066039.1	WP_347386738.1	WP_123062536.1
Bacillota	*Paenibacillus andongensis*	WP_261306455.1	WP_261301090.1	WP_261305761.1
Bacillota	*Paenibacillus anseongense*	WP_326060907.1	WP_326062979.1	WP_326060556.1
Bacillota	*Paenibacillus antarcticus*	WP_068648876.1	WP_068648878.1	WP_068651994.1
Bacillota	*Paenibacillus antibioticophila*	WP_212939118.1	WP_212939119.1	WP_044479289.1
Bacillota	*Paenibacillus antri*	WP_138193362.1	WP_138193361.1	WP_158299477.1
Bacillota	*Paenibacillus apiarius*	WP_087435421.1	WP_087435420.1	WP_206096386.1
Bacillota	*Paenibacillus apii*	WP_165094357.1	WP_165094360.1	WP_168130059.1
Bacillota	*Paenibacillus apis*	WP_301630174.1	WP_301627953.1	WP_301624390.1
Bacillota	*Paenibacillus aquistagni*	WP_085493644.1	WP_176228866.1	WP_085492660.1
Bacillota	*Paenibacillus arenilitoris*	WP_190861985.1	WP_190861983.1	WP_190862815.1
Bacillota	*Paenibacillus arenosi*	WP_192024005.1	WP_192024006.1	WP_192023195.1
Bacillota	*Paenibacillus artemisiicola*	WP_208847075.1	WP_240545972.1	WP_208846887.1
Bacillota	*Paenibacillus assamensis*	WP_028593560.1	WP_028593559.1	WP_028595832.1
Bacillota	*Paenibacillus athensensis*	WP_134750232.1	WP_134750231.1	WP_134757304.1
Bacillota	*Paenibacillus auburnensis*	WP_236331603.1	WP_236331605.1	WP_236332662.1
Bacillota	*Paenibacillus aurantiacus*	WP_377500029.1	WP_377500027.1	WP_377491245.1
Bacillota	*Paenibacillus aurantius*	WP_315603029.1	WP_315603030.1	WP_315607290.1
Bacillota	*Paenibacillus azoreducens*	WP_212979992.1	WP_212979993.1	WP_415998835.1

Bacillota	*Paenibacillus baekrokdamisoli*	WP_125656665.1	WP_125656668.1	WP_125657417.1
Bacillota	*Paenibacillus barcinonensis*	WP_110894264.1	WP_110894265.1	WP_216517219.1
Bacillota	*Paenibacillus barengoltzii*	WP_127576637.1	WP_016311834.1	WP_333556376.1
Bacillota	*Paenibacillus beijingensis*	WP_045672563.1	WP_045672564.1	WP_045671783.1
Bacillota	*Paenibacillus borealis*	WP_042215472.1	WP_042215475.1	WP_076109039.1
Bacillota	*Paenibacillus bouchesdurhonensis*	WP_110932522.1	WP_110932521.1	WP_110932164.1
Bacillota	*Paenibacillus bovis*	WP_060535533.1	WP_060535534.1	WP_060535797.1
Bacillota	*Paenibacillus brasilensis*	WP_152381998.1	WP_152381999.1	WP_152378823.1
Bacillota	*Paenibacillus brevis*	WP_216481004.1	WP_216481005.1	WP_216481043.1
Bacillota	*Paenibacillus camerounensis*	WP_042196114.1	WP_042204296.1	WP_042197491.1
Bacillota	*Paenibacillus castaneae*	WP_102710847.1	WP_102710846.1	WP_102712600.1
Bacillota	*Paenibacillus catalpae*	WP_091189044.1	WP_091189043.1	WP_091180719.1
Bacillota	*Paenibacillus caui*	WP_223066654.1	WP_223066691.1	WP_223066358.1
Bacillota	*Paenibacillus cellulosilyticus*	WP_110043359.1	WP_110043360.1	WP_110046471.1
Bacillota	*Paenibacillus cellulositrophicus*	WP_152398281.1	WP_152398282.1	WP_152398424.1
Bacillota	*Paenibacillus chartarius*	WP_377472238.1	WP_377472239.1	WP_377471493.1
Bacillota	*Paenibacillus chibensis*	WP_127607164.1	WP_328277582.1	WP_127601684.1
Bacillota	*Paenibacillus chitinolyticus*	WP_405079470.1	WP_387535989.1	WP_377646096.1
Bacillota	*Paenibacillus chungangensis*	WP_377562651.1	WP_377562650.1	WP_377568441.1
Bacillota	*Paenibacillus cineris*	WP_212984345.1	WP_212967858.1	WP_212967761.1
Bacillota	*Paenibacillus contaminans*	WP_113033634.1	WP_113033636.1	WP_113032225.1
Bacillota	*Paenibacillus crassostreae*	WP_068657922.1	WP_068657921.1	WP_068659503.1
Bacillota	*Paenibacillus cremeus*	WP_144842940.1	WP_261381216.1	WP_144847196.1
Bacillota	*Paenibacillus curdlanolyticus*	WP_006039229.1	WP_006039230.1	WP_006036336.1
Bacillota	*Paenibacillus cymbidii*	WP_135551466.1	WP_135551469.1	WP_135546844.1
Bacillota	*Paenibacillus daejeonensis*	WP_020616645.1	WP_020616646.1	WP_020615593.1
Bacillota	*Paenibacillus dakarensis*	WP_054957615.1	WP_054957616.1	WP_054956622.1
Bacillota	*Paenibacillus dendritiformis*	WP_111154157.1	WP_168181507.1	WP_213469088.1

Bacillota	*Paenibacillus dendrobii*	WP_202128686.1	WP_160498316.1	WP_160498813.1
Bacillota	*Paenibacillus dokdonensis*	WP_136606159.1	WP_136606160.1	WP_136606510.1
Bacillota	*Paenibacillus donghaensis*	WP_087914496.1	WP_194230145.1	WP_087917553.1
Bacillota	*Paenibacillus durus*	WP_042207282.1	WP_046723361.1	WP_046723444.1
Bacillota	*Paenibacillus ehimensis*	WP_127485603.1	WP_127485605.1	WP_025853430.1
Bacillota	*Paenibacillus elgii*	WP_010501027.1	WP_127460064.1	WP_163858210.1
Bacillota	*Paenibacillus endophyticus*	WP_183561091.1	WP_183561093.1	WP_183563669.1
Bacillota	*Paenibacillus endoradicis*	WP_258203218.1	WP_258203219.1	WP_258199419.1
Bacillota	*Paenibacillus enshidis*	WP_375353491.1	WP_375353492.1	WP_375353779.1
Bacillota	*Paenibacillus etheri*	WP_060622731.1	WP_060622730.1	WP_060622030.1
Bacillota	*Paenibacillus eucommiae*	WP_209976595.1	WP_209976594.1	WP_209974125.1
Bacillota	*Paenibacillus faecalis*	WP_106767939.1	WP_106767940.1	WP_106768318.1
Bacillota	*Paenibacillus faecis*	WP_213504414.1	WP_213504415.1	WP_148451589.1
Bacillota	*Paenibacillus farraposensis*	WP_229525501.1	WP_229525500.1	WP_229524566.1
Bacillota	*Paenibacillus favisporus*	WP_163879295.1	WP_326104187.1	WP_163879530.1
Bacillota	*Paenibacillus ferrarius*	WP_079420736.1	WP_079420735.1	WP_079420014.1
Bacillota	*Paenibacillus filicis*	WP_341414414.1	WP_341414415.1	WP_341416827.1
Bacillota	*Paenibacillus flagellatus*	WP_110839882.1	WP_110839883.1	WP_110840646.1
Bacillota	*Paenibacillus foliorum*	WP_171655782.1	WP_171655783.1	WP_312886573.1
Bacillota	*Paenibacillus fonticola*	WP_019640396.1	WP_019640395.1	WP_019636244.1
Bacillota	*Paenibacillus forsythiae*	WP_025699477.1	WP_025699479.1	WP_025697264.1
Bacillota	*Paenibacillus frigoriresistens*	WP_173184821.1	WP_173184605.1	WP_173186448.1
Bacillota	*Paenibacillus gallinarum*	WP_191799275.1	WP_191799569.1	WP_191798882.1
Bacillota	*Paenibacillus gansuensis*	WP_377601245.1	WP_377601243.1	WP_377602596.1
Bacillota	*Paenibacillus germinis*	WP_171692528.1	WP_171692346.1	WP_171688446.1
Bacillota	*Paenibacillus ginsengarvi*	WP_120750693.1	WP_120750694.1	WP_120745612.1
Bacillota	*Paenibacillus ginsengihumi*	WP_019534029.1	WP_019534030.1	WP_019533053.1
Bacillota	*Paenibacillus glacialis*	WP_068530497.1	WP_068530494.1	WP_068537660.1

Bacillota	*Paenibacillus glucanolyticus*	WP_152825198.1	WP_076152431.1	WP_340462073.1
Bacillota	*Paenibacillus glycanilyticus*	WP_251653058.1	WP_284239737.1	WP_127498518.1
Bacillota	*Paenibacillus glycinis*	WP_161740498.1	WP_161740500.1	WP_161740967.1
Bacillota	*Paenibacillus graminis*	WP_025707470.1	WP_025707469.1	WP_025708368.1
Bacillota	*Paenibacillus guangzhouensis*	WP_152393925.1	WP_152393926.1	WP_265333443.1
Bacillota	*Paenibacillus gyeongsangnamensis*	WP_269881438.1	WP_269881437.1	WP_269879583.1
Bacillota	*Paenibacillus haidiansis*	WP_331844562.1	WP_331844563.1	WP_331844893.1
Bacillota	*Paenibacillus hamazuiensis*	WP_248925463.1	WP_248925462.1	WP_248926615.1
Bacillota	*Paenibacillus harenae*	WP_307201541.1	WP_307467111.1	WP_028611235.1
Bacillota	*Paenibacillus helianthi*	WP_074109058.1	WP_074107562.1	WP_074106746.1
Bacillota	*Paenibacillus hemerocallicola*	WP_139604983.1	WP_139604984.1	WP_139602082.1
Bacillota	*Paenibacillus herberti*	WP_176444729.1	WP_089523697.1	WP_176444650.1
Bacillota	*Paenibacillus hexagrammi*	WP_235118659.1	WP_235122911.1	WP_235121698.1
Bacillota	*Paenibacillus hodogayensis*	WP_344908541.1	WP_344908543.1	WP_344907027.1
Bacillota	*Paenibacillus humicola*	WP_274651616.1	WP_274651617.1	WP_274651867.1
Bacillota	*Paenibacillus hunanensis*	WP_188775443.1	WP_320544467.1	WP_320544258.1
Bacillota	*Paenibacillus ihbetae*	WP_077565455.1	WP_099476792.1	WP_077565014.1
Bacillota	*Paenibacillus ihuae*	WP_054941331.1	WP_054941332.1	WP_054941758.1
Bacillota	*Paenibacillus ihumii*	WP_055107795.1	WP_055110100.1	WP_055107462.1
Bacillota	*Paenibacillus illinoisensis*	WP_390582895.1	WP_337033012.1	WP_127537776.1
Bacillota	*Paenibacillus jilunlii*	WP_062519683.1	WP_062519685.1	WP_062527005.1
Bacillota	*Paenibacillus kobensis*	WP_127530721.1	WP_127530722.1	WP_127533050.1
Bacillota	*Paenibacillus koleovorans*	WP_127588578.1	WP_127588579.1	WP_127582757.1
Bacillota	*Paenibacillus kribbensis*	WP_179032748.1	WP_179032749.1	WP_068502150.1
Bacillota	*Paenibacillus lacisoli*	WP_305022224.1	WP_305022225.1	WP_305022581.1
Bacillota	*Paenibacillus larvae*	WP_268570009.1	WP_023482678.1	WP_079940202.1
Bacillota	*Paenibacillus lautus*	WP_127594125.1	WP_216530988.1	WP_220686041.1
Bacillota	*Paenibacillus lemnae*	WP_169506788.1	WP_169506789.1	WP_169503480.1

Bacillota	*Paenibacillus lentus*	WP_125083238.1	WP_379392405.1	WP_379392043.1
Bacillota	*Paenibacillus lignilyticus*	WP_210659859.1	WP_210659857.1	WP_210659384.1
Bacillota	*Paenibacillus lupini*	WP_167273182.1	WP_167273178.1	WP_167277696.1
Bacillota	*Paenibacillus luteus*	WP_141501475.1	WP_141501476.1	WP_141505818.1
Bacillota	*Paenibacillus lutimineralis*	WP_126997891.1	WP_126997889.1	WP_126997225.1
Bacillota	*Paenibacillus lutrae*	WP_157335440.1	WP_157335441.1	WP_157334343.1
Bacillota	*Paenibacillus lycopersici*	WP_162356891.1	WP_162356889.1	WP_162356475.1
Bacillota	*Paenibacillus macerans*	WP_251586340.1	WP_316560952.1	WP_036621380.1
Bacillota	*Paenibacillus macquariensis*	WP_068590251.1	WP_068590249.1	WP_068587569.1
Bacillota	*Paenibacillus mangrovi*	WP_244721110.1	WP_244721114.1	WP_244722015.1
Bacillota	*Paenibacillus marchantiophytorum*	WP_189009728.1	WP_189014345.1	WP_189006243.1
Bacillota	*Paenibacillus massiliensis*	WP_028589105.1	WP_028589104.1	WP_018886771.1
Bacillota	*Paenibacillus maysiensis*	WP_025685875.1	WP_025685876.1	WP_025685742.1
Bacillota	*Paenibacillus medicaginis*	WP_375521638.1	WP_375521639.1	WP_375518551.1
Bacillota	*Paenibacillus mellifer*	WP_248550581.1	WP_248550580.1	WP_248551931.1
Bacillota	*Paenibacillus mendelii*	WP_204816264.1	WP_204816265.1	WP_204816513.1
Bacillota	*Paenibacillus mesophilus*	WP_138878350.1	WP_138878351.1	WP_138882220.1
Bacillota	*Paenibacillus mesotrionivorans*	WP_416214544.1	WP_416214545.1	WP_416219599.1
Bacillota	*Paenibacillus methanolicus*	WP_148928224.1	WP_148928225.1	WP_148928442.1
Bacillota	*Paenibacillus montanisoli*	WP_112882578.1	WP_112882579.1	WP_112882883.1
Bacillota	*Paenibacillus montaniterrae*	WP_213513802.1	WP_213513264.1	WP_246563468.1
Bacillota	*Paenibacillus monticola*	WP_154118393.1	WP_154118394.1	WP_154121344.1
Bacillota	*Paenibacillus motobuensis*	WP_343863633.1	WP_343863631.1	WP_343865702.1
Bacillota	*Paenibacillus mucilaginosus*	WP_013916540.1	WP_014370053.1	WP_236628849.1
Bacillota	*Paenibacillus nanensis*	WP_119599127.1	WP_119599126.1	WP_119598178.1
Bacillota	*Paenibacillus naphthalenovorans*	WP_175472024.1	WP_074729390.1	WP_074727048.1
Bacillota	*Paenibacillus nasutitermitis*	WP_188992611.1	WP_188992612.1	WP_188992857.1
Bacillota	*Paenibacillus nicotianae*	WP_204823540.1	WP_204823539.1	WP_204823265.1

Bacillota	*Paenibacillus oceani*	WP_190925107.1	WP_190925109.1	WP_190926136.1
Bacillota	*Paenibacillus odorifer*	WP_076284576.1	WP_248548496.1	WP_111505257.1
Bacillota	*Paenibacillus oenotherae*	WP_219870643.1	WP_219870644.1	WP_219870874.1
Bacillota	*Paenibacillus oleatilyticus*	WP_216792181.1	WP_373949330.1	WP_373955658.1
Bacillota	*Paenibacillus oralis*	WP_128634089.1	WP_128634090.1	WP_128634460.1
Bacillota	*Paenibacillus oryzae*	WP_068683840.1	WP_068683842.1	WP_068684506.1
Bacillota	*Paenibacillus oryzisoli*	WP_068670283.1	WP_068670284.1	WP_068670715.1
Bacillota	*Paenibacillus ottowii*	WP_064796416.1	WP_326400406.1	WP_305164649.1
Bacillota	*Paenibacillus pabuli*	WP_247898189.1	WP_426251136.1	WP_062326419.1
Bacillota	*Paenibacillus paeoniae*	WP_116044696.1	WP_116044698.1	WP_240644312.1
Bacillota	*Paenibacillus paridis*	WP_139999520.1	WP_139999522.1	WP_139997909.1
Bacillota	*Paenibacillus pasadenensis*	WP_251560931.1	WP_251560930.1	WP_101809358.1
Bacillota	*Paenibacillus pectinilyticus*	WP_065857228.1	WP_065857226.1	WP_244163060.1
Bacillota	*Paenibacillus pedocola*	WP_310829302.1	WP_310829303.1	WP_310829725.1
Bacillota	*Paenibacillus peoriae*	WP_010345798.1	WP_076293050.1	WP_348623108.1
Bacillota	*Paenibacillus periandrae*	WP_240413846.1	WP_240413847.1	WP_240417094.1
Bacillota	*Paenibacillus phocaensis*	WP_068785288.1	WP_068785287.1	WP_068784959.1
Bacillota	*Paenibacillus phyllosphaerae*	WP_183598253.1	WP_183598251.1	WP_246427471.1
Bacillota	*Paenibacillus physcomitrellae*	WP_094093303.1	WP_094093304.1	WP_094093583.1
Bacillota	*Paenibacillus phytohabitans*	WP_171716664.1	WP_171716665.1	WP_171721012.1
Bacillota	*Paenibacillus phytorum*	WP_171643358.1	WP_171643359.1	WP_171648381.1
Bacillota	*Paenibacillus pinihumi*	WP_028561258.1	WP_028561259.1	WP_028561628.1
Bacillota	*Paenibacillus pinisoli*	WP_120113277.1	WP_424452460.1	WP_243643891.1
Bacillota	*Paenibacillus pinistramenti*	WP_138492936.1	WP_138492937.1	WP_138493228.1
Bacillota	*Paenibacillus piri*	WP_133225913.1	WP_133225912.1	WP_342774573.1
Bacillota	*Paenibacillus piscarius*	WP_238652982.1	WP_238652983.1	WP_238654633.1
Bacillota	*Paenibacillus plantarum*	WP_171628794.1	WP_171628795.1	WP_171635300.1
Bacillota	*Paenibacillus planticolens*	WP_171682055.1	WP_171682056.1	WP_246294449.1

Bacillota	*Paenibacillus plantiphilus*	WP_236344839.1	WP_371877750.1	WP_236344089.1
Bacillota	*Paenibacillus polymyxa*	WP_311078506.1	WP_215076021.1	WP_075154454.1
Bacillota	*Paenibacillus polysaccharolyticus*	WP_251505656.1	WP_251505659.1	WP_253492605.1
Bacillota	*Paenibacillus popilliae*	WP_006285465.1	WP_142543324.1	WP_142543523.1
Bacillota	*Paenibacillus prosopidis*	WP_181873345.1	WP_114378576.1	WP_114381166.1
Bacillota	*Paenibacillus protaetiae*	WP_129439405.1	WP_129439407.1	WP_129444004.1
Bacillota	*Paenibacillus pseudetheri*	WP_234531948.1	WP_234531942.1	WP_234533213.1
Bacillota	*Paenibacillus psychroresistens*	WP_155701927.1	WP_155701928.1	WP_155700801.1
Bacillota	*Paenibacillus puerhi*	WP_159883059.1	WP_159883057.1	WP_235941410.1
Bacillota	*Paenibacillus puldeungensis*	WP_379321196.1	WP_379321195.1	WP_379318034.1
Bacillota	*Paenibacillus qinlingensis*	WP_310223539.1	WP_173110235.1	WP_310226997.1
Bacillota	*Paenibacillus radicibacter*	WP_258206429.1	WP_258206430.1	WP_258206078.1
Bacillota	*Paenibacillus radicis (ex Gao et al. 2016)*	WP_188887732.1	WP_188887731.1	WP_229692423.1
Bacillota	*Paenibacillus rhizophilus*	WP_124693619.1	WP_124693618.1	WP_124695680.1
Bacillota	*Paenibacillus rhizoplanae*	WP_209994246.1	WP_209993887.1	WP_209987956.1
Bacillota	*Paenibacillus rhizosphaerae*	WP_183581975.1	WP_183581974.1	WP_183581722.1
Bacillota	*Paenibacillus rhizovicinus*	WP_162638887.1	WP_162638888.1	WP_162639117.1
Bacillota	*Paenibacillus rigui*	WP_094013285.1	WP_094013286.1	WP_094015209.1
Bacillota	*Paenibacillus riograndensis*	WP_020427657.1	WP_020427658.1	WP_020427852.1
Bacillota	*Paenibacillus sabinae*	WP_025335550.1	WP_025335551.1	WP_025335875.1
Bacillota	*Paenibacillus sabuli*	WP_190916452.1	WP_190916454.1	WP_190915075.1
Bacillota	*Paenibacillus sacheonensis*	WP_161696576.1	WP_161696574.1	WP_161703033.1
Bacillota	*Paenibacillus sambharensis*	WP_111149231.1	WP_111149230.1	WP_111148711.1
Bacillota	*Paenibacillus sanguinis*	WP_018751371.1	WP_018751372.1	WP_018753264.1
Bacillota	*Paenibacillus sedimenti*	WP_188174342.1	WP_188174343.1	WP_188172947.1
Bacillota	*Paenibacillus sediminis*	WP_209845359.1	WP_209845357.1	WP_209844695.1
Bacillota	*Paenibacillus segetis*	WP_188538934.1	WP_188538936.1	WP_188539579.1
Bacillota	*Paenibacillus selenitireducens*	WP_078497211.1	WP_078497210.1	WP_078498745.1

Bacillota	*Paenibacillus senegalensis*	WP_026021375.1	WP_010273795.1	WP_010270149.1
Bacillota	*Paenibacillus senegalimassiliensis*	WP_059052566.1	WP_059052564.1	WP_059052170.1
Bacillota	*Paenibacillus septentrionalis*	WP_379233282.1	WP_379232422.1	WP_379230547.1
Bacillota	*Paenibacillus sepulcri*	WP_210041358.1	WP_210041361.1	WP_210037664.1
Bacillota	*Paenibacillus shirakamiensis*	WP_209858989.1	WP_209858987.1	WP_209858427.1
Bacillota	*Paenibacillus silagei*	WP_209874571.1	WP_209869954.1	WP_209871997.1
Bacillota	*Paenibacillus silvae*	WP_111271384.1	WP_188591051.1	WP_111271909.1
Bacillota	*Paenibacillus silvestris*	WP_161408517.1	WP_161408564.1	WP_235959055.1
Bacillota	*Paenibacillus silvisoli*	WP_308639168.1	WP_308639167.1	WP_308638903.1
Bacillota	*Paenibacillus silviterrae*	WP_284643950.1	WP_284640174.1	WP_284638745.1
Bacillota	*Paenibacillus sinopodophylli*	WP_138750981.1	WP_138750980.1	WP_138751928.1
Bacillota	*Paenibacillus solanacearum*	WP_218091182.1	WP_218091183.1	WP_218090647.1
Bacillota	*Paenibacillus solani*	WP_054401724.1	WP_054401723.1	WP_054401438.1
Bacillota	*Paenibacillus solisilvae*	WP_379189360.1	WP_379189361.1	WP_379190327.1
Bacillota	*Paenibacillus sonchi*	WP_233183868.1	WP_039835064.1	WP_233184314.1
Bacillota	*Paenibacillus sophorae*	WP_036594357.1	WP_036594356.1	WP_036596753.1
Bacillota	*Paenibacillus soyae*	WP_257442904.1	WP_257442906.1	WP_257444241.1
Bacillota	*Paenibacillus sp.*	WP_325352875.1	WP_313639716.1	WP_313642091.1
Bacillota	*Paenibacillus spongiae*	WP_258388478.1	WP_258388477.1	WP_258388242.1
Bacillota	*Paenibacillus stellifer*	WP_038697001.1	WP_038697002.1	WP_038697658.1
Bacillota	*Paenibacillus swuensis*	WP_068609185.1	WP_068609188.1	WP_231891263.1
Bacillota	*Paenibacillus taichungensis*	WP_413372314.1	WP_376037005.1	WP_175383163.1
Bacillota	*Paenibacillus taihuensis*	WP_116187053.1	WP_116187052.1	WP_116187460.1
Bacillota	*Paenibacillus taiwanensis*	WP_028544285.1	WP_028544284.1	WP_245596021.1
Bacillota	*Paenibacillus tarimensis*	WP_235285858.1	WP_235285857.1	WP_235285404.1
Bacillota	*Paenibacillus tengchongensis*	WP_151734500.1	WP_151734499.1	WP_151735851.1
Bacillota	*Paenibacillus tepidiphilus*	WP_150273581.1	WP_150273580.1	WP_150266292.1
Bacillota	*Paenibacillus terrae*	WP_014282065.1	WP_145158499.1	WP_014282450.1

Bacillota	*Paenibacillus terreus*	WP_375523607.1	WP_375523606.1	WP_375526893.1
Bacillota	*Paenibacillus terricola*	WP_191203123.1	WP_191203122.1	WP_191202242.1
Bacillota	*Paenibacillus terrigena*	WP_018755349.1	WP_314585441.1	WP_018756691.1
Bacillota	*Paenibacillus thailandensis*	WP_379275504.1	WP_379275508.1	WP_379273623.1
Bacillota	*Paenibacillus thalictri*	WP_131012956.1	WP_131012957.1	WP_131014125.1
Bacillota	*Paenibacillus thermoaerophilus*	WP_138787912.1	WP_138787913.1	WP_170209381.1
Bacillota	*Paenibacillus thermotolerans*	WP_274364624.1	WP_274364693.1	WP_274363310.1
Bacillota	*Paenibacillus thiaminolyticus*	WP_143800797.1	WP_374018712.1	WP_087442146.1
Bacillota	*Paenibacillus tianjinensis*	WP_206100817.1	WP_206100818.1	WP_206101483.1
Bacillota	*Paenibacillus tianmuensis*	WP_090665888.1	WP_090665886.1	WP_090670061.1
Bacillota	*Paenibacillus timonensis*	WP_240268406.1	WP_240268405.1	WP_240269054.1
Bacillota	*Paenibacillus tritici*	WP_173129013.1	WP_173129016.1	WP_211720737.1
Bacillota	*Paenibacillus tundrae*	WP_307212880.1	WP_307212877.1	WP_338540183.1
Bacillota	*Paenibacillus turicensis*	WP_210091285.1	WP_210091286.1	WP_210088043.1
Bacillota	*Paenibacillus turpanensis*	WP_166239436.1	WP_166239434.1	WP_166243811.1
Bacillota	*Paenibacillus typhae*	WP_090714162.1	WP_221798010.1	WP_090712285.1
Bacillota	*Paenibacillus tyrfis*	WP_253409611.1	WP_088833390.1	WP_281940216.1
Bacillota	*Paenibacillus uliginis*	WP_208914519.1	WP_208914520.1	WP_208915055.1
Bacillota	*Paenibacillus vandeheii*	WP_301246512.1	WP_301246510.1	WP_301243803.1
Bacillota	*Paenibacillus vietnamensis*	WP_224722471.1	WP_224722472.1	WP_224721890.1
Bacillota	*Paenibacillus vini*	WP_213654996.1	WP_213654995.1	WP_213653843.1
Bacillota	*Paenibacillus wenxiniae*	WP_347325917.1	WP_347325918.1	WP_347325656.1
Bacillota	*Paenibacillus whitsoniae*	WP_126140510.1	WP_126140511.1	WP_126143135.1
Bacillota	*Paenibacillus woosongensis*	WP_155610100.1	WP_283924922.1	WP_155610841.1
Bacillota	*Paenibacillus wulumuqiensis*	WP_046215039.1	WP_046215038.1	WP_046214747.1
Bacillota	*Paenibacillus wynnii*	WP_036657105.1	WP_307586656.1	WP_036657888.1
Bacillota	*Paenibacillus xanthanilyticus*	WP_377722267.1	WP_377722268.1	WP_377722477.1
Bacillota	*Paenibacillus xerothermodurans*	WP_089199061.1	WP_089199062.1	WP_243633136.1

Bacillota	*Paenibacillus xylanexedens*	WP_419889253.1	WP_124116226.1	WP_145332067.1
Bacillota	*Paenibacillus xylaniclasticus*	WP_127566875.1	WP_127566877.1	WP_127567498.1
Bacillota	*Paenibacillus xylanilyticus*	WP_418038329.1	WP_413035371.1	WP_175397529.1
Bacillota	*Paenibacillus xylanivorans*	WP_053780711.1	WP_053780710.1	WP_053781229.1
Bacillota	*Paenibacillus yanchengensis*	WP_377771597.1	WP_377772049.1	WP_377772207.1
Bacillota	*Paenibacillus yonginensis*	WP_068695883.1	WP_068695885.1	WP_068696453.1
Bacillota	*Paenibacillus zanthoxyli*	WP_025692677.1	WP_025692678.1	WP_025691633.1
Bacillota	*Paenibacillus zeirhizosphaerae*	WP_305754248.1	WP_305754249.1	WP_305754549.1
Bacillota	*Paenibacillus zeisoli*	WP_127197751.1	WP_127197752.1	WP_127198081.1
Bacillota	*Paenisporosarcina antarctica*	WP_134209959.1	WP_134210720.1	WP_134210153.1
Bacillota	*Paenisporosarcina cavernae*	WP_119883146.1	WP_119882510.1	WP_119882894.1
Bacillota	*Paenisporosarcina indica*	WP_075617693.1	WP_075618437.1	WP_075617467.1
Bacillota	*Paenisporosarcina macmurdoensis*	WP_377732563.1	WP_377735773.1	WP_377732245.1
Bacillota	*Paenisporosarcina quisquiliarum*	WP_394191999.1	WP_269927160.1	WP_394187284.1
Bacillota	*Paenisporosarcina sp.*	WP_424473098.1	WP_424472036.1	WP_424415549.1
Bacillota	*Pallidibacillus pasinlerensis*	WP_161919234.1	WP_161920773.1	WP_161919021.1
Bacillota	*Pallidibacillus thermolactis*	WP_173660149.1	WP_263061301.1	WP_173662503.1
Bacillota	*Paludifilum halophilum*	WP_094262789.1	WP_094262790.1	WP_240511973.1
Bacillota	*Parageobacillus genomosp. 1*	WP_043905239.1	WP_043905240.1	WP_043904021.1
Bacillota	*Parageobacillus sp. VR-IP*	WP_175245149.1	WP_175245148.1	WP_175243359.1
Bacillota	*Parageobacillus thermantarcticus*	WP_090948791.1	WP_208601770.1	WP_090948889.1
Bacillota	*Parageobacillus thermoglucosidasius*	WP_003249633.1	WP_064550242.1	WP_064551126.1
Bacillota	*Parageobacillus toebii*	WP_205424629.1	WP_374718483.1	WP_374720214.1
Bacillota	*Paraliobacillus quinghaiensis*	WP_117152167.1	WP_117155941.1	WP_117153002.1
Bacillota	*Paraliobacillus ryukyuensis*	WP_079709683.1	WP_113868836.1	WP_079709353.1
Bacillota	*Paraliobacillus salinarum*	WP_182199625.1	WP_182201226.1	WP_182199062.1
Bacillota	*Paraliobacillus sediminis*	WP_117169373.1	WP_117168819.1	WP_117169811.1
Bacillota	*Paraliobacillus sp. JSM ZJ581*	WP_374065147.1	WP_374067258.1	WP_374067853.1

Bacillota	*Paranoxybacillus vitaminiphilus*	WP_111643996.1	WP_111643997.1	WP_111643621.1
Bacillota	*Paucisalibacillus globulus*	WP_096270445.1	WP_026909002.1	WP_096269978.1
Bacillota	*Paucisalibacillus sp. EB02*	WP_042145630.1	WP_042142703.1	WP_042145019.1
Bacillota	*Peribacillus acanthi*	WP_108671147.1	WP_108671146.1	WP_108669794.1
Bacillota	*Peribacillus alkalitolerans*	WP_163100974.1	WP_163100975.1	WP_163101404.1
Bacillota	*Peribacillus asahii*	WP_127761156.1	WP_252286581.1	WP_252289885.1
Bacillota	*Peribacillus butanolivorans*	WP_328114255.1	WP_402878124.1	WP_053345228.1
Bacillota	*Peribacillus castrilensis*	WP_367409646.1	WP_367407233.1	WP_367407051.1
Bacillota	*Peribacillus cavernae*	WP_126864739.1	WP_126863591.1	WP_126864368.1
Bacillota	*Peribacillus deserti*	WP_101645019.1	WP_204542463.1	WP_204541793.1
Bacillota	*Peribacillus faecalis*	WP_190999950.1	WP_190999949.1	WP_190996378.1
Bacillota	*Peribacillus frigoritolerans*	WP_406593472.1	WP_260358989.1	WP_289352261.1
Bacillota	*Peribacillus glennii*	WP_117321694.1	WP_233522557.1	WP_117322745.1
Bacillota	*Peribacillus huizhouensis*	WP_182503146.1	WP_182503147.1	WP_182501307.1
Bacillota	*Peribacillus kribbensis*	WP_026694847.1	WP_155890147.1	WP_026692131.1
Bacillota	*Peribacillus loiseleuriae*	WP_049681938.1	WP_049681939.1	WP_049680679.1
Bacillota	*Peribacillus muralis*	WP_064463009.1	WP_377363119.1	WP_427674549.1
Bacillota	*Peribacillus psychrosaccharolyticus*	WP_040376265.1	WP_040376266.1	WP_040374614.1
Bacillota	*Peribacillus saganii*	WP_199478491.1	WP_117327000.1	WP_117325393.1
Bacillota	*Peribacillus simplex*	WP_347939832.1	WP_352920322.1	WP_144527054.1
Bacillota	*Peribacillus sp. NJ11*	WP_289334827.1	WP_289334826.1	WP_289335970.1
Bacillota	*Peribacillus tepidiphilus*	WP_374722489.1	WP_374722488.1	WP_153124242.1
Bacillota	*Perspicuibacillus lycopersici*	WP_263072930.1	WP_263072726.1	WP_263071344.1
Bacillota	*Phocicoccus pinnipedialis*	WP_186077510.1	WP_377275301.1	WP_186078057.1
Bacillota	*Phocicoccus schoeneichii*	WP_186087927.1	WP_186084444.1	WP_186086908.1
Bacillota	*Phosphitispora fastidiosa*	WP_231685226.1	WP_231685227.1	WP_231685477.1
Bacillota	*Piscibacillus halophilus*	WP_091772403.1	WP_175614577.1	WP_091771979.1
Bacillota	*Piscibacillus salipiscarius*	WP_377330544.1	WP_054751699.1	WP_377327791.1

Bacillota	*Planifilum fimeticola*	WP_106345631.1	WP_106345581.1	WP_106344368.1
Bacillota	*Planifilum fulgidum*	WP_092035968.1	WP_092035967.1	WP_092041673.1
Bacillota	*Planococcus antarcticus*	WP_040852325.1	WP_006829302.1	WP_006828840.1
Bacillota	*Planococcus chinensis*	WP_224076780.1	WP_224076704.1	WP_204892578.1
Bacillota	*Planococcus dechangensis*	WP_377277442.1	WP_377278760.1	WP_377277778.1
Bacillota	*Planococcus donghaensis*	WP_394121751.1	WP_394121384.1	WP_065525910.1
Bacillota	*Planococcus glaciei*	WP_200904932.1	WP_036802959.1	WP_053166399.1
Bacillota	*Planococcus halocryophilus*	WP_240747830.1	WP_008496839.1	WP_008498021.1
Bacillota	*Planococcus halotolerans*	WP_112222673.1	WP_135815217.1	WP_233785852.1
Bacillota	*Planococcus kocurii*	WP_058385059.1	WP_058384407.1	WP_058384864.1
Bacillota	*Planococcus lenghuensis*	WP_077589509.1	WP_077590103.1	WP_077589700.1
Bacillota	*Planococcus liqunii*	WP_300981076.1	WP_301725568.1	WP_300981278.1
Bacillota	*Planococcus maitriensis*	WP_205853838.1	WP_112230374.1	WP_112231356.1
Bacillota	*Planococcus maritimus*	WP_068465707.1	WP_068464323.1	WP_068486073.1
Bacillota	*Planococcus notacanthi*	WP_290214263.1	WP_290214969.1	WP_290214207.1
Bacillota	*Planococcus plakortidis*	WP_411361501.1	WP_405313062.1	WP_411361557.1
Bacillota	*Planococcus salinarum*	WP_117303781.1	WP_071151755.1	WP_117301439.1
Bacillota	*Planococcus salinus*	WP_192892912.1	WP_123164587.1	WP_123164382.1
Bacillota	*Planococcus shenhongbingii*	WP_301856043.1	WP_300990175.1	WP_301854711.1
Bacillota	*Planococcus shixiaomingii*	WP_301722914.1	WP_300985885.1	WP_300985285.1
Bacillota	*Planococcus soli*	WP_142826438.1	WP_142827080.1	WP_142826638.1
Bacillota	*Planococcus sp. CAU13*	WP_033541215.1	WP_033541336.1	WP_033543526.1
Bacillota	*Planococcus versutus*	WP_065524488.1	WP_049692979.1	WP_049693354.1
Bacillota	*Planomicrobium okeanokoites*	WP_117312365.1	WP_117313702.1	WP_084244977.1
Bacillota	*Planomicrobium soli*	WP_106531759.1	WP_106532793.1	WP_106534878.1
Bacillota	*Planomicrobium sp. Y74*	WP_121632147.1	WP_121635614.1	WP_121633166.1
Bacillota	*Planomicrobium stackebrandtii*	WP_308785862.1	WP_308786454.1	WP_308786093.1
Bacillota	*Polycladomyces abyssicola*	WP_212772256.1	WP_212772255.1	WP_212772617.1

Bacillota	*Polycladomyces subterraneus*	WP_301237983.1	WP_301237984.1	WP_301239880.1
Bacillota	*Polycladomyces zharkentensis*	WP_205494863.1	WP_302104637.1	WP_205492524.1
Bacillota	*Polycladospora coralii*	WP_347239741.1	WP_191138248.1	WP_191140021.1
Bacillota	*Pontibacillus chungwhensis*	WP_036779359.1	WP_036782886.1	WP_036778420.1
Bacillota	*Pontibacillus halophilus*	WP_026799494.1	WP_026800003.1	WP_026799673.1
Bacillota	*Pontibacillus litoralis*	WP_036832895.1	WP_036834667.1	WP_084600106.1
Bacillota	*Pontibacillus marinus*	WP_027447180.1	WP_027445858.1	WP_027448187.1
Bacillota	*Pontibacillus salicampi*	WP_377345715.1	WP_377351052.1	WP_377347591.1
Bacillota	*Pontibacillus salipaludis*	WP_188650249.1	WP_188656141.1	WP_345238777.1
Bacillota	*Pontibacillus sp. HMF3514*	WP_160101657.1	WP_160103686.1	WP_160100870.1
Bacillota	*Pontibacillus yanchengensis*	WP_036818586.1	WP_160848860.1	WP_036820012.1
Bacillota	*Pradoshia eiseniae*	WP_104848023.1	WP_104848022.1	WP_104849624.1
Bacillota	*Priestia abyssalis*	WP_078409368.1	WP_078409367.1	WP_078413473.1
Bacillota	*Priestia aryabhattai*	WP_216142501.1	WP_226555819.1	WP_048019823.1
Bacillota	*Priestia endophytica*	WP_268582576.1	WP_111925402.1	WP_113749129.1
Bacillota	*Priestia filamentosa*	WP_243513722.1	WP_120046138.1	WP_019391719.1
Bacillota	*Priestia flexa*	WP_277716739.1	WP_281998771.1	WP_210609843.1
Bacillota	*Priestia koreensis*	WP_367018079.1	WP_336882656.1	WP_053403380.1
Bacillota	*Priestia megaterium*	WP_289522846.1	WP_414184424.1	WP_097824533.1
Bacillota	*Priestia sp. 179-F W1.4 NHS*	WP_406900744.1	WP_406901522.1	WP_406900855.1
Bacillota	*Priestia taiwanensis*	WP_188386729.1	WP_188386728.1	WP_188387811.1
Bacillota	*Pseudalkalibacillus berkeleyi*	WP_236333256.1	WP_236333258.1	WP_236332239.1
Bacillota	*Pseudalkalibacillus caeni*	WP_138128327.1	WP_138128328.1	WP_138129213.1
Bacillota	*Pseudalkalibacillus decolorationis*	WP_257351784.1	WP_257351783.1	WP_257350563.1
Bacillota	*Pseudalkalibacillus hwajinpoensis*	WP_136947247.1	WP_224880743.1	WP_347550457.1
Bacillota	*Pseudalkalibacillus salsuginis*	WP_236310908.1	WP_236310910.1	WP_236313441.1
Bacillota	*Pseudalkalibacillus sedimenti*	WP_273832483.1	WP_273835075.1	WP_273834772.1
Bacillota	*Pseudalkalibacillus sp. A8*	WP_408006508.1	WP_408006509.1	WP_408010883.1

Bacillota	*Pseudalkalibacillus spartinae*	WP_273852285.1	WP_273852284.1	WP_273850317.1
Bacillota	*Pseudogracilibacillus auburnensis*	WP_110394591.1	WP_110394227.1	WP_207520801.1
Bacillota	*Pseudogracilibacillus sp. SO10305*	WP_336265594.1	WP_336265976.1	WP_336265821.1
Bacillota	*Pseudoneobacillus rhizosphaerae*	WP_230496521.1	WP_230496522.1	WP_230495772.1
Bacillota	*Psychrobacillus antarcticus*	WP_277585418.1	WP_277586887.1	WP_277585738.1
Bacillota	*Psychrobacillus insolitus*	WP_111438313.1	WP_111439258.1	WP_170122323.1
Bacillota	*Psychrobacillus lasiicapitis*	WP_142537194.1	WP_142538007.1	WP_170206445.1
Bacillota	*Psychrobacillus mangrovi*	WP_336495936.1	WP_336496523.1	WP_336498714.1
Bacillota	*Psychrobacillus psychrodurans*	WP_269920920.1	WP_269920784.1	WP_427138498.1
Bacillota	*Psychrobacillus psychrotolerans*	WP_350473437.1	WP_422194696.1	WP_350472967.1
Bacillota	*Psychrobacillus soli*	WP_142607215.1	WP_142608489.1	WP_185907990.1
Bacillota	*Psychrobacillus sp.*	WP_313890972.1	WP_313893284.1	WP_313891380.1
Bacillota	*Psychrobacillus vulpis*	WP_142642666.1	WP_142640994.1	WP_166462473.1
Bacillota	*Pueribacillus sp. YX66*	WP_379668435.1	WP_379668182.1	WP_379668012.1
Bacillota	*Pueribacillus theae*	WP_116553981.1	WP_116554940.1	WP_116552904.1
Bacillota	*Pullulanibacillus camelliae*	WP_188692193.1	WP_188692495.1	WP_188692993.1
Bacillota	*Pullulanibacillus pueri*	WP_188496588.1	WP_188496589.1	WP_229745355.1
Bacillota	*Pullulanibacillus sp. KACC 23026*	WP_275280749.1	WP_275280751.1	WP_275281084.1
Bacillota	*Radiobacillus deserti*	WP_143894017.1	WP_143895601.1	WP_143893315.1
Bacillota	*Radiobacillus kanasensis*	WP_231097091.1	WP_231094100.1	WP_231096654.1
Bacillota	*Radiobacillus sp. PE A8.2*	WP_407268678.1	WP_407270775.1	WP_407268083.1
Bacillota	*Risungbinella massiliensis*	WP_044640190.1	WP_044640263.1	WP_044642465.1
Bacillota	*Robertmurraya andreesenii*	WP_307149767.1	WP_307149769.1	WP_307150434.1
Bacillota	*Robertmurraya korlensis*	WP_251514695.1	WP_251514693.1	WP_251515900.1
Bacillota	*Robertmurraya kyonggiensis*	WP_136830600.1	WP_136830601.1	WP_136829269.1
Bacillota	*Robertmurraya mangrovi*	WP_322447533.1	WP_322447534.1	WP_322444982.1
Bacillota	*Robertmurraya massiliosenegalensis*	WP_019156012.1	WP_019156013.1	WP_019152893.1
Bacillota	*Robertmurraya sp.*	WP_391559015.1	WP_391559014.1	WP_119707447.1

Bacillota	*Robertmurraya yapensis*	WP_126405731.1	WP_126409130.1	WP_126408518.1
Bacillota	*Rossellomorea aquimaris*	WP_064091048.1	WP_226672924.1	WP_226674332.1
Bacillota	*Rossellomorea arthrocnemi*	WP_201713300.1	WP_201713301.1	WP_230980984.1
Bacillota	*Rossellomorea marisflavi*	WP_290032330.1	WP_079516756.1	WP_063191188.1
Bacillota	*Rossellomorea oryzaecorticis*	WP_341983163.1	WP_341983161.1	WP_341982479.1
Bacillota	*Rossellomorea sp. NPDC077527*	WP_397549110.1	WP_397550578.1	WP_397549808.1
Bacillota	*Rossellomorea vietnamensis*	WP_406686849.1	WP_426898446.1	WP_426895544.1
Bacillota	*Rubeoparvulum massiliense*	WP_048601877.1	WP_315969544.1	WP_048602591.1
Bacillota	*Rummeliibacillus pycnus*	WP_102692493.1	WP_397536986.1	WP_102692757.1
Bacillota	*Rummeliibacillus sp. TYF-LIM-RU47*	WP_150283994.1	WP_150283419.1	WP_150283850.1
Bacillota	*Rummeliibacillus stabekisii*	WP_066786104.1	WP_392995461.1	WP_251645578.1
Bacillota	*Rummeliibacillus suwonensis*	WP_146549179.1	WP_146550224.1	WP_146549454.1
Bacillota	*Saccharibacillus alkalitolerans*	WP_166272347.1	WP_166272345.1	WP_166271645.1
Bacillota	*Saccharibacillus deserti*	WP_172250470.1	WP_172250468.1	WP_172249784.1
Bacillota	*Saccharibacillus endophyticus*	WP_172239876.1	WP_172239879.1	WP_172247082.1
Bacillota	*Saccharibacillus kuerlensis*	WP_018976427.1	WP_018976426.1	WP_018975684.1
Bacillota	*Saccharibacillus qingshengii*	WP_172196256.1	WP_172196255.1	WP_172194137.1
Bacillota	*Saccharibacillus sacchari*	WP_340227389.1	WP_037289210.1	WP_037289904.1
Bacillota	*Saccharibacillus sp. CPCC 101409*	WP_302726611.1	WP_302726610.1	WP_302726059.1
Bacillota	*Saccharococcus caldoxylosilyticus*	WP_017435847.1	WP_017435848.1	WP_244380647.1
Bacillota	*Saccharococcus sp. Marseille-Q5394*	WP_262173031.1	WP_262174476.1	WP_262172861.1
Bacillota	*Saccharococcus thermophilus*	WP_166909718.1	WP_208404355.1	WP_166908162.1
Bacillota	*Salibacterium aidingense*	WP_026700929.1	WP_422787443.1	WP_026700134.1
Bacillota	*Salibacterium halotolerans*	WP_093335998.1	WP_093335997.1	WP_212634930.1
Bacillota	*Salibacterium lacus*	WP_380711740.1	WP_380711739.1	WP_380712172.1
Bacillota	*Salibacterium qingdaonense*	WP_090925891.1	WP_090925890.1	WP_177195403.1
Bacillota	*Salibacterium salarium*	WP_125555844.1	WP_348639443.1	WP_306897393.1
Bacillota	*Salicibibacter cibarius*	WP_200128665.1	WP_200127415.1	WP_200123865.1

Bacillota	*Salicibibacter cibi*	WP_200090038.1	WP_200088136.1	WP_200084986.1
Bacillota	*Salicibibacter halophilus*	WP_142086661.1	WP_142091167.1	WP_142087858.1
Bacillota	*Salicibibacter kimchii*	WP_114370407.1	WP_114375494.1	WP_227002793.1
Bacillota	*Salimicrobium album*	WP_093106267.1	WP_093107793.1	WP_176765374.1
Bacillota	*Salimicrobium flavidum*	WP_076556523.1	WP_200806510.1	WP_076556754.1
Bacillota	*Salimicrobium halophilum*	WP_093191291.1	WP_093193794.1	WP_176757401.1
Bacillota	*Salimicrobium humidisoli*	WP_095822251.1	WP_095822692.1	WP_158221305.1
Bacillota	*Salimicrobium jeotgali*	WP_102335850.1	WP_008588459.1	WP_180962605.1
Bacillota	*Salimicrobium sp. PL1-032A*	WP_347860490.1	WP_347861340.1	WP_347860669.1
Bacillota	*Salinibacillus aidingensis*	WP_425542165.1	WP_343842300.1	WP_343840898.1
Bacillota	*Salinibacillus kushneri*	WP_093136767.1	WP_093131125.1	WP_093136479.1
Bacillota	*Salinibacillus xinjiangensis*	WP_153728306.1	WP_323741785.1	WP_153727443.1
Bacillota	*Salinicoccus albus*	WP_020008327.1	WP_020007583.1	WP_020008736.1
Bacillota	*Salinicoccus carnicancri*	WP_017548951.1	WP_017549953.1	WP_017548448.1
Bacillota	*Salinicoccus halitifaciens*	WP_230820728.1	WP_230822019.1	WP_230820368.1
Bacillota	*Salinicoccus halodurans*	WP_046790275.1	WP_046791245.1	WP_046789886.1
Bacillota	*Salinicoccus hispanicus*	WP_160652528.1	WP_160655972.1	WP_160651099.1
Bacillota	*Salinicoccus jeotgali*	WP_344702988.1	WP_344704246.1	WP_344701231.1
Bacillota	*Salinicoccus kekensis*	WP_097038961.1	WP_097041364.1	WP_097038167.1
Bacillota	*Salinicoccus roseus*	WP_271398009.1	WP_222999666.1	WP_040105713.1
Bacillota	*Salinicoccus sediminis*	WP_046511680.1	WP_046513205.1	WP_046515947.1
Bacillota	*Salinicoccus sesuvii*	WP_380653846.1	WP_380651283.1	WP_380655097.1
Bacillota	*Salinicoccus siamensis*	WP_380569492.1	WP_380570766.1	WP_380571727.1
Bacillota	*Salinicoccus sp. YB14-2*	WP_052256313.1	WP_052254894.1	WP_052256699.1
Bacillota	*Salinithrix halophila*	WP_380705887.1	WP_380705888.1	WP_380703580.1
Bacillota	*Salipaludibacillus agaradhaerens*	WP_257832383.1	WP_078576843.1	WP_078576125.1
Bacillota	*Salipaludibacillus aurantiacus*	WP_093047103.1	WP_093047104.1	WP_093047693.1
Bacillota	*Salipaludibacillus daqingensis*	WP_280768198.1	WP_280768199.1	WP_280769090.1

Bacillota	*Salipaludibacillus keqinensis*	WP_110608171.1	WP_110608170.1	WP_110609279.1
Bacillota	*Salipaludibacillus neizhouensis*	WP_110935274.1	WP_110935273.1	WP_110936461.1
Bacillota	*Salipaludibacillus sp. CUR1*	WP_230896188.1	WP_230896189.1	WP_230898551.1
Bacillota	*Salirhabdus euzebyi*	WP_174495092.1	WP_174496052.1	WP_174497492.1
Bacillota	*Salirhabdus salicampi*	WP_254496435.1	WP_254497455.1	WP_254496371.1
Bacillota	*Salirhabdus sp. Marseille-P4669*	WP_102027217.1	WP_102028662.1	WP_102027290.1
Bacillota	*Salisediminibacterium beveridgei*	WP_069364871.1	WP_232318275.1	WP_232318319.1
Bacillota	*Salisediminibacterium halotolerans*	WP_121438815.1	WP_121438816.1	WP_121439723.1
Bacillota	*Salisediminibacterium selenitireducens*	WP_013173031.1	WP_013173032.1	WP_177304823.1
Bacillota	*Saliterribacillus persicus*	WP_114353814.1	WP_114353214.1	WP_114352801.1
Bacillota	*Salsuginibacillus halophilus*	WP_106587852.1	WP_106587851.1	WP_106587505.1
Bacillota	*Salsuginibacillus kocurii*	WP_018922278.1	WP_018922277.1	WP_018921352.1
Bacillota	*Savagea faecisuis*	WP_381011375.1	WP_381008702.1	WP_381010748.1
Bacillota	*Savagea serpentis*	WP_194561555.1	WP_194562939.1	WP_194561334.1
Bacillota	*Schinkia azotoformans*	WP_003331124.1	WP_035194851.1	WP_328240042.1
Bacillota	*Scopulibacillus cellulosilyticus*	WP_380965033.1	WP_380965031.1	WP_380964575.1
Bacillota	*Scopulibacillus daqui*	WP_205003192.1	WP_205003193.1	WP_205002233.1
Bacillota	*Scopulibacillus darangshiensis*	WP_132743194.1	WP_207902904.1	WP_132745980.1
Bacillota	*Sediminibacillus albus*	WP_093210385.1	WP_093213481.1	WP_093210933.1
Bacillota	*Sediminibacillus dalangtanensis*	WP_209369049.1	WP_209365498.1	WP_209368594.1
Bacillota	*Sediminibacillus halophilus*	WP_074597154.1	WP_074599060.1	WP_074597461.1
Bacillota	*Sediminibacillus massiliensis*	WP_077621753.1	WP_077624813.1	WP_077622117.1
Bacillota	*Selenihalanaerobacter shriftii*	WP_078809290.1	WP_078809291.1	WP_078809292.1
Bacillota	*Shimazuella alba*	WP_160799579.1	WP_160799580.1	WP_160802711.1
Bacillota	*Shimazuella kribbensis*	WP_028775877.1	WP_037463927.1	WP_028775472.1
Bacillota	*Shimazuella soli*	WP_240875942.1	WP_240875941.1	WP_240873807.1
Bacillota	*Shouchella clausii*	WP_011246913.1	WP_308899182.1	WP_094427356.1
Bacillota	*Shouchella hunanensis*	WP_274272691.1	WP_274272692.1	WP_274272785.1

Bacillota	*Shouchella lehensis*	WP_124742136.1	WP_038480190.1	WP_051667554.1
Bacillota	*Shouchella lonarensis*	WP_090774967.1	WP_090774966.1	WP_090775969.1
Bacillota	*Shouchella miscanthi*	WP_060705393.1	WP_035398362.1	WP_144559213.1
Bacillota	*Shouchella patagoniensis*	WP_078391140.1	WP_078391141.1	WP_078391589.1
Bacillota	*Shouchella shacheensis*	WP_059105136.1	WP_245628145.1	WP_059104709.1
Bacillota	*Shouchella tritolerans*	WP_263706276.1	WP_212953660.1	WP_263707492.1
Bacillota	*Shouchella xiaoxiensis*	WP_204467695.1	WP_204467693.1	WP_204464544.1
Bacillota	*Siminovitchia acidinfaciens*	WP_126051034.1	WP_126051035.1	WP_126049851.1
Bacillota	*Siminovitchia fordii*	WP_144516662.1	WP_212962979.1	WP_018705236.1
Bacillota	*Siminovitchia fortis*	WP_144461110.1	WP_144461111.1	WP_120073885.1
Bacillota	*Siminovitchia sediminis*	WP_380773864.1	WP_380773863.1	WP_380772334.1
Bacillota	*Siminovitchia sp. 179-K 8D1 HS*	WP_413064294.1	WP_413064293.1	WP_413062858.1
Bacillota	*Siminovitchia terrae*	WP_120117407.1	WP_213020524.1	WP_212948967.1
Bacillota	*Sinobaca qinghaiensis*	WP_120193227.1	WP_120193228.1	WP_120192335.1
Bacillota	*Solibacillus cecembensis*	WP_057982258.1	WP_057986633.1	WP_427763025.1
Bacillota	*Solibacillus daqui*	WP_274309732.1	WP_274309051.1	WP_274309369.1
Bacillota	*Solibacillus isronensis*	WP_079525889.1	WP_413079058.1	WP_079525008.1
Bacillota	*Solibacillus kalamii*	WP_087618188.1	WP_087615704.1	WP_087615483.1
Bacillota	*Solibacillus palustris*	WP_241368297.1	WP_241367517.1	WP_241367882.1
Bacillota	*Solibacillus sp.*	WP_350479019.1	WP_350478533.1	WP_350477183.1
Bacillota	*Sporosarcina aquimarina*	WP_317934445.1	WP_317934225.1	WP_221836181.1
Bacillota	*Sporosarcina beigongshangi*	WP_212391108.1	WP_322555715.1	WP_203246136.1
Bacillota	*Sporosarcina contaminans*	WP_381481929.1	WP_381480857.1	WP_381479742.1
Bacillota	*Sporosarcina cyprini*	WP_238083723.1	WP_238079673.1	WP_238084261.1
Bacillota	*Sporosarcina gallistercoris*	WP_191688084.1	WP_191690044.1	WP_191688376.1
Bacillota	*Sporosarcina globispora*	WP_053434600.1	WP_053434599.1	WP_053436572.1
Bacillota	*Sporosarcina highlanderae*	WP_301244972.1	WP_301241610.1	WP_301243091.1
Bacillota	*Sporosarcina jeotgali*	WP_323693048.1	WP_323692251.1	WP_323692818.1

Bacillota	*Sporosarcina jiandibaonis*	WP_172369382.1	WP_172371255.1	WP_172369776.1
Bacillota	*Sporosarcina koreensis*	WP_060208210.1	WP_235588630.1	WP_082713665.1
Bacillota	*Sporosarcina limicola*	WP_192597920.1	WP_192596793.1	WP_192597375.1
Bacillota	*Sporosarcina luteola*	WP_147059772.1	WP_251697812.1	WP_147054836.1
Bacillota	*Sporosarcina obsidiansis*	WP_153732236.1	WP_153730310.1	WP_153731198.1
Bacillota	*Sporosarcina oncorhynchi*	WP_317970799.1	WP_317970857.1	WP_317965138.1
Bacillota	*Sporosarcina pasteurii*	WP_115360685.1	WP_115359879.1	WP_115360375.1
Bacillota	*Sporosarcina quadrami*	WP_191695924.1	WP_191695183.1	WP_191693077.1
Bacillota	*Sporosarcina siberiensis*	WP_381537257.1	WP_381539562.1	WP_381538696.1
Bacillota	*Sporosarcina soli*	WP_381431378.1	WP_381434861.1	WP_381431890.1
Bacillota	*Sporosarcina sp.*	WP_301107046.1	WP_301107252.1	WP_301108213.1
Bacillota	*Sporosarcina thermotolerans*	WP_283731851.1	WP_381426396.1	WP_283731650.1
Bacillota	*Sporosarcina trichiuri*	WP_290148243.1	WP_290148363.1	WP_290150366.1
Bacillota	*Sporosarcina ureae*	WP_029053023.1	WP_303969287.1	WP_029052381.1
Bacillota	*Sporosarcina ureilytica*	WP_075528193.1	WP_075529057.1	WP_075528509.1
Bacillota	*Staphylococcus agnetis*	WP_105995156.1	WP_186444660.1	WP_037564834.1
Bacillota	*Staphylococcus americanisciuri*	WP_259197863.1	WP_259199938.1	WP_259199379.1
Bacillota	*Staphylococcus argenteus*	WP_000690034.1	WP_088839925.1	WP_000340487.1
Bacillota	*Staphylococcus arlettae*	WP_313693173.1	WP_107377050.1	WP_002510904.1
Bacillota	*Staphylococcus aureus*	WP_061651898.1	WP_064139785.1	WP_046400101.1
Bacillota	*Staphylococcus auricularis*	WP_278925602.1	WP_059107383.1	WP_059106611.1
Bacillota	*Staphylococcus borealis*	WP_325968792.1	WP_320795828.1	WP_053029182.1
Bacillota	*Staphylococcus caeli*	WP_069996062.1	WP_069994236.1	WP_069995665.1
Bacillota	*Staphylococcus caledonicus*	WP_423584440.1	WP_423585406.1	WP_198611262.1
Bacillota	*Staphylococcus canis*	WP_198618143.1	WP_198617867.1	WP_198617317.1
Bacillota	*Staphylococcus capitis*	WP_227938630.1	WP_367121073.1	WP_103156962.1
Bacillota	*Staphylococcus caprae*	WP_206416781.1	WP_206416920.1	WP_002442407.1
Bacillota	*Staphylococcus carnosus*	WP_103211976.1	WP_012664257.1	WP_015900042.1

Bacillota	*Staphylococcus casei*	WP_069824026.1	WP_069823448.1	WP_103267406.1
Bacillota	*Staphylococcus chromogenes*	WP_037573738.1	WP_105985726.1	WP_145401049.1
Bacillota	*Staphylococcus coagulans*	WP_182317341.1	WP_050346061.1	WP_241415290.1
Bacillota	*Staphylococcus cohnii*	WP_119561916.1	WP_107504835.1	WP_103211509.1
Bacillota	*Staphylococcus cornubiensis*	WP_086427769.1	WP_086428521.1	WP_086429092.1
Bacillota	*Staphylococcus croceilyticus*	WP_103328611.1	WP_103329602.1	WP_103330022.1
Bacillota	*Staphylococcus debuckii*	WP_123144728.1	WP_123145576.1	WP_123144472.1
Bacillota	*Staphylococcus delphini*	WP_160215323.1	WP_212574807.1	WP_262612528.1
Bacillota	*Staphylococcus devriesei*	WP_119526972.1	WP_103166080.1	WP_103167490.1
Bacillota	*Staphylococcus durrellii*	WP_195720778.1	WP_195721453.1	WP_195721025.1
Bacillota	*Staphylococcus edaphicus*	WP_099090706.1	WP_099089595.1	WP_099089336.1
Bacillota	*Staphylococcus epidermidis*	WP_193390020.1	WP_139689271.1	WP_060556068.1
Bacillota	*Staphylococcus equorum*	WP_277580753.1	WP_197911036.1	WP_021338687.1
Bacillota	*Staphylococcus felis*	WP_262632082.1	WP_349421082.1	WP_103208719.1
Bacillota	*Staphylococcus gallinarum*	WP_119485698.1	WP_042738051.1	WP_232154922.1
Bacillota	*Staphylococcus haemolyticus*	WP_070111072.1	WP_053026387.1	WP_057504811.1
Bacillota	*Staphylococcus hominis*	WP_172473662.1	WP_311045257.1	WP_119651854.1
Bacillota	*Staphylococcus hyicus*	WP_039645720.1	WP_167694023.1	WP_039646206.1
Bacillota	*Staphylococcus intermedius*	WP_019167612.1	WP_019169043.1	WP_019169118.1
Bacillota	*Staphylococcus kloosii*	WP_195708453.1	WP_103295076.1	WP_313544265.1
Bacillota	*Staphylococcus lloydii*	WP_316670929.1	WP_048792414.1	WP_195718300.1
Bacillota	*Staphylococcus lugdunensis*	WP_002459091.1	WP_002459906.1	WP_037545920.1
Bacillota	*Staphylococcus lutrae*	WP_085238316.1	WP_085237590.1	WP_085238060.1
Bacillota	*Staphylococcus marylandisciuri*	WP_262854638.1	WP_262856279.1	WP_262855078.1
Bacillota	*Staphylococcus massiliensis*	WP_009383771.1	WP_009383870.1	WP_009381584.1
Bacillota	*Staphylococcus microti*	WP_044358858.1	WP_044359276.1	WP_044359784.1
Bacillota	*Staphylococcus muscae*	WP_095116950.1	WP_095115276.1	WP_095116212.1
Bacillota	*Staphylococcus nepalensis*	WP_349790011.1	WP_103373391.1	WP_096810346.1

Bacillota	*Staphylococcus pasteuri*	WP_326002342.1	WP_259966835.1	WP_126568352.1
Bacillota	*Staphylococcus petrasii*	WP_242238694.1	WP_103298240.1	WP_103366839.1
Bacillota	*Staphylococcus pettenkoferi*	WP_002472138.1	WP_268216578.1	WP_002472853.1
Bacillota	*Staphylococcus pseudintermedius*	WP_390575169.1	WP_199274268.1	WP_063278835.1
Bacillota	*Staphylococcus pseudoxylosus*	WP_214269407.1	WP_214307630.1	WP_326020120.1
Bacillota	*Staphylococcus ratti*	WP_229291658.1	WP_229292403.1	WP_229293642.1
Bacillota	*Staphylococcus rostri*	WP_103358010.1	WP_103358500.1	WP_103358994.1
Bacillota	*Staphylococcus saccharolyticus*	WP_349546522.1	WP_130559112.1	WP_115313559.1
Bacillota	*Staphylococcus saprophyticus*	WP_129319395.1	WP_326023640.1	WP_011303331.1
Bacillota	*Staphylococcus schweitzeri*	WP_047552404.1	WP_047427805.1	WP_047549618.1
Bacillota	*Staphylococcus shinii*	WP_101049392.1	WP_318760341.1	WP_107548731.1
Bacillota	*Staphylococcus simiae*	WP_002463071.1	WP_207517890.1	WP_002465050.1
Bacillota	*Staphylococcus simulans*	WP_107604644.1	WP_412519330.1	WP_119551259.1
Bacillota	*Staphylococcus sp.*	WP_287173972.1	WP_293735409.1	WP_287236828.1
Bacillota	*Staphylococcus warneri*	WP_270232372.1	WP_107546604.1	WP_058709479.1
Bacillota	*Staphylococcus xylosus*	WP_119614750.1	WP_101104024.1	WP_042362890.1
Bacillota	*Staphylospora marina*	WP_124727366.1	WP_124727365.1	WP_124727719.1
Bacillota	*Sulfoacidibacillus ferrooxidans*	WP_241715794.1	WP_241715796.1	WP_241711416.1
Bacillota	*Sulfoacidibacillus thermotolerans*	WP_109431150.1	WP_109431149.1	WP_109430457.1
Bacillota	*Sutcliffiella cohnii*	WP_066415539.1	WP_412766311.1	WP_328225366.1
Bacillota	*Sutcliffiella deserti*	WP_223702018.1	WP_223702019.1	WP_223700326.1
Bacillota	*Sutcliffiella halmapala*	WP_078380210.1	WP_078380209.1	WP_169865011.1
Bacillota	*Sutcliffiella horikoshii*	WP_426910852.1	WP_251429387.1	WP_237661957.1
Bacillota	*Sutcliffiella rhizosphaerae*	WP_230503349.1	WP_230503351.1	WP_230499742.1
Bacillota	*Sutcliffiella sp. NPDC057660*	WP_386638742.1	WP_386638745.1	WP_386644139.1
Bacillota	*Symbiobacterium terraclitae*	WP_209467923.1	WP_209467487.1	WP_209467439.1
Bacillota	*Symbiobacterium thermophilum*	WP_011195753.1	WP_273380602.1	WP_043714106.1
Bacillota	*Tenuibacillus multivorans*	WP_093857175.1	WP_093857303.1	WP_093855768.1

Bacillota	*Terribacillus aidingensis*	WP_366249817.1	WP_097039613.1	WP_097040255.1
Bacillota	*Terribacillus halophilus*	WP_093728367.1	WP_093725557.1	WP_411230823.1
Bacillota	*Terribacillus saccharophilus*	WP_411213887.1	WP_411216852.1	WP_411218624.1
Bacillota	*Terribacillus sp. 179-K 1B1 HS*	WP_413088889.1	WP_413088162.1	WP_413091881.1
Bacillota	*Terrihalobacillus insolitus*	WP_272436575.1	WP_272434696.1	WP_272435445.1
Bacillota	*Terrilactibacillus laevilacticus*	WP_141603530.1	WP_141189502.1	WP_141189187.1
Bacillota	*Terrilactibacillus tamarindi*	WP_155220068.1	WP_155220070.1	WP_155218608.1
Bacillota	*Tetzosporium hominis*	WP_243370631.1	WP_094943548.1	WP_094941578.1
Bacillota	*Texcoconibacillus texcoconensis*	WP_184664026.1	WP_184664025.1	WP_184662655.1
Bacillota	*Thalassobacillus cyri*	WP_093044700.1	WP_176791319.1	WP_093046616.1
Bacillota	*Thalassobacillus devorans*	WP_085508675.1	WP_037986477.1	WP_085508061.1
Bacillota	*Thalassobacillus hwangdonensis*	WP_386056987.1	WP_386062940.1	WP_386056301.1
Bacillota	*Thalassobacillus pellis*	WP_205091761.1	WP_205092936.1	WP_205092104.1
Bacillota	*Thalassobacillus sp. FIB228*	WP_394460240.1	WP_394460934.1	WP_394459867.1
Bacillota	*Thermaerobacillus caldiproteolyticus*	WP_181555922.1	WP_194520666.1	WP_181555025.1
Bacillota	*Thermaerobacter composti*	WP_318751533.1	WP_318751084.1	WP_318751445.1
Bacillota	*Thermaerobacter marianensis*	WP_013495781.1	WP_013495780.1	WP_013495475.1
Bacillota	*Thermaerobacter sp. FW80*	WP_135225485.1	WP_135225000.1	WP_135225372.1
Bacillota	*Thermaerobacter subterraneus*	WP_006903784.1	WP_006902983.1	WP_423219092.1
Bacillota	*Thermicanus aegyptius*	WP_028986721.1	WP_005582017.1	WP_156920558.1
Bacillota	*Thermincola ferriacetica*	WP_052218853.1	WP_052218852.1	WP_052218485.1
Bacillota	*Thermincola potens*	WP_013121178.1	WP_013121179.1	WP_013119642.1
Bacillota	*Thermoactinomyces daqus*	WP_033101403.1	WP_033101447.1	WP_052154059.1
Bacillota	*Thermoactinomyces mirandus*	WP_181740253.1	WP_181740251.1	WP_181737370.1
Bacillota	*Thermoactinomyces sp. DSM 45891*	WP_072329871.1	WP_072329873.1	WP_072331764.1
Bacillota	*Thermobacillus xylanilyticus*	WP_213484588.1	WP_213484587.1	WP_213484019.1
Bacillota	*Thermoflavimicrobium daqui*	WP_113660335.1	WP_113660334.1	WP_113658619.1
Bacillota	*Thermoflavimicrobium dichotomicum*	WP_093230852.1	WP_093230851.1	WP_175482318.1

Bacillota	*Thermolongibacillus altinsuensis*	WP_132947109.1	WP_132947108.1	WP_132948044.1
Bacillota	*Tuberibacillus calidus*	WP_027723795.1	WP_027725603.1	WP_027726143.1
Bacillota	*Tuberibacillus sp. Marseille-P3662*	WP_085523572.1	WP_085523628.1	WP_085524079.1
Bacillota	*Ureibacillus acetophenoni*	WP_097148931.1	WP_097147578.1	WP_097147917.1
Bacillota	*Ureibacillus aquaedulcis*	WP_301138178.1	WP_301136159.1	WP_301136504.1
Bacillota	*Ureibacillus chungkukjangi*	WP_107934324.1	WP_107936772.1	WP_251397087.1
Bacillota	*Ureibacillus galli*	WP_191705773.1	WP_191706082.1	WP_191706386.1
Bacillota	*Ureibacillus manganicus*	WP_036183726.1	WP_036182408.1	WP_036181806.1
Bacillota	*Ureibacillus massiliensis*	WP_036176731.1	WP_036171102.1	WP_036171897.1
Bacillota	*Ureibacillus sinduriensis*	WP_036203690.1	WP_036200036.1	WP_036202732.1
Bacillota	*Ureibacillus sp. GCM10028918*	WP_390284955.1	WP_390286420.1	WP_390285722.1
Bacillota	*Ureibacillus thermophilus*	WP_208649562.1	WP_208651565.1	WP_208651930.1
Bacillota	*Ureibacillus thermosphaericus*	WP_168411771.1	WP_340436786.1	WP_168411946.1
Bacillota	*Ureibacillus xyleni*	WP_097074454.1	WP_097071621.1	WP_097072042.1
Bacillota	*Virgibacillus alimentarius*	WP_029267903.1	WP_226370754.1	WP_226370470.1
Bacillota	*Virgibacillus byunsanensis*	WP_390361856.1	WP_390362218.1	WP_390362069.1
Bacillota	*Virgibacillus dokdonensis*	WP_116277069.1	WP_077706929.1	WP_077704362.1
Bacillota	*Virgibacillus dougmboii*	WP_164668779.1	WP_164668118.1	WP_164669103.1
Bacillota	*Virgibacillus halodenitrificans*	WP_121614998.1	WP_019377733.1	WP_283875060.1
Bacillota	*Virgibacillus halophilus*	WP_390353554.1	WP_390357844.1	WP_390352980.1
Bacillota	*Virgibacillus halotolerans*	WP_205135334.1	WP_205137470.1	WP_205135629.1
Bacillota	*Virgibacillus ihumii*	WP_174613585.1	WP_174614267.1	WP_174613153.1
Bacillota	*Virgibacillus indicus*	WP_094883727.1	WP_094885865.1	WP_094884008.1
Bacillota	*Virgibacillus kekensis*	WP_390293313.1	WP_390293469.1	WP_390292948.1
Bacillota	*Virgibacillus litoralis*	WP_209481076.1	WP_209479546.1	WP_209482083.1
Bacillota	*Virgibacillus natechei*	WP_209462782.1	WP_209462604.1	WP_209461433.1
Bacillota	*Virgibacillus ndiopensis*	WP_099158340.1	WP_099159900.1	WP_099157985.1
Bacillota	*Virgibacillus necropolis*	WP_404452830.1	WP_404454248.1	WP_089532707.1

Bacillota	*Virgibacillus oceani*	WP_188453881.1	WP_188454115.1	WP_188454492.1
Bacillota	*Virgibacillus pantothenticus*	WP_077303373.1	WP_077301211.1	WP_077300414.1
Bacillota	*Virgibacillus phasianinus*	WP_089062613.1	WP_089061994.1	WP_089062850.1
Bacillota	*Virgibacillus profundi*	WP_095655681.1	WP_095657386.1	WP_095655102.1
Bacillota	*Virgibacillus proomii*	WP_077320600.1	WP_077320023.1	WP_077320327.1
Bacillota	*Virgibacillus sediminis*	WP_390305164.1	WP_390302484.1	WP_390308289.1
Bacillota	*Virgibacillus senegalensis*	WP_053219421.1	WP_053218627.1	WP_053219771.1
Bacillota	*Virgibacillus siamensis*	WP_077326649.1	WP_077328044.1	WP_077325994.1
Bacillota	*Virgibacillus soli*	WP_390185828.1	WP_320379603.1	WP_320378859.1
Bacillota	*Virgibacillus sp. MSJ-26*	WP_216487690.1	WP_216489624.1	WP_216489590.1
Bacillota	*Virgibacillus tibetensis*	WP_327606045.1	WP_327608938.1	WP_327606340.1
Bacillota	*Virgibacillus xinjiangensis*	WP_390267290.1	WP_390273709.1	WP_390271245.1
Bacillota	*Viridibacillus arvi*	WP_390008744.1	WP_053417950.1	WP_053417543.1
Bacillota	*Viridibacillus soli*	WP_200750120.1	WP_200749327.1	WP_200748351.1
Bacillota	*Weizmannia acidilactici*	WP_151680746.1	WP_151697946.1	WP_151681373.1
Bacillota	*Xylanibacillus composti*	WP_213410179.1	WP_213410178.1	WP_213410594.1
Bacillota	*Zhaonella formicivorans*	WP_227766183.1	WP_227767134.1	WP_227767136.1
Bacteroidota	*Abyssalbus ytuae*	WP_255842322.1	WP_255842580.1	WP_255843861.1
Bacteroidota	*Acidiluteibacter ferrifornacis*	WP_160634009.1	WP_160633238.1	WP_160632436.1
Bacteroidota	*Adhaeribacter aerolatus*	WP_146903393.1	WP_146896245.1	WP_146900283.1
Bacteroidota	*Adhaeribacter aquaticus*	WP_026462874.1	WP_026464488.1	WP_026461962.1
Bacteroidota	*Adhaeribacter arboris*	WP_106925362.1	WP_106927209.1	WP_106933576.1
Bacteroidota	*Adhaeribacter pallidiroseus*	WP_115373291.1	WP_115373435.1	WP_115375762.1
Bacteroidota	*Adhaeribacter radiodurans*	WP_182414787.1	WP_182415598.1	WP_182414468.1
Bacteroidota	*Adhaeribacter rhizoryzae*	WP_150087427.1	WP_150087644.1	WP_150086256.1
Bacteroidota	*Adhaeribacter soli*	WP_150904862.1	WP_150905911.1	WP_150903363.1
Bacteroidota	*Adhaeribacter swui*	WP_185270322.1	WP_185274619.1	WP_185272839.1
Bacteroidota	*Adhaeribacter terreus*	WP_378015753.1	WP_378016465.1	WP_378017653.1

Bacteroidota	*Adhaeribacter terrigena*	WP_200504364.1	WP_200505621.1	WP_200506036.1
Bacteroidota	*Aegicerativicinus sediminis*	WP_228851488.1	WP_228851243.1	WP_228851022.1
Bacteroidota	*Aequorivita antarctica*	WP_111843834.1	WP_111845043.1	WP_111844811.1
Bacteroidota	*Aequorivita aquimaris*	WP_062621430.1	WP_062619548.1	WP_062619660.1
Bacteroidota	*Aequorivita aurantiaca*	WP_290253605.1	WP_290255165.1	WP_290255086.1
Bacteroidota	*Aequorivita capsosiphonis*	WP_026449899.1	WP_026451927.1	WP_026450721.1
Bacteroidota	*Aequorivita ciconiae*	WP_128249060.1	WP_128249027.1	WP_128251637.1
Bacteroidota	*Aequorivita echinoideorum*	WP_214113607.1	WP_214113075.1	WP_214113159.1
Bacteroidota	*Aequorivita lipolytica*	WP_111815969.1	WP_111816248.1	WP_111816891.1
Bacteroidota	*Aequorivita marina*	WP_310991922.1	WP_310992157.1	WP_310993816.1
Bacteroidota	*Aequorivita nionensis*	WP_410005228.1	WP_410004785.1	WP_410007104.1
Bacteroidota	*Aequorivita sinensis*	WP_271424522.1	WP_271393576.1	WP_271393529.1
Bacteroidota	*Aequorivita soesokkakensis*	WP_068760878.1	WP_068761363.1	WP_068760990.1
Bacteroidota	*Aequorivita sp. Q41*	WP_347374707.1	WP_347372786.1	WP_347374868.1
Bacteroidota	*Aequorivita sublithincola*	WP_014783441.1	WP_014781031.1	WP_014780829.1
Bacteroidota	*Aequorivita todarodis*	WP_272854907.1	WP_272856410.1	WP_272856446.1
Bacteroidota	*Aequorivita viscosa*	WP_073219450.1	WP_073221462.1	WP_073214181.1
Bacteroidota	*Aequorivita vitellina*	WP_237602884.1	WP_237604082.1	WP_237604065.1
Bacteroidota	*Aequorivita vladivostokensis*	WP_045080904.1	WP_045080108.1	WP_045081657.1
Bacteroidota	*Aequorivita xiaoshiensis*	WP_237607055.1	WP_237608260.1	WP_237608850.1
Bacteroidota	*Aestuariibaculum lutulentum*	WP_240572916.1	WP_240574573.1	WP_240573340.1
Bacteroidota	*Aestuariibaculum marinum*	WP_188223804.1	WP_188221776.1	WP_188222367.1
Bacteroidota	*Aestuariibaculum sediminum*	WP_188229943.1	WP_188229495.1	WP_188230458.1
Bacteroidota	*Aestuariibaculum sp. YM273*	WP_307881004.1	WP_307881203.1	WP_307881758.1
Bacteroidota	*Aestuariibaculum suncheonense*	WP_188215830.1	WP_188215657.1	WP_188216389.1
Bacteroidota	*Aestuariivivens insulae*	WP_242204282.1	WP_242206092.1	WP_242205612.1
Bacteroidota	*Aestuariivivens marinum*	WP_242132290.1	WP_242135394.1	WP_242135503.1
Bacteroidota	*Aestuariivivens sediminicola*	WP_242120336.1	WP_242121371.1	WP_242119724.1

Bacteroidota	*Aestuariivivens sediminis*	WP_242086932.1	WP_242158599.1	WP_242085353.1
Bacteroidota	*Aestuariivivens sp. NBU2969*	WP_223548296.1	WP_223551508.1	WP_223552062.1
Bacteroidota	*Agaribacillus aureus*	WP_346757862.1	WP_346755833.1	WP_346761836.1
Bacteroidota	*Aggregatimonas sangjinii*	WP_138852003.1	WP_138852101.1	WP_138851661.1
Bacteroidota	*Agriterribacter humi*	WP_152268168.1	WP_152270501.1	WP_152266726.1
Bacteroidota	*Agriterribacter sp.*	WP_325404972.1	WP_325403507.1	WP_324556562.1
Bacteroidota	*Albibacterium bauzanense*	WP_132222972.1	WP_132221473.1	WP_132222344.1
Bacteroidota	*Albibacterium sp.*	WP_325357452.1	WP_325358942.1	WP_325359824.1
Bacteroidota	*Algibacter agarivorans*	WP_345189876.1	WP_345193043.1	WP_345193815.1
Bacteroidota	*Algibacter amylolyticus*	WP_144118081.1	WP_144117049.1	WP_144116980.1
Bacteroidota	*Algibacter aquimarinus*	WP_345169810.1	WP_345170338.1	WP_345169783.1
Bacteroidota	*Algibacter lectus*	WP_074936123.1	WP_304407024.1	WP_282148460.1
Bacteroidota	*Algibacter luteus*	WP_019386403.1	WP_289194526.1	WP_019388342.1
Bacteroidota	*Algibacter marinivivus*	WP_109353276.1	WP_109353074.1	WP_109353815.1
Bacteroidota	*Algibacter mikhailovii*	WP_189358466.1	WP_189362427.1	WP_189362358.1
Bacteroidota	*Algibacter miyuki*	WP_290273454.1	WP_290271960.1	WP_290273464.1
Bacteroidota	*Algibacter pacificus*	WP_147678726.1	WP_147678533.1	WP_147678795.1
Bacteroidota	*Algibacter pectinivorans*	WP_092853154.1	WP_092852392.1	WP_092851984.1
Bacteroidota	*Algibacter sp.*	WP_424201046.1	WP_348451855.1	WP_348451771.1
Bacteroidota	*Algivirga pacifica*	WP_345373764.1	WP_345373213.1	WP_345371319.1
Bacteroidota	*Algoriella sp.*	WP_332544110.1	WP_332546255.1	WP_332543467.1
Bacteroidota	*Algoriphagus aestuariicola*	WP_206570757.1	WP_206568757.1	WP_206569828.1
Bacteroidota	*Algoriphagus alkaliphilus*	WP_092730293.1	WP_092732049.1	WP_092729109.1
Bacteroidota	*Algoriphagus antarcticus*	WP_086543870.1	WP_086542156.1	WP_086541399.1
Bacteroidota	*Algoriphagus aquaeductus*	WP_409682485.1	WP_406949959.1	WP_406949864.1
Bacteroidota	*Algoriphagus aquatilis*	WP_377917023.1	WP_377912258.1	WP_377917659.1
Bacteroidota	*Algoriphagus aquimarinus*	WP_092894558.1	WP_146919054.1	WP_092894759.1
Bacteroidota	*Algoriphagus aquimaris*	WP_133390194.1	WP_133391627.1	WP_240686591.1

Bacteroidota	*Algoriphagus boritolerans*	WP_332911655.1	WP_103925912.1	WP_103924010.1
Bacteroidota	*Algoriphagus boseongensis*	WP_133555455.1	WP_133552919.1	WP_133555600.1
Bacteroidota	*Algoriphagus chordae*	WP_111316191.1	WP_111323425.1	WP_111318264.1
Bacteroidota	*Algoriphagus confluentis*	WP_338223545.1	WP_338223409.1	WP_338223464.1
Bacteroidota	*Algoriphagus faecimaris*	WP_087938083.1	WP_087938226.1	WP_087940368.1
Bacteroidota	*Algoriphagus formosus*	WP_100628282.1	WP_100627774.1	WP_232724787.1
Bacteroidota	*Algoriphagus halophilus*	WP_074226768.1	WP_074224025.1	WP_373400315.1
Bacteroidota	*Algoriphagus hitonicola*	WP_092791543.1	WP_092789748.1	WP_092791970.1
Bacteroidota	*Algoriphagus iocasae*	WP_184494711.1	WP_184497394.1	WP_184495802.1
Bacteroidota	*Algoriphagus jejuensis*	WP_343852720.1	WP_343853072.1	WP_343850080.1
Bacteroidota	*Algoriphagus kandeliae*	WP_135075615.1	WP_135070713.1	WP_135072807.1
Bacteroidota	*Algoriphagus lacus*	WP_119477098.1	WP_119477573.1	WP_119477184.1
Bacteroidota	*Algoriphagus limi*	WP_259415152.1	WP_259413116.1	WP_259414277.1
Bacteroidota	*Algoriphagus litoralis*	WP_111670835.1	WP_111669801.1	WP_111671671.1
Bacteroidota	*Algoriphagus locisalis*	WP_091692921.1	WP_091691830.1	WP_091695981.1
Bacteroidota	*Algoriphagus lutimaris*	WP_205646464.1	WP_205644250.1	WP_205645590.1
Bacteroidota	*Algoriphagus machipongonensis*	WP_008203198.1	WP_008200935.1	WP_008198563.1
Bacteroidota	*Algoriphagus mannitolivorans*	WP_026951984.1	WP_026951705.1	WP_026951897.1
Bacteroidota	*Algoriphagus marinus*	WP_075350005.1	WP_075352665.1	WP_075350307.1
Bacteroidota	*Algoriphagus namhaensis*	WP_377903089.1	WP_377906576.1	WP_377903203.1
Bacteroidota	*Algoriphagus oliviformis*	WP_206576919.1	WP_206577607.1	WP_206579989.1
Bacteroidota	*Algoriphagus ornithinivorans*	WP_091652719.1	WP_091650703.1	WP_091651060.1
Bacteroidota	*Algoriphagus pacificus*	WP_206584645.1	WP_206587470.1	WP_206585619.1
Bacteroidota	*Algoriphagus ratkowskyi*	WP_086500922.1	WP_086501427.1	WP_086501982.1
Bacteroidota	*Algoriphagus resistens*	WP_057936911.1	WP_057936316.1	WP_057939717.1
Bacteroidota	*Algoriphagus sanaruensis*	WP_067550178.1	WP_067550458.1	WP_067544506.1
Bacteroidota	*Algoriphagus sediminis*	WP_290000812.1	WP_289999935.1	WP_290000871.1
Bacteroidota	*Algoriphagus sp.*	WP_348275023.1	WP_300316368.1	WP_348277066.1

Bacteroidota	*Algoriphagus taiwanensis*	WP_338230563.1	WP_338227733.1	WP_338229737.1
Bacteroidota	*Algoriphagus terrigena*	WP_026968918.1	WP_026967199.1	WP_026967940.1
Bacteroidota	*Algoriphagus vanfongensis*	WP_026956411.1	WP_026954082.1	WP_026955295.1
Bacteroidota	*Algoriphagus winogradskyi*	WP_283412018.1	WP_283413402.1	WP_283414707.1
Bacteroidota	*Algoriphagus yeomjeoni*	WP_111609732.1	WP_425639078.1	WP_425636259.1
Bacteroidota	*Algoriphagus zhangzhouensis*	WP_073572735.1	WP_073570160.1	WP_073571012.1
Bacteroidota	*Allomuricauda sp. SCSIO 65647*	WP_235265680.1	WP_235265793.1	WP_235266678.1
Bacteroidota	*Allotamlana fucoidanivorans*	WP_139694982.1	WP_139698433.1	WP_139696462.1
Bacteroidota	*Altibacter lentus*	WP_034258177.1	WP_034257797.1	WP_034259005.1
Bacteroidota	*Altibacter sp.*	WP_290787002.1	WP_290785786.1	WP_290785445.1
Bacteroidota	*Anditalea andensis*	WP_035075984.1	WP_035072741.1	WP_035075153.1
Bacteroidota	*Anseongella ginsenosidimutans*	WP_132129421.1	WP_132128381.1	WP_132129763.1
Bacteroidota	*Antarcticibacterium sp. 1MA-6-2*	WP_235328210.1	WP_235329999.1	WP_235330129.1
Bacteroidota	*Apibacter adventoris*	WP_105193923.1	WP_181042806.1	WP_105245879.1
Bacteroidota	*Apibacter mensalis*	WP_303849861.1	WP_303839970.1	WP_303822039.1
Bacteroidota	*Apibacter muscae*	WP_146292415.1	WP_146292170.1	WP_146311829.1
Bacteroidota	*Apibacter sp.*	WP_293917054.1	WP_293918312.1	WP_293918330.1
Bacteroidota	*Aquaticitalea lipolytica*	WP_283636607.1	WP_188604491.1	WP_188606921.1
Bacteroidota	*Aquiflexum balticum*	WP_084118598.1	WP_084121789.1	WP_084118744.1
Bacteroidota	*Aquiflexum gelatinilyticum*	WP_258422916.1	WP_258424477.1	WP_259263102.1
Bacteroidota	*Aquiflexum lacus*	WP_194972829.1	WP_194976628.1	WP_194975748.1
Bacteroidota	*Aquiflexum sp.*	WP_373522937.1	WP_373523815.1	WP_373521777.1
Bacteroidota	*Aquimarina acroporae*	WP_248325971.1	WP_248327184.1	WP_248329473.1
Bacteroidota	*Aquimarina addita*	WP_344928121.1	WP_344926945.1	WP_344927438.1
Bacteroidota	*Aquimarina agarilytica*	WP_010182235.1	WP_010178350.1	WP_010182594.1
Bacteroidota	*Aquimarina agarivorans*	WP_010521832.1	WP_010522891.1	WP_010520711.1
Bacteroidota	*Aquimarina aggregata*	WP_281986244.1	WP_066317910.1	WP_281990476.1
Bacteroidota	*Aquimarina algicola*	WP_140589169.1	WP_140595569.1	WP_140595083.1

Bacteroidota	*Aquimarina algiphila*	WP_271767707.1	WP_271766777.1	WP_282086395.1
Bacteroidota	*Aquimarina amphilecti*	WP_091404647.1	WP_091403964.1	WP_091410641.1
Bacteroidota	*Aquimarina aquimarini*	WP_108869272.1	WP_108868242.1	WP_108868066.1
Bacteroidota	*Aquimarina atlantica*	WP_034238049.1	WP_034241498.1	WP_034246824.1
Bacteroidota	*Aquimarina brevivitae*	WP_130286860.1	WP_130287960.1	WP_130286092.1
Bacteroidota	*Aquimarina celericrescens*	WP_378318285.1	WP_378318615.1	WP_378321627.1
Bacteroidota	*Aquimarina gracilis*	WP_324180621.1	WP_324180101.1	WP_324178525.1
Bacteroidota	*Aquimarina intermedia*	WP_148782085.1	WP_148782431.1	WP_148783651.1
Bacteroidota	*Aquimarina litoralis*	WP_219008440.1	WP_219009007.1	WP_219010050.1
Bacteroidota	*Aquimarina longa*	WP_062057664.1	WP_062058925.1	WP_062059664.1
Bacteroidota	*Aquimarina macrocephali*	WP_024770031.1	WP_024771131.1	WP_024771276.1
Bacteroidota	*Aquimarina muelleri*	WP_027411125.1	WP_027411711.1	WP_027411300.1
Bacteroidota	*Aquimarina mytili*	WP_201917632.1	WP_201924142.1	WP_201922323.1
Bacteroidota	*Aquimarina pacifica*	WP_025742060.1	WP_025743084.1	WP_025742517.1
Bacteroidota	*Aquimarina rubra*	WP_378290376.1	WP_378288455.1	WP_378295432.1
Bacteroidota	*Aquimarina sediminis*	WP_103068351.1	WP_103070573.1	WP_103070728.1
Bacteroidota	*Aquimarina sp. I32.4*	WP_103863893.1	WP_103864881.1	WP_103866837.1
Bacteroidota	*Aquimarina spongiae*	WP_073314409.1	WP_073314355.1	WP_073320496.1
Bacteroidota	*Aquirufa antheringensis*	WP_130922775.1	WP_130923145.1	WP_269012307.1
Bacteroidota	*Aquirufa aurantiipilula*	WP_276344163.1	WP_223142429.1	WP_223143335.1
Bacteroidota	*Aquirufa avitistagni*	WP_377983723.1	WP_377982913.1	WP_377983456.1
Bacteroidota	*Aquirufa beregesia*	WP_166230096.1	WP_166228982.1	WP_166229514.1
Bacteroidota	*Aquirufa ecclesiirivi*	WP_275612994.1	WP_166375689.1	WP_275612331.1
Bacteroidota	*Aquirufa echingensis*	WP_377976744.1	WP_377976516.1	WP_377974603.1
Bacteroidota	*Aquirufa lenticrescens*	WP_223131088.1	WP_223130902.1	WP_223129962.1
Bacteroidota	*Aquirufa nivalisilvae*	WP_269013300.1	WP_269024951.1	WP_109324451.1
Bacteroidota	*Aquirufa novilacunae*	WP_406800710.1	WP_406776800.1	WP_406800572.1
Bacteroidota	*Aquirufa originis*	WP_377979066.1	WP_377978324.1	WP_377978916.1

Bacteroidota	*Aquirufa regiilacus*	WP_315576891.1	WP_315575113.1	WP_315574784.1
Bacteroidota	*Aquirufa rosea*	WP_129026517.1	WP_129027631.1	WP_129027468.1
Bacteroidota	*Aquirufa salirivi*	WP_406749496.1	WP_406751867.1	WP_406750838.1
Bacteroidota	*Aquirufa sp.*	WP_395767743.1	WP_395785771.1	WP_395785934.1
Bacteroidota	*Arachidicoccus ginsenosidivorans*	WP_146779924.1	WP_146780403.1	WP_158639367.1
Bacteroidota	*Arachidicoccus rhizosphaerae*	WP_091397094.1	WP_091395310.1	WP_091399636.1
Bacteroidota	*Arachidicoccus soli*	WP_119987445.1	WP_119989760.1	WP_119984939.1
Bacteroidota	*Arachidicoccus sp. BS20*	WP_066202682.1	WP_066206247.1	WP_066208570.1
Bacteroidota	*Arachidicoccus terrestris*	WP_224070549.1	WP_224071115.1	WP_224070363.1
Bacteroidota	*Arcicella aquatica*	WP_323246239.1	WP_323249301.1	WP_323247214.1
Bacteroidota	*Arcicella aurantiaca*	WP_109743205.1	WP_109741526.1	WP_109744417.1
Bacteroidota	*Arcicella gelida*	WP_323328161.1	WP_323697626.1	WP_323697301.1
Bacteroidota	*Arcicella rigui*	WP_323297640.1	WP_323296787.1	WP_323294779.1
Bacteroidota	*Arcicella rosea*	WP_184128752.1	WP_184134267.1	WP_367285748.1
Bacteroidota	*Arcicella sp. LKC2W*	WP_323347125.1	WP_323345881.1	WP_323348259.1
Bacteroidota	*Arcticibacter eurypsychrophilus*	WP_069660197.1	WP_069658220.1	WP_069658143.1
Bacteroidota	*Arcticibacter pallidicorallinus*	WP_106295235.1	WP_106293321.1	WP_106293700.1
Bacteroidota	*Arcticibacter sp.*	WP_407426349.1	WP_407428255.1	WP_407430250.1
Bacteroidota	*Arcticibacter svalbardensis*	WP_016197349.1	WP_016193496.1	WP_016195818.1
Bacteroidota	*Arcticibacter tournemirensis*	WP_141816717.1	WP_128769017.1	WP_141816323.1
Bacteroidota	*Arcticibacterium luteifluviistationis*	WP_111371990.1	WP_111374148.1	WP_111373335.1
Bacteroidota	*Arenibacter algicola*	WP_093979083.1	WP_215950131.1	WP_142189624.1
Bacteroidota	*Arenibacter amylolyticus*	WP_086478617.1	WP_086478264.1	WP_086477215.1
Bacteroidota	*Arenibacter aquaticus*	WP_126161809.1	WP_126163894.1	WP_126162343.1
Bacteroidota	*Arenibacter arenosicollis*	WP_187583026.1	WP_187584347.1	WP_187584983.1
Bacteroidota	*Arenibacter certesii*	WP_026815150.1	WP_026812673.1	WP_026812862.1
Bacteroidota	*Arenibacter echinorum*	WP_111622257.1	WP_111623164.1	WP_111621924.1
Bacteroidota	*Arenibacter lacus*	WP_150452474.1	WP_150451320.1	WP_150450833.1

Bacteroidota	*Arenibacter latericius*	WP_026811139.1	WP_026810798.1	WP_026809646.1
Bacteroidota	*Arenibacter nanhaiticus*	WP_072764415.1	WP_072765535.1	WP_072763073.1
Bacteroidota	*Arenibacter palladensis*	WP_332927960.1	WP_303585876.1	WP_072862337.1
Bacteroidota	*Arenibacter sp. GZD-96*	WP_322931903.1	WP_322930698.1	WP_322929451.1
Bacteroidota	*Arenibacter troitsensis*	WP_085497318.1	WP_085500282.1	WP_085495928.1
Bacteroidota	*Aridibaculum aurantiacum*	WP_207496171.1	WP_207493540.1	WP_207493862.1
Bacteroidota	*Arsenicibacter rosenii*	WP_071502120.1	WP_071502670.1	WP_071505339.1
Bacteroidota	*Arthrospiribacter ruber*	WP_219291266.1	WP_219289258.1	WP_219288141.1
Bacteroidota	*Arundinibacter roseus*	WP_132114301.1	WP_132116095.1	WP_132121505.1
Bacteroidota	*Ascidiimonas aurantiaca*	WP_340063238.1	WP_340065951.1	WP_340063664.1
Bacteroidota	*Ascidiimonas meishanensis*	WP_340202346.1	WP_340201815.1	WP_340203336.1
Bacteroidota	*Asinibacterium sp. OR53*	WP_026768116.1	WP_026770043.1	WP_026770422.1
Bacteroidota	*Asprobacillus argus*	WP_349240976.1	WP_349241222.1	WP_349242390.1
Bacteroidota	*Aurantibacillus circumpalustris*	WP_317897266.1	WP_317899147.1	WP_317898617.1
Bacteroidota	*Aurantibacter aestuarii*	WP_106463421.1	WP_106463824.1	WP_106463759.1
Bacteroidota	*Aurantibacter crassamenti*	WP_203252934.1	WP_203253053.1	WP_203254514.1
Bacteroidota	*Aurantibacter sp.*	WP_348148950.1	WP_375239853.1	WP_375239007.1
Bacteroidota	*Aureibacter tunicatorum*	WP_309937555.1	WP_309941955.1	WP_309937614.1
Bacteroidota	*Aureibaculum algae*	WP_138950357.1	WP_138950222.1	WP_138948862.1
Bacteroidota	*Aureibaculum flavum*	WP_198839813.1	WP_198839693.1	WP_198842913.1
Bacteroidota	*Aureibaculum luteum*	WP_117883329.1	WP_117883048.1	WP_117885144.1
Bacteroidota	*Aureibaculum marinum*	WP_123896962.1	WP_123897081.1	WP_123896527.1
Bacteroidota	*Aureibaculum sp. 2210JD6-5*	WP_321974792.1	WP_321976430.1	WP_321975147.1
Bacteroidota	*Aureicoccus marinus*	WP_105000905.1	WP_105000782.1	WP_105000272.1
Bacteroidota	*Aureisphaera galaxeae*	WP_272862417.1	WP_272863078.1	WP_272861625.1
Bacteroidota	*Aureispira anguillae*	WP_264788426.1	WP_264791552.1	WP_264791511.1
Bacteroidota	*Aureitalea sp. L0-47*	WP_265198881.1	WP_265199191.1	WP_265200177.1
Bacteroidota	*Aureivirga marina*	WP_196892692.1	WP_196894274.1	WP_196894243.1

Bacteroidota	*Aureivirga sp. CE67*	WP_196888611.1	WP_196888714.1	WP_196887798.1
Bacteroidota	*Autumnicola edwardsiae*	WP_311484892.1	WP_311485020.1	WP_311484481.1
Bacteroidota	*Autumnicola lenta*	WP_311495763.1	WP_311493373.1	WP_311493415.1
Bacteroidota	*Autumnicola musiva*	WP_311503703.1	WP_311502472.1	WP_311502434.1
Bacteroidota	*Autumnicola patrickiae*	WP_311684452.1	WP_311679862.1	WP_311679937.1
Bacteroidota	*Autumnicola psychrophila*	WP_311499815.1	WP_311499532.1	WP_311500054.1
Bacteroidota	*Autumnicola tepida*	WP_311533564.1	WP_311534518.1	WP_311535150.1
Bacteroidota	*Avrilella dinanensis*	WP_277631760.1	WP_100677930.1	WP_100678779.1
Bacteroidota	*Belliella alkalica*	WP_241411179.1	WP_241409255.1	WP_241411311.1
Bacteroidota	*Belliella aquatica*	WP_188442687.1	WP_188439546.1	WP_188439325.1
Bacteroidota	*Belliella baltica*	WP_014772208.1	WP_014773237.1	WP_014771954.1
Bacteroidota	*Belliella buryatensis*	WP_089237558.1	WP_089239844.1	WP_089240194.1
Bacteroidota	*Belliella calami*	WP_241276453.1	WP_241275745.1	WP_241275082.1
Bacteroidota	*Belliella filtrata*	WP_241347112.1	WP_241346774.1	WP_241350002.1
Bacteroidota	*Belliella kenyensis*	WP_241296136.1	WP_241291243.1	WP_241292937.1
Bacteroidota	*Belliella marina*	WP_376886587.1	WP_376887078.1	WP_376886810.1
Bacteroidota	*Belliella pelovolcani*	WP_409629045.1	WP_076502952.1	WP_076502588.1
Bacteroidota	*Bergeyella cardium*	WP_120489413.1	WP_160223991.1	WP_160224032.1
Bacteroidota	*Bergeyella porcorum*	WP_327985048.1	WP_327984028.1	WP_376832879.1
Bacteroidota	*Bergeyella sp. RCAD1439*	WP_327609899.1	WP_327610773.1	WP_327610132.1
Bacteroidota	*Bergeyella zoohelcum*	WP_125150706.1	WP_002686932.1	WP_002689017.1
Bacteroidota	*Bernardetia litoralis*	WP_014799449.1	WP_014796014.1	WP_014797464.1
Bacteroidota	*Bernardetia sp.*	WP_291727868.1	WP_291727328.1	WP_291721233.1
Bacteroidota	*Bizionia arctica*	WP_188463855.1	WP_188463365.1	WP_188465515.1
Bacteroidota	*Bizionia argentinensis*	WP_008634538.1	WP_008636439.1	WP_008637444.1
Bacteroidota	*Bizionia echini*	WP_092207375.1	WP_339635095.1	WP_092209708.1
Bacteroidota	*Bizionia hallyeonensis*	WP_376859852.1	WP_376859305.1	WP_376860208.1
Bacteroidota	*Bizionia myxarmorum*	WP_148404171.1	WP_148405154.1	WP_148403799.1

Bacteroidota	*Bizionia paragorgiae*	WP_318908187.1	WP_092135828.1	WP_417236466.1
Bacteroidota	*Bizionia sediminis*	WP_376891938.1	WP_376891554.1	WP_376891096.1
Bacteroidota	*Bizionia sp.*	WP_417195014.1	WP_417199749.1	WP_417238594.1
Bacteroidota	*Botryobacter ruber*	WP_114781647.1	WP_114781543.1	WP_240676079.1
Bacteroidota	*Brumimicrobium aurantiacum*	WP_116880684.1	WP_116880515.1	WP_116881141.1
Bacteroidota	*Brumimicrobium glaciale*	WP_130094022.1	WP_242494536.1	WP_165366249.1
Bacteroidota	*Brumimicrobium mesophilum*	WP_107040258.1	WP_107039225.1	WP_107039918.1
Bacteroidota	*Brumimicrobium oceani*	WP_109358790.1	WP_233244208.1	WP_109360517.1
Bacteroidota	*Brumimicrobium salinarum*	WP_101335045.1	WP_101334281.1	WP_101335294.1
Bacteroidota	*Brumimicrobium sp.*	WP_417265319.1	WP_417266280.1	WP_417265785.1
Bacteroidota	*Candidatus Arcticimaribacter forsetii*	WP_252158231.1	WP_252158092.1	WP_252158673.1
Bacteroidota	*Candidatus Brachybacter algidus*	WP_287401746.1	WP_287400426.1	WP_287402622.1
Bacteroidota	*Candidatus Kaistella beijingensis*	WP_224135010.1	WP_224134386.1	WP_224134929.1
Bacteroidota	*Candidatus Ornithobacterium hominis*	WP_119058188.1	WP_119058084.1	WP_119058210.1
Bacteroidota	*Candidatus Ulvibacter alkanivorans*	WP_114491340.1	WP_114491714.1	WP_114491961.1
Bacteroidota	*Capnocytophaga bilenii*	WP_208058769.1	WP_288898588.1	WP_208059362.1
Bacteroidota	*Capnocytophaga canimorsus*	WP_095918297.1	WP_212891728.1	WP_212901490.1
Bacteroidota	*Capnocytophaga canis*	WP_119653065.1	WP_172919092.1	WP_042347325.1
Bacteroidota	*Capnocytophaga catalasegens*	WP_264846145.1	WP_264846002.1	WP_264846610.1
Bacteroidota	*Capnocytophaga cynodegmi*	WP_405251651.1	WP_238253373.1	WP_018278526.1
Bacteroidota	*Capnocytophaga felis*	WP_155285449.1	WP_155284653.1	WP_155284893.1
Bacteroidota	*Capnocytophaga genosp. AHN8471*	WP_203093402.1	WP_203080770.1	WP_203081813.1
Bacteroidota	*Capnocytophaga gingivalis*	WP_273084178.1	WP_424651423.1	WP_095911379.1
Bacteroidota	*Capnocytophaga granulosa*	WP_424655771.1	WP_314810622.1	WP_424656280.1
Bacteroidota	*Capnocytophaga haemolytica*	WP_066429520.1	WP_066429263.1	WP_066430160.1
Bacteroidota	*Capnocytophaga leadbetteri*	WP_095912921.1	WP_304348063.1	WP_311338546.1
Bacteroidota	*Capnocytophaga ochracea*	WP_264854235.1	WP_002676140.1	WP_264852541.1
Bacteroidota	*Capnocytophaga periodontitidis*	WP_198472540.1	WP_198475329.1	WP_198472515.1

Bacteroidota	*Capnocytophaga sp.*	WP_316374321.1	WP_303076861.1	WP_308766617.1
Bacteroidota	*Capnocytophaga sputigena*	WP_424653469.1	WP_314278883.1	WP_002681527.1
Bacteroidota	*Capnocytophaga stomatis*	WP_095894594.1	WP_405254939.1	WP_203969405.1
Bacteroidota	*Catalinimonas alkaloidigena*	WP_089681298.1	WP_089683750.1	WP_277486639.1
Bacteroidota	*Catalinimonas niigatensis*	WP_302248781.1	WP_302253366.1	WP_302248794.1
Bacteroidota	*Catalinimonas sp. 4WD22*	WP_341296119.1	WP_341294730.1	WP_341297279.1
Bacteroidota	*Cecembia calidifontis*	WP_130274964.1	WP_130276585.1	WP_130274130.1
Bacteroidota	*Cecembia lonarensis*	WP_009186108.1	WP_009185929.1	WP_009186875.1
Bacteroidota	*Cecembia rubra*	WP_106568491.1	WP_106565721.1	WP_106568070.1
Bacteroidota	*Cecembia sp.*	WP_291784869.1	WP_291778684.1	WP_291784052.1
Bacteroidota	*Cellulophaga baltica*	WP_025614771.1	WP_282164778.1	WP_025614484.1
Bacteroidota	*Cellulophaga fucicola*	WP_072301909.1	WP_072302457.1	WP_072302783.1
Bacteroidota	*Cellulophaga lytica*	WP_303519488.1	WP_038506972.1	WP_075694236.1
Bacteroidota	*Cellulophaga omnivescoria*	WP_077402429.1	WP_271081074.1	WP_077398634.1
Bacteroidota	*Cellulophaga sp. HaHaR_3_176*	WP_216785145.1	WP_216785278.1	WP_216784904.1
Bacteroidota	*Cellulophaga tyrosinoxydans*	WP_084061103.1	WP_084060995.1	WP_084062724.1
Bacteroidota	*Cerina litoralis*	WP_317902400.1	WP_317902167.1	WP_317901971.1
Bacteroidota	*Cesiribacter sp. SM1*	WP_224999480.1	WP_224998922.1	WP_224999441.1
Bacteroidota	*Changchengzhania lutea*	WP_142785956.1	WP_142783246.1	WP_142783659.1
Bacteroidota	*Chishuiella changwenlii*	WP_072931684.1	WP_333662326.1	WP_333660450.1
Bacteroidota	*Chishuiella sp.*	WP_313375460.1	WP_313373552.1	WP_313579345.1
Bacteroidota	*Chitinophaga agri*	WP_162333742.1	WP_162331142.1	WP_238430125.1
Bacteroidota	*Chitinophaga agrisoli*	WP_149836969.1	WP_149837932.1	WP_149840340.1
Bacteroidota	*Chitinophaga alhagiae*	WP_119077216.1	WP_119079661.1	WP_157986662.1
Bacteroidota	*Chitinophaga arvensicola*	WP_089889289.1	WP_089896020.1	WP_177192308.1
Bacteroidota	*Chitinophaga barathri*	WP_120518200.1	WP_120514463.1	WP_162946241.1
Bacteroidota	*Chitinophaga caeni*	WP_098192515.1	WP_098194845.1	WP_157760992.1
Bacteroidota	*Chitinophaga caseinilytica*	WP_341842633.1	WP_341839141.1	WP_341840093.1

Bacteroidota	*Chitinophaga costaii*	WP_089715512.1	WP_089710726.1	WP_089715615.1
Bacteroidota	*Chitinophaga cymbidii*	WP_146858207.1	WP_146866479.1	WP_186831128.1
Bacteroidota	*Chitinophaga defluvii*	WP_354661261.1	WP_354660547.1	WP_354659270.1
Bacteroidota	*Chitinophaga deserti*	WP_109694755.1	WP_109699103.1	WP_157962811.1
Bacteroidota	*Chitinophaga dinghuensis*	WP_111591532.1	WP_111592684.1	WP_111595453.1
Bacteroidota	*Chitinophaga eiseniae*	WP_078670110.1	WP_078670884.1	WP_078668293.1
Bacteroidota	*Chitinophaga filiformis*	WP_247813009.1	WP_089836010.1	WP_089830986.1
Bacteroidota	*Chitinophaga flava*	WP_113616485.1	WP_113614148.1	WP_113618438.1
Bacteroidota	*Chitinophaga fulva*	WP_169222926.1	WP_169225324.1	WP_169227571.1
Bacteroidota	*Chitinophaga ginsengisoli*	WP_106600515.1	WP_106605529.1	WP_106600880.1
Bacteroidota	*Chitinophaga horti*	WP_244842465.1	WP_264282605.1	WP_264282541.1
Bacteroidota	*Chitinophaga hostae*	WP_211973759.1	WP_211974175.1	WP_211973914.1
Bacteroidota	*Chitinophaga japonensis*	WP_145711536.1	WP_145715336.1	WP_145717070.1
Bacteroidota	*Chitinophaga jiangningensis*	WP_073084114.1	WP_073086198.1	WP_073087053.1
Bacteroidota	*Chitinophaga lutea*	WP_123845536.1	WP_123849538.1	WP_158618180.1
Bacteroidota	*Chitinophaga niabensis*	WP_343304213.1	WP_343308041.1	WP_343308270.1
Bacteroidota	*Chitinophaga niastensis*	WP_106526122.1	WP_106528908.1	WP_106530351.1
Bacteroidota	*Chitinophaga nivalis*	WP_264735150.1	WP_264728856.1	WP_264732223.1
Bacteroidota	*Chitinophaga oryzae*	WP_168807646.1	WP_168802561.1	WP_168804602.1
Bacteroidota	*Chitinophaga oryziterrae*	WP_157302517.1	WP_157302627.1	WP_157300745.1
Bacteroidota	*Chitinophaga parva*	WP_108684680.1	WP_108687857.1	WP_108687548.1
Bacteroidota	*Chitinophaga pinensis*	WP_012788863.1	WP_146306657.1	WP_012792326.1
Bacteroidota	*Chitinophaga pollutisoli*	WP_341838311.1	WP_341836676.1	WP_341835840.1
Bacteroidota	*Chitinophaga polysaccharea*	WP_145660751.1	WP_142685119.1	WP_142688553.1
Bacteroidota	*Chitinophaga qingshengii*	WP_188091016.1	WP_188086724.1	WP_188091470.1
Bacteroidota	*Chitinophaga rhizophila*	WP_220251837.1	WP_220253029.1	WP_220252641.1
Bacteroidota	*Chitinophaga rhizosphaerae*	WP_126245588.1	WP_126249185.1	WP_164714245.1
Bacteroidota	*Chitinophaga rupis*	WP_089920443.1	WP_089919493.1	WP_089909602.1

Bacteroidota	*Chitinophaga sancti*	WP_320580275.1	WP_320572784.1	WP_072357357.1
Bacteroidota	*Chitinophaga silvatica*	WP_116975180.1	WP_116973669.1	WP_116976693.1
Bacteroidota	*Chitinophaga silvisoli*	WP_116853605.1	WP_116854710.1	WP_116854545.1
Bacteroidota	*Chitinophaga skermanii*	WP_111595753.1	WP_111597493.1	WP_111596564.1
Bacteroidota	*Chitinophaga solisilvae*	WP_127037710.1	WP_160712735.1	WP_160716070.1
Bacteroidota	*Chitinophaga sp.*	WP_326461457.1	WP_343703673.1	WP_291942926.1
Bacteroidota	*Chitinophaga terrae (ex Kim and Jung 2007)*	WP_089758629.1	WP_089759461.1	WP_089760460.1
Bacteroidota	*Chitinophaga tropicalis*	WP_157304928.1	WP_157309866.1	WP_157306277.1
Bacteroidota	*Chitinophaga varians*	WP_188096056.1	WP_188101673.1	WP_168873033.1
Bacteroidota	*Chitinophaga vietnamensis*	WP_143310755.1	WP_143310472.1	WP_143306111.1
Bacteroidota	*Chondrinema litorale*	WP_284682294.1	WP_284684089.1	WP_284684822.1
Bacteroidota	*Christiangramia aestuarii*	WP_156276367.1	WP_156277420.1	WP_156273350.1
Bacteroidota	*Christiangramia aquimixticola*	WP_417907117.1	WP_417907863.1	WP_417905913.1
Bacteroidota	*Christiangramia crocea*	WP_240099472.1	WP_240100482.1	WP_240100745.1
Bacteroidota	*Christiangramia echinicola*	WP_089663554.1	WP_026934815.1	WP_089662858.1
Bacteroidota	*Christiangramia flava*	WP_083645604.1	WP_083645022.1	WP_083644987.1
Bacteroidota	*Christiangramia forsetii*	WP_011709514.1	WP_011708676.1	WP_011710100.1
Bacteroidota	*Christiangramia fulva*	WP_107013167.1	WP_107012684.1	WP_107012643.1
Bacteroidota	*Christiangramia gaetbulicola*	WP_108171519.1	WP_108172085.1	WP_108172706.1
Bacteroidota	*Christiangramia lutea*	WP_240713313.1	WP_240714481.1	WP_240713769.1
Bacteroidota	*Christiangramia marina*	WP_417920970.1	WP_417921409.1	WP_417920483.1
Bacteroidota	*Christiangramia oceanisediminis*	WP_241551229.1	WP_241552613.1	WP_241552703.1
Bacteroidota	*Christiangramia portivictoriae*	WP_026916233.1	WP_026914858.1	WP_026915542.1
Bacteroidota	*Christiangramia sabulilitoris*	WP_143409212.1	WP_143409686.1	WP_143411055.1
Bacteroidota	*Christiangramia salexigens*	WP_072552188.1	WP_072551724.1	WP_072552596.1
Bacteroidota	*Christiangramia sediminis*	WP_229337945.1	WP_229337042.1	WP_229339163.1
Bacteroidota	*Christiangramia sp.*	WP_300437579.1	WP_347410098.1	WP_300434533.1

Bacteroidota	*Chryseobacterium angstadtii*	WP_048504691.1	WP_048508403.1	WP_048504839.1
Bacteroidota	*Chryseobacterium aquaeductus*	WP_162087455.1	WP_162088291.1	WP_162087563.1
Bacteroidota	*Chryseobacterium aquaticum*	WP_332453612.1	WP_332454044.1	WP_332453903.1
Bacteroidota	*Chryseobacterium arachidis*	WP_072953422.1	WP_072962042.1	WP_072953694.1
Bacteroidota	*Chryseobacterium arthrosphaerae*	WP_065401126.1	WP_065399462.1	WP_278377839.1
Bacteroidota	*Chryseobacterium artocarpi*	WP_065393438.1	WP_065395308.1	WP_065395850.1
Bacteroidota	*Chryseobacterium aureum*	WP_126653405.1	WP_126652494.1	WP_126653278.1
Bacteroidota	*Chryseobacterium binzhouense*	WP_143884827.1	WP_312344148.1	WP_143885655.1
Bacteroidota	*Chryseobacterium camelliae*	WP_100076556.1	WP_100075739.1	WP_100076441.1
Bacteroidota	*Chryseobacterium carnipullorum*	WP_073335484.1	WP_073329474.1	WP_123877096.1
Bacteroidota	*Chryseobacterium cheonjiense*	WP_169231258.1	WP_169232082.1	WP_169231368.1
Bacteroidota	*Chryseobacterium contaminans*	WP_066699585.1	WP_336963978.1	WP_336962821.1
Bacteroidota	*Chryseobacterium culicis*	WP_193546784.1	WP_105681681.1	WP_089692756.1
Bacteroidota	*Chryseobacterium daecheongense*	WP_123263640.1	WP_245143878.1	WP_245142849.1
Bacteroidota	*Chryseobacterium daeguense*	WP_027380310.1	WP_027381351.1	WP_027380381.1
Bacteroidota	*Chryseobacterium defluvii*	WP_184192087.1	WP_121459854.1	WP_121462258.1
Bacteroidota	*Chryseobacterium echinoideorum*	WP_144283498.1	WP_144281767.1	WP_144281870.1
Bacteroidota	*Chryseobacterium edaphi*	WP_263004635.1	WP_263003815.1	WP_263003315.1
Bacteroidota	*Chryseobacterium endalhagicum*	WP_202092377.1	WP_202088381.1	WP_202092137.1
Bacteroidota	*Chryseobacterium flavum*	WP_115962526.1	WP_115959980.1	WP_333596648.1
Bacteroidota	*Chryseobacterium fluminis*	WP_266173542.1	WP_266172508.1	WP_266173392.1
Bacteroidota	*Chryseobacterium foetidum*	WP_262150941.1	WP_262149356.1	WP_262150698.1
Bacteroidota	*Chryseobacterium formosense*	WP_034678117.1	WP_034679053.1	WP_034677951.1
Bacteroidota	*Chryseobacterium formosus*	WP_267266397.1	WP_267266997.1	WP_267266551.1
Bacteroidota	*Chryseobacterium gambrini*	WP_421744305.1	WP_421745152.1	WP_271160214.1
Bacteroidota	*Chryseobacterium gilvum*	WP_262989998.1	WP_262991445.1	WP_262990122.1
Bacteroidota	*Chryseobacterium ginsengisoli*	WP_345204417.1	WP_345207700.1	WP_345204007.1
Bacteroidota	*Chryseobacterium ginsenosidimutans*	WP_306617673.1	WP_344823491.1	WP_344828136.1

Bacteroidota	*Chryseobacterium gleum*	WP_002981824.1	WP_002976287.1	WP_002982977.1
Bacteroidota	*Chryseobacterium gotjawalense*	WP_282903957.1	WP_282903963.1	WP_282903911.1
Bacteroidota	*Chryseobacterium gregarium*	WP_027387127.1	WP_027387487.1	WP_027387596.1
Bacteroidota	*Chryseobacterium group*	WP_063971113.1	WP_063971246.1	WP_063971373.1
Bacteroidota	*Chryseobacterium gwangjuense*	WP_233112162.1	WP_233112332.1	WP_233112287.1
Bacteroidota	*Chryseobacterium hagamense*	WP_146939999.1	WP_146942032.1	WP_146939862.1
Bacteroidota	*Chryseobacterium herbae*	WP_259839341.1	WP_259838793.1	WP_259839482.1
Bacteroidota	*Chryseobacterium indologenes*	WP_213277952.1	WP_061084393.1	WP_213277829.1
Bacteroidota	*Chryseobacterium jejuense*	WP_209919674.1	WP_209916422.1	WP_209918947.1
Bacteroidota	*Chryseobacterium koreense*	WP_304343474.1	WP_304343482.1	WP_304343634.1
Bacteroidota	*Chryseobacterium kwangjuense*	WP_062650765.1	WP_062650258.1	WP_409358204.1
Bacteroidota	*Chryseobacterium lactis*	WP_047098204.1	WP_185288522.1	WP_103289094.1
Bacteroidota	*Chryseobacterium lacus*	WP_114304519.1	WP_114302695.1	WP_114303026.1
Bacteroidota	*Chryseobacterium lathyri*	WP_307211175.1	WP_306845405.1	WP_111956929.1
Bacteroidota	*Chryseobacterium limigenitum*	WP_072407513.1	WP_072411150.1	WP_072407276.1
Bacteroidota	*Chryseobacterium luquanense*	WP_267280638.1	WP_267282236.1	WP_267280772.1
Bacteroidota	*Chryseobacterium luteum*	WP_034702980.1	WP_034701920.1	WP_034702712.1
Bacteroidota	*Chryseobacterium manosquense*	WP_123248881.1	WP_188322246.1	WP_188320621.1
Bacteroidota	*Chryseobacterium mucoviscidosis*	WP_087707876.1	WP_336717035.1	WP_087706563.1
Bacteroidota	*Chryseobacterium muglaense*	WP_191178277.1	WP_191179160.1	WP_191178394.1
Bacteroidota	*Chryseobacterium nakagawai*	WP_123858886.1	WP_123857931.1	WP_123858751.1
Bacteroidota	*Chryseobacterium nepalense*	WP_248390388.1	WP_326988922.1	WP_326988498.1
Bacteroidota	*Chryseobacterium oleae*	WP_090025259.1	WP_090025667.1	WP_090025098.1
Bacteroidota	*Chryseobacterium oranimense*	WP_260540510.1	WP_260542181.1	WP_040993467.1
Bacteroidota	*Chryseobacterium oryctis*	WP_264743935.1	WP_264742216.1	WP_264744072.1
Bacteroidota	*Chryseobacterium panacisoli*	WP_047382874.1	WP_149387241.1	WP_149387698.1
Bacteroidota	*Chryseobacterium paridis*	WP_200241446.1	WP_200248911.1	WP_200241814.1
Bacteroidota	*Chryseobacterium pennipullorum*	WP_115929148.1	WP_115928623.1	WP_115927319.1

Bacteroidota	*Chryseobacterium phocaeense*	WP_080777849.1	WP_080776683.1	WP_080777716.1
Bacteroidota	*Chryseobacterium phosphatilyticum*	WP_103248064.1	WP_103247118.1	WP_109713458.1
Bacteroidota	*Chryseobacterium piperi*	WP_034687838.1	WP_034681116.1	WP_034680385.1
Bacteroidota	*Chryseobacterium piscicola*	WP_076449729.1	WP_076452144.1	WP_076449478.1
Bacteroidota	*Chryseobacterium polytrichastri*	WP_073295856.1	WP_073293519.1	WP_073296113.1
Bacteroidota	*Chryseobacterium populi*	WP_007846994.1	WP_034669435.1	WP_007847155.1
Bacteroidota	*Chryseobacterium profundimaris*	WP_283421978.1	WP_283422706.1	WP_283421026.1
Bacteroidota	*Chryseobacterium salviniae*	WP_326320873.1	WP_326319633.1	WP_326321868.1
Bacteroidota	*Chryseobacterium scophthalmum*	WP_427317969.1	WP_210150477.1	WP_427313732.1
Bacteroidota	*Chryseobacterium sediminis*	WP_309876686.1	WP_184557784.1	WP_184558456.1
Bacteroidota	*Chryseobacterium shandongense*	WP_123852675.1	WP_123854981.1	WP_123854495.1
Bacteroidota	*Chryseobacterium shigense*	WP_184164982.1	WP_076508834.1	WP_076507142.1
Bacteroidota	*Chryseobacterium soldanellicola*	WP_089754460.1	WP_089756315.1	WP_089754742.1
Bacteroidota	*Chryseobacterium sp.*	WP_312396624.1	WP_375180805.1	WP_317132566.1
Bacteroidota	*Chryseobacterium suipulveris*	WP_243548920.1	WP_243548777.1	WP_243550068.1
Bacteroidota	*Chryseobacterium taeanense*	WP_374458709.1	WP_374460750.1	WP_089856336.1
Bacteroidota	*Chryseobacterium tagetis*	WP_225686926.1	WP_225690509.1	WP_225687090.1
Bacteroidota	*Chryseobacterium taichungense*	WP_089999797.1	WP_312899911.1	WP_312902982.1
Bacteroidota	*Chryseobacterium taihuense*	WP_089744917.1	WP_089744036.1	WP_089741825.1
Bacteroidota	*Chryseobacterium taiwanense*	WP_039364300.1	WP_039365139.1	WP_039364551.1
Bacteroidota	*Chryseobacterium takakiae*	WP_072883998.1	WP_072883461.1	WP_072883882.1
Bacteroidota	*Chryseobacterium taklimakanense*	WP_095071275.1	WP_095071556.1	WP_277111743.1
Bacteroidota	*Chryseobacterium terrae*	WP_408090487.1	WP_408091166.1	WP_408090916.1
Bacteroidota	*Chryseobacterium tructae*	WP_290301454.1	WP_290295476.1	WP_290301759.1
Bacteroidota	*Chryseobacterium turcicum*	WP_230672020.1	WP_230667182.1	WP_230672276.1
Bacteroidota	*Chryseobacterium ureilyticum*	WP_076553735.1	WP_076551667.1	WP_076552180.1
Bacteroidota	*Chryseobacterium vietnamense*	WP_047429091.1	WP_310405478.1	WP_310404856.1
Bacteroidota	*Chryseobacterium vrystaatense*	WP_034738641.1	WP_034746935.1	WP_034738968.1

Bacteroidota	*Chryseobacterium wanjuense*	WP_089793124.1	WP_089795693.1	WP_089793430.1
Bacteroidota	*Chryseobacterium zhengzhouense*	WP_378177090.1	WP_378181010.1	WP_378176574.1
Bacteroidota	*Chryseolinea lacunae*	WP_202010734.1	WP_202013028.1	WP_202008930.1
Bacteroidota	*Chryseolinea serpens*	WP_073133656.1	WP_073130757.1	WP_073134550.1
Bacteroidota	*Chryseolinea soli*	WP_119754613.1	WP_119757032.1	WP_119754129.1
Bacteroidota	*Chryseolinea sp. H1M3-3*	WP_276373192.1	WP_276374865.1	WP_276368923.1
Bacteroidota	*Chryseosolibacter histidini*	WP_254170009.1	WP_254162248.1	WP_254168819.1
Bacteroidota	*Chryseosolibacter indicus*	WP_254155073.1	WP_254151535.1	WP_254152595.1
Bacteroidota	*Chryseotalea sanaruensis*	WP_127124308.1	WP_127123771.1	WP_127123286.1
Bacteroidota	*Cloacibacterium caeni*	WP_213197414.1	WP_213196799.1	WP_213196929.1
Bacteroidota	*Cloacibacterium normanense*	WP_371488702.1	WP_409623128.1	WP_069800177.1
Bacteroidota	*Cloacibacterium rupense*	WP_188618261.1	WP_188616825.1	WP_229663381.1
Bacteroidota	*Cloacibacterium sp.*	WP_424305291.1	WP_424304899.1	WP_414982983.1
Bacteroidota	*Cnuella takakiae*	WP_073048635.1	WP_073047240.1	WP_073046215.1
Bacteroidota	*Cochleicola gelatinilyticus*	WP_068592651.1	WP_068591820.1	WP_068588824.1
Bacteroidota	*Cognataquiflexum aquatile*	WP_113923999.1	WP_113922158.1	WP_113923884.1
Bacteroidota	*Cognataquiflexum nitidum*	WP_241124226.1	WP_241126391.1	WP_241124347.1
Bacteroidota	*Cognataquiflexum rubidum*	WP_241139927.1	WP_241143543.1	WP_241140045.1
Bacteroidota	*Cognatitamlana onchidii*	WP_136481225.1	WP_136481392.1	WP_136480656.1
Bacteroidota	*Compostibacter hankyongensis*	WP_344975939.1	WP_344977829.1	WP_344977805.1
Bacteroidota	*Confluentibacter citreus*	WP_100614548.1	WP_100614611.1	WP_100615531.1
Bacteroidota	*Confluentibacter flavum*	WP_106660758.1	WP_106658464.1	WP_106659928.1
Bacteroidota	*Confluentibacter lentus*	WP_100611433.1	WP_100611373.1	WP_100612254.1
Bacteroidota	*Confluentibacter sediminis*	WP_111307457.1	WP_111307398.1	WP_111309144.1
Bacteroidota	*Constantimarinum furrinae*	WP_186988759.1	WP_186989389.1	WP_186987917.1
Bacteroidota	*Corallibacter sp.*	WP_417290984.1	WP_418509762.1	WP_418509659.1
Bacteroidota	*Costertonia aggregata*	WP_179242954.1	WP_179243042.1	WP_179242674.1
Bacteroidota	*Croceibacter atlanticus*	WP_348661630.1	WP_341200636.1	WP_348661122.1

Bacteroidota	*Croceimicrobium hydrocarbonivorans*	WP_210758119.1	WP_210758057.1	WP_210758765.1
Bacteroidota	*Croceimicrobium sp.*	WP_421753003.1	WP_421752888.1	WP_421753676.1
Bacteroidota	*Croceitalea dokdonensis*	WP_054560488.1	WP_054557427.1	WP_054559144.1
Bacteroidota	*Croceitalea marina*	WP_377767746.1	WP_377766699.1	WP_377765282.1
Bacteroidota	*Croceitalea rosinachiae*	WP_311353170.1	WP_311350950.1	WP_311353314.1
Bacteroidota	*Croceitalea sp. MTPC5*	WP_339144497.1	WP_339140946.1	WP_339144417.1
Bacteroidota	*Croceivirga lutea*	WP_185783657.1	WP_185781643.1	WP_185783995.1
Bacteroidota	*Croceivirga radicis*	WP_010517378.1	WP_010517241.1	WP_080319389.1
Bacteroidota	*Croceivirga sp. JEA036*	WP_167987617.1	WP_167987710.1	WP_167989412.1
Bacteroidota	*Croceivirga thetidis*	WP_168552228.1	WP_168552311.1	WP_168553752.1
Bacteroidota	*Crocinitomix algicola*	WP_070138319.1	WP_066755789.1	WP_066757945.1
Bacteroidota	*Crocinitomix catalasitica*	WP_027419381.1	WP_027421155.1	WP_051568672.1
Bacteroidota	*Cruoricaptor ignavus*	WP_392437594.1	WP_073177968.1	WP_073179178.1
Bacteroidota	*Cryomorpha ignava*	WP_163283374.1	WP_163285353.1	WP_163285151.1
Bacteroidota	*Cyclobacterium amurskyense*	WP_048642148.1	WP_048642687.1	WP_048644384.1
Bacteroidota	*Cyclobacterium jeungdonense*	WP_163383924.1	WP_163385926.1	WP_163385429.1
Bacteroidota	*Cyclobacterium lianum*	WP_073091641.1	WP_073096623.1	WP_073090639.1
Bacteroidota	*Cyclobacterium marinum*	WP_149393370.1	WP_014021153.1	WP_014019414.1
Bacteroidota	*Cyclobacterium plantarum*	WP_166151612.1	WP_166149796.1	WP_166146008.1
Bacteroidota	*Cyclobacterium qasimii*	WP_020888752.1	WP_020893013.1	WP_020890698.1
Bacteroidota	*Cyclobacterium roseum*	WP_162419248.1	WP_162416044.1	WP_162418251.1
Bacteroidota	*Cyclobacterium salsum*	WP_162341569.1	WP_162343545.1	WP_162343434.1
Bacteroidota	*Cyclobacterium sp.*	WP_291369886.1	WP_410265425.1	WP_291372784.1
Bacteroidota	*Cyclobacterium xiamenense*	WP_375584005.1	WP_375583490.1	WP_092172779.1
Bacteroidota	*Cytophaga aurantiaca*	WP_018341432.1	WP_018344950.1	WP_018344260.1
Bacteroidota	*Cytophaga hutchinsonii*	WP_011583422.1	WP_011585160.1	WP_011584089.1
Bacteroidota	*Cytophaga sp. FL35*	WP_187456895.1	WP_187458128.1	WP_222937458.1
Bacteroidota	*Daejeonella lutea*	WP_079702447.1	WP_079700741.1	WP_079703645.1

Bacteroidota	*Daejeonella oryzae*	WP_026896934.1	WP_026898669.1	WP_026896820.1
Bacteroidota	*Daejeonella rubra*	WP_090706014.1	WP_090700380.1	WP_090706315.1
Bacteroidota	*Daejeonella sp.*	WP_411274008.1	WP_340380637.1	WP_324485892.1
Bacteroidota	*Danxiaibacter flavus*	WP_369329328.1	WP_369329440.1	WP_369330972.1
Bacteroidota	*Dawidia cretensis*	WP_254085524.1	WP_254087815.1	WP_254082658.1
Bacteroidota	*Dawidia soli*	WP_254090799.1	WP_254093836.1	WP_254092065.1
Bacteroidota	*Deminuibacter soli*	WP_116845410.1	WP_116847565.1	WP_116846911.1
Bacteroidota	*Desertivirga arenae*	WP_207535454.1	WP_207532598.1	WP_207534650.1
Bacteroidota	*Desertivirga brevis*	WP_207424218.1	WP_207422904.1	WP_207424873.1
Bacteroidota	*Desertivirga xinjiangensis*	WP_256013323.1	WP_256009515.1	WP_256009196.1
Bacteroidota	*Dinghuibacter silviterrae*	WP_133989948.1	WP_133993427.1	WP_133993337.1
Bacteroidota	*Dokdonia donghaensis*	WP_375251903.1	WP_035324608.1	WP_035327796.1
Bacteroidota	*Dokdonia pacifica*	WP_089371124.1	WP_089371369.1	WP_308489842.1
Bacteroidota	*Dokdonia ponticola*	WP_379978956.1	WP_379978462.1	WP_379977171.1
Bacteroidota	*Dokdonia sinensis*	WP_121916639.1	WP_121917143.1	WP_121915746.1
Bacteroidota	*Dokdonia sp.*	WP_348168451.1	WP_348171663.1	WP_348163442.1
Bacteroidota	*Dyadobacter alkalitolerans*	WP_026629948.1	WP_026629725.1	WP_026632001.1
Bacteroidota	*Dyadobacter arcticus*	WP_167269929.1	WP_167274150.1	WP_167266676.1
Bacteroidota	*Dyadobacter beijingensis*	WP_019940798.1	WP_019944208.1	WP_019944815.1
Bacteroidota	*Dyadobacter bucti*	WP_138479005.1	WP_138481385.1	WP_138482428.1
Bacteroidota	*Dyadobacter chenwenxiniae*	WP_234607536.1	WP_234655679.1	WP_234658388.1
Bacteroidota	*Dyadobacter crusticola*	WP_031526644.1	WP_031527697.1	WP_031528518.1
Bacteroidota	*Dyadobacter diqingensis*	WP_254411353.1	WP_254412135.1	WP_254561571.1
Bacteroidota	*Dyadobacter endophyticus*	WP_188935609.1	WP_188927460.1	WP_188938595.1
Bacteroidota	*Dyadobacter fanqingshengii*	WP_234612655.1	WP_235138158.1	WP_234615245.1
Bacteroidota	*Dyadobacter fermentans*	WP_223403261.1	WP_012779703.1	WP_050774706.1
Bacteroidota	*Dyadobacter flavalbus*	WP_139010466.1	WP_139010683.1	WP_139012196.1
Bacteroidota	*Dyadobacter frigoris*	WP_137343065.1	WP_137339996.1	WP_137341069.1

Bacteroidota	*Dyadobacter helix*	WP_215237805.1	WP_215238273.1	WP_406566865.1
Bacteroidota	*Dyadobacter jejuensis*	WP_109674238.1	WP_109676885.1	WP_310588680.1
Bacteroidota	*Dyadobacter koreensis*	WP_090334368.1	WP_090338592.1	WP_090340372.1
Bacteroidota	*Dyadobacter luteus*	WP_115833335.1	WP_115829263.1	WP_115832780.1
Bacteroidota	*Dyadobacter luticola*	WP_138367717.1	WP_138363610.1	WP_138368071.1
Bacteroidota	*Dyadobacter pollutisoli*	WP_244822797.1	WP_244819587.1	WP_244822558.1
Bacteroidota	*Dyadobacter psychrophilus*	WP_082212856.1	WP_082214096.1	WP_082216753.1
Bacteroidota	*Dyadobacter psychrotolerans*	WP_131955926.1	WP_131957012.1	WP_131960551.1
Bacteroidota	*Dyadobacter sandarakinus*	WP_204663031.1	WP_204660248.1	WP_204658485.1
Bacteroidota	*Dyadobacter sediminis*	WP_138279644.1	WP_138281443.1	WP_138283311.1
Bacteroidota	*Dyadobacter soli*	WP_090147482.1	WP_090146956.1	WP_090149517.1
Bacteroidota	*Dyadobacter sp.*	WP_348067880.1	WP_291206586.1	WP_291200977.1
Bacteroidota	*Dyadobacter subterraneus*	WP_194122670.1	WP_194120119.1	WP_194124010.1
Bacteroidota	*Dyadobacter tibetensis*	WP_025762109.1	WP_025761910.1	WP_229236027.1
Bacteroidota	*Echinicola arenosa*	WP_192009333.1	WP_192010197.1	WP_192010504.1
Bacteroidota	*Echinicola jeungdonensis*	WP_290247152.1	WP_290249494.1	WP_290247245.1
Bacteroidota	*Echinicola marina*	WP_226334858.1	WP_226333278.1	WP_226335464.1
Bacteroidota	*Echinicola pacifica*	WP_018472072.1	WP_018473938.1	WP_018475493.1
Bacteroidota	*Echinicola rosea*	WP_137401295.1	WP_137403783.1	WP_137401942.1
Bacteroidota	*Echinicola salinicaeni*	WP_186753604.1	WP_186757912.1	WP_186755834.1
Bacteroidota	*Echinicola shivajiensis*	WP_215225948.1	WP_215226756.1	WP_215225276.1
Bacteroidota	*Echinicola soli*	WP_141613416.1	WP_141615741.1	WP_141614086.1
Bacteroidota	*Echinicola sp. 20G*	WP_200974775.1	WP_200978684.1	WP_200975499.1
Bacteroidota	*Echinicola strongylocentroti*	WP_112783852.1	WP_112782133.1	WP_112784437.1
Bacteroidota	*Echinicola vietnamensis*	WP_015264618.1	WP_015267203.1	WP_015265338.1
Bacteroidota	*Edaphocola aurantiacus*	WP_222166449.1	WP_222167030.1	WP_222166341.1
Bacteroidota	*Edaphocola flava*	WP_129021451.1	WP_129019585.1	WP_161971320.1
Bacteroidota	*Eisenibacter elegans*	WP_027000537.1	WP_027000503.1	WP_027000333.1

Bacteroidota	*Ekhidna lutea*	WP_089356447.1	WP_089357853.1	WP_089355072.1
Bacteroidota	*Ekhidna sp.*	WP_350219655.1	WP_420575267.1	WP_424962706.1
Bacteroidota	*Elizabethkingia anophelis*	WP_407478991.1	WP_367604863.1	WP_024567208.1
Bacteroidota	*Elizabethkingia argenteiflava*	WP_166519254.1	WP_166519561.1	WP_166520285.1
Bacteroidota	*Elizabethkingia meningoseptica*	WP_326516950.1	WP_249063179.1	WP_078770067.1
Bacteroidota	*Elizabethkingia sp. YR214*	WP_107809619.1	WP_107807644.1	WP_107807205.1
Bacteroidota	*Elizabethkingia ursingii*	WP_249077995.1	WP_078403340.1	WP_249078125.1
Bacteroidota	*Empedobacter brevis*	WP_312557397.1	WP_019976302.1	WP_353097325.1
Bacteroidota	*Empedobacter falsenii*	WP_219033575.1	WP_038336617.1	WP_416403977.1
Bacteroidota	*Empedobacter sedimenti*	WP_282628566.1	WP_282630778.1	WP_282629868.1
Bacteroidota	*Empedobacter tilapiae*	WP_314241946.1	WP_135834524.1	WP_314244267.1
Bacteroidota	*Emticicia agri*	WP_130024155.1	WP_130019593.1	WP_130020679.1
Bacteroidota	*Emticicia aquatica*	WP_238804023.1	WP_238806179.1	WP_238807146.1
Bacteroidota	*Emticicia aquatilis*	WP_188768899.1	WP_188770815.1	WP_188765468.1
Bacteroidota	*Emticicia oligotrophica*	WP_305951787.1	WP_015027285.1	WP_015028296.1
Bacteroidota	*Emticicia soli*	WP_340239816.1	WP_340237658.1	WP_340233778.1
Bacteroidota	*Emticicia sp.*	WP_421769690.1	WP_421772207.1	WP_421771868.1
Bacteroidota	*Epilithonimonas arachidiradicis*	WP_120213936.1	WP_120213882.1	WP_120212215.1
Bacteroidota	*Epilithonimonas bovis*	WP_076781783.1	WP_076783416.1	WP_076783565.1
Bacteroidota	*Epilithonimonas caeni*	WP_027383614.1	WP_027383664.1	WP_034671664.1
Bacteroidota	*Epilithonimonas hispanica*	WP_116033190.1	WP_116033058.1	WP_116033454.1
Bacteroidota	*Epilithonimonas hominis*	WP_089768928.1	WP_312750933.1	WP_313306453.1
Bacteroidota	*Epilithonimonas hungarica*	WP_089874829.1	WP_307311947.1	WP_307310474.1
Bacteroidota	*Epilithonimonas lactis*	WP_034976505.1	WP_034976395.1	WP_311316404.1
Bacteroidota	*Epilithonimonas mollis*	WP_072997678.1	WP_072996781.1	WP_072997439.1
Bacteroidota	*Epilithonimonas pallida*	WP_283416258.1	WP_283416210.1	WP_283415997.1
Bacteroidota	*Epilithonimonas sp.*	WP_374440986.1	WP_312768802.1	WP_374441781.1
Bacteroidota	*Epilithonimonas tenax*	WP_028121144.1	WP_028121200.1	WP_316928783.1

Bacteroidota	*Epilithonimonas vandammei*	WP_124801570.1	WP_124801530.1	WP_312194444.1
Bacteroidota	*Epilithonimonas xixisoli*	WP_133945463.1	WP_133945376.1	WP_133944863.1
Bacteroidota	*Epilithonimonas zeae*	WP_074236880.1	WP_074236937.1	WP_248875071.1
Bacteroidota	*Eudoraea adriatica*	WP_019671452.1	WP_019671554.1	WP_026348505.1
Bacteroidota	*Eudoraea chungangensis*	WP_276390869.1	WP_276391857.1	WP_276391193.1
Bacteroidota	*Eudoraea sp.*	WP_424290759.1	WP_424281830.1	WP_424285184.1
Bacteroidota	*Euzebyella marina*	WP_121849468.1	WP_121849330.1	WP_121850639.1
Bacteroidota	*Euzebyella saccharophila*	WP_192462928.1	WP_192463011.1	WP_192462489.1
Bacteroidota	*Faecalibacter bovis*	WP_230477862.1	WP_230476304.1	WP_230477768.1
Bacteroidota	*Faecalibacter macacae*	WP_121935498.1	WP_121934504.1	WP_121933187.1
Bacteroidota	*Faecalibacter rhinopitheci*	WP_194183591.1	WP_194181660.1	WP_194183128.1
Bacteroidota	*Faecalibacter sp. LW9*	WP_323674094.1	WP_322969619.1	WP_322971732.1
Bacteroidota	*Ferruginibacter albus*	WP_224015377.1	WP_224019142.1	WP_224018449.1
Bacteroidota	*Ferruginibacter lapsinanis*	WP_229761199.1	WP_229759758.1	WP_229760830.1
Bacteroidota	*Ferruginibacter sp.*	WP_301920859.1	WP_301932169.1	WP_301928244.1
Bacteroidota	*Ferruginibacter yonginensis*	WP_379710127.1	WP_379706315.1	WP_379706877.1
Bacteroidota	*Fibrella aestuarina*	WP_015329526.1	WP_015331947.1	WP_015329586.1
Bacteroidota	*Fibrella aquatilis*	WP_207338346.1	WP_207337558.1	WP_207336838.1
Bacteroidota	*Fibrella forsythiae*	WP_207329357.1	WP_207327138.1	WP_207329926.1
Bacteroidota	*Fibrella rubiginis*	WP_207364873.1	WP_207366683.1	WP_207365617.1
Bacteroidota	*Fibrella sp. GW2-5*	WP_370726049.1	WP_370722175.1	WP_370724314.1
Bacteroidota	*Fibrisoma limi*	WP_009283656.1	WP_009283245.1	WP_009284943.1
Bacteroidota	*Fibrisoma montanum*	WP_119666868.1	WP_119667212.1	WP_119666485.1
Bacteroidota	*Fibrivirga algicola*	WP_085411010.1	WP_166693286.1	WP_166692858.1
Bacteroidota	*Filimonas effusa*	WP_129005128.1	WP_246022425.1	WP_129001150.1
Bacteroidota	*Filimonas lacunae*	WP_076379672.1	WP_076378918.1	WP_231940298.1
Bacteroidota	*Filimonas zeae*	WP_188957660.1	WP_188951939.1	WP_188952473.1
Bacteroidota	*Flagellimonas aequoris*	WP_119638374.1	WP_119641752.1	WP_119639284.1

Bacteroidota	*Flagellimonas algicola*	WP_138832362.1	WP_138832555.1	WP_138831947.1
Bacteroidota	*Flagellimonas allohymeniacidonis*	WP_130608790.1	WP_130609031.1	WP_130607915.1
Bacteroidota	*Flagellimonas alvinocaridis*	WP_136567100.1	WP_136567470.1	WP_136566847.1
Bacteroidota	*Flagellimonas amoyensis*	WP_108424753.1	WP_108424665.1	WP_108425404.1
Bacteroidota	*Flagellimonas aquimarina*	WP_109664998.1	WP_109663516.1	WP_109664479.1
Bacteroidota	*Flagellimonas aurea*	WP_225604307.1	WP_225604384.1	WP_207036507.1
Bacteroidota	*Flagellimonas baculiformis*	WP_318345083.1	WP_318344797.1	WP_318344658.1
Bacteroidota	*Flagellimonas beolgyonensis*	WP_127019374.1	WP_127019184.1	WP_127020011.1
Bacteroidota	*Flagellimonas crocea*	WP_318308088.1	WP_318308224.1	WP_318312414.1
Bacteroidota	*Flagellimonas eckloniae*	WP_055393033.1	WP_055392895.1	WP_055393334.1
Bacteroidota	*Flagellimonas flava*	WP_073182112.1	WP_073176911.1	WP_073181092.1
Bacteroidota	*Flagellimonas halotolerans*	WP_326278964.1	WP_293162775.1	WP_326279016.1
Bacteroidota	*Flagellimonas hymeniacidonis*	WP_147744767.1	WP_147744681.1	WP_147745025.1
Bacteroidota	*Flagellimonas iocasae*	WP_379829643.1	WP_379829557.1	WP_379830012.1
Bacteroidota	*Flagellimonas lutaonensis*	WP_045802637.1	WP_045802737.1	WP_045802577.1
Bacteroidota	*Flagellimonas lutimaris*	WP_119609232.1	WP_119609392.1	WP_414459438.1
Bacteroidota	*Flagellimonas marinaquae*	WP_338194641.1	WP_127140727.1	WP_338195901.1
Bacteroidota	*Flagellimonas maritima*	WP_112379135.1	WP_112379253.1	WP_112378974.1
Bacteroidota	*Flagellimonas meishanensis*	WP_222983916.1	WP_222983994.1	WP_222983733.1
Bacteroidota	*Flagellimonas meridianipacifica*	WP_106147075.1	WP_106146871.1	WP_106147422.1
Bacteroidota	*Flagellimonas myxillae*	WP_249969211.1	WP_249969116.1	WP_249969413.1
Bacteroidota	*Flagellimonas nanhaiensis*	WP_116185870.1	WP_116183645.1	WP_116185290.1
Bacteroidota	*Flagellimonas oceanensis*	WP_421824386.1	WP_127138859.1	WP_421824618.1
Bacteroidota	*Flagellimonas oceani*	WP_166247842.1	WP_166247919.1	WP_166247384.1
Bacteroidota	*Flagellimonas ochracea*	WP_166522626.1	WP_166522542.1	WP_166524569.1
Bacteroidota	*Flagellimonas okinawensis*	WP_275650265.1	WP_275650364.1	WP_275650583.1
Bacteroidota	*Flagellimonas olearia*	WP_129654481.1	WP_152130461.1	WP_152129927.1
Bacteroidota	*Flagellimonas onchidii*	WP_136465462.1	WP_136465354.1	WP_136468868.1

Bacteroidota	*Flagellimonas pacifica*	WP_097045101.1	WP_097045209.1	WP_097044884.1
Bacteroidota	*Flagellimonas pelagia*	WP_119646572.1	WP_119646449.1	WP_119648244.1
Bacteroidota	*Flagellimonas profundi*	WP_207026198.1	WP_207026359.1	WP_207025555.1
Bacteroidota	*Flagellimonas ruestringensis*	WP_014033624.1	WP_014034266.1	WP_224836465.1
Bacteroidota	*Flagellimonas sediminis*	WP_163633580.1	WP_163633770.1	WP_163636359.1
Bacteroidota	*Flagellimonas sp.*	WP_420600871.1	WP_418499172.1	WP_420322096.1
Bacteroidota	*Flagellimonas spongiicola*	WP_249658705.1	WP_249656751.1	WP_249658493.1
Bacteroidota	*Flagellimonas yonaguniensis*	WP_275616580.1	WP_275615732.1	WP_275616724.1
Bacteroidota	*Flagellimonas zhangzhouensis*	WP_090298686.1	WP_090297115.1	WP_090298155.1
Bacteroidota	*Flammeovirga kamogawensis*	WP_144073762.1	WP_144073022.1	WP_144074686.1
Bacteroidota	*Flammeovirga pacifica*	WP_044227781.1	WP_044219798.1	WP_044228724.1
Bacteroidota	*Flammeovirga pectinis*	WP_126616529.1	WP_126610898.1	WP_126614491.1
Bacteroidota	*Flammeovirga sp. MY04*	WP_066210782.1	WP_066212474.1	WP_066205624.1
Bacteroidota	*Flammeovirga yaeyamensis*	WP_169664813.1	WP_169662846.1	WP_169665136.1
Bacteroidota	*Flaviaesturariibacter amylovorans*	WP_345252897.1	WP_345255691.1	WP_345253761.1
Bacteroidota	*Flaviaesturariibacter aridisoli*	WP_131850298.1	WP_131850986.1	WP_131853005.1
Bacteroidota	*Flaviaesturariibacter flavus*	WP_131449966.1	WP_243647739.1	WP_131449650.1
Bacteroidota	*Flavicella marina*	WP_152286514.1	WP_152286678.1	WP_226913082.1
Bacteroidota	*Flavicella sediminum*	WP_139959103.1	WP_139959517.1	WP_139959645.1
Bacteroidota	*Flavicella sp.*	WP_336891546.1	WP_300326719.1	WP_336891758.1
Bacteroidota	*Flavihumibacter cheonanensis*	WP_239602470.1	WP_239595074.1	WP_239595392.1
Bacteroidota	*Flavihumibacter fluminis*	WP_234867994.1	WP_234865708.1	WP_234865854.1
Bacteroidota	*Flavihumibacter fluvii*	WP_214458631.1	WP_214457879.1	WP_214457758.1
Bacteroidota	*Flavihumibacter petaseus*	WP_046367457.1	WP_046370235.1	WP_046370374.1
Bacteroidota	*Flavihumibacter profundi*	WP_224117810.1	WP_224115123.1	WP_224115252.1
Bacteroidota	*Flavihumibacter rivuli*	WP_214447400.1	WP_214449400.1	WP_239804314.1
Bacteroidota	*Flavihumibacter solisilvae*	WP_039138289.1	WP_039140031.1	WP_039141623.1
Bacteroidota	*Flavihumibacter sp.*	WP_332732135.1	WP_332734476.1	WP_332734753.1

Bacteroidota	*Flavihumibacter stibioxidans*	WP_187255864.1	WP_187255311.1	WP_187255180.1
Bacteroidota	*Flavilitoribacter nigricans*	WP_099155523.1	WP_099155433.1	WP_099148935.1
Bacteroidota	*Flavimarina sp. Hel_I_48*	WP_031427791.1	WP_031425752.1	WP_031427820.1
Bacteroidota	*Flavipsychrobacter stenotrophus*	WP_105041010.1	WP_105040190.1	WP_105037087.1
Bacteroidota	*Flaviramulus aquimarinus*	WP_345272414.1	WP_345274344.1	WP_345272394.1
Bacteroidota	*Flaviramulus basaltis*	WP_072401320.1	WP_171946619.1	WP_072402259.1
Bacteroidota	*Flaviramulus multivorans*	WP_237230794.1	WP_237232560.1	WP_237230925.1
Bacteroidota	*Flaviramulus sp. BrNp1-15*	WP_238673743.1	WP_238676151.1	WP_238673896.1
Bacteroidota	*Flavisolibacter ginsengisoli*	WP_072835562.1	WP_072835367.1	WP_072835393.1
Bacteroidota	*Flavisolibacter ginsenosidimutans*	WP_146785371.1	WP_146781711.1	WP_146781592.1
Bacteroidota	*Flavisolibacter nicotianae*	WP_121354209.1	WP_121356021.1	WP_121356571.1
Bacteroidota	*Flavisolibacter tropicus*	WP_066404609.1	WP_066401941.1	WP_066402027.1
Bacteroidota	*Flavitalea sp. BT771*	WP_303588332.1	WP_303587254.1	WP_303591133.1
Bacteroidota	*Flavivirga abyssicola*	WP_303317827.1	WP_303315654.1	WP_303319200.1
Bacteroidota	*Flavivirga algicola*	WP_169673212.1	WP_169669341.1	WP_169676338.1
Bacteroidota	*Flavivirga amylovorans*	WP_303282627.1	WP_303283341.1	WP_303283293.1
Bacteroidota	*Flavivirga aquatica*	WP_069830633.1	WP_069831205.1	WP_069830554.1
Bacteroidota	*Flavivirga aquimarina*	WP_303279803.1	WP_303277252.1	WP_303276492.1
Bacteroidota	*Flavivirga eckloniae*	WP_102758028.1	WP_102754615.1	WP_102754426.1
Bacteroidota	*Flavivirga jejuensis*	WP_303303193.1	WP_303301116.1	WP_303302142.1
Bacteroidota	*Flavivirga rizhaonensis*	WP_135878189.1	WP_135874426.1	WP_135874598.1
Bacteroidota	*Flavivirga sp. 57AJ16*	WP_274184694.1	WP_274186157.1	WP_274185721.1
Bacteroidota	*Flavivirga spongiicola*	WP_303307549.1	WP_303308519.1	WP_303308337.1
Bacteroidota	*Flavobacterium acetivorans*	WP_230062547.1	WP_230061645.1	WP_230061457.1
Bacteroidota	*Flavobacterium aciduliphilum*	WP_112111771.1	WP_112113984.1	WP_112111999.1
Bacteroidota	*Flavobacterium adhaerens*	WP_348800068.1	WP_348799652.1	WP_348800379.1
Bacteroidota	*Flavobacterium aerium*	WP_306350197.1	WP_306352814.1	WP_306352934.1
Bacteroidota	*Flavobacterium aestivum*	WP_268846490.1	WP_268846139.1	WP_281322463.1

Bacteroidota	*Flavobacterium agri*	WP_176004808.1	WP_176004297.1	WP_176006078.1
Bacteroidota	*Flavobacterium agrisoli*	WP_200104139.1	WP_200104943.1	WP_200105035.1
Bacteroidota	*Flavobacterium ajazii*	WP_163410375.1	WP_163408156.1	WP_163408377.1
Bacteroidota	*Flavobacterium akiainvivens*	WP_054406461.1	WP_054409670.1	WP_054409766.1
Bacteroidota	*Flavobacterium album*	WP_108778230.1	WP_108778512.1	WP_108779563.1
Bacteroidota	*Flavobacterium algicola*	WP_239865649.1	WP_239867780.1	WP_239866461.1
Bacteroidota	*Flavobacterium alkalisoli*	WP_147583880.1	WP_147583557.1	WP_417351809.1
Bacteroidota	*Flavobacterium alvei*	WP_103805007.1	WP_304197511.1	WP_304201011.1
Bacteroidota	*Flavobacterium ammonificans*	WP_229324657.1	WP_229323846.1	WP_229325007.1
Bacteroidota	*Flavobacterium ammoniigenes*	WP_229316756.1	WP_229316411.1	WP_229316318.1
Bacteroidota	*Flavobacterium amnicola*	WP_129435686.1	WP_129436520.1	WP_129436066.1
Bacteroidota	*Flavobacterium amniphilum*	WP_250595108.1	WP_250597894.1	WP_250598363.1
Bacteroidota	*Flavobacterium anhuiense*	WP_091133020.1	WP_091133221.1	WP_129745120.1
Bacteroidota	*Flavobacterium antarcticum*	WP_022827233.1	WP_022828706.1	WP_022828531.1
Bacteroidota	*Flavobacterium aquariorum*	WP_111410466.1	WP_111409112.1	WP_111409004.1
Bacteroidota	*Flavobacterium aquaticum*	WP_111567236.1	WP_111565803.1	WP_111565483.1
Bacteroidota	*Flavobacterium aquatile*	WP_035127441.1	WP_035126550.1	WP_035126315.1
Bacteroidota	*Flavobacterium aquicola*	WP_115812379.1	WP_115809349.1	WP_115810936.1
Bacteroidota	*Flavobacterium aquidurense*	WP_055096489.1	WP_055096091.1	WP_055095569.1
Bacteroidota	*Flavobacterium aquiphilum*	WP_281228934.1	WP_281226080.1	WP_281228415.1
Bacteroidota	*Flavobacterium araucananum*	WP_089480192.1	WP_089481795.1	WP_089478665.1
Bacteroidota	*Flavobacterium arcticum*	WP_114678538.1	WP_114678788.1	WP_114676491.1
Bacteroidota	*Flavobacterium ardleyense*	WP_318640429.1	WP_379804239.1	WP_318639709.1
Bacteroidota	*Flavobacterium arsenatis*	WP_310025109.1	WP_310028268.1	WP_310023609.1
Bacteroidota	*Flavobacterium artemisiae*	WP_379816507.1	WP_379817049.1	WP_379816923.1
Bacteroidota	*Flavobacterium arundinis*	WP_341695324.1	WP_341696461.1	WP_341696278.1
Bacteroidota	*Flavobacterium aurantiibacter*	WP_094485081.1	WP_094486620.1	WP_094487510.1
Bacteroidota	*Flavobacterium aureirubrum*	WP_342695397.1	WP_342696707.1	WP_342696298.1

Bacteroidota	*Flavobacterium azizsancarii*	WP_271336029.1	WP_271338417.1	WP_271334397.1
Bacteroidota	*Flavobacterium azooxidireducens*	WP_248434158.1	WP_248433393.1	WP_248433145.1
Bacteroidota	*Flavobacterium beibuense*	WP_035133659.1	WP_129750265.1	WP_417366338.1
Bacteroidota	*Flavobacterium bernardetii*	WP_166125315.1	WP_166126073.1	WP_166126115.1
Bacteroidota	*Flavobacterium bizetiae*	WP_263759732.1	WP_173971207.1	WP_395368299.1
Bacteroidota	*Flavobacterium branchiarum*	WP_290262884.1	WP_290262510.1	WP_290266136.1
Bacteroidota	*Flavobacterium branchiicola*	WP_213258301.1	WP_213258164.1	WP_213257900.1
Bacteroidota	*Flavobacterium branchiophilum*	WP_014083711.1	WP_014083730.1	WP_089081437.1
Bacteroidota	*Flavobacterium buctense*	WP_341432331.1	WP_187660755.1	WP_187660569.1
Bacteroidota	*Flavobacterium caeni*	WP_091141017.1	WP_091140782.1	WP_091139974.1
Bacteroidota	*Flavobacterium calami*	WP_341690908.1	WP_341692856.1	WP_341692452.1
Bacteroidota	*Flavobacterium capsici*	WP_313321563.1	WP_313323035.1	WP_313325783.1
Bacteroidota	*Flavobacterium caseinilyticum*	WP_131908690.1	WP_131910707.1	WP_131909271.1
Bacteroidota	*Flavobacterium cauense*	WP_023571266.1	WP_023571697.1	WP_023570087.1
Bacteroidota	*Flavobacterium celericrescens*	WP_166235729.1	WP_166236799.1	WP_166235918.1
Bacteroidota	*Flavobacterium cellulosilyticum*	WP_132006492.1	WP_132006986.1	WP_132004434.1
Bacteroidota	*Flavobacterium cerinum*	WP_256550675.1	WP_128391093.1	WP_128388847.1
Bacteroidota	*Flavobacterium channae*	WP_231834529.1	WP_231836887.1	WP_231836528.1
Bacteroidota	*Flavobacterium cheonanense*	WP_344816363.1	WP_344816714.1	WP_344816211.1
Bacteroidota	*Flavobacterium cheongpyeongense*	WP_110307633.1	WP_110304959.1	WP_110304752.1
Bacteroidota	*Flavobacterium chilense*	WP_068842693.1	WP_068842835.1	WP_068844503.1
Bacteroidota	*Flavobacterium chuncheonense*	WP_379810082.1	WP_379811473.1	WP_379811128.1
Bacteroidota	*Flavobacterium chungnamense*	WP_345094576.1	WP_345095656.1	WP_345093744.1
Bacteroidota	*Flavobacterium circumlabens*	WP_348080008.1	WP_132034782.1	WP_348078518.1
Bacteroidota	*Flavobacterium collinsii*	WP_173968038.1	WP_263361526.1	WP_173966104.1
Bacteroidota	*Flavobacterium columnare*	WP_014165519.1	WP_107317836.1	WP_324712696.1
Bacteroidota	*Flavobacterium coralii*	WP_223052048.1	WP_348679228.1	WP_223053151.1
Bacteroidota	*Flavobacterium crassostreae*	WP_066335673.1	WP_066334792.1	WP_066334607.1

Bacteroidota	*Flavobacterium croceum*	WP_103725045.1	WP_103726256.1	WP_103726419.1
Bacteroidota	*Flavobacterium crocinum*	WP_109194175.1	WP_109194355.1	WP_109194604.1
Bacteroidota	*Flavobacterium cucumis*	WP_073584520.1	WP_073582549.1	WP_073580360.1
Bacteroidota	*Flavobacterium cupreum*	WP_127338736.1	WP_127338606.1	WP_127339709.1
Bacteroidota	*Flavobacterium cupriresistens*	WP_230002453.1	WP_230002663.1	WP_230002945.1
Bacteroidota	*Flavobacterium cutihirudinis*	WP_115888499.1	WP_115888668.1	WP_115888903.1
Bacteroidota	*Flavobacterium cyanobacteriorum*	WP_094416373.1	WP_094412463.1	WP_094417039.1
Bacteroidota	*Flavobacterium cyclinae*	WP_231843804.1	WP_231841638.1	WP_231841964.1
Bacteroidota	*Flavobacterium daejeonense*	WP_026714153.1	WP_026715500.1	WP_026714113.1
Bacteroidota	*Flavobacterium daemonense*	WP_144339228.1	WP_144337841.1	WP_144338222.1
Bacteroidota	*Flavobacterium dankookense*	WP_133531808.1	WP_133531489.1	WP_133533978.1
Bacteroidota	*Flavobacterium dauae*	WP_129758015.1	WP_129757852.1	WP_129757042.1
Bacteroidota	*Flavobacterium defluvii*	WP_073416481.1	WP_073416641.1	WP_073417082.1
Bacteroidota	*Flavobacterium degerlachei*	WP_091434817.1	WP_091429243.1	WP_091434165.1
Bacteroidota	*Flavobacterium denitrificans*	WP_026730144.1	WP_026730335.1	WP_026728034.1
Bacteroidota	*Flavobacterium difficile*	WP_166077145.1	WP_166078006.1	WP_166077778.1
Bacteroidota	*Flavobacterium eburneipallidum*	WP_281336077.1	WP_269225176.1	WP_281337225.1
Bacteroidota	*Flavobacterium enshiense*	WP_367230005.1	WP_367230350.1	WP_367230791.1
Bacteroidota	*Flavobacterium erciyesense*	WP_210788498.1	WP_210788461.1	WP_210788629.1
Bacteroidota	*Flavobacterium facile*	WP_329804786.1	WP_329806052.1	WP_329806076.1
Bacteroidota	*Flavobacterium faecale*	WP_108741200.1	WP_108740786.1	WP_108740713.1
Bacteroidota	*Flavobacterium filum*	WP_276979020.1	WP_026709932.1	WP_026711516.1
Bacteroidota	*Flavobacterium flavigenum*	WP_269236635.1	WP_269236927.1	WP_269237110.1
Bacteroidota	*Flavobacterium flavipallidum*	WP_341700249.1	WP_341700978.1	WP_341701690.1
Bacteroidota	*Flavobacterium flevense*	WP_073245585.1	WP_073245546.1	WP_073244749.1
Bacteroidota	*Flavobacterium fluviale*	WP_113679386.1	WP_113680010.1	WP_113679743.1
Bacteroidota	*Flavobacterium fluviatile*	WP_163399805.1	WP_163401290.1	WP_163400519.1
Bacteroidota	*Flavobacterium fluvii*	WP_073370950.1	WP_073371333.1	WP_073370273.1

Bacteroidota	*Flavobacterium foetidum*	WP_135223692.1	WP_135220578.1	WP_135220849.1
Bacteroidota	*Flavobacterium fontis*	WP_073362329.1	WP_073365076.1	WP_073362776.1
Bacteroidota	*Flavobacterium fragile*	WP_250581805.1	WP_250582693.1	WP_250582588.1
Bacteroidota	*Flavobacterium franklandianum*	WP_143391881.1	WP_143391434.1	WP_143391604.1
Bacteroidota	*Flavobacterium frigidarium*	WP_371570782.1	WP_339654805.1	WP_026706692.1
Bacteroidota	*Flavobacterium frigidimaris*	WP_074658449.1	WP_074658704.1	WP_074659117.1
Bacteroidota	*Flavobacterium frigoris*	WP_074722389.1	WP_074723038.1	WP_007138112.1
Bacteroidota	*Flavobacterium frigoritolerans*	WP_264287449.1	WP_264287159.1	WP_264285323.1
Bacteroidota	*Flavobacterium fructosi*	WP_379857536.1	WP_379858922.1	WP_379857307.1
Bacteroidota	*Flavobacterium fryxellicola*	WP_066078745.1	WP_066078551.1	WP_066078063.1
Bacteroidota	*Flavobacterium galactosidilyticum*	WP_229942020.1	WP_229941185.1	WP_229943974.1
Bacteroidota	*Flavobacterium gelatinilyticum*	WP_281232719.1	WP_281232539.1	WP_281232268.1
Bacteroidota	*Flavobacterium gelidilacus*	WP_026715688.1	WP_026716442.1	WP_026716198.1
Bacteroidota	*Flavobacterium gillisiae*	WP_091086427.1	WP_091093031.1	WP_091090389.1
Bacteroidota	*Flavobacterium gilvum*	WP_035637836.1	WP_035638905.1	WP_035640009.1
Bacteroidota	*Flavobacterium ginsengiterrae*	WP_345146172.1	WP_345145973.1	WP_345145719.1
Bacteroidota	*Flavobacterium ginsenosidimutans*	WP_111285252.1	WP_111285576.1	WP_111289076.1
Bacteroidota	*Flavobacterium glaciei*	WP_114755205.1	WP_114755304.1	WP_114753358.1
Bacteroidota	*Flavobacterium glycines*	WP_066329059.1	WP_066329643.1	WP_066324645.1
Bacteroidota	*Flavobacterium granuli*	WP_072937916.1	WP_310002898.1	WP_072941334.1
Bacteroidota	*Flavobacterium gyeonganense*	WP_278010294.1	WP_278010560.1	WP_278010745.1
Bacteroidota	*Flavobacterium haoranii*	WP_072783792.1	WP_072785678.1	WP_072785004.1
Bacteroidota	*Flavobacterium hercynium*	WP_089051503.1	WP_089050641.1	WP_089051567.1
Bacteroidota	*Flavobacterium hibernum*	WP_041518361.1	WP_041515948.1	WP_041517094.1
Bacteroidota	*Flavobacterium hibisci*	WP_223680455.1	WP_223683385.1	WP_223681683.1
Bacteroidota	*Flavobacterium hiemivividum*	WP_132111183.1	WP_132111004.1	WP_132110648.1
Bacteroidota	*Flavobacterium humi*	WP_135524654.1	WP_135526473.1	WP_135526235.1
Bacteroidota	*Flavobacterium humidisoli*	WP_248727515.1	WP_248727640.1	WP_248727894.1

Bacteroidota	*Flavobacterium hungaricum*	WP_194139755.1	WP_194139922.1	WP_194140192.1
Bacteroidota	*Flavobacterium hydatis*	WP_035620475.1	WP_035627907.1	WP_035625949.1
Bacteroidota	*Flavobacterium hydrocarbonoxydans*	WP_160373147.1	WP_160376079.1	WP_160375866.1
Bacteroidota	*Flavobacterium hydrophilum*	WP_110345293.1	WP_110344961.1	WP_110344757.1
Bacteroidota	*Flavobacterium ichthyis*	WP_166536024.1	WP_166535779.1	WP_166537713.1
Bacteroidota	*Flavobacterium jejuense*	WP_140963139.1	WP_140959233.1	WP_140960741.1
Bacteroidota	*Flavobacterium johnsoniae*	WP_073408372.1	WP_289877969.1	WP_289878288.1
Bacteroidota	*Flavobacterium kayseriense*	WP_187011015.1	WP_187011005.1	WP_187010795.1
Bacteroidota	*Flavobacterium kingsejongi*	WP_108737943.1	WP_108737494.1	WP_108738362.1
Bacteroidota	*Flavobacterium lacisediminis*	WP_264369442.1	WP_264369666.1	WP_264368558.1
Bacteroidota	*Flavobacterium lacus*	WP_112085796.1	WP_112085450.1	WP_112086584.1
Bacteroidota	*Flavobacterium lacustre*	WP_269684458.1	WP_269685141.1	WP_269684167.1
Bacteroidota	*Flavobacterium laiguense*	WP_116763267.1	WP_116763918.1	WP_116760445.1
Bacteroidota	*Flavobacterium limi*	WP_163396595.1	WP_163394177.1	WP_163394387.1
Bacteroidota	*Flavobacterium limicola*	WP_121365842.1	WP_121365603.1	WP_121365642.1
Bacteroidota	*Flavobacterium limnophilum*	WP_281299364.1	WP_281299001.1	WP_281297214.1
Bacteroidota	*Flavobacterium limnosediminis*	WP_023579171.1	WP_023579392.1	WP_023580327.1
Bacteroidota	*Flavobacterium litorale*	WP_220639998.1	WP_220640443.1	WP_220640681.1
Bacteroidota	*Flavobacterium longum*	WP_415580331.1	WP_415580030.1	WP_415581162.1
Bacteroidota	*Flavobacterium lotistagni*	WP_166335680.1	WP_166336123.1	WP_166334538.1
Bacteroidota	*Flavobacterium luminosum*	WP_250592150.1	WP_250591819.1	WP_250591576.1
Bacteroidota	*Flavobacterium luteum*	WP_151108338.1	WP_151106222.1	WP_151107915.1
Bacteroidota	*Flavobacterium macacae*	WP_125011741.1	WP_125013480.1	WP_125012357.1
Bacteroidota	*Flavobacterium magnesitis*	WP_373391948.1	WP_373393194.1	WP_373390870.1
Bacteroidota	*Flavobacterium magnum*	WP_108371837.1	WP_108373551.1	WP_108370611.1
Bacteroidota	*Flavobacterium maritimum*	WP_348812361.1	WP_348811647.1	WP_348811765.1
Bacteroidota	*Flavobacterium microcysteis*	WP_140000093.1	WP_140001393.1	WP_140002987.1
Bacteroidota	*Flavobacterium micromati*	WP_073021752.1	WP_073021956.1	WP_073021419.1

Bacteroidota	*Flavobacterium muglaense*	WP_187020500.1	WP_187019474.1	WP_187016867.1
Bacteroidota	*Flavobacterium myungsuense*	WP_379759358.1	WP_379758019.1	WP_379755506.1
Bacteroidota	*Flavobacterium nackdongense*	WP_133277831.1	WP_133275103.1	WP_133275856.1
Bacteroidota	*Flavobacterium nakdongensis*	WP_309533082.1	WP_309532800.1	WP_309531613.1
Bacteroidota	*Flavobacterium nitratireducens*	WP_278035832.1	WP_278035364.1	WP_278035249.1
Bacteroidota	*Flavobacterium noncentrifugens*	WP_091396656.1	WP_091398032.1	WP_091393211.1
Bacteroidota	*Flavobacterium odoriferum*	WP_262318657.1	WP_262318477.1	WP_262317886.1
Bacteroidota	*Flavobacterium okayamense*	WP_221258181.1	WP_221258373.1	WP_221257683.1
Bacteroidota	*Flavobacterium olei*	WP_347049574.1	WP_347049694.1	WP_347049942.1
Bacteroidota	*Flavobacterium omnivorum*	WP_091258353.1	WP_091257743.1	WP_091259028.1
Bacteroidota	*Flavobacterium oreochromis*	WP_088397939.1	WP_088399325.1	WP_088397908.1
Bacteroidota	*Flavobacterium orientale*	WP_188361792.1	WP_188361733.1	WP_188361861.1
Bacteroidota	*Flavobacterium ovatum*	WP_366183810.1	WP_366183988.1	WP_366184218.1
Bacteroidota	*Flavobacterium pallidum*	WP_108904208.1	WP_108904408.1	WP_108903708.1
Bacteroidota	*Flavobacterium palustre*	WP_188493166.1	WP_188493705.1	WP_188493752.1
Bacteroidota	*Flavobacterium paronense*	WP_290285180.1	WP_290285908.1	WP_290284915.1
Bacteroidota	*Flavobacterium pectinovorum*	WP_140511595.1	WP_243232481.1	WP_073395410.1
Bacteroidota	*Flavobacterium petrolei*	WP_113666649.1	WP_395380808.1	WP_395380779.1
Bacteroidota	*Flavobacterium phragmitis*	WP_091492985.1	WP_091491738.1	WP_091492158.1
Bacteroidota	*Flavobacterium phycosphaerae*	WP_162127750.1	WP_162126086.1	WP_162128342.1
Bacteroidota	*Flavobacterium piscinae*	WP_129464813.1	WP_129464344.1	WP_129464983.1
Bacteroidota	*Flavobacterium pisciphilum*	WP_229987522.1	WP_229987734.1	WP_229990525.1
Bacteroidota	*Flavobacterium piscis*	WP_310284124.1	WP_065450509.1	WP_310281901.1
Bacteroidota	*Flavobacterium piscisymbiosum*	WP_230039643.1	WP_230039970.1	WP_230040703.1
Bacteroidota	*Flavobacterium plantiphilum*	WP_408078841.1	WP_408078801.1	WP_408081465.1
Bacteroidota	*Flavobacterium pokkalii*	WP_188220226.1	WP_055092636.1	WP_188221459.1
Bacteroidota	*Flavobacterium polysaccharolyticum*	WP_342692940.1	WP_342690826.1	WP_342691523.1
Bacteroidota	*Flavobacterium ponti*	WP_379740641.1	WP_379741904.1	WP_379743091.1

Bacteroidota	*Flavobacterium potami*	WP_223704457.1	WP_223704287.1	WP_223704074.1
Bacteroidota	*Flavobacterium poyangense*	WP_166923811.1	WP_166925746.1	WP_166922132.1
Bacteroidota	*Flavobacterium praedii*	WP_281239829.1	WP_281240117.1	WP_281239177.1
Bacteroidota	*Flavobacterium procerum*	WP_379682565.1	WP_379682729.1	WP_379683027.1
Bacteroidota	*Flavobacterium profundi*	WP_140996821.1	WP_140999128.1	WP_140998869.1
Bacteroidota	*Flavobacterium proteolyticum*	WP_194095444.1	WP_194095749.1	WP_194094760.1
Bacteroidota	*Flavobacterium psychraquaticum*	WP_322549922.1	WP_322549451.1	WP_322550351.1
Bacteroidota	*Flavobacterium psychrophilum*	WP_011962921.1	WP_011962816.1	WP_203095712.1
Bacteroidota	*Flavobacterium psychroterrae*	WP_213298471.1	WP_213298967.1	WP_213305312.1
Bacteroidota	*Flavobacterium psychrotolerans*	WP_116725783.1	WP_116723503.1	WP_116723560.1
Bacteroidota	*Flavobacterium psychrotrophum*	WP_116788075.1	WP_116788784.1	WP_116789352.1
Bacteroidota	*Flavobacterium pygoscelis*	WP_248427562.1	WP_248429168.1	WP_248427783.1
Bacteroidota	*Flavobacterium qiangtangense*	WP_379790684.1	WP_379791238.1	WP_379792899.1
Bacteroidota	*Flavobacterium rakeshii*	WP_157483142.1	WP_157481072.1	WP_157483317.1
Bacteroidota	*Flavobacterium reichenbachii*	WP_035683155.1	WP_035682855.1	WP_035690269.1
Bacteroidota	*Flavobacterium restrictum*	WP_144255860.1	WP_144254725.1	WP_144257666.1
Bacteroidota	*Flavobacterium rhizophilum*	WP_408075709.1	WP_408073956.1	WP_408074961.1
Bacteroidota	*Flavobacterium rhizosphaerae*	WP_408083731.1	WP_408083897.1	WP_408083921.1
Bacteroidota	*Flavobacterium rivuli*	WP_020214032.1	WP_020212488.1	WP_020212097.1
Bacteroidota	*Flavobacterium rivulicola*	WP_171222509.1	WP_171221989.1	WP_171223169.1
Bacteroidota	*Flavobacterium saccharophilum*	WP_072972051.1	WP_072972649.1	WP_072973028.1
Bacteroidota	*Flavobacterium saliperosum*	WP_023576809.1	WP_023576527.1	WP_023577062.1
Bacteroidota	*Flavobacterium sandaracinum*	WP_132066739.1	WP_132064968.1	WP_132064488.1
Bacteroidota	*Flavobacterium sangjuense*	WP_136152510.1	WP_136152911.1	WP_136152372.1
Bacteroidota	*Flavobacterium sasangense*	WP_026725803.1	WP_026726094.1	WP_026724613.1
Bacteroidota	*Flavobacterium sedimenticola*	WP_283238790.1	WP_283238321.1	WP_283238626.1
Bacteroidota	*Flavobacterium sediminis*	WP_109570272.1	WP_109567860.1	WP_109569801.1
Bacteroidota	*Flavobacterium segetis*	WP_072992850.1	WP_072993824.1	WP_072987071.1

Bacteroidota	*Flavobacterium selenitireducens*	WP_191018474.1	WP_191018872.1	WP_191020079.1
Bacteroidota	*Flavobacterium seoulense*	WP_035660257.1	WP_035661625.1	WP_035662614.1
Bacteroidota	*Flavobacterium sharifuzzamanii*	WP_111426281.1	WP_111426401.1	WP_111426645.1
Bacteroidota	*Flavobacterium shii*	WP_264206984.1	WP_264204354.1	WP_264206383.1
Bacteroidota	*Flavobacterium silvaticum*	WP_169528127.1	WP_169526441.1	WP_169528369.1
Bacteroidota	*Flavobacterium silvisoli*	WP_131476214.1	WP_131476810.1	WP_131475440.1
Bacteroidota	*Flavobacterium sinopsychrotolerans*	WP_091168489.1	WP_091166664.1	WP_091166756.1
Bacteroidota	*Flavobacterium soli*	WP_026705573.1	WP_026705749.1	WP_026704342.1
Bacteroidota	*Flavobacterium solisilvae*	WP_169523750.1	WP_169524792.1	WP_169524984.1
Bacteroidota	*Flavobacterium soyae*	WP_232682279.1	WP_232679388.1	WP_406844274.1
Bacteroidota	*Flavobacterium soyangense*	WP_194311707.1	WP_194312282.1	WP_194310813.1
Bacteroidota	*Flavobacterium sp.*	WP_371266015.1	WP_396184720.1	WP_324217648.1
Bacteroidota	*Flavobacterium stagni*	WP_129461143.1	WP_129462148.1	WP_129460802.1
Bacteroidota	*Flavobacterium suaedae*	WP_188621315.1	WP_188620323.1	WP_188620523.1
Bacteroidota	*Flavobacterium subsaxonicum*	WP_026991950.1	WP_026990105.1	WP_026993033.1
Bacteroidota	*Flavobacterium succinicans*	WP_064714826.1	WP_024980033.1	WP_024980258.1
Bacteroidota	*Flavobacterium sufflavum*	WP_128196693.1	WP_128194842.1	WP_128194048.1
Bacteroidota	*Flavobacterium suncheonense*	WP_026981611.1	WP_026979946.1	WP_026981151.1
Bacteroidota	*Flavobacterium supellecticarium*	WP_136404178.1	WP_136401869.1	WP_136403284.1
Bacteroidota	*Flavobacterium swingsii*	WP_091474524.1	WP_091474723.1	WP_091472898.1
Bacteroidota	*Flavobacterium tagetis*	WP_202003963.1	WP_202003572.1	WP_202006294.1
Bacteroidota	*Flavobacterium taihuense*	WP_219318647.1	WP_219318958.1	WP_219319062.1
Bacteroidota	*Flavobacterium tegetincola*	WP_026978617.1	WP_026977492.1	WP_026978195.1
Bacteroidota	*Flavobacterium terrae*	WP_073310928.1	WP_073311178.1	WP_073309335.1
Bacteroidota	*Flavobacterium terrigena*	WP_091313686.1	WP_091315883.1	WP_091311805.1
Bacteroidota	*Flavobacterium terrisoli*	WP_284652565.1	WP_284653435.1	WP_284652285.1
Bacteroidota	*Flavobacterium tibetense*	WP_113988761.1	WP_113989048.1	WP_113988628.1
Bacteroidota	*Flavobacterium tistrianum*	WP_111365996.1	WP_111366193.1	WP_111364009.1

Bacteroidota	*Flavobacterium tructae*	WP_374173388.1	WP_374173498.1	WP_070907458.1
Bacteroidota	*Flavobacterium turcicum*	WP_166135359.1	WP_166135473.1	WP_166134952.1
Bacteroidota	*Flavobacterium undicola*	WP_161640825.1	WP_161643606.1	WP_161643714.1
Bacteroidota	*Flavobacterium urocaniciphilum*	WP_091465144.1	WP_091468925.1	WP_091468962.1
Bacteroidota	*Flavobacterium urumqiense*	WP_104000151.1	WP_104000107.1	WP_103999846.1
Bacteroidota	*Flavobacterium ustbae*	WP_125722837.1	WP_125720253.1	WP_125723234.1
Bacteroidota	*Flavobacterium xanthum*	WP_073353088.1	WP_073353397.1	WP_073355174.1
Bacteroidota	*Flavobacterium xinjiangense*	WP_073211262.1	WP_073208242.1	WP_073207460.1
Bacteroidota	*Flavobacterium xueshanense*	WP_091206099.1	WP_091207614.1	WP_091203754.1
Bacteroidota	*Flavobacterium xylosi*	WP_379854913.1	WP_379853196.1	WP_379854551.1
Bacteroidota	*Flavobacterium yafengii*	WP_282728797.1	WP_282715470.1	WP_282730033.1
Bacteroidota	*Flavobacterium zepuense*	WP_143372536.1	WP_143375269.1	WP_143373232.1
Bacteroidota	*Flavobacterium zhairuonense*	WP_132988062.1	WP_132989944.1	WP_132989696.1
Bacteroidota	*Flavobacterium zhouii*	WP_379852713.1	WP_379851381.1	WP_379849947.1
Bacteroidota	*Flavobacterium zubiriense*	WP_373406039.1	WP_373406299.1	WP_373406703.1
Bacteroidota	*Flectobacillus longus*	WP_283371972.1	WP_283325847.1	WP_283328046.1
Bacteroidota	*Flectobacillus major*	WP_026997808.1	WP_026994392.1	WP_026997855.1
Bacteroidota	*Flectobacillus roseus*	WP_283373285.1	WP_283373442.1	WP_283346348.1
Bacteroidota	*Flectobacillus sp.*	WP_421820662.1	WP_421819229.1	WP_421818880.1
Bacteroidota	*Flexibacter flexilis*	WP_091508130.1	WP_091505765.1	WP_091516603.1
Bacteroidota	*Flexibacter sp. ATCC 35103*	WP_083692816.1	WP_083692959.1	WP_083693241.1
Bacteroidota	*Flexithrix dorotheae*	WP_020532198.1	WP_020528523.1	WP_020533907.1
Bacteroidota	*Fluviicola chungangensis*	WP_144331325.1	WP_186280074.1	WP_186280185.1
Bacteroidota	*Fluviicola sp.*	WP_309280893.1	WP_300359153.1	WP_300659961.1
Bacteroidota	*Fluviicola taffensis*	WP_013688266.1	WP_341903383.1	WP_013687422.1
Bacteroidota	*Foetidibacter luteolus*	WP_153796776.1	WP_153800762.1	WP_153795974.1
Bacteroidota	*Fontibacter flavus*	WP_382385908.1	WP_382387759.1	WP_382387480.1
Bacteroidota	*Formosa agariphila*	WP_038529567.1	WP_038528827.1	WP_038527824.1

Bacteroidota	*Formosa algae*	WP_057779342.1	WP_057778720.1	WP_057778503.1
Bacteroidota	*Formosa maritima*	WP_148452936.1	WP_148454901.1	WP_148455345.1
Bacteroidota	*Formosa sediminum*	WP_143381701.1	WP_143382029.1	WP_143380981.1
Bacteroidota	*Formosa sp. PL04*	WP_318498907.1	WP_318498557.1	WP_318501113.1
Bacteroidota	*Formosa undariae*	WP_382381391.1	WP_382381605.1	WP_382382124.1
Bacteroidota	*Frigoriflavimonas asaccharolytica*	WP_173780209.1	WP_173780018.1	WP_173779288.1
Bacteroidota	*Fulvitalea axinellae*	WP_338392299.1	WP_338391720.1	WP_338391676.1
Bacteroidota	*Fulvivirga aurantia*	WP_185152832.1	WP_155210970.1	WP_155212377.1
Bacteroidota	*Fulvivirga imtechensis*	WP_009583631.1	WP_009577485.1	WP_009579199.1
Bacteroidota	*Fulvivirga kasyanovii*	WP_155174132.1	WP_155171568.1	WP_343855452.1
Bacteroidota	*Fulvivirga ligni*	WP_233771349.1	WP_233770555.1	WP_233771846.1
Bacteroidota	*Fulvivirga lutea*	WP_205721864.1	WP_205721098.1	WP_205722411.1
Bacteroidota	*Fulvivirga lutimaris*	WP_155187492.1	WP_155185506.1	WP_155188308.1
Bacteroidota	*Fulvivirga marina*	WP_202854470.1	WP_202855140.1	WP_202854505.1
Bacteroidota	*Fulvivirga maritima*	WP_233778822.1	WP_233780993.1	WP_233780020.1
Bacteroidota	*Fulvivirga sedimenti*	WP_225698760.1	WP_225696347.1	WP_225699844.1
Bacteroidota	*Fulvivirga sediminis*	WP_202244114.1	WP_202243775.1	WP_202244855.1
Bacteroidota	*Fulvivirga sp.*	WP_350067676.1	WP_350116307.1	WP_350098834.1
Bacteroidota	*Fulvivirga ulvae*	WP_233767761.1	WP_233762065.1	WP_233765613.1
Bacteroidota	*Gaetbulibacter aestuarii*	WP_344741495.1	WP_344741373.1	WP_344742218.1
Bacteroidota	*Gaetbulibacter aquiaggeris*	WP_395438398.1	WP_395438696.1	WP_395437480.1
Bacteroidota	*Gaetbulibacter jejuensis*	WP_335976051.1	WP_343799672.1	WP_335974384.1
Bacteroidota	*Gaetbulibacter saemankumensis*	WP_027136548.1	WP_027136618.1	WP_027136785.1
Bacteroidota	*Gaetbulibacter sp. M235*	WP_394881643.1	WP_394882940.1	WP_394882766.1
Bacteroidota	*Galbibacter marinus*	WP_008991857.1	WP_008991011.1	WP_008991389.1
Bacteroidota	*Galbibacter mesophilus*	WP_202029560.1	WP_202027634.1	WP_202029510.1
Bacteroidota	*Galbibacter orientalis*	WP_335588164.1	WP_008614805.1	WP_337900065.1
Bacteroidota	*Galbibacter pacificus*	WP_277898839.1	WP_277899085.1	WP_277898211.1

Bacteroidota	*Galbibacter sp.*	WP_417360466.1	WP_417364129.1	WP_324301206.1
Bacteroidota	*Gangjinia marincola*	WP_343762526.1	WP_343764592.1	WP_343764901.1
Bacteroidota	*Gelatiniphilus marinus*	WP_388014987.1	WP_388013439.1	WP_388016878.1
Bacteroidota	*Gelidibacter algens*	WP_066431358.1	WP_066437546.1	WP_111625946.1
Bacteroidota	*Gelidibacter gilvus*	WP_129016144.1	WP_129017242.1	WP_129017912.1
Bacteroidota	*Gelidibacter japonicus*	WP_163515830.1	WP_250066750.1	WP_323027882.1
Bacteroidota	*Gelidibacter maritimus*	WP_182206455.1	WP_182204067.1	WP_182205246.1
Bacteroidota	*Gelidibacter mesophilus*	WP_027126355.1	WP_027127133.1	WP_027125360.1
Bacteroidota	*Gelidibacter pelagius*	WP_208233111.1	WP_208233378.1	WP_208232401.1
Bacteroidota	*Gelidibacter salicanalis*	WP_146893995.1	WP_199597625.1	WP_146888335.1
Bacteroidota	*Gelidibacter sediminis*	WP_133758180.1	WP_133757911.1	WP_133757493.1
Bacteroidota	*Gelidibacter sp.*	WP_348037356.1	WP_348037583.1	WP_325452820.1
Bacteroidota	*Geojedonia litorea*	WP_387961949.1	WP_387961441.1	WP_387960537.1
Bacteroidota	*Gillisia hiemivivida*	WP_146931822.1	WP_146931177.1	WP_146933916.1
Bacteroidota	*Gillisia limnaea*	WP_006987563.1	WP_006990115.1	WP_006987928.1
Bacteroidota	*Gillisia lutea*	WP_236133762.1	WP_236133415.1	WP_236133176.1
Bacteroidota	*Gillisia mitskevichiae*	WP_121345154.1	WP_121345645.1	WP_121345880.1
Bacteroidota	*Gillisia sp. Hel_I_86*	WP_144963149.1	WP_144962968.1	WP_144958730.1
Bacteroidota	*Gilvibacter sediminis*	WP_272832536.1	WP_272832132.1	WP_272833033.1
Bacteroidota	*Gilvibacter sp.*	WP_291185014.1	WP_420379623.1	WP_374958602.1
Bacteroidota	*Gilvirhabdus luticola*	WP_316663118.1	WP_316662346.1	WP_316661412.1
Bacteroidota	*Ginsengibacter hankyongi*	WP_150413724.1	WP_150415216.1	WP_150413131.1
Bacteroidota	*Gramella jeungdoensis*	WP_252113824.1	WP_252110723.1	WP_252110617.1
Bacteroidota	*Gramella sp. MAR_2010_147*	WP_089664712.1	WP_089665144.1	WP_089664164.1
Bacteroidota	*Gynurincola endophyticus*	WP_126973031.1	WP_126971669.1	WP_126971071.1
Bacteroidota	*Haliscomenobacter hydrossis*	WP_013765591.1	WP_013766642.1	WP_044234184.1
Bacteroidota	*Haliscomenobacter sp.*	WP_421797522.1	WP_353485078.1	WP_353483610.1
Bacteroidota	*Haloflavibacter putidus*	WP_141420749.1	WP_141421610.1	WP_141422424.1

Bacteroidota	*Halomarinibacterium sedimenti*	WP_219052331.1	WP_219052695.1	WP_219052270.1
Bacteroidota	*Halpernia frigidisoli*	WP_090079850.1	WP_090080935.1	WP_177205450.1
Bacteroidota	*Halpernia humi*	WP_103913058.1	WP_103913318.1	WP_103913510.1
Bacteroidota	*Halpernia sp.*	WP_417428735.1	WP_417428411.1	WP_417427724.1
Bacteroidota	*Hanamia caeni*	WP_123121589.1	WP_123121851.1	WP_123122442.1
Bacteroidota	*Hanstruepera flava*	WP_250436433.1	WP_250432405.1	WP_250434084.1
Bacteroidota	*Hanstruepera marina*	WP_223034528.1	WP_223033481.1	WP_223033900.1
Bacteroidota	*Hanstruepera neustonica*	WP_103052034.1	WP_103052338.1	WP_103051335.1
Bacteroidota	*Hanstruepera ponticola*	WP_104733670.1	WP_104733978.1	WP_191859243.1
Bacteroidota	*Haoranjiania flava*	WP_263039019.1	WP_263036497.1	WP_263036413.1
Bacteroidota	*Hufsiella arboris*	WP_160844963.1	WP_160844516.1	WP_160845831.1
Bacteroidota	*Hufsiella ginkgonis*	WP_160907940.1	WP_160906799.1	WP_160908011.1
Bacteroidota	*Hugenholtzia roseola*	WP_027001197.1	WP_027003762.1	WP_051203851.1
Bacteroidota	*Hwangdonia lutea*	WP_316983175.1	WP_316983375.1	WP_316982178.1
Bacteroidota	*Hwangdonia seohaensis*	WP_311938626.1	WP_311938917.1	WP_311939926.1
Bacteroidota	*Hwangdonia sp.*	WP_418604453.1	WP_418604389.1	WP_418603464.1
Bacteroidota	*Hydrobacter penzbergensis*	WP_092724081.1	WP_092725408.1	WP_092722670.1
Bacteroidota	*Hydrotalea flava*	WP_068247804.1	WP_068244712.1	WP_068244803.1
Bacteroidota	*Hydrotalea sandarakina*	WP_111297305.1	WP_211307670.1	WP_111293278.1
Bacteroidota	*Hydrotalea sp.*	WP_298412767.1	WP_298298368.1	WP_288071285.1
Bacteroidota	*Hymenobacter actinosclerus*	WP_092772166.1	WP_092770383.1	WP_092773236.1
Bacteroidota	*Hymenobacter aerilatus*	WP_245091095.1	WP_245096394.1	WP_245092938.1
Bacteroidota	*Hymenobacter aerophilus*	WP_019947749.1	WP_019946669.1	WP_019946883.1
Bacteroidota	*Hymenobacter algoricola*	WP_345113237.1	WP_345114690.1	WP_345109111.1
Bacteroidota	*Hymenobacter amundsenii*	WP_088464214.1	WP_088464474.1	WP_088462720.1
Bacteroidota	*Hymenobacter antarcticus*	WP_345121348.1	WP_345123254.1	WP_345125416.1
Bacteroidota	*Hymenobacter aquaticus*	WP_210114293.1	WP_135461986.1	WP_135463988.1
Bacteroidota	*Hymenobacter aranciens*	WP_305005511.1	WP_305005013.1	WP_305006523.1

Bacteroidota	*Hymenobacter arizonensis*	WP_092673250.1	WP_092670262.1	WP_092670509.1
Bacteroidota	*Hymenobacter armeniacus*	WP_190923097.1	WP_190922562.1	WP_190924915.1
Bacteroidota	*Hymenobacter artigasi*	WP_168675017.1	WP_168672938.1	WP_168672520.1
Bacteroidota	*Hymenobacter baengnokdamensis*	WP_151087692.1	WP_151088453.1	WP_151086729.1
Bacteroidota	*Hymenobacter bucti*	WP_382316155.1	WP_382313791.1	WP_382315386.1
Bacteroidota	*Hymenobacter busanensis*	WP_151079239.1	WP_338074383.1	WP_151078604.1
Bacteroidota	*Hymenobacter caeli*	WP_173808102.1	WP_317171099.1	WP_173808997.1
Bacteroidota	*Hymenobacter canadensis*	WP_269560067.1	WP_269561050.1	WP_269558985.1
Bacteroidota	*Hymenobacter cavernae*	WP_188815435.1	WP_188813621.1	WP_188811646.1
Bacteroidota	*Hymenobacter chitinivorans*	WP_100334769.1	WP_100336020.1	WP_100337998.1
Bacteroidota	*Hymenobacter citatus*	WP_187319983.1	WP_187321137.1	WP_187319347.1
Bacteroidota	*Hymenobacter coccineus*	WP_070739286.1	WP_070744121.1	WP_070747050.1
Bacteroidota	*Hymenobacter crusticola*	WP_086594948.1	WP_086593780.1	WP_086592258.1
Bacteroidota	*Hymenobacter cyanobacteriorum*	WP_241937162.1	WP_241935769.1	WP_241936268.1
Bacteroidota	*Hymenobacter daecheongensis*	WP_073108984.1	WP_073111368.1	WP_073105191.1
Bacteroidota	*Hymenobacter duratus*	WP_190784662.1	WP_190785735.1	WP_190783158.1
Bacteroidota	*Hymenobacter edaphi*	WP_111478724.1	WP_111476768.1	WP_111477643.1
Bacteroidota	*Hymenobacter elongatus*	WP_135499093.1	WP_135499484.1	WP_135498247.1
Bacteroidota	*Hymenobacter endophyticus*	WP_315999127.1	WP_315997629.1	WP_315997288.1
Bacteroidota	*Hymenobacter fastidiosus*	WP_345074853.1	WP_345072700.1	WP_345071857.1
Bacteroidota	*Hymenobacter fodinae*	WP_135433580.1	WP_135436110.1	WP_135430550.1
Bacteroidota	*Hymenobacter frigidus*	WP_188561526.1	WP_188560312.1	WP_188561206.1
Bacteroidota	*Hymenobacter gelipurpurascens*	WP_088843741.1	WP_088842140.1	WP_088844610.1
Bacteroidota	*Hymenobacter ginkgonis*	WP_157568925.1	WP_157562471.1	WP_157563796.1
Bacteroidota	*Hymenobacter glacialis*	WP_070734572.1	WP_070731728.1	WP_070735962.1
Bacteroidota	*Hymenobacter glaciei*	WP_345051671.1	WP_345050062.1	WP_345056698.1
Bacteroidota	*Hymenobacter glacieicola*	WP_188559509.1	WP_188558562.1	WP_188556977.1
Bacteroidota	*Hymenobacter gummosus*	WP_126696445.1	WP_126695648.1	WP_126694911.1

Bacteroidota	*Hymenobacter guriensis*	WP_196953312.1	WP_196955704.1	WP_196956148.1
Bacteroidota	*Hymenobacter jeollabukensis*	WP_138077952.1	WP_138074696.1	WP_138076488.1
Bacteroidota	*Hymenobacter jeongseonensis*	WP_196281111.1	WP_196283366.1	WP_196282795.1
Bacteroidota	*Hymenobacter koreensis*	WP_345220804.1	WP_345225765.1	WP_345227550.1
Bacteroidota	*Hymenobacter lapidiphilus*	WP_176907383.1	WP_176906325.1	WP_176910239.1
Bacteroidota	*Hymenobacter lucidus*	WP_226175553.1	WP_226177909.1	WP_226170797.1
Bacteroidota	*Hymenobacter lutimineralis*	WP_149070077.1	WP_149069064.1	WP_149069601.1
Bacteroidota	*Hymenobacter mellowenesis*	WP_305011453.1	WP_305014459.1	WP_305010093.1
Bacteroidota	*Hymenobacter metallicola*	WP_135394722.1	WP_135392312.1	WP_135395606.1
Bacteroidota	*Hymenobacter metallilatus*	WP_125427055.1	WP_125432977.1	WP_125426004.1
Bacteroidota	*Hymenobacter montanus*	WP_191003208.1	WP_191005164.1	WP_191003835.1
Bacteroidota	*Hymenobacter monticola*	WP_243508845.1	WP_243512292.1	WP_243516219.1
Bacteroidota	*Hymenobacter negativus*	WP_198066871.1	WP_198068458.1	WP_198069842.1
Bacteroidota	*Hymenobacter nitidus*	WP_226189119.1	WP_226183341.1	WP_226186023.1
Bacteroidota	*Hymenobacter nivis*	WP_140465004.1	WP_342767440.1	WP_109657402.1
Bacteroidota	*Hymenobacter norwichensis*	WP_022823951.1	WP_022823749.1	WP_022825075.1
Bacteroidota	*Hymenobacter oligotrophus*	WP_119444743.1	WP_119443979.1	WP_119445303.1
Bacteroidota	*Hymenobacter perfusus*	WP_125439651.1	WP_125440281.1	WP_125435945.1
Bacteroidota	*Hymenobacter persicinus*	WP_129922767.1	WP_129922804.1	WP_129923055.1
Bacteroidota	*Hymenobacter pini*	WP_226265934.1	WP_226268672.1	WP_226271625.1
Bacteroidota	*Hymenobacter piscis*	WP_215594052.1	WP_215592429.1	WP_215591981.1
Bacteroidota	*Hymenobacter polaris*	WP_169528951.1	WP_169530971.1	WP_169532678.1
Bacteroidota	*Hymenobacter profundi*	WP_219160458.1	WP_219159751.1	WP_219161744.1
Bacteroidota	*Hymenobacter properus*	WP_196287755.1	WP_196284682.1	WP_196285685.1
Bacteroidota	*Hymenobacter psoromatis*	WP_223648793.1	WP_223649629.1	WP_223653225.1
Bacteroidota	*Hymenobacter psychrophilus*	WP_092739454.1	WP_092741564.1	WP_092739666.1
Bacteroidota	*Hymenobacter psychrotolerans*	WP_073286580.1	WP_073287465.1	WP_073285851.1
Bacteroidota	*Hymenobacter qilianensis*	WP_187732856.1	WP_187732044.1	WP_187733782.1

Bacteroidota	*Hymenobacter rigui*	WP_125420006.1	WP_125423685.1	WP_125417329.1
Bacteroidota	*Hymenobacter roseosalivarius*	WP_084444424.1	WP_084447454.1	WP_084445205.1
Bacteroidota	*Hymenobacter rubidus*	WP_201986503.1	WP_201984882.1	WP_201984100.1
Bacteroidota	*Hymenobacter rubripertinctus*	WP_119656221.1	WP_119654072.1	WP_119654547.1
Bacteroidota	*Hymenobacter ruricola*	WP_196293568.1	WP_196291767.1	WP_196293999.1
Bacteroidota	*Hymenobacter saemangeumensis*	WP_345238436.1	WP_345237286.1	WP_345237946.1
Bacteroidota	*Hymenobacter sedentarius*	WP_068197752.1	WP_068195151.1	WP_068191064.1
Bacteroidota	*Hymenobacter sediminicola*	WP_185887369.1	WP_185886273.1	WP_185888447.1
Bacteroidota	*Hymenobacter sediminis*	WP_202910460.1	WP_110979241.1	WP_110976343.1
Bacteroidota	*Hymenobacter segetis*	WP_342300887.1	WP_342301024.1	WP_342295151.1
Bacteroidota	*Hymenobacter setariae*	WP_144851550.1	WP_144845586.1	WP_144847603.1
Bacteroidota	*Hymenobacter siberiensis*	WP_216727714.1	WP_216688865.1	WP_216679529.1
Bacteroidota	*Hymenobacter sp.*	WP_325135645.1	WP_310397190.1	WP_310393716.1
Bacteroidota	*Hymenobacter sublimis*	WP_247975982.1	WP_247976812.1	WP_247975169.1
Bacteroidota	*Hymenobacter swuensis*	WP_044001162.1	WP_044002076.1	WP_044003628.1
Bacteroidota	*Hymenobacter telluris*	WP_206982133.1	WP_206984410.1	WP_206986445.1
Bacteroidota	*Hymenobacter terrenus*	WP_046243211.1	WP_046243040.1	WP_046246906.1
Bacteroidota	*Hymenobacter terrestris*	WP_176900196.1	WP_176898993.1	WP_176901120.1
Bacteroidota	*Hymenobacter terricola*	WP_210518078.1	WP_210515764.1	WP_210513663.1
Bacteroidota	*Hymenobacter tibetensis*	WP_243800611.1	WP_243802657.1	WP_243799028.1
Bacteroidota	*Hymenobacter translucens*	WP_227609277.1	WP_227606247.1	WP_227610336.1
Bacteroidota	*Hymenobacter volaticus*	WP_245122496.1	WP_245125362.1	WP_245120586.1
Bacteroidota	*Hymenobacter wooponensis*	WP_135529106.1	WP_135530529.1	WP_135530138.1
Bacteroidota	*Hymenobacter yonginensis*	WP_270127571.1	WP_270128601.1	WP_270126306.1
Bacteroidota	*Hyunsoonleella aestuarii*	WP_139002770.1	WP_139000583.1	WP_139000362.1
Bacteroidota	*Hyunsoonleella aquatilis*	WP_186563531.1	WP_186563450.1	WP_186559073.1
Bacteroidota	*Hyunsoonleella flava*	WP_130962624.1	WP_130962429.1	WP_130963622.1
Bacteroidota	*Hyunsoonleella jejuensis*	WP_092578219.1	WP_092578669.1	WP_092579037.1

Bacteroidota	*Hyunsoonleella pacifica*	WP_130935536.1	WP_130935871.1	WP_130938023.1
Bacteroidota	*Hyunsoonleella rubra*	WP_380288079.1	WP_380289797.1	WP_380292807.1
Bacteroidota	*Hyunsoonleella sp. 2307UL5-6*	WP_413972052.1	WP_413973366.1	WP_413973261.1
Bacteroidota	*Hyunsoonleella ulvae*	WP_203256821.1	WP_203258597.1	WP_203257890.1
Bacteroidota	*Ichthyenterobacterium magnum*	WP_120200732.1	WP_120201318.1	WP_120199279.1
Bacteroidota	*Ichthyobacterium seriolicida*	WP_096686479.1	WP_096686568.1	WP_096685584.1
Bacteroidota	*Ilyomonas limi*	WP_137261080.1	WP_137261789.1	WP_137263876.1
Bacteroidota	*Imperialibacter roseus*	WP_317488528.1	WP_317492116.1	WP_317489223.1
Bacteroidota	*Imperialibacter sp.*	WP_350078291.1	WP_350095892.1	WP_350117640.1
Bacteroidota	*Imtechella halotolerans*	WP_008240408.1	WP_008238821.1	WP_008238112.1
Bacteroidota	*Indibacter alkaliphilus*	WP_009032885.1	WP_009036264.1	WP_009033840.1
Bacteroidota	*Jejudonia soesokkakensis*	WP_380216994.1	WP_380217385.1	WP_380218067.1
Bacteroidota	*Jejuia pallidilutea*	WP_042243901.1	WP_105474849.1	WP_105474438.1
Bacteroidota	*Jejuia sp. DST062*	WP_406971608.1	WP_406969494.1	WP_406968753.1
Bacteroidota	*Jejuia spongiicola*	WP_249972989.1	WP_249973577.1	WP_249972470.1
Bacteroidota	*Jiulongibacter sediminis*	WP_304235437.1	WP_055145038.1	WP_304237026.1
Bacteroidota	*Joostella atrarenae*	WP_236959290.1	WP_236959699.1	WP_236958707.1
Bacteroidota	*Joostella sp.*	WP_417444730.1	WP_417444046.1	WP_417444373.1
Bacteroidota	*Kaistella antarctica*	WP_034718549.1	WP_034718534.1	WP_051803858.1
Bacteroidota	*Kaistella carnis*	WP_125025455.1	WP_125025449.1	WP_313502080.1
Bacteroidota	*Kaistella chaponensis*	WP_324472738.1	WP_076386374.1	WP_076387281.1
Bacteroidota	*Kaistella daneshvariae*	WP_123266406.1	WP_123266400.1	WP_123266721.1
Bacteroidota	*Kaistella faecalis*	WP_218248450.1	WP_218248442.1	WP_218250327.1
Bacteroidota	*Kaistella flava (ex Peng et al. 2021)*	WP_193811537.1	WP_193811529.1	WP_193811028.1
Bacteroidota	*Kaistella gelatinilytica*	WP_196079956.1	WP_196079963.1	WP_196080199.1
Bacteroidota	*Kaistella haifensis*	WP_031501350.1	WP_031501345.1	WP_031504621.1
Bacteroidota	*Kaistella jeonii*	WP_039351184.1	WP_039351160.1	WP_039350477.1
Bacteroidota	*Kaistella montana*	WP_255929243.1	WP_255929267.1	WP_255930280.1

Bacteroidota	*Kaistella palustris*	WP_027376032.1	WP_027376024.1	WP_027377070.1
Bacteroidota	*Kaistella polysaccharea*	WP_226063347.1	WP_226063355.1	WP_226063808.1
Bacteroidota	*Kaistella soli*	WP_088358885.1	WP_217201330.1	WP_217201368.1
Bacteroidota	*Kaistella solincola*	WP_039342678.1	WP_039341850.1	WP_039341001.1
Bacteroidota	*Kaistella sp.*	WP_332031973.1	WP_332031967.1	WP_423974762.1
Bacteroidota	*Kaistella treverensis*	WP_089819909.1	WP_089819511.1	WP_089819931.1
Bacteroidota	*Kaistella yananensis*	WP_265142890.1	WP_265142884.1	WP_265144070.1
Bacteroidota	*Kaistella yonginensis*	WP_290216955.1	WP_290216940.1	WP_290217812.1
Bacteroidota	*Kordia aestuariivivens*	WP_187563470.1	WP_187561095.1	WP_187564525.1
Bacteroidota	*Kordia algicida*	WP_007093984.1	WP_040559864.1	WP_007094736.1
Bacteroidota	*Kordia antarctica*	WP_160128089.1	WP_160128402.1	WP_160131354.1
Bacteroidota	*Kordia jejudonensis*	WP_046758503.1	WP_046758012.1	WP_046757471.1
Bacteroidota	*Kordia periserrulae*	WP_108114659.1	WP_108114435.1	WP_108116523.1
Bacteroidota	*Kordia sp.*	WP_405302502.1	WP_420572871.1	WP_405302795.1
Bacteroidota	*Kordia zhangzhouensis*	WP_046744617.1	WP_046744409.1	WP_046745889.1
Bacteroidota	*Kriegella aquimaris*	WP_089885287.1	WP_089884735.1	WP_089894501.1
Bacteroidota	*Lacibacter cauensis*	WP_144886451.1	WP_144884137.1	WP_144886127.1
Bacteroidota	*Lacibacter luteus*	WP_129131595.1	WP_129128817.1	WP_129132186.1
Bacteroidota	*Lacibacter sediminis*	WP_182803974.1	WP_182801129.1	WP_182801935.1
Bacteroidota	*Lacibacter sp.*	WP_324231404.1	WP_324232070.1	WP_324230093.1
Bacteroidota	*Lacihabitans lacunae*	WP_379835146.1	WP_379834768.1	WP_379838877.1
Bacteroidota	*Lacihabitans soyangensis*	WP_255038281.1	WP_255039040.1	WP_255037505.1
Bacteroidota	*Lacihabitans sp. CCS-44*	WP_255080260.1	WP_255076565.1	WP_255077870.1
Bacteroidota	*Lacinutrix algicola*	WP_055437101.1	WP_055437076.1	WP_055435440.1
Bacteroidota	*Lacinutrix gracilariae*	WP_379901067.1	WP_379899746.1	WP_379902270.1
Bacteroidota	*Lacinutrix himadriensis*	WP_055444954.1	WP_055443481.1	WP_055445236.1
Bacteroidota	*Lacinutrix iliipiscaria*	WP_183487450.1	WP_183488070.1	WP_183486898.1
Bacteroidota	*Lacinutrix jangbogonensis*	WP_034058557.1	WP_034059251.1	WP_034057457.1

Bacteroidota	*Lacinutrix mariniflava*	WP_055446055.1	WP_055448589.1	WP_055448788.1
Bacteroidota	*Lacinutrix sp.*	WP_340169257.1	WP_340169113.1	WP_290698518.1
Bacteroidota	*Lacinutrix venerupis*	WP_121048868.1	WP_076733424.1	WP_121052254.1
Bacteroidota	*Larkinella arboricola*	WP_111628622.1	WP_111628276.1	WP_111628983.1
Bacteroidota	*Larkinella bovis*	WP_379847279.1	WP_379840490.1	WP_379846488.1
Bacteroidota	*Larkinella humicola*	WP_150876445.1	WP_150874987.1	WP_150877174.1
Bacteroidota	*Larkinella insperata*	WP_265992729.1	WP_265990224.1	WP_265993129.1
Bacteroidota	*Larkinella knui*	WP_124910643.1	WP_124904358.1	WP_124909924.1
Bacteroidota	*Larkinella rosea*	WP_124875391.1	WP_124870784.1	WP_124874597.1
Bacteroidota	*Larkinella soli*	WP_128548767.1	WP_128546727.1	WP_128545547.1
Bacteroidota	*Larkinella sp.*	WP_421828999.1	WP_421830674.1	WP_421827887.1
Bacteroidota	*Larkinella terrae*	WP_154172480.1	WP_154176098.1	WP_154174654.1
Bacteroidota	*Leadbetterella byssophila*	WP_013407413.1	WP_412814525.1	WP_412814608.1
Bacteroidota	*Leadbetterella sp. DM7*	WP_367915067.1	WP_367915579.1	WP_367913745.1
Bacteroidota	*Leeuwenhoekiella aequorea*	WP_128758357.1	WP_128757498.1	WP_128758662.1
Bacteroidota	*Leeuwenhoekiella aestuarii*	WP_128760275.1	WP_128762123.1	WP_128760454.1
Bacteroidota	*Leeuwenhoekiella marinoflava*	WP_335620825.1	WP_073099091.1	WP_335620663.1
Bacteroidota	*Leeuwenhoekiella nanhaiensis*	WP_099646209.1	WP_099646328.1	WP_099645389.1
Bacteroidota	*Leeuwenhoekiella palythoae*	WP_225597923.1	WP_072979912.1	WP_370174603.1
Bacteroidota	*Leeuwenhoekiella parthenopeia*	WP_228230864.1	WP_228230944.1	WP_228228275.1
Bacteroidota	*Leeuwenhoekiella polynyae*	WP_128764228.1	WP_128763846.1	WP_128765667.1
Bacteroidota	*Leeuwenhoekiella sp. NPDC079379*	WP_396511161.1	WP_396512396.1	WP_396510153.1
Bacteroidota	*Lentiprolixibacter aurantiacus*	WP_266013079.1	WP_266012786.1	WP_266013347.1
Bacteroidota	*Leptobacterium flavescens*	WP_163607265.1	WP_163607288.1	WP_163605276.1
Bacteroidota	*Leptobacterium meishanense*	WP_340077561.1	WP_340073967.1	WP_340074705.1
Bacteroidota	*Lewinella cohaerens*	WP_020536610.1	WP_020536903.1	WP_020538753.1
Bacteroidota	*Lewinella sp. LCG006*	WP_367391533.1	WP_367388024.1	WP_367390254.1
Bacteroidota	*Limibacter armeniacum*	WP_334246576.1	WP_334246496.1	WP_334246523.1

Bacteroidota	*Limnovirga soli*	WP_171609007.1	WP_171609473.1	WP_171609767.1
Bacteroidota	*Lishizhenia sp.*	WP_318958285.1	WP_318958486.1	WP_318958685.1
Bacteroidota	*Lishizhenia tianjinensis*	WP_090246723.1	WP_090247519.1	WP_090245381.1
Bacteroidota	*Litoribacter populi*	WP_143962451.1	WP_143960986.1	WP_143959597.1
Bacteroidota	*Litoribacter ruber*	WP_213943340.1	WP_213945420.1	WP_214162654.1
Bacteroidota	*Litoribaculum gwangyangense*	WP_345276104.1	WP_345276075.1	WP_345276767.1
Bacteroidota	*Longitalea arenae*	WP_205508771.1	WP_205512896.1	WP_205509770.1
Bacteroidota	*Longitalea luteola*	WP_207511955.1	WP_207513435.1	WP_207513616.1
Bacteroidota	*Lunatibacter salilacus*	WP_158858628.1	WP_158857098.1	WP_158855923.1
Bacteroidota	*Lunatimonas lonarensis*	WP_010853832.1	WP_035805042.1	WP_010856401.1
Bacteroidota	*Lunatimonas salinarum*	WP_209329637.1	WP_209330338.1	WP_209332122.1
Bacteroidota	*Lunatimonas sp.*	WP_228693011.1	WP_228691852.1	WP_228692448.1
Bacteroidota	*Luteibaculum oceani*	WP_147014709.1	WP_147014190.1	WP_147013057.1
Bacteroidota	*Luteirhabdus pelagi*	WP_203295780.1	WP_203296683.1	WP_203296609.1
Bacteroidota	*Lutibacter agarilyticus*	WP_089380012.1	WP_089380156.1	WP_089381860.1
Bacteroidota	*Lutibacter citreus*	WP_111708460.1	WP_111707821.1	WP_111708609.1
Bacteroidota	*Lutibacter flavus*	WP_089377124.1	WP_089376832.1	WP_089377379.1
Bacteroidota	*Lutibacter holmesii*	WP_386809209.1	WP_386809111.1	WP_386807184.1
Bacteroidota	*Lutibacter maritimus*	WP_090222967.1	WP_090222428.1	WP_090221845.1
Bacteroidota	*Lutibacter oceani*	WP_115879279.1	WP_115879655.1	WP_115882099.1
Bacteroidota	*Lutibacter oricola*	WP_090121462.1	WP_090120722.1	WP_090123020.1
Bacteroidota	*Lutibacter profundi*	WP_068205635.1	WP_068205690.1	WP_068206420.1
Bacteroidota	*Lutibacter sp.*	WP_408670705.1	WP_291970443.1	WP_291975456.1
Bacteroidota	*Lutimonas halocynthiae*	WP_290293069.1	WP_290293732.1	WP_290289555.1
Bacteroidota	*Lutimonas saemankumensis*	WP_224927446.1	WP_224927476.1	WP_224932014.1
Bacteroidota	*Lutimonas sp.*	WP_424350756.1	WP_424350450.1	WP_424352018.1
Bacteroidota	*Lutimonas vermicola*	WP_342158194.1	WP_342159627.1	WP_342159791.1
Bacteroidota	*Lutimonas zeaxanthinifaciens*	WP_302043014.1	WP_302043042.1	WP_302043417.1

Bacteroidota	*Mangrovimonas aestuarii*	WP_274475567.1	WP_274475634.1	WP_274475942.1
Bacteroidota	*Mangrovimonas cancribranchiae*	WP_338733894.1	WP_338733664.1	WP_338733268.1
Bacteroidota	*Mangrovimonas futianensis*	WP_232634821.1	WP_232395550.1	WP_232634478.1
Bacteroidota	*Mangrovimonas sp. DI 80*	WP_076663859.1	WP_076664802.1	WP_076663450.1
Bacteroidota	*Mangrovimonas spongiae*	WP_125468490.1	WP_125467189.1	WP_125467469.1
Bacteroidota	*Mangrovimonas xylaniphaga*	WP_053977790.1	WP_053977261.1	WP_053977459.1
Bacteroidota	*Mangrovimonas yunxiaonensis*	WP_036123792.1	WP_036123026.1	WP_036122376.1
Bacteroidota	*Mangrovivirga halotolerans*	WP_266058647.1	WP_266056794.1	WP_266057182.1
Bacteroidota	*Maribacter aestuarii*	WP_289644541.1	WP_281543132.1	WP_289644842.1
Bacteroidota	*Maribacter algarum*	WP_138659431.1	WP_138658534.1	WP_138658835.1
Bacteroidota	*Maribacter algicola*	WP_125223587.1	WP_125223285.1	WP_125224015.1
Bacteroidota	*Maribacter antarcticus*	WP_027077533.1	WP_027075876.1	WP_027075275.1
Bacteroidota	*Maribacter aquimaris*	WP_188244283.1	WP_188241762.1	WP_188244462.1
Bacteroidota	*Maribacter aquivivus*	WP_073246261.1	WP_073243963.1	WP_282051155.1
Bacteroidota	*Maribacter arcticus*	WP_079512125.1	WP_335637209.1	WP_335637955.1
Bacteroidota	*Maribacter arenosus*	WP_188312956.1	WP_188312492.1	WP_188315229.1
Bacteroidota	*Maribacter aurantiacus*	WP_138259457.1	WP_138258082.1	WP_138259233.1
Bacteroidota	*Maribacter caenipelagi*	WP_133673642.1	WP_133671069.1	WP_133673480.1
Bacteroidota	*Maribacter chungangensis*	WP_379935338.1	WP_379934434.1	WP_379934574.1
Bacteroidota	*Maribacter cobaltidurans*	WP_094995491.1	WP_094998851.1	WP_094997970.1
Bacteroidota	*Maribacter confluentis*	WP_304437470.1	WP_304437268.1	WP_304435015.1
Bacteroidota	*Maribacter dokdonensis*	WP_210709020.1	WP_348427586.1	WP_321828019.1
Bacteroidota	*Maribacter forsetii*	WP_036153520.1	WP_036154123.1	WP_036155838.1
Bacteroidota	*Maribacter halichondriae*	WP_273566554.1	WP_273566662.1	WP_337251723.1
Bacteroidota	*Maribacter huludaoensis*	WP_276473091.1	WP_276473340.1	WP_276472642.1
Bacteroidota	*Maribacter hydrothermalis*	WP_068482505.1	WP_068484963.1	WP_068484075.1
Bacteroidota	*Maribacter litoralis*	WP_116772091.1	WP_116768890.1	WP_159301745.1
Bacteroidota	*Maribacter luteus*	WP_282056634.1	WP_282054775.1	WP_154368496.1

Bacteroidota	*Maribacter orientalis*	WP_091627189.1	WP_091625859.1	WP_091626195.1
Bacteroidota	*Maribacter polysaccharolyticus*	WP_274826574.1	WP_274823406.1	WP_274824402.1
Bacteroidota	*Maribacter polysiphoniae*	WP_109652914.1	WP_109654071.1	WP_273276891.1
Bacteroidota	*Maribacter sp.*	WP_291960873.1	WP_291964572.1	WP_348369336.1
Bacteroidota	*Maribacter spongiicola*	WP_133687150.1	WP_133687444.1	WP_133688997.1
Bacteroidota	*Maribacter stanieri*	WP_282113329.1	WP_339725903.1	WP_282113675.1
Bacteroidota	*Maribacter thermophilus*	WP_047245800.1	WP_047245436.1	WP_047244756.1
Bacteroidota	*Maribacter ulvicola*	WP_076548560.1	WP_076549865.1	WP_076549380.1
Bacteroidota	*Maribacter vaceletii*	WP_121069140.1	WP_121065987.1	WP_121069339.1
Bacteroidota	*Maribacter zhoushanensis*	WP_276494026.1	WP_276493126.1	WP_276494673.1
Bacteroidota	*Mariniflexile aquimaris*	WP_379942415.1	WP_379942788.1	WP_379941225.1
Bacteroidota	*Mariniflexile fucanivorans*	WP_132219156.1	WP_132215582.1	WP_132216338.1
Bacteroidota	*Mariniflexile gromovii*	WP_209656289.1	WP_209657102.1	WP_209654042.1
Bacteroidota	*Mariniflexile jejuense*	WP_379924078.1	WP_379926576.1	WP_379926525.1
Bacteroidota	*Mariniflexile litorale*	WP_308992851.1	WP_308992244.1	WP_308992065.1
Bacteroidota	*Mariniflexile maritimum*	WP_157206636.1	WP_157208718.1	WP_157206179.1
Bacteroidota	*Mariniflexile ostreae*	WP_379860781.1	WP_379861361.1	WP_379860627.1
Bacteroidota	*Mariniflexile soesokkakense*	WP_346242016.1	WP_346239891.1	WP_346242332.1
Bacteroidota	*Mariniflexile sp.*	WP_372755176.1	WP_372754476.1	WP_372754686.1
Bacteroidota	*Marinigracilibium pacificum*	WP_169684667.1	WP_169684819.1	WP_169677532.1
Bacteroidota	*Marinilongibacter aquaticus*	WP_261451105.1	WP_261449973.1	WP_261447952.1
Bacteroidota	*Mariniradius saccharolyticus*	WP_008627210.1	WP_008628996.1	WP_040480123.1
Bacteroidota	*Mariniradius sediminis*	WP_234862381.1	WP_234862495.1	WP_234861300.1
Bacteroidota	*Marinirhabdus gelatinilytica*	WP_115124645.1	WP_115123303.1	WP_115123992.1
Bacteroidota	*Marinoscillum furvescens*	WP_115866794.1	WP_245986162.1	WP_115868181.1
Bacteroidota	*Marinoscillum pacificum*	WP_258099391.1	WP_258099852.1	WP_258100254.1
Bacteroidota	*Marinoscillum sp.*	WP_421873655.1	WP_421878018.1	WP_421892226.1
Bacteroidota	*Marivirga arenosa*	WP_308356037.1	WP_308356793.1	WP_308357795.1

Bacteroidota	*Marivirga atlantica*	WP_201918138.1	WP_201924100.1	WP_201916581.1
Bacteroidota	*Marivirga aurantiaca*	WP_201432316.1	WP_201429891.1	WP_201432555.1
Bacteroidota	*Marivirga harenae*	WP_303267928.1	WP_303272113.1	WP_303270879.1
Bacteroidota	*Marivirga lumbricoides*	WP_188460934.1	WP_188462202.1	WP_188465104.1
Bacteroidota	*Marivirga salinae*	WP_308351288.1	WP_308347826.1	WP_308349786.1
Bacteroidota	*Marivirga sericea*	WP_085517122.1	WP_085515726.1	WP_085518009.1
Bacteroidota	*Marivirga sp.*	WP_296618831.1	WP_296622034.1	WP_325121820.1
Bacteroidota	*Marivirga tractuosa*	WP_375580939.1	WP_375577921.1	WP_013454056.1
Bacteroidota	*Marixanthomonas ophiurae*	WP_117160044.1	WP_117160475.1	WP_117159718.1
Bacteroidota	*Marixanthomonas sp. SCSIO 43207*	WP_223107920.1	WP_223107512.1	WP_223109995.1
Bacteroidota	*Marixanthomonas spongiae*	WP_116693667.1	WP_116693396.1	WP_116694569.1
Bacteroidota	*Marixanthotalea marina*	WP_318543194.1	WP_318543261.1	WP_318542961.1
Bacteroidota	*Marnyiella aurantia*	WP_181886854.1	WP_209815644.1	WP_209814436.1
Bacteroidota	*Membranihabitans marinus*	WP_236970699.1	WP_236969875.1	WP_236969502.1
Bacteroidota	*Membranihabitans maritimus*	WP_236974510.1	WP_236979183.1	WP_236979239.1
Bacteroidota	*Mesoflavibacter sp.*	WP_370225638.1	WP_370228053.1	WP_370225799.1
Bacteroidota	*Mesoflavibacter zeaxanthinifaciens*	WP_417557548.1	WP_027879723.1	WP_027880087.1
Bacteroidota	*Mesohalobacter halotolerans*	WP_138931676.1	WP_138932211.1	WP_138932670.1
Bacteroidota	*Mesonia aestuariivivens*	WP_219039071.1	WP_219040755.1	WP_219041146.1
Bacteroidota	*Mesonia algae*	WP_111540188.1	WP_111541586.1	WP_111541566.1
Bacteroidota	*Mesonia aquimarina*	WP_121665309.1	WP_121665496.1	WP_121666282.1
Bacteroidota	*Mesonia hippocampi*	WP_183477614.1	WP_183475703.1	WP_183477477.1
Bacteroidota	*Mesonia maritima*	WP_309729227.1	WP_309728181.1	WP_309728893.1
Bacteroidota	*Mesonia mobilis*	WP_304137891.1	WP_304038444.1	WP_304136972.1
Bacteroidota	*Mesonia ostreae*	WP_311401302.1	WP_311400781.1	WP_311400862.1
Bacteroidota	*Mesonia phycicola*	WP_073148113.1	WP_073149804.1	WP_073149941.1
Bacteroidota	*Mesonia profundi*	WP_308863989.1	WP_308864680.1	WP_308863498.1
Bacteroidota	*Mesonia sediminis*	WP_379044484.1	WP_379046647.1	WP_379045708.1

Bacteroidota	*Mesonia sp.*	WP_292248828.1	WP_292246739.1	WP_347418592.1
Bacteroidota	*Microcosmobacter mediterraneus*	WP_311427926.1	WP_311426391.1	WP_311425829.1
Bacteroidota	*Microscilla marina*	WP_002698220.1	WP_002693779.1	WP_002700262.1
Bacteroidota	*Moheibacter lacus*	WP_182044234.1	WP_182043139.1	WP_182042476.1
Bacteroidota	*Moheibacter sediminis*	WP_084017991.1	WP_084017945.1	WP_159447435.1
Bacteroidota	*Moheibacter sp.*	WP_424368457.1	WP_424367864.1	WP_424368122.1
Bacteroidota	*Moheibacter stercoris*	WP_354510703.1	WP_354506512.1	WP_354505341.1
Bacteroidota	*Mongoliibacter ruber*	WP_106133080.1	WP_106132015.1	WP_106134676.1
Bacteroidota	*Mongoliibacter sp.*	WP_293012583.1	WP_293012793.1	WP_293011822.1
Bacteroidota	*Mongoliitalea daihaiensis*	WP_236139112.1	WP_236136828.1	WP_236139309.1
Bacteroidota	*Mongoliitalea lutea*	WP_189579276.1	WP_189585048.1	WP_189579197.1
Bacteroidota	*Mucilaginibacter agri*	WP_166584801.1	WP_166584559.1	WP_166587319.1
Bacteroidota	*Mucilaginibacter angelicae*	WP_377026524.1	WP_377023939.1	WP_377021327.1
Bacteroidota	*Mucilaginibacter antarcticus*	WP_377126946.1	WP_377129763.1	WP_377128107.1
Bacteroidota	*Mucilaginibacter aquaedulcis*	WP_290327273.1	WP_290321937.1	WP_290325587.1
Bacteroidota	*Mucilaginibacter aquariorum*	WP_256540140.1	WP_256539051.1	WP_256538611.1
Bacteroidota	*Mucilaginibacter aquatilis*	WP_157540593.1	WP_317163585.1	WP_157539959.1
Bacteroidota	*Mucilaginibacter arboris*	WP_157566211.1	WP_157564779.1	WP_157567059.1
Bacteroidota	*Mucilaginibacter auburnensis*	WP_100340163.1	WP_100340579.1	WP_100342736.1
Bacteroidota	*Mucilaginibacter aurantiaciroseus*	WP_252759152.1	WP_252758200.1	WP_252755998.1
Bacteroidota	*Mucilaginibacter boryungensis*	WP_194107392.1	WP_194105065.1	WP_194106909.1
Bacteroidota	*Mucilaginibacter calamicampi*	WP_377100119.1	WP_377096542.1	WP_377101964.1
Bacteroidota	*Mucilaginibacter celer*	WP_119406908.1	WP_119410587.1	WP_119409068.1
Bacteroidota	*Mucilaginibacter conchicola*	WP_117392726.1	WP_117393950.1	WP_117394106.1
Bacteroidota	*Mucilaginibacter corticis*	WP_144246735.1	WP_144249890.1	WP_144247614.1
Bacteroidota	*Mucilaginibacter daejeonensis*	WP_228170624.1	WP_228173292.1	WP_228171161.1
Bacteroidota	*Mucilaginibacter defluvii*	WP_345328736.1	WP_345331670.1	WP_345334012.1
Bacteroidota	*Mucilaginibacter dorajii*	WP_259095654.1	WP_259088923.1	WP_259092943.1

Bacteroidota	*Mucilaginibacter endophyticus*	WP_114939907.1	WP_114936456.1	WP_114939283.1
Bacteroidota	*Mucilaginibacter flavidus*	WP_252792180.1	WP_252787898.1	WP_252790012.1
Bacteroidota	*Mucilaginibacter flavus*	WP_290306153.1	WP_290305038.1	WP_290304195.1
Bacteroidota	*Mucilaginibacter frigoritolerans*	WP_144913145.1	WP_144911351.1	WP_144914774.1
Bacteroidota	*Mucilaginibacter galii*	WP_188413953.1	WP_188415640.1	WP_377169483.1
Bacteroidota	*Mucilaginibacter gilvus*	WP_128535439.1	WP_128534534.1	WP_128536213.1
Bacteroidota	*Mucilaginibacter ginkgonis*	WP_157525657.1	WP_157526112.1	WP_157525403.1
Bacteroidota	*Mucilaginibacter ginsenosidivorans*	WP_147030842.1	WP_147033817.1	WP_147031469.1
Bacteroidota	*Mucilaginibacter ginsenosidivorax*	WP_147054731.1	WP_147052744.1	WP_147055663.1
Bacteroidota	*Mucilaginibacter glaciei*	WP_191163871.1	WP_191164270.1	WP_191162443.1
Bacteroidota	*Mucilaginibacter gossypiicola*	WP_091215239.1	WP_091222143.1	WP_317040865.1
Bacteroidota	*Mucilaginibacter gotjawali*	WP_096349449.1	WP_096350522.1	WP_183476404.1
Bacteroidota	*Mucilaginibacter gracilis*	WP_121201357.1	WP_121198186.1	WP_121197576.1
Bacteroidota	*Mucilaginibacter gynuensis*	WP_345210895.1	WP_345213621.1	WP_345214337.1
Bacteroidota	*Mucilaginibacter hurinus*	WP_114005415.1	WP_114003385.1	WP_114005266.1
Bacteroidota	*Mucilaginibacter inviolabilis*	WP_166092465.1	WP_166089827.1	WP_166091221.1
Bacteroidota	*Mucilaginibacter jinjuensis*	WP_273630002.1	WP_273632286.1	WP_273629463.1
Bacteroidota	*Mucilaginibacter kameinonensis*	WP_121808705.1	WP_121809185.1	WP_121808023.1
Bacteroidota	*Mucilaginibacter lacusdianchii*	WP_158825378.1	WP_158824798.1	WP_158827222.1
Bacteroidota	*Mucilaginibacter lappiensis*	WP_076371559.1	WP_076374166.1	WP_317617672.1
Bacteroidota	*Mucilaginibacter limnophilus*	WP_127707196.1	WP_127706749.1	WP_127703902.1
Bacteroidota	*Mucilaginibacter litoreus*	WP_377116600.1	WP_377111737.1	WP_377111387.1
Bacteroidota	*Mucilaginibacter lutimaris*	WP_377143437.1	WP_377145568.1	WP_377142764.1
Bacteroidota	*Mucilaginibacter mali*	WP_173416735.1	WP_173414054.1	WP_173415910.1
Bacteroidota	*Mucilaginibacter mallensis*	WP_091378892.1	WP_091370817.1	WP_091376794.1
Bacteroidota	*Mucilaginibacter myungsuensis*	WP_194110220.1	WP_194112504.1	WP_194112018.1
Bacteroidota	*Mucilaginibacter oryzae*	WP_109609816.1	WP_109608412.1	WP_109609481.1
Bacteroidota	*Mucilaginibacter pallidiroseus*	WP_146380790.1	WP_146382585.1	WP_146382325.1

Bacteroidota	*Mucilaginibacter paludis*	WP_040627802.1	WP_008511304.1	WP_008505046.1
Bacteroidota	*Mucilaginibacter panaciglaebae*	WP_345105444.1	WP_345106671.1	WP_345104577.1
Bacteroidota	*Mucilaginibacter pankratovii*	WP_191190127.1	WP_191191809.1	WP_191191606.1
Bacteroidota	*Mucilaginibacter pedocola*	WP_078349397.1	WP_078348817.1	WP_078348021.1
Bacteroidota	*Mucilaginibacter phenanthrenivorans*	WP_258138196.1	WP_258137176.1	WP_258138502.1
Bacteroidota	*Mucilaginibacter phyllosphaerae*	WP_134337205.1	WP_134334969.1	WP_134335263.1
Bacteroidota	*Mucilaginibacter pineti*	WP_091149399.1	WP_091145974.1	WP_091149996.1
Bacteroidota	*Mucilaginibacter pocheonensis*	WP_310095523.1	WP_310091551.1	WP_310103339.1
Bacteroidota	*Mucilaginibacter polytrichastri*	WP_074490993.1	WP_216351114.1	WP_074490320.1
Bacteroidota	*Mucilaginibacter psychrotolerans*	WP_133233713.1	WP_133228008.1	WP_133230210.1
Bacteroidota	*Mucilaginibacter rigui*	WP_191177093.1	WP_191173972.1	WP_191173641.1
Bacteroidota	*Mucilaginibacter rivuli*	WP_219489402.1	WP_219489650.1	WP_219489433.1
Bacteroidota	*Mucilaginibacter robiniae*	WP_169606372.1	WP_169605812.1	WP_169605632.1
Bacteroidota	*Mucilaginibacter roseus*	WP_232179103.1	WP_232178139.1	WP_232177772.1
Bacteroidota	*Mucilaginibacter rubeus*	WP_112574782.1	WP_112567222.1	WP_112575728.1
Bacteroidota	*Mucilaginibacter sabulilitoris*	WP_321563156.1	WP_321562246.1	WP_321562877.1
Bacteroidota	*Mucilaginibacter segetis*	WP_200063558.1	WP_200063914.1	WP_200066997.1
Bacteroidota	*Mucilaginibacter sp.*	WP_299359762.1	WP_343114049.1	WP_369591216.1
Bacteroidota	*Mucilaginibacter straminoryzae*	WP_245131972.1	WP_245129948.1	WP_245129198.1
Bacteroidota	*Mucilaginibacter terrae*	WP_311951396.1	WP_377161838.1	WP_311949776.1
Bacteroidota	*Mucilaginibacter terrenus*	WP_117384536.1	WP_117382517.1	WP_117382866.1
Bacteroidota	*Mucilaginibacter terrigena*	WP_129878240.1	WP_129875404.1	WP_129876957.1
Bacteroidota	*Mucilaginibacter ximonensis*	WP_377184449.1	WP_377187730.1	WP_377186185.1
Bacteroidota	*Mucilaginibacter xinganensis*	WP_094572437.1	WP_094569776.1	WP_317043860.1
Bacteroidota	*Mucilaginibacter yixingensis*	WP_107827472.1	WP_107826445.1	WP_107826477.1
Bacteroidota	*Muricauda lutisoli*	WP_207070618.1	WP_207070520.1	WP_207072086.1
Bacteroidota	*Muriicola jejuensis*	WP_163694011.1	WP_163693910.1	WP_163691096.1
Bacteroidota	*Muriicola marianensis*	WP_188369167.1	WP_188369072.1	WP_188369306.1

Bacteroidota	*Muriicola soli*	WP_129603256.1	WP_129603418.1	WP_129602993.1
Bacteroidota	*Muriicola sp.*	WP_424370009.1	WP_372944865.1	WP_372944702.1
Bacteroidota	*Myroides fluvii*	WP_158960886.1	WP_158963905.1	WP_158962616.1
Bacteroidota	*Myroides guanonis*	WP_090677506.1	WP_090677655.1	WP_090680943.1
Bacteroidota	*Myroides indicus*	WP_133711415.1	WP_133711710.1	WP_133712705.1
Bacteroidota	*Myroides injenensis*	WP_010254937.1	WP_267739598.1	WP_010255812.1
Bacteroidota	*Myroides marinus*	WP_286405314.1	WP_038986771.1	WP_286405152.1
Bacteroidota	*Myroides odoratimimus*	WP_025125853.1	WP_286386351.1	WP_286420923.1
Bacteroidota	*Myroides odoratus*	WP_060873191.1	WP_413511832.1	WP_002987277.1
Bacteroidota	*Myroides oncorhynchi*	WP_229948831.1	WP_229948027.1	WP_229946215.1
Bacteroidota	*Myroides pelagicus*	WP_155035200.1	WP_155036246.1	WP_155035270.1
Bacteroidota	*Myroides phaeus*	WP_090406041.1	WP_090408418.1	WP_090409129.1
Bacteroidota	*Myroides sp. N17-2*	WP_121965180.1	WP_121966521.1	WP_121967414.1
Bacteroidota	*Nafulsella turpanensis*	WP_017731237.1	WP_017729874.1	WP_017731273.1
Bacteroidota	*Namhaeicola litoreus*	WP_377178661.1	WP_377178571.1	WP_377178062.1
Bacteroidota	*Negadavirga shengliensis*	WP_377060634.1	WP_377064796.1	WP_377067651.1
Bacteroidota	*Nemorincola caseinilytica*	WP_345077030.1	WP_345084461.1	WP_345085155.1
Bacteroidota	*Neolewinella agarilytica*	WP_090169585.1	WP_090165148.1	WP_090169212.1
Bacteroidota	*Neolewinella antarctica*	WP_168039119.1	WP_168035921.1	WP_168035849.1
Bacteroidota	*Neolewinella aquimaris*	WP_183495849.1	WP_183493761.1	WP_183494136.1
Bacteroidota	*Neolewinella aurantiaca*	WP_147932863.1	WP_147928689.1	WP_147932599.1
Bacteroidota	*Neolewinella lacunae*	WP_187464858.1	WP_187468026.1	WP_187465779.1
Bacteroidota	*Neolewinella litorea*	WP_136459915.1	WP_136455929.1	WP_136456325.1
Bacteroidota	*Neolewinella marina*	WP_099107199.1	WP_099106183.1	WP_099104521.1
Bacteroidota	*Neolewinella maritima*	WP_238748977.1	WP_238752014.1	WP_238750331.1
Bacteroidota	*Neolewinella persica*	WP_020567182.1	WP_020567298.1	WP_020568668.1
Bacteroidota	*Neolewinella sp.*	WP_420458282.1	WP_420459922.1	WP_420460317.1
Bacteroidota	*Neolewinella xylanilytica*	WP_104418305.1	WP_104421724.1	WP_104420911.1

Bacteroidota	*Neotamlana laminarinivorans*	WP_226540228.1	WP_226540714.1	WP_226544469.1
Bacteroidota	*Neotamlana nanhaiensis*	WP_044626297.1	WP_044624653.1	WP_044627344.1
Bacteroidota	*Neotamlana sargassicola*	WP_226695741.1	WP_226695825.1	WP_226695554.1
Bacteroidota	*Neotamlana sedimentorum*	WP_044632100.1	WP_044632017.1	WP_044633752.1
Bacteroidota	*Neptunitalea chrysea*	WP_281752677.1	WP_281751884.1	WP_281754931.1
Bacteroidota	*Neptunitalea lumnitzerae*	WP_281765818.1	WP_281765697.1	WP_281766268.1
Bacteroidota	*Niabella agricola*	WP_235554510.1	WP_235553365.1	WP_235553023.1
Bacteroidota	*Niabella aurantiaca*	WP_018627122.1	WP_018630035.1	WP_018626360.1
Bacteroidota	*Niabella beijingensis*	WP_223708802.1	WP_223707379.1	WP_223713486.1
Bacteroidota	*Niabella drilacis*	WP_090389250.1	WP_090388761.1	WP_090389126.1
Bacteroidota	*Niabella ginsenosidivorans*	WP_067761319.1	WP_067752730.1	WP_067753521.1
Bacteroidota	*Niabella pedocola*	WP_231007196.1	WP_231004163.1	WP_231003342.1
Bacteroidota	*Niabella soli*	WP_008587685.1	WP_008584562.1	WP_008582146.1
Bacteroidota	*Niabella sp.*	WP_300602989.1	WP_300602285.1	WP_300600842.1
Bacteroidota	*Niabella yanshanensis*	WP_114791872.1	WP_114789308.1	WP_114789230.1
Bacteroidota	*Niastella caeni*	WP_243751588.1	WP_136576828.1	WP_136578394.1
Bacteroidota	*Niastella koreensis*	WP_014223133.1	WP_014220554.1	WP_014218826.1
Bacteroidota	*Niastella populi*	WP_081170820.1	WP_081163880.1	WP_081168632.1
Bacteroidota	*Niastella sp.*	WP_407743925.1	WP_407744206.1	WP_407743428.1
Bacteroidota	*Niastella vici*	WP_081151070.1	WP_081147991.1	WP_081155202.1
Bacteroidota	*Niastella yeongjuensis*	WP_081199676.1	WP_081203469.1	WP_081202772.1
Bacteroidota	*Nibrella saemangeumensis*	WP_345240722.1	WP_345243353.1	WP_345248971.1
Bacteroidota	*Nibrella viscosa*	WP_345269585.1	WP_345263130.1	WP_345268198.1
Bacteroidota	*Nibribacter koreensis*	WP_345169332.1	WP_345162107.1	WP_345165865.1
Bacteroidota	*Nibribacter ruber*	WP_160690431.1	WP_160694538.1	WP_160692977.1
Bacteroidota	*Nitritalea halalkaliphila*	WP_009055316.1	WP_009057114.1	WP_009057364.1
Bacteroidota	*Nonlabens agnitus*	WP_105982533.1	WP_105981802.1	WP_105981856.1
Bacteroidota	*Nonlabens antarcticus*	WP_194850349.1	WP_194852000.1	WP_194850045.1

Bacteroidota	*Nonlabens arenilitoris*	WP_105071680.1	WP_245910827.1	WP_105072145.1
Bacteroidota	*Nonlabens dokdonensis*	WP_015363711.1	WP_303685940.1	WP_304017239.1
Bacteroidota	*Nonlabens marinus*	WP_041497035.1	WP_041494828.1	WP_041497313.1
Bacteroidota	*Nonlabens ponticola*	WP_126448044.1	WP_126448506.1	WP_126448453.1
Bacteroidota	*Nonlabens sp.*	WP_415181852.1	WP_347196734.1	WP_347197301.1
Bacteroidota	*Nonlabens spongiae*	WP_085766589.1	WP_085765672.1	WP_085766476.1
Bacteroidota	*Nonlabens tegetincola*	WP_085774733.1	WP_042278472.1	WP_042278067.1
Bacteroidota	*Nonlabens ulvanivorans*	WP_348021067.1	WP_317291836.1	WP_036584679.1
Bacteroidota	*Nonlabens xiamenensis*	WP_124981425.1	WP_124979846.1	WP_240642324.1
Bacteroidota	*Nubsella zeaxanthinifaciens*	WP_113637362.1	WP_427529302.1	WP_113637772.1
Bacteroidota	*Oceanihabitans sediminis*	WP_113966153.1	WP_072351629.1	WP_113966430.1
Bacteroidota	*Oceanihabitans sp. 2_MG-2023*	WP_303425751.1	WP_303424129.1	WP_303424880.1
Bacteroidota	*Ochrovirga pacifica*	WP_010136703.1	WP_010135397.1	WP_010134725.1
Bacteroidota	*Ohtaekwangia koreensis*	WP_079687037.1	WP_079688647.1	WP_079688007.1
Bacteroidota	*Ohtaekwangia kribbensis*	WP_377580141.1	WP_377585692.1	WP_377579531.1
Bacteroidota	*Ohtaekwangia sp.*	WP_331979302.1	WP_331959849.1	WP_331964706.1
Bacteroidota	*Olivibacter domesticus*	WP_093328473.1	WP_093321673.1	WP_317040863.1
Bacteroidota	*Olivibacter ginsenosidimutans*	WP_345230593.1	WP_345234589.1	WP_345232325.1
Bacteroidota	*Olivibacter sitiensis*	WP_028297179.1	WP_028295449.1	WP_028296240.1
Bacteroidota	*Olivibacter sp. XZL3*	WP_134089489.1	WP_134089330.1	WP_134088390.1
Bacteroidota	*Olleya aquimaris*	WP_111659383.1	WP_111659072.1	WP_111660549.1
Bacteroidota	*Olleya marilimosa*	WP_028282215.1	WP_028282949.1	WP_191101322.1
Bacteroidota	*Olleya namhaensis*	WP_281848004.1	WP_272022623.1	WP_272023392.1
Bacteroidota	*Olleya sp. YS*	WP_279301442.1	WP_279301861.1	WP_279301014.1
Bacteroidota	*Ornithobacterium rhinotracheale*	WP_153828604.1	WP_247117320.1	WP_413371110.1
Bacteroidota	*Owenweeksia hongkongensis*	WP_417609137.1	WP_417591921.1	WP_417589851.1
Bacteroidota	*Paenimyroides aestuarii*	WP_257498498.1	WP_257498436.1	WP_257498655.1
Bacteroidota	*Paenimyroides baculatum*	WP_150014147.1	WP_150014239.1	WP_150011125.1

Bacteroidota	*Paenimyroides ceti*	WP_290362695.1	WP_290363368.1	WP_290363635.1
Bacteroidota	*Paenimyroides marinum*	WP_091095377.1	WP_091095373.1	WP_091095038.1
Bacteroidota	*Paenimyroides tangerinum*	WP_125018708.1	WP_125017044.1	WP_125017160.1
Bacteroidota	*Paenimyroides ummariense*	WP_091522971.1	WP_091526027.1	WP_091521721.1
Bacteroidota	*Paenimyroides viscosum*	WP_124899808.1	WP_124898830.1	WP_124898761.1
Bacteroidota	*Panacibacter ginsenosidivorans*	WP_147189023.1	WP_147192662.1	WP_147188223.1
Bacteroidota	*Panacibacter microcysteis*	WP_196990551.1	WP_196991319.1	WP_196991932.1
Bacteroidota	*Parachryseolinea silvisoli*	WP_267298128.1	WP_267296791.1	WP_267293654.1
Bacteroidota	*Paracnuella aquatica*	WP_114236417.1	WP_241558418.1	WP_114236187.1
Bacteroidota	*Paracrocinitomix mangrovi*	WP_221834572.1	WP_221834850.1	WP_221836018.1
Bacteroidota	*Paradesertivirga mongoliensis*	WP_255897988.1	WP_255902703.1	WP_255902177.1
Bacteroidota	*Parafilimonas sp.*	WP_415161251.1	WP_415157779.1	WP_415157992.1
Bacteroidota	*Parafilimonas terrae*	WP_090655776.1	WP_245751513.1	WP_090658732.1
Bacteroidota	*Paraflavisolibacter caeni*	WP_279295855.1	WP_279299632.1	WP_279297706.1
Bacteroidota	*Paraflavisolibacter sp. H34*	WP_336722650.1	WP_336725373.1	WP_336725333.1
Bacteroidota	*Paraflavitalea devenefica*	WP_167287404.1	WP_167290942.1	WP_167291895.1
Bacteroidota	*Paraflavitalea pollutisoli*	WP_276480049.1	WP_276486104.1	WP_276485702.1
Bacteroidota	*Paraflavitalea soli*	WP_119051981.1	WP_119050955.1	WP_119050606.1
Bacteroidota	*Paraflavitalea sp. CAU 1676*	WP_275834834.1	WP_275832317.1	WP_275834035.1
Bacteroidota	*Parapedobacter composti*	WP_090970143.1	WP_090972664.1	WP_090971754.1
Bacteroidota	*Parapedobacter defluvii*	WP_353197832.1	WP_188753847.1	WP_353195674.1
Bacteroidota	*Parapedobacter deserti*	WP_379023536.1	WP_379021282.1	WP_379020811.1
Bacteroidota	*Parapedobacter indicus*	WP_090627862.1	WP_090624180.1	WP_090626682.1
Bacteroidota	*Parapedobacter koreensis*	WP_090608055.1	WP_090608876.1	WP_090609168.1
Bacteroidota	*Parapedobacter lycopersici*	WP_353181428.1	WP_353181923.1	WP_257658627.1
Bacteroidota	*Parapedobacter pyrenivorans*	WP_188504305.1	WP_353127354.1	WP_188507784.1
Bacteroidota	*Parapedobacter soli*	WP_262246792.1	WP_262248233.1	WP_262245092.1
Bacteroidota	*Parapedobacter sp.*	WP_325635624.1	WP_325636015.1	WP_325637971.1

Bacteroidota	*Parapedobacter tibetensis*	WP_257666263.1	WP_257667877.1	WP_257667435.1
Bacteroidota	*Pararcticibacter amylolyticus*	WP_109416979.1	WP_109414587.1	WP_109416868.1
Bacteroidota	*Pararhodonellum marinum*	WP_194774176.1	WP_194775591.1	WP_194774932.1
Bacteroidota	*Parasediminibacterium paludis*	WP_379013407.1	WP_379014846.1	WP_379012157.1
Bacteroidota	*Parasediminibacterium sp. JCM 36343*	WP_410844105.1	WP_410844722.1	WP_410847215.1
Bacteroidota	*Parasegetibacter sp. NRK P23*	WP_251761400.1	WP_251758786.1	WP_251759009.1
Bacteroidota	*Pareuzebyella sediminis*	WP_149277078.1	WP_149275542.1	WP_149277551.1
Bacteroidota	*Parvicella tangerina*	WP_258541382.1	WP_258543270.1	WP_258541479.1
Bacteroidota	*Patiriisocius hiemis*	WP_311331756.1	WP_311331402.1	WP_311331573.1
Bacteroidota	*Patiriisocius marinistellae*	WP_151894385.1	WP_151894039.1	WP_151894628.1
Bacteroidota	*Patiriisocius marinus*	WP_151673679.1	WP_151673105.1	WP_151673038.1
Bacteroidota	*Patiriisocius sp. Uisw_047*	WP_415371185.1	WP_415370858.1	WP_415370868.1
Bacteroidota	*Paucihalobacter ruber*	WP_140990425.1	WP_140990909.1	WP_140990640.1
Bacteroidota	*Paucihalobacter sp.*	WP_334112096.1	WP_334113206.1	WP_334111572.1
Bacteroidota	*Pedobacter africanus*	WP_084237760.1	WP_084240834.1	WP_310143232.1
Bacteroidota	*Pedobacter agri*	WP_316805750.1	WP_029204146.1	WP_307292130.1
Bacteroidota	*Pedobacter albus*	WP_330109376.1	WP_330105924.1	WP_330107668.1
Bacteroidota	*Pedobacter alluvionis*	WP_121287746.1	WP_121287178.1	WP_121283637.1
Bacteroidota	*Pedobacter alpinus*	WP_379048161.1	WP_379040262.1	WP_379047338.1
Bacteroidota	*Pedobacter antarcticus*	WP_219224099.1	WP_219226882.1	WP_074591307.1
Bacteroidota	*Pedobacter aquae*	WP_149074866.1	WP_149073748.1	WP_149074685.1
Bacteroidota	*Pedobacter aquatilis*	WP_290241275.1	WP_316830188.1	WP_316734565.1
Bacteroidota	*Pedobacter arcticus*	WP_017256750.1	WP_017259123.1	WP_040539760.1
Bacteroidota	*Pedobacter borealis*	WP_029280013.1	WP_029278913.1	WP_029276083.1
Bacteroidota	*Pedobacter boryungensis*	WP_173272792.1	WP_173273727.1	WP_173273906.1
Bacteroidota	*Pedobacter caeni*	WP_073232208.1	WP_073234076.1	WP_073229707.1
Bacteroidota	*Pedobacter changchengzhani*	WP_133263516.1	WP_133262326.1	WP_133261648.1
Bacteroidota	*Pedobacter chinensis*	WP_115402615.1	WP_115404077.1	WP_115404238.1

Bacteroidota	*Pedobacter chitinilyticus*	WP_113648051.1	WP_113645262.1	WP_113647052.1
Bacteroidota	*Pedobacter cryoconitis*	WP_183881087.1	WP_068402913.1	WP_068406289.1
Bacteroidota	*Pedobacter cryophilus*	WP_136825249.1	WP_136826977.1	WP_317130548.1
Bacteroidota	*Pedobacter cryotolerans*	WP_136874354.1	WP_136876996.1	WP_136878258.1
Bacteroidota	*Pedobacter deserti*	WP_256001905.1	WP_256007584.1	WP_256007120.1
Bacteroidota	*Pedobacter duraquae*	WP_133556412.1	WP_133553364.1	WP_133555384.1
Bacteroidota	*Pedobacter endophyticus*	WP_196100950.1	WP_196099093.1	WP_196100596.1
Bacteroidota	*Pedobacter faecalis*	WP_285010636.1	WP_285011385.1	WP_285010782.1
Bacteroidota	*Pedobacter fastidiosus*	WP_187069658.1	WP_187071857.1	WP_187070164.1
Bacteroidota	*Pedobacter flavus*	WP_330144997.1	WP_330145864.1	WP_330145931.1
Bacteroidota	*Pedobacter foliorum*	WP_173090079.1	WP_173093305.1	WP_173091865.1
Bacteroidota	*Pedobacter frigidisoli*	WP_131560922.1	WP_131561206.1	WP_316800050.1
Bacteroidota	*Pedobacter frigiditerrae*	WP_316768949.1	WP_131554479.1	WP_316772800.1
Bacteroidota	*Pedobacter frigoris*	WP_136835606.1	WP_136837513.1	WP_316791726.1
Bacteroidota	*Pedobacter gandavensis*	WP_316842888.1	WP_182960205.1	WP_316840536.1
Bacteroidota	*Pedobacter ghigonis*	WP_175636134.1	WP_175633854.1	WP_175636440.1
Bacteroidota	*Pedobacter ginsengisoli*	WP_099440654.1	WP_285056991.1	WP_285054377.1
Bacteroidota	*Pedobacter glucosidilyticus*	WP_026905129.1	WP_304064693.1	WP_026902661.1
Bacteroidota	*Pedobacter hartonius*	WP_090555335.1	WP_090555089.1	WP_090556570.1
Bacteroidota	*Pedobacter helvus*	WP_138729967.1	WP_138730109.1	WP_138728912.1
Bacteroidota	*Pedobacter heparinus*	WP_012780673.1	WP_012781370.1	WP_316812515.1
Bacteroidota	*Pedobacter hiemivivus*	WP_131610061.1	WP_136881953.1	WP_131607297.1
Bacteroidota	*Pedobacter indicus*	WP_118193771.1	WP_118196262.1	WP_118193992.1
Bacteroidota	*Pedobacter insulae*	WP_090996882.1	WP_090992110.1	WP_090996648.1
Bacteroidota	*Pedobacter jamesrossensis*	WP_378962165.1	WP_378958657.1	WP_378962518.1
Bacteroidota	*Pedobacter jejuensis*	WP_123204999.1	WP_123205102.1	WP_123206726.1
Bacteroidota	*Pedobacter jeongneungensis*	WP_344853497.1	WP_344850380.1	WP_344852960.1
Bacteroidota	*Pedobacter kyungheensis*	WP_039479543.1	WP_039479903.1	WP_039479944.1

Bacteroidota	*Pedobacter lithocola*	WP_378987507.1	WP_378981496.1	WP_378984310.1
Bacteroidota	*Pedobacter lusitanus*	WP_041883719.1	WP_041885191.1	WP_041887252.1
Bacteroidota	*Pedobacter mendelii*	WP_188411375.1	WP_188412809.1	WP_188415496.1
Bacteroidota	*Pedobacter metabolipauper*	WP_133577613.1	WP_133577989.1	WP_133577171.1
Bacteroidota	*Pedobacter miscanthi*	WP_316824526.1	WP_113952201.1	WP_316827092.1
Bacteroidota	*Pedobacter montanisoli*	WP_243360292.1	WP_243363041.1	WP_243360692.1
Bacteroidota	*Pedobacter mucosus*	WP_238414581.1	WP_238414089.1	WP_238414324.1
Bacteroidota	*Pedobacter namyangjuensis*	WP_113654293.1	WP_113653838.1	WP_113654129.1
Bacteroidota	*Pedobacter nanyangensis*	WP_113662147.1	WP_113662675.1	WP_113661786.1
Bacteroidota	*Pedobacter nototheniae*	WP_316801765.1	WP_131538947.1	WP_131535627.1
Bacteroidota	*Pedobacter nutrimenti*	WP_110833957.1	WP_110834448.1	WP_110831824.1
Bacteroidota	*Pedobacter nyackensis*	WP_316816358.1	WP_084286656.1	WP_084289426.1
Bacteroidota	*Pedobacter paludis*	WP_109930788.1	WP_109932520.1	WP_109931200.1
Bacteroidota	*Pedobacter panaciterrae*	WP_172661574.1	WP_172659420.1	WP_337717474.1
Bacteroidota	*Pedobacter petrophilus*	WP_154281502.1	WP_154282467.1	WP_154278704.1
Bacteroidota	*Pedobacter planticolens*	WP_182922361.1	WP_182922156.1	WP_182920612.1
Bacteroidota	*Pedobacter polaris*	WP_136838023.1	WP_136844270.1	WP_136841447.1
Bacteroidota	*Pedobacter psychrodurus*	WP_131533002.1	WP_131534490.1	WP_131530597.1
Bacteroidota	*Pedobacter psychrophilus*	WP_068822602.1	WP_068823604.1	WP_068822891.1
Bacteroidota	*Pedobacter psychroterrae*	WP_131594357.1	WP_131597463.1	WP_131597727.1
Bacteroidota	*Pedobacter psychrotolerans*	WP_132530194.1	WP_132536433.1	WP_132529721.1
Bacteroidota	*Pedobacter punctiformis*	WP_269428406.1	WP_269427832.1	WP_269428613.1
Bacteroidota	*Pedobacter puniceum*	WP_154288091.1	WP_154286091.1	WP_154288349.1
Bacteroidota	*Pedobacter quisquiliarum*	WP_188626080.1	WP_188627188.1	WP_188624811.1
Bacteroidota	*Pedobacter rhizosphaerae*	WP_090884952.1	WP_090881040.1	WP_090883636.1
Bacteroidota	*Pedobacter rhodius*	WP_269417121.1	WP_269413907.1	WP_269416549.1
Bacteroidota	*Pedobacter roseus*	WP_187592982.1	WP_187591282.1	WP_187591487.1
Bacteroidota	*Pedobacter sandarakinus*	WP_265854285.1	WP_265855752.1	WP_265854120.1

Bacteroidota	*Pedobacter schmidteae*	WP_121269434.1	WP_121273731.1	WP_121271022.1
Bacteroidota	*Pedobacter segetis*	WP_200587972.1	WP_200585255.1	WP_200586207.1
Bacteroidota	*Pedobacter soli*	WP_090772814.1	WP_090772601.1	WP_090766432.1
Bacteroidota	*Pedobacter sp.*	WP_421938346.1	WP_343522411.1	WP_353903901.1
Bacteroidota	*Pedobacter steynii*	WP_069379562.1	WP_074606995.1	WP_069378909.1
Bacteroidota	*Pedobacter suwonensis*	WP_145857173.1	WP_293741410.1	WP_145860125.1
Bacteroidota	*Pedobacter terrae*	WP_335317504.1	WP_335320483.1	WP_335318463.1
Bacteroidota	*Pedobacter ureilyticus*	WP_138722568.1	WP_138723946.1	WP_138721851.1
Bacteroidota	*Pedobacter vanadiisoli*	WP_379081851.1	WP_379080222.1	WP_379081366.1
Bacteroidota	*Pedobacter westerhofensis*	WP_142528417.1	WP_142529467.1	WP_142530983.1
Bacteroidota	*Pedobacter xixiisoli*	WP_097132640.1	WP_097127343.1	WP_097132993.1
Bacteroidota	*Pedobacter yonginense*	WP_109925110.1	WP_109927344.1	WP_109926179.1
Bacteroidota	*Pedobacter yulinensis*	WP_107214288.1	WP_107216326.1	WP_107216633.1
Bacteroidota	*Pedobacter zeae*	WP_183765082.1	WP_183759401.1	WP_183768407.1
Bacteroidota	*Pelagihabitans pacificus*	WP_152575596.1	WP_152574733.1	WP_152575827.1
Bacteroidota	*Pelobium manganitolerans*	WP_120181251.1	WP_120181798.1	WP_120181363.1
Bacteroidota	*Penaeicola halotolerans*	WP_226390126.1	WP_226389474.1	WP_226390042.1
Bacteroidota	*Persicitalea jodogahamensis*	WP_189563363.1	WP_189566008.1	WP_189562809.1
Bacteroidota	*Persicitalea sp.*	WP_373514763.1	WP_373514327.1	WP_373511538.1
Bacteroidota	*Persicobacter diffluens*	WP_338237395.1	WP_338236170.1	WP_338235858.1
Bacteroidota	*Persicobacter psychrovividus*	WP_332922396.1	WP_338397812.1	WP_338397565.1
Bacteroidota	*Persicobacter sp. CCB-QB2*	WP_053406831.1	WP_053404325.1	WP_053405934.1
Bacteroidota	*Phaeocystidibacter luteus*	WP_151668120.1	WP_151668392.1	WP_170266297.1
Bacteroidota	*Phaeocystidibacter marisrubri*	WP_151691796.1	WP_151692856.1	WP_151692644.1
Bacteroidota	*Phaeodactylibacter luteus*	WP_147168864.1	WP_147166612.1	WP_147167063.1
Bacteroidota	*Phaeodactylibacter sp.*	WP_350176227.1	WP_350177840.1	WP_293564349.1
Bacteroidota	*Phaeodactylibacter xiamenensis*	WP_044220771.1	WP_345894640.1	WP_421948296.1
Bacteroidota	Phnomibacter ginsenosidimutans	WP_157479401.1	WP_157478826.1	WP_157478517.1

Bacteroidota	*Phnomibacter sp.*	WP_333801136.1	WP_333798932.1	WP_333799269.1
Bacteroidota	*Pinibacter aurantiacus*	WP_217789871.1	WP_217794396.1	WP_217791097.1
Bacteroidota	*Pinibacter soli*	WP_282334310.1	WP_282334518.1	WP_282335433.1
Bacteroidota	*Planktosalinus lacus*	WP_188441418.1	WP_188443026.1	WP_188439440.1
Bacteroidota	*Planobacterium oryzisoli*	WP_194739118.1	WP_194739626.1	WP_194738760.1
Bacteroidota	*Pleomorphovibrio marinus*	WP_114751276.1	WP_114750181.1	WP_114747671.1
Bacteroidota	*Polaribacter aestuariivivens*	WP_138534867.1	WP_138535111.1	WP_138537235.1
Bacteroidota	*Polaribacter aquimarinus*	WP_109404621.1	WP_109405612.1	WP_109404950.1
Bacteroidota	*Polaribacter atrinae*	WP_068452971.1	WP_068451824.1	WP_341222082.1
Bacteroidota	*Polaribacter batillariae*	WP_207972030.1	WP_207972378.1	WP_207971661.1
Bacteroidota	*Polaribacter butkevichii*	WP_105048550.1	WP_105048114.1	WP_105048036.1
Bacteroidota	*Polaribacter cellanae*	WP_208079180.1	WP_208079597.1	WP_208079626.1
Bacteroidota	*Polaribacter dokdonensis*	WP_053974708.1	WP_053974658.1	WP_053975070.1
Bacteroidota	*Polaribacter filamentus*	WP_104809256.1	WP_104808805.1	WP_104808646.1
Bacteroidota	*Polaribacter gangjinensis*	WP_105045548.1	WP_105047271.1	WP_105047223.1
Bacteroidota	*Polaribacter glomeratus*	WP_105020554.1	WP_105020177.1	WP_105020891.1
Bacteroidota	*Polaribacter haliotis*	WP_088353541.1	WP_088353291.1	WP_088352880.1
Bacteroidota	*Polaribacter huanghezhanensis*	WP_301400243.1	WP_301400130.1	WP_301400796.1
Bacteroidota	*Polaribacter irgensii*	WP_026288371.1	WP_018942509.1	WP_004570425.1
Bacteroidota	*Polaribacter litorisediminis*	WP_223446044.1	WP_223446128.1	WP_223439388.1
Bacteroidota	*Polaribacter marinivivus*	WP_377410213.1	WP_377410502.1	WP_377409873.1
Bacteroidota	*Polaribacter marinus*	WP_242177238.1	WP_242177468.1	WP_242176929.1
Bacteroidota	*Polaribacter pacificus*	WP_188598849.1	WP_188598986.1	WP_188599336.1
Bacteroidota	*Polaribacter pectinis*	WP_187483341.1	WP_187483380.1	WP_187482972.1
Bacteroidota	*Polaribacter ponticola*	WP_265724554.1	WP_265724573.1	WP_274270124.1
Bacteroidota	*Polaribacter porphyrae*	WP_105014864.1	WP_105014974.1	WP_105015495.1
Bacteroidota	*Polaribacter reichenbachii*	WP_068360224.1	WP_068360040.1	WP_068364870.1
Bacteroidota	*Polaribacter sejongensis*	WP_208889400.1	WP_208888940.1	WP_208888764.1

Bacteroidota	*Polaribacter septentrionalilitoris*	WP_159947352.1	WP_159947839.1	WP_159948279.1
Bacteroidota	*Polaribacter sp.*	WP_422162747.1	WP_422162769.1	WP_298948141.1
Bacteroidota	*Polaribacter staleyi*	WP_343329764.1	WP_343329481.1	WP_343329414.1
Bacteroidota	*Polaribacter tangerinus*	WP_088322902.1	WP_088322791.1	WP_088322699.1
Bacteroidota	*Polaribacter vadi*	WP_065318254.1	WP_339883837.1	WP_339884802.1
Bacteroidota	*Pollutibacter soli*	WP_336516241.1	WP_336514272.1	WP_336514248.1
Bacteroidota	*Polluticaenibacter yanchengensis*	WP_407031566.1	WP_407030962.1	WP_407032955.1
Bacteroidota	*Polluticoccus soli*	WP_276134619.1	WP_276134772.1	WP_276132203.1
Bacteroidota	*Pontibacter actiniarum*	WP_025607855.1	WP_025605102.1	WP_025608381.1
Bacteroidota	*Pontibacter akesuensis*	WP_068837038.1	WP_068839774.1	WP_082815155.1
Bacteroidota	*Pontibacter amylolyticus*	WP_188502506.1	WP_188499622.1	WP_188500582.1
Bacteroidota	*Pontibacter anaerobius*	WP_266051738.1	WP_266054022.1	WP_266054192.1
Bacteroidota	*Pontibacter aquaedesilientis*	WP_191182936.1	WP_191182164.1	WP_224744371.1
Bacteroidota	*Pontibacter arcticus*	WP_112306243.1	WP_112305500.1	WP_239020921.1
Bacteroidota	*Pontibacter aydingkolensis*	WP_219876295.1	WP_219878462.1	WP_246596896.1
Bacteroidota	*Pontibacter beigongshangensis*	WP_187260967.1	WP_187260582.1	WP_187264052.1
Bacteroidota	*Pontibacter burrus*	WP_163915162.1	WP_163914727.1	WP_163910788.1
Bacteroidota	*Pontibacter cellulosilyticus*	WP_187067611.1	WP_187065817.1	WP_230407259.1
Bacteroidota	*Pontibacter chinhatensis*	WP_092099925.1	WP_092098427.1	WP_092101233.1
Bacteroidota	*Pontibacter chitinilyticus*	WP_347159781.1	WP_347157039.1	WP_347157394.1
Bacteroidota	*Pontibacter diazotrophicus*	WP_115563627.1	WP_115565944.1	WP_246000815.1
Bacteroidota	*Pontibacter fetidus*	WP_162345166.1	WP_162344390.1	WP_162346564.1
Bacteroidota	*Pontibacter flavimaris*	WP_170866119.1	WP_073851109.1	WP_073851885.1
Bacteroidota	*Pontibacter harenae*	WP_229972779.1	WP_229969948.1	WP_229968909.1
Bacteroidota	*Pontibacter indicus*	WP_170871914.1	WP_076666330.1	WP_244554682.1
Bacteroidota	*Pontibacter kalidii*	WP_266202819.1	WP_266205387.1	WP_266203411.1
Bacteroidota	*Pontibacter korlensis*	WP_046308896.1	WP_046312400.1	WP_084694729.1
Bacteroidota	*Pontibacter liquoris*	WP_242916368.1	WP_242918685.1	WP_242921285.1

Bacteroidota	*Pontibacter litorisediminis*	WP_276499133.1	WP_276495720.1	WP_276498631.1
Bacteroidota	*Pontibacter locisalis*	WP_377507272.1	WP_377512209.1	WP_377503573.1
Bacteroidota	*Pontibacter mangrovi*	WP_140621972.1	WP_140618665.1	WP_239022830.1
Bacteroidota	*Pontibacter mucosus*	WP_108212430.1	WP_108213444.1	WP_108210166.1
Bacteroidota	*Pontibacter oryzae*	WP_119430834.1	WP_119431269.1	WP_119432100.1
Bacteroidota	*Pontibacter pamirensis*	WP_162053720.1	WP_162052484.1	WP_237144869.1
Bacteroidota	*Pontibacter populi*	WP_350410730.1	WP_350412632.1	WP_350411634.1
Bacteroidota	*Pontibacter pudoricolor*	WP_162428816.1	WP_162426266.1	WP_162427917.1
Bacteroidota	*Pontibacter qinzhouensis*	WP_147922611.1	WP_147923662.1	WP_255474196.1
Bacteroidota	*Pontibacter ramchanderi*	WP_101446093.1	WP_101442876.1	WP_180336380.1
Bacteroidota	*Pontibacter roseus*	WP_211214358.1	WP_018478075.1	WP_018476588.1
Bacteroidota	*Pontibacter ruber*	WP_250431823.1	WP_250429605.1	WP_250428291.1
Bacteroidota	*Pontibacter rugosus*	WP_377526565.1	WP_377529224.1	WP_377526710.1
Bacteroidota	*Pontibacter russatus*	WP_161890271.1	WP_161887828.1	WP_237586605.1
Bacteroidota	*Pontibacter saemangeumensis*	WP_345161860.1	WP_345157442.1	WP_345159741.1
Bacteroidota	*Pontibacter silvestris*	WP_229962237.1	WP_229958046.1	WP_229960458.1
Bacteroidota	*Pontibacter sp. HSC-36F09*	WP_253402509.1	WP_253405866.1	WP_253401128.1
Bacteroidota	*Pontibacter toksunensis*	WP_377487128.1	WP_377479545.1	WP_377485187.1
Bacteroidota	*Pontibacter ummariensis*	WP_089319534.1	WP_089317724.1	WP_089319831.1
Bacteroidota	*Pontibacter virosus*	WP_207774867.1	WP_116543526.1	WP_116541785.1
Bacteroidota	*Pontibacter vulgaris*	WP_242927759.1	WP_242928228.1	WP_242926450.1
Bacteroidota	*Pontimicrobium aquaticum*	WP_136844924.1	WP_136842894.1	WP_136842336.1
Bacteroidota	*Pontimicrobium sp. IMCC45349*	WP_412984909.1	WP_412985655.1	WP_412984315.1
Bacteroidota	*Porifericola rhodea*	WP_302241969.1	WP_302238587.1	WP_302241977.1
Bacteroidota	*Poritiphilus flavus*	WP_161435387.1	WP_161436029.1	WP_161435034.1
Bacteroidota	*Portibacter lacus*	WP_235295426.1	WP_235292611.1	WP_235293306.1
Bacteroidota	*Portibacter marinus*	WP_235298559.1	WP_235296124.1	WP_235298989.1
Bacteroidota	*Postechiella marina*	WP_344786197.1	WP_344786381.1	WP_344789558.1

Bacteroidota	*Pricia antarctica*	WP_091870366.1	WP_091870156.1	WP_091868748.1
Bacteroidota	*Pricia mediterranea*	WP_314016435.1	WP_314016377.1	WP_314012617.1
Bacteroidota	*Pricia sp.*	WP_373518980.1	WP_373518823.1	WP_373517750.1
Bacteroidota	*Profundicola chukchiensis*	WP_304420887.1	WP_304420718.1	WP_304416377.1
Bacteroidota	*Pseudalgibacter alginicilyticus*	WP_054724859.1	WP_054724689.1	WP_054725144.1
Bacteroidota	*Pseudarcicella hirudinis*	WP_092018740.1	WP_092016199.1	WP_092018609.1
Bacteroidota	*Pseudobacter ginsenosidimutans*	WP_130542873.1	WP_130543804.1	WP_130540330.1
Bacteroidota	*Pseudochryseolinea flava*	WP_112748661.1	WP_112749846.1	WP_112745532.1
Bacteroidota	*Pseudocnuella soli*	WP_132050710.1	WP_240040623.1	WP_132055477.1
Bacteroidota	*Pseudoflavitalea rhizosphaerae*	WP_127128830.1	WP_127132125.1	WP_127125031.1
Bacteroidota	*Pseudoflavitalea sp. G-6-1-2*	WP_169423241.1	WP_169427293.1	WP_169427852.1
Bacteroidota	*Pseudofulvibacter geojedonensis*	WP_377715660.1	WP_377712568.1	WP_377713360.1
Bacteroidota	*Pseudopedobacter beijingensis*	WP_379661639.1	WP_379663639.1	WP_379660747.1
Bacteroidota	*Pseudopedobacter saltans*	WP_013634471.1	WP_013633057.1	WP_013631349.1
Bacteroidota	*Pseudopedobacter sp.*	WP_353133078.1	WP_353136078.1	WP_353135823.1
Bacteroidota	*Pseudotamlana agarivorans*	WP_067147999.1	WP_216068537.1	WP_216068325.1
Bacteroidota	*Pseudotamlana carrageenivorans*	WP_102996535.1	WP_102996350.1	WP_102996863.1
Bacteroidota	*Pseudotamlana haliotis*	WP_150935676.1	WP_150937802.1	WP_150940490.1
Bacteroidota	*Pseudotenacibaculum haliotis*	WP_379666268.1	WP_379666551.1	WP_379664548.1
Bacteroidota	*Pseudotenacibaculum sp. MALMAid0570*	WP_349553908.1	WP_349553590.1	WP_349554108.1
Bacteroidota	*Pseudozobellia sp. WGM2*	WP_209399679.1	WP_209403114.1	WP_209405363.1
Bacteroidota	*Pseudozobellia thermophila*	WP_072994189.1	WP_072994881.1	WP_072995245.1
Bacteroidota	*Psychroflexus aestuariivivens*	WP_127844959.1	WP_127845023.1	WP_127846452.1
Bacteroidota	*Psychroflexus aurantiacus*	WP_164005076.1	WP_164004848.1	WP_164004585.1
Bacteroidota	*Psychroflexus curvus*	WP_224486199.1	WP_224509615.1	WP_224487413.1
Bacteroidota	*Psychroflexus gondwanensis*	WP_003438426.1	WP_003442182.1	WP_003440994.1
Bacteroidota	*Psychroflexus halocasei*	WP_093239311.1	WP_093241794.1	WP_093244459.1
Bacteroidota	*Psychroflexus lacisalsi*	WP_224454393.1	WP_224454992.1	WP_224454775.1

Bacteroidota	*Psychroflexus longus*	WP_224461640.1	WP_224461157.1	WP_224461978.1
Bacteroidota	*Psychroflexus maritimus*	WP_166400947.1	WP_166400890.1	WP_166399151.1
Bacteroidota	*Psychroflexus montanilacus*	WP_224462274.1	WP_224465188.1	WP_224464209.1
Bacteroidota	*Psychroflexus planctonicus*	WP_188457486.1	WP_188458122.1	WP_188459313.1
Bacteroidota	*Psychroflexus salarius*	WP_073191291.1	WP_073190977.1	WP_073193286.1
Bacteroidota	*Psychroflexus salinarum*	WP_379657626.1	WP_379658489.1	WP_379658701.1
Bacteroidota	*Psychroflexus salis*	WP_188405766.1	WP_188405842.1	WP_188406275.1
Bacteroidota	*Psychroflexus sediminis*	WP_093369230.1	WP_093367813.1	WP_093367458.1
Bacteroidota	*Psychroflexus sp. MES1-P1E*	WP_102089321.1	WP_102087993.1	WP_102089097.1
Bacteroidota	*Psychroflexus torquis*	WP_015025785.1	WP_015025542.1	WP_015024936.1
Bacteroidota	*Psychroflexus tropicus*	WP_019038808.1	WP_019039259.1	WP_019038943.1
Bacteroidota	*Psychroserpens algicola*	WP_204345061.1	WP_204345741.1	WP_204346159.1
Bacteroidota	*Psychroserpens burtonensis*	WP_028870830.1	WP_028872699.1	WP_028872803.1
Bacteroidota	*Psychroserpens damuponensis*	WP_040279120.1	WP_040280953.1	WP_040281111.1
Bacteroidota	*Psychroserpens jangbogonensis*	WP_033959147.1	WP_033960345.1	WP_033956243.1
Bacteroidota	*Psychroserpens luteolus*	WP_230935135.1	WP_230935982.1	WP_230936893.1
Bacteroidota	*Psychroserpens luteus*	WP_194507318.1	WP_194507631.1	WP_194508324.1
Bacteroidota	*Psychroserpens mesophilus*	WP_040251637.1	WP_174435320.1	WP_040250904.1
Bacteroidota	*Psychroserpens ponticola*	WP_249995923.1	WP_249995559.1	WP_249995438.1
Bacteroidota	*Psychroserpens sp.*	WP_425223194.1	WP_405335216.1	WP_424516448.1
Bacteroidota	*Puia dinghuensis*	WP_188937963.1	WP_188929879.1	WP_188927805.1
Bacteroidota	*Puia sp.*	WP_331576494.1	WP_331578006.1	WP_331576466.1
Bacteroidota	*Pukyongia salina*	WP_105217152.1	WP_105216883.1	WP_105214167.1
Bacteroidota	*Pustulibacterium marinum*	WP_093026409.1	WP_093025214.1	WP_093023162.1
Bacteroidota	*Putridiphycobacter roseus*	WP_111062192.1	WP_111062136.1	WP_111061356.1
Bacteroidota	*Raineya orbicola*	WP_101359701.1	WP_101357744.1	WP_165778063.1
Bacteroidota	*Rapidithrix thailandica*	WP_346820230.1	WP_346819169.1	WP_346819304.1
Bacteroidota	*Rasiella rasia*	WP_164678373.1	WP_164678848.1	WP_164679013.1

Bacteroidota	*Ravibacter arvi*	WP_345028311.1	WP_345026905.1	WP_345026675.1
Bacteroidota	*Reichenbachiella agariperforans*	WP_073124535.1	WP_073123459.1	WP_216011389.1
Bacteroidota	*Reichenbachiella agarivorans*	WP_262309824.1	WP_262308525.1	WP_262310351.1
Bacteroidota	*Reichenbachiella carrageenanivorans*	WP_263050107.1	WP_263049587.1	WP_263051374.1
Bacteroidota	*Reichenbachiella faecimaris*	WP_084371036.1	WP_084371183.1	WP_084371882.1
Bacteroidota	*Reichenbachiella sp.*	WP_348179303.1	WP_420580935.1	WP_348183144.1
Bacteroidota	*Reichenbachiella ulvae*	WP_264137898.1	WP_264137298.1	WP_264138500.1
Bacteroidota	*Reichenbachiella versicolor*	WP_109829597.1	WP_109831061.1	WP_109832016.1
Bacteroidota	*Rhabdobacter roseus*	WP_184174142.1	WP_184172287.1	WP_184174737.1
Bacteroidota	*Rhinopithecimicrobium faecis*	WP_353546519.1	WP_353546293.1	WP_353547657.1
Bacteroidota	*Rhizosphaericola mali*	WP_131328136.1	WP_131330415.1	WP_131330649.1
Bacteroidota	*Rhodocytophaga aerolata*	WP_302039324.1	WP_302037742.1	WP_302038683.1
Bacteroidota	*Rhodocytophaga rosea*	WP_162443911.1	WP_162448146.1	WP_232064797.1
Bacteroidota	*Rhodoflexus caldus*	WP_250631486.1	WP_250632666.1	WP_250629984.1
Bacteroidota	*Riemerella anatipestifer*	WP_340305057.1	WP_004917237.1	WP_310492218.1
Bacteroidota	*Riemerella columbina*	WP_302505457.1	WP_302505408.1	WP_018675101.1
Bacteroidota	*Riemerella columbipharyngis*	WP_092736975.1	WP_092737568.1	WP_092736482.1
Bacteroidota	*Robertkochia marina*	WP_224490187.1	WP_224491073.1	WP_136336997.1
Bacteroidota	*Robertkochia sediminum*	WP_203061522.1	WP_203060466.1	WP_203059512.1
Bacteroidota	*Robertkochia solimangrovi*	WP_143955679.1	WP_143955932.1	WP_143956131.1
Bacteroidota	*Robertkochia sp. 3YJGBD-33*	WP_224483128.1	WP_224484353.1	WP_224484607.1
Bacteroidota	*Robiginitalea aestuariiviva*	WP_277707881.1	WP_277707969.1	WP_277707719.1
Bacteroidota	*Robiginitalea aurantiaca*	WP_289725935.1	WP_289725543.1	WP_289725990.1
Bacteroidota	*Robiginitalea marina*	WP_252739687.1	WP_252739769.1	WP_252740229.1
Bacteroidota	*Robiginitalea myxolifaciens*	WP_092982559.1	WP_092982473.1	WP_092982694.1
Bacteroidota	*Robiginitalea sediminis*	WP_088340947.1	WP_088341021.1	WP_088340817.1
Bacteroidota	*Robiginitalea sp.*	WP_424498482.1	WP_424497095.1	WP_424498923.1
Bacteroidota	*Roseivirga echinicomitans*	WP_068416355.1	WP_068413391.1	WP_068414968.1

Bacteroidota	*Roseivirga ehrenbergii*	WP_062588049.1	WP_062591750.1	WP_062594148.1
Bacteroidota	*Roseivirga misakiensis*	WP_069833988.1	WP_069835979.1	WP_069833712.1
Bacteroidota	*Roseivirga pacifica*	WP_252937943.1	WP_422354073.1	WP_268123344.1
Bacteroidota	*Roseivirga seohaensis*	WP_421977005.1	WP_421976220.1	WP_421976594.1
Bacteroidota	*Roseivirga sp.*	WP_421986854.1	WP_273274618.1	WP_323756790.1
Bacteroidota	*Rudanella lutea*	WP_019988432.1	WP_027302744.1	WP_019989311.1
Bacteroidota	*Rudanella paleaurantiibacter*	WP_152125379.1	WP_152125921.1	WP_152123479.1
Bacteroidota	*Rufibacter aurantiacus*	WP_210487332.1	WP_210488992.1	WP_210488416.1
Bacteroidota	*Rufibacter glacialis*	WP_149097938.1	WP_149098143.1	WP_149098844.1
Bacteroidota	*Rufibacter hautae*	WP_149088950.1	WP_149091153.1	WP_149089626.1
Bacteroidota	*Rufibacter immobilis*	WP_123132599.1	WP_377059760.1	WP_123133193.1
Bacteroidota	*Rufibacter latericius*	WP_123125038.1	WP_123125940.1	WP_123126617.1
Bacteroidota	*Rufibacter psychrotolerans*	WP_205502872.1	WP_205500681.1	WP_205500297.1
Bacteroidota	*Rufibacter radiotolerans*	WP_048921188.1	WP_048920482.1	WP_048921794.1
Bacteroidota	*Rufibacter roseolus*	WP_210463819.1	WP_210464973.1	WP_210463158.1
Bacteroidota	*Rufibacter roseus*	WP_066618735.1	WP_066621324.1	WP_066623342.1
Bacteroidota	*Rufibacter sediminis*	WP_186640101.1	WP_186636070.1	WP_186639587.1
Bacteroidota	*Rufibacter sp. XAAS-G3-1*	WP_181305783.1	WP_181306455.1	WP_181305147.1
Bacteroidota	*Rufibacter tibetensis*	WP_062542449.1	WP_062545229.1	WP_062543027.1
Bacteroidota	*Runella aurantiaca*	WP_114461994.1	WP_114463800.1	WP_114460029.1
Bacteroidota	*Runella defluvii*	WP_183970994.1	WP_183975005.1	WP_183975628.1
Bacteroidota	*Runella limosa*	WP_051397804.1	WP_028522048.1	WP_028525003.1
Bacteroidota	*Runella slithyformis*	WP_013928840.1	WP_013925979.1	WP_013926801.1
Bacteroidota	*Runella sp.*	WP_298349780.1	WP_298356035.1	WP_298353356.1
Bacteroidota	*Runella zeae*	WP_028666985.1	WP_273215829.1	WP_028664036.1
Bacteroidota	*Rurimicrobium arvi*	WP_344826516.1	WP_344828551.1	WP_344822986.1
Bacteroidota	*Sabulibacter ruber*	WP_207435692.1	WP_207430476.1	WP_207434099.1
Bacteroidota	*Salegentibacter chungangensis*	WP_380745092.1	WP_380744225.1	WP_380744138.1

Bacteroidota	*Salegentibacter echinorum*	WP_072880367.1	WP_072876739.1	WP_072877766.1
Bacteroidota	*Salegentibacter holothuriorum*	WP_079719038.1	WP_079719586.1	WP_079721408.1
Bacteroidota	*Salegentibacter lacus*	WP_224470366.1	WP_224469924.1	WP_224471196.1
Bacteroidota	*Salegentibacter maritimus*	WP_198637701.1	WP_198638642.1	WP_198639097.1
Bacteroidota	*Salegentibacter mishustinae*	WP_057483160.1	WP_057481422.1	WP_282019038.1
Bacteroidota	*Salegentibacter salarius*	WP_070054325.1	WP_070053585.1	WP_070054088.1
Bacteroidota	*Salegentibacter salegens*	WP_079734098.1	WP_079734565.1	WP_079734433.1
Bacteroidota	*Salegentibacter salinarum*	WP_079712555.1	WP_079713981.1	WP_079712005.1
Bacteroidota	*Salegentibacter sediminis*	WP_081211134.1	WP_081212117.1	WP_081212067.1
Bacteroidota	*Salegentibacter sp. Hel_I_6*	WP_037322480.1	WP_037315191.1	WP_037320299.1
Bacteroidota	*Salegentibacter tibetensis*	WP_224473551.1	WP_224474186.1	WP_224474207.1
Bacteroidota	*Salibacter halophilus*	WP_151167347.1	WP_151166061.1	WP_151165890.1
Bacteroidota	*Salibacter sp.*	WP_310667847.1	WP_310667026.1	WP_310683725.1
Bacteroidota	*Salinimicrobium catena*	WP_093112685.1	WP_093112502.1	WP_093113704.1
Bacteroidota	*Salinimicrobium flavum*	WP_380752956.1	WP_380747343.1	WP_380748127.1
Bacteroidota	*Salinimicrobium gaetbulicola*	WP_380736752.1	WP_380736910.1	WP_380737270.1
Bacteroidota	*Salinimicrobium marinum*	WP_189603621.1	WP_189604486.1	WP_189604708.1
Bacteroidota	*Salinimicrobium oceani*	WP_168138491.1	WP_168136558.1	WP_168136841.1
Bacteroidota	*Salinimicrobium profundisediminis*	WP_266068280.1	WP_266070952.1	WP_266070336.1
Bacteroidota	*Salinimicrobium sediminis*	WP_097056067.1	WP_097056276.1	WP_097057124.1
Bacteroidota	*Salinimicrobium soli*	WP_418335852.1	WP_418335645.1	WP_418334907.1
Bacteroidota	*Salinimicrobium sp. HB62*	WP_324719156.1	WP_324719385.1	WP_324721883.1
Bacteroidota	*Salinimicrobium terrae*	WP_029033011.1	WP_029033552.1	WP_029034990.1
Bacteroidota	*Salinimicrobium tongyeongense*	WP_265163551.1	WP_265163274.1	WP_265164049.1
Bacteroidota	*Salinimicrobium xinjiangense*	WP_029036068.1	WP_029037520.1	WP_029038605.1
Bacteroidota	*Salmonirosea aquatica*	WP_152762569.1	WP_152764206.1	WP_152759519.1
Bacteroidota	*Sandaracinomonas limnophila*	WP_127804355.1	WP_127802513.1	WP_127803171.1
Bacteroidota	*Sanyastnella coralliicola*	WP_306643031.1	WP_306639898.1	WP_306643246.1

Bacteroidota	*Saonia flava*	WP_167960989.1	WP_167960713.1	WP_167961412.1
Bacteroidota	*Saprospira grandis*	WP_270100066.1	WP_015691616.1	WP_014373498.1
Bacteroidota	*Saprospira sp. CCB-QB6*	WP_272620540.1	WP_272620609.1	WP_272620088.1
Bacteroidota	*Schleiferia thermophila*	WP_037355887.1	WP_037358008.1	WP_160171979.1
Bacteroidota	*Sediminibacter sp. Hel_I_10*	WP_026755702.1	WP_026755964.1	WP_026753280.1
Bacteroidota	*Sediminibacterium ginsengisoli*	WP_078831188.1	WP_078832335.1	WP_245825700.1
Bacteroidota	*Sediminibacterium goheungense*	WP_133474678.1	WP_133475808.1	WP_133475323.1
Bacteroidota	*Sediminibacterium roseum*	WP_161817057.1	WP_161818326.1	WP_161817922.1
Bacteroidota	*Sediminibacterium salmoneum*	WP_026764238.1	WP_026763456.1	WP_026763181.1
Bacteroidota	*Sediminibacterium soli*	WP_161835386.1	WP_161834296.1	WP_161834578.1
Bacteroidota	*Sediminibacterium sp.*	WP_322194853.1	WP_322196213.1	WP_295204650.1
Bacteroidota	*Sediminicola arcticus*	WP_354615426.1	WP_354615298.1	WP_354615560.1
Bacteroidota	*Sediminicola luteus*	WP_097443678.1	WP_097442876.1	WP_097440349.1
Bacteroidota	*Sediminicola sp. 1XM1-17*	WP_337300318.1	WP_337300228.1	WP_337299993.1
Bacteroidota	*Sediminitomix flava*	WP_109616372.1	WP_109618873.1	WP_245935572.1
Bacteroidota	*Segetibacter aerophilus*	WP_147203107.1	WP_147204351.1	WP_147202618.1
Bacteroidota	*Segetibacter koreensis*	WP_018617501.1	WP_018610874.1	WP_018612798.1
Bacteroidota	*Segetibacter sp.*	WP_297821751.1	WP_297821841.1	WP_297822188.1
Bacteroidota	*Seonamhaeicola algicola*	WP_147130549.1	WP_147133745.1	WP_147133851.1
Bacteroidota	*Seonamhaeicola aphaedonensis*	WP_116039943.1	WP_116523873.1	WP_116524046.1
Bacteroidota	*Seonamhaeicola marinus*	WP_148541872.1	WP_148544579.1	WP_262713610.1
Bacteroidota	*Seonamhaeicola maritimus*	WP_282135950.1	WP_147769343.1	WP_147769247.1
Bacteroidota	*Seonamhaeicola sediminis*	WP_133356602.1	WP_133357173.1	WP_133354879.1
Bacteroidota	*Seonamhaeicola sp.*	WP_299547549.1	WP_299550404.1	WP_299551035.1
Bacteroidota	*Shiella aurantiaca*	WP_320003240.1	WP_320003921.1	WP_320004771.1
Bacteroidota	*Shivajiella indica*	WP_380799824.1	WP_380800894.1	WP_380799678.1
Bacteroidota	*Siansivirga zeaxanthinifaciens*	WP_044638880.1	WP_044638641.1	WP_044638743.1
Bacteroidota	*Sinomicrobium kalidii*	WP_233030326.1	WP_233028330.1	WP_233028368.1

Bacteroidota	*Sinomicrobium oceani*	WP_072317176.1	WP_072315514.1	WP_326492528.1
Bacteroidota	*Sinomicrobium pectinilyticum*	WP_123214022.1	WP_123218411.1	WP_123214924.1
Bacteroidota	*Sinomicrobium sp. N-1-3-6*	WP_112372356.1	WP_112370791.1	WP_112373290.1
Bacteroidota	*Sinomicrobium weinanense*	WP_187964674.1	WP_187966821.1	WP_187964446.1
Bacteroidota	*Siphonobacter aquaeclarae*	WP_093207878.1	WP_093203666.1	WP_093200151.1
Bacteroidota	*Snuella lapsa*	WP_345003601.1	WP_345003664.1	WP_345007529.1
Bacteroidota	*Snuella sedimenti*	WP_199115617.1	WP_199116703.1	WP_199114629.1
Bacteroidota	*Solirubrum puertoriconensis*	WP_059067657.1	WP_059071390.1	WP_059068878.1
Bacteroidota	*Solitalea canadensis*	WP_014682578.1	WP_014680712.1	WP_014681131.1
Bacteroidota	*Solitalea koreensis*	WP_142603364.1	WP_142604438.1	WP_142600541.1
Bacteroidota	*Solitalea lacus*	WP_237848968.1	WP_237846591.1	WP_237847143.1
Bacteroidota	*Solitalea longa*	WP_103787391.1	WP_103790159.1	WP_103788875.1
Bacteroidota	*Soonwooa buanensis*	WP_079668157.1	WP_079667385.1	WP_079666718.1
Bacteroidota	*Soonwooa sp.*	WP_300672259.1	WP_300673687.1	WP_300674339.1
Bacteroidota	*Sphingobacterium alimentarium*	WP_132776151.1	WP_132777594.1	WP_132777741.1
Bacteroidota	*Sphingobacterium alkalisoli*	WP_136819553.1	WP_136821884.1	WP_136818617.1
Bacteroidota	*Sphingobacterium allocomposti*	WP_148907204.1	WP_148908684.1	WP_148909427.1
Bacteroidota	*Sphingobacterium arenae*	WP_190310305.1	WP_190308564.1	WP_190307389.1
Bacteroidota	*Sphingobacterium bambusae*	WP_320185828.1	WP_320184487.1	WP_320186568.1
Bacteroidota	*Sphingobacterium bovisgrunnientis*	WP_160068841.1	WP_160069544.1	WP_160070284.1
Bacteroidota	*Sphingobacterium bovistauri*	WP_225551901.1	WP_225550927.1	WP_225554940.1
Bacteroidota	*Sphingobacterium cavernae*	WP_149913121.1	WP_149915679.1	WP_149913630.1
Bacteroidota	*Sphingobacterium chungjuense*	WP_166332274.1	WP_166333473.1	WP_166332900.1
Bacteroidota	*Sphingobacterium chuzhouense*	WP_190315297.1	WP_190314194.1	WP_190312758.1
Bacteroidota	*Sphingobacterium composti*	WP_159634226.1	WP_159636159.1	WP_159634454.1
Bacteroidota	*Sphingobacterium corticibacter*	WP_116776166.1	WP_116774497.1	WP_116775555.1
Bacteroidota	*Sphingobacterium corticibacterium*	WP_130143238.1	WP_130139939.1	WP_130142176.1
Bacteroidota	*Sphingobacterium corticis*	WP_380867056.1	WP_380869886.1	WP_380868428.1

Bacteroidota	*Sphingobacterium daejeonense*	WP_260027722.1	WP_260033050.1	WP_380894417.1
Bacteroidota	*Sphingobacterium deserti*	WP_037495749.1	WP_037503513.1	WP_037497633.1
Bacteroidota	*Sphingobacterium detergens*	WP_120261583.1	WP_120259190.1	WP_418359127.1
Bacteroidota	*Sphingobacterium endophyticum*	WP_156306153.1	WP_156309128.1	WP_156307566.1
Bacteroidota	*Sphingobacterium faecale*	WP_202101697.1	WP_202102283.1	WP_202103280.1
Bacteroidota	*Sphingobacterium faecium*	WP_108160791.1	WP_346604585.1	WP_108159374.1
Bacteroidota	*Sphingobacterium gobiense*	WP_105727300.1	WP_105723300.1	WP_105725618.1
Bacteroidota	*Sphingobacterium griseoflavum*	WP_189625151.1	WP_189627229.1	WP_189625520.1
Bacteroidota	*Sphingobacterium haloxyli*	WP_105717127.1	WP_105716264.1	WP_105715364.1
Bacteroidota	*Sphingobacterium hotanense*	WP_149527035.1	WP_260044612.1	WP_260039951.1
Bacteroidota	*Sphingobacterium humi*	WP_160370061.1	WP_160367591.1	WP_160369867.1
Bacteroidota	*Sphingobacterium hungaricum*	WP_196935180.1	WP_196936872.1	WP_196935741.1
Bacteroidota	*Sphingobacterium kyonggiense*	WP_344673062.1	WP_344674680.1	WP_344675295.1
Bacteroidota	*Sphingobacterium lactis*	WP_103904721.1	WP_103906279.1	WP_103906628.1
Bacteroidota	*Sphingobacterium lumbrici*	WP_140937507.1	WP_140938382.1	WP_140937032.1
Bacteroidota	*Sphingobacterium micropteri*	WP_190995033.1	WP_190993283.1	WP_190994339.1
Bacteroidota	*Sphingobacterium multivorum*	WP_312744889.1	WP_313529588.1	WP_201666921.1
Bacteroidota	*Sphingobacterium nematocida*	WP_079641174.1	WP_079641716.1	WP_079644723.1
Bacteroidota	*Sphingobacterium olei*	WP_136900903.1	WP_136899696.1	WP_136900632.1
Bacteroidota	*Sphingobacterium oryzagri*	WP_274266794.1	WP_274269451.1	WP_274267454.1
Bacteroidota	*Sphingobacterium paludis*	WP_133639213.1	WP_133640419.1	WP_133639947.1
Bacteroidota	*Sphingobacterium paucimobilis*	WP_021068833.1	WP_021070158.1	WP_021071774.1
Bacteroidota	*Sphingobacterium pedocola*	WP_196937863.1	WP_196938004.1	WP_196939367.1
Bacteroidota	*Sphingobacterium phlebotomi*	WP_148919322.1	WP_148918764.1	WP_148918117.1
Bacteroidota	*Sphingobacterium prati*	WP_172393777.1	WP_172394118.1	WP_172392574.1
Bacteroidota	*Sphingobacterium psychroaquaticum*	WP_085472502.1	WP_085471717.1	WP_134430177.1
Bacteroidota	*Sphingobacterium puteale*	WP_121126256.1	WP_121123514.1	WP_121125361.1
Bacteroidota	*Sphingobacterium rhinopitheci*	WP_241790133.1	WP_241788344.1	WP_241790031.1

Bacteroidota	*Sphingobacterium shayense*	WP_172389207.1	WP_172389514.1	WP_172389689.1
Bacteroidota	*Sphingobacterium siyangense*	WP_424545452.1	WP_346068928.1	WP_205400216.1
Bacteroidota	*Sphingobacterium sp.*	WP_312337529.1	WP_343566882.1	WP_313261371.1
Bacteroidota	*Sphingobacterium spiritivorum*	WP_002992708.1	WP_115171584.1	WP_201681064.1
Bacteroidota	*Sphingobacterium suaedae*	WP_380903687.1	WP_380901132.1	WP_380900344.1
Bacteroidota	*Sphingobacterium tabacisoli*	WP_210355260.1	WP_210353897.1	WP_210355779.1
Bacteroidota	*Sphingobacterium thalpophilum*	WP_424543296.1	WP_282636042.1	WP_370481172.1
Bacteroidota	*Sphingobacterium thermophilum*	WP_345067253.1	WP_345068116.1	WP_345069121.1
Bacteroidota	*Sphingobacterium wenxiniae*	WP_093365916.1	WP_093363732.1	WP_093364962.1
Bacteroidota	*Sphingobacterium yanglingense*	WP_133585347.1	WP_133584895.1	WP_133586886.1
Bacteroidota	*Sphingobacterium zhuxiongii*	WP_153509740.1	WP_153511565.1	WP_153509167.1
Bacteroidota	*Spirosoma aerolatum*	WP_080058510.1	WP_080057299.1	WP_080053908.1
Bacteroidota	*Spirosoma agri*	WP_164036195.1	WP_164036465.1	WP_164041052.1
Bacteroidota	*Spirosoma arboris*	WP_157586299.1	WP_157589715.1	WP_157587946.1
Bacteroidota	*Spirosoma aureum*	WP_167215690.1	WP_167214910.1	WP_167204164.1
Bacteroidota	*Spirosoma endbachense*	WP_162391124.1	WP_162391301.1	WP_162388914.1
Bacteroidota	*Spirosoma endophyticum*	WP_093823250.1	WP_093828459.1	WP_093825618.1
Bacteroidota	*Spirosoma flavum*	WP_381497588.1	WP_381506423.1	WP_381508764.1
Bacteroidota	*Spirosoma fluviale*	WP_097128538.1	WP_097124341.1	WP_097130254.1
Bacteroidota	*Spirosoma foliorum*	WP_182457958.1	WP_182461395.1	WP_182459763.1
Bacteroidota	*Spirosoma lacussanchae*	WP_138499346.1	WP_138500323.1	WP_138507235.1
Bacteroidota	*Spirosoma linguale*	WP_012928013.1	WP_012925276.1	WP_012929824.1
Bacteroidota	*Spirosoma luteum*	WP_018621355.1	WP_018619126.1	WP_018617925.1
Bacteroidota	*Spirosoma montaniterrae*	WP_077131233.1	WP_077131579.1	WP_077132411.1
Bacteroidota	*Spirosoma oryzae*	WP_106140358.1	WP_106137905.1	WP_106136314.1
Bacteroidota	*Spirosoma panaciterrae*	WP_020600438.1	WP_020595618.1	WP_020598523.1
Bacteroidota	*Spirosoma pollinicola*	WP_100987614.1	WP_100986176.1	WP_100988906.1
Bacteroidota	*Spirosoma profusum*	WP_190888856.1	WP_190888710.1	WP_190889448.1

Bacteroidota	*Spirosoma radiotolerans*	WP_046576037.1	WP_046376221.1	WP_046578030.1
Bacteroidota	*Spirosoma rigui*	WP_080238489.1	WP_080241085.1	WP_080238045.1
Bacteroidota	*Spirosoma soli*	WP_381520133.1	WP_381518935.1	WP_381524593.1
Bacteroidota	*Spirosoma sp.*	WP_420149467.1	WP_420150375.1	WP_420151292.1
Bacteroidota	*Spirosoma spitsbergense*	WP_020601793.1	WP_020602422.1	WP_020607185.1
Bacteroidota	*Spirosoma taeanense*	WP_171740860.1	WP_171739650.1	WP_171741738.1
Bacteroidota	*Spirosoma terrae*	WP_163946965.1	WP_163944895.1	WP_163941605.1
Bacteroidota	*Spirosoma utsteinense*	WP_186739525.1	WP_186739119.1	WP_186737346.1
Bacteroidota	*Spirosoma validum*	WP_191036995.1	WP_191039184.1	WP_191040336.1
Bacteroidota	*Splendidivirga corallicola*	WP_346754110.1	WP_346754927.1	WP_346750622.1
Bacteroidota	*Spongiimicrobium salis*	WP_394749418.1	WP_394749267.1	WP_394749767.1
Bacteroidota	*Spongiimicrobium sp. 3-5*	WP_411032168.1	WP_411030488.1	WP_411031717.1
Bacteroidota	*Spongiivirga citrea*	WP_164029170.1	WP_164028867.1	WP_164032428.1
Bacteroidota	*Spongiivirga sp. MCCC 1A20706*	WP_410484950.1	WP_410483661.1	WP_410485712.1
Bacteroidota	*Sporocytophaga myxococcoides*	WP_045459397.1	WP_045464077.1	WP_045459954.1
Bacteroidota	*Sporocytophaga sp.*	WP_293897300.1	WP_293894613.1	WP_293892478.1
Bacteroidota	*Subsaxibacter sp. CAU 1640*	WP_248025836.1	WP_248025383.1	WP_248024187.1
Bacteroidota	*Subsaximicrobium wynnwilliamsii*	WP_147084534.1	WP_147085199.1	WP_317128650.1
Bacteroidota	*Sungkyunkwania multivorans*	WP_386407340.1	WP_386405566.1	WP_386407050.1
Bacteroidota	*Taibaiella chishuiensis*	WP_106525076.1	WP_106521535.1	WP_181358454.1
Bacteroidota	*Taibaiella helva*	WP_118953545.1	WP_118949944.1	WP_157976869.1
Bacteroidota	*Taibaiella koreensis*	WP_118972826.1	WP_118974487.1	WP_162903180.1
Bacteroidota	*Taibaiella lutea*	WP_150031081.1	WP_150033014.1	WP_190277335.1
Bacteroidota	*Taibaiella soli*	WP_110999133.1	WP_110996983.1	WP_110999509.1
Bacteroidota	*Taibaiella sp. KBW10*	WP_124636061.1	WP_124635204.1	WP_124636155.1
Bacteroidota	*Taishania pollutisoli*	WP_163492192.1	WP_163490697.1	WP_216714193.1
Bacteroidota	*Tamlana crocina*	WP_167917677.1	WP_167919677.1	WP_167918689.1
Bacteroidota	*Tamlana flava*	WP_370479806.1	WP_370476504.1	WP_370476851.1

Bacteroidota	*Tamlana sp. I1*	WP_194767652.1	WP_194767741.1	WP_194768077.1
Bacteroidota	*Telluribacter humicola*	WP_207505055.1	WP_207507151.1	WP_310586155.1
Bacteroidota	*Telluribacter sp.*	WP_327487111.1	WP_327487727.1	WP_327484708.1
Bacteroidota	*Tellurirhabdus bombi*	WP_234735125.1	WP_234734379.1	WP_234736306.1
Bacteroidota	*Tellurirhabdus rosea*	WP_266368610.1	WP_266365853.1	WP_266369101.1
Bacteroidota	*Tenacibaculum adriaticum*	WP_148869874.1	WP_148871029.1	WP_148871047.1
Bacteroidota	*Tenacibaculum aestuarii*	WP_408035614.1	WP_408036213.1	WP_408036178.1
Bacteroidota	*Tenacibaculum aestuariivivum*	WP_418650557.1	WP_418649929.1	WP_418649959.1
Bacteroidota	*Tenacibaculum agarivorans*	WP_075342671.1	WP_075343661.1	WP_075343920.1
Bacteroidota	*Tenacibaculum aiptasiae*	WP_420552946.1	WP_420551224.1	WP_272151765.1
Bacteroidota	*Tenacibaculum amylolyticum*	WP_408039680.1	WP_408039792.1	WP_408037409.1
Bacteroidota	*Tenacibaculum ascidiaceicola*	WP_425658519.1	WP_415268493.1	WP_415268506.1
Bacteroidota	*Tenacibaculum caenipelagi*	WP_133536489.1	WP_133534486.1	WP_133534460.1
Bacteroidota	*Tenacibaculum crassostreae*	WP_408046929.1	WP_408046466.1	WP_408046492.1
Bacteroidota	*Tenacibaculum dicentrarchi*	WP_101902041.1	WP_101902640.1	WP_370407980.1
Bacteroidota	*Tenacibaculum discolor*	WP_124588550.1	WP_124590251.1	WP_099213840.1
Bacteroidota	*Tenacibaculum finnmarkense*	WP_101915721.1	WP_232125399.1	WP_193707775.1
Bacteroidota	*Tenacibaculum gallaicum*	WP_115901618.1	WP_115900961.1	WP_115900927.1
Bacteroidota	*Tenacibaculum geojense*	WP_386104704.1	WP_386104940.1	WP_386104979.1
Bacteroidota	*Tenacibaculum holothuriorum*	WP_086029382.1	WP_086030732.1	WP_086030767.1
Bacteroidota	*Tenacibaculum insulae*	WP_418644914.1	WP_418644674.1	WP_418644704.1
Bacteroidota	*Tenacibaculum jejuense*	WP_095072327.1	WP_095073068.1	WP_095072980.1
Bacteroidota	*Tenacibaculum larymnensis*	WP_274639816.1	WP_274640215.1	WP_274640185.1
Bacteroidota	*Tenacibaculum litopenaei*	WP_408044075.1	WP_408043301.1	WP_408044502.1
Bacteroidota	*Tenacibaculum lutimaris*	WP_120186681.1	WP_120187165.1	WP_120187140.1
Bacteroidota	*Tenacibaculum maritimum*	WP_406741777.1	WP_232641412.1	WP_159245217.1
Bacteroidota	*Tenacibaculum mesophilum*	WP_281979306.1	WP_253679894.1	WP_281980437.1
Bacteroidota	*Tenacibaculum ovolyticum*	WP_422091942.1	WP_422092133.1	WP_237276067.1

Bacteroidota	*Tenacibaculum pelagium*	WP_182123523.1	WP_182123957.1	WP_182123931.1
Bacteroidota	*Tenacibaculum piscium*	WP_101917182.1	WP_101916480.1	WP_101916845.1
Bacteroidota	*Tenacibaculum platacis*	WP_348725202.1	WP_348742779.1	WP_348724435.1
Bacteroidota	*Tenacibaculum polynesiense*	WP_348716780.1	WP_348715293.1	WP_348718874.1
Bacteroidota	*Tenacibaculum sediminilitoris*	WP_408024139.1	WP_408023732.1	WP_408025091.1
Bacteroidota	*Tenacibaculum singaporense*	WP_125067654.1	WP_125344468.1	WP_125068044.1
Bacteroidota	*Tenacibaculum skagerrakense*	WP_132794789.1	WP_132795009.1	WP_132795025.1
Bacteroidota	*Tenacibaculum soleae*	WP_068705066.1	WP_418654330.1	WP_271407244.1
Bacteroidota	*Tenacibaculum sp.*	WP_273694360.1	WP_417799530.1	WP_417786282.1
Bacteroidota	*Tenacibaculum tangerinum*	WP_279652411.1	WP_279651372.1	WP_279651401.1
Bacteroidota	*Tenacibaculum todarodis*	WP_072554883.1	WP_072555161.1	WP_072555142.1
Bacteroidota	*Tenacibaculum vairaonense*	WP_348707420.1	WP_348702102.1	WP_348746797.1
Bacteroidota	*Tenacibaculum xiamenense*	WP_408031608.1	WP_408030665.1	WP_408032154.1
Bacteroidota	*Terrimonas ferruginea*	WP_028787882.1	WP_303892481.1	WP_303891090.1
Bacteroidota	*Terrimonas ginsenosidimutans*	WP_237871313.1	WP_237876689.1	WP_237877120.1
Bacteroidota	*Terrimonas pollutisoli*	WP_276500859.1	WP_276504086.1	WP_276502652.1
Bacteroidota	*Terrimonas rubra*	WP_386097367.1	WP_386096749.1	WP_386099849.1
Bacteroidota	*Terrimonas sp.*	WP_116875316.1	WP_116872616.1	WP_116872878.1
Bacteroidota	*Thalassobellus suaedae*	WP_415866465.1	WP_415861774.1	WP_415862084.1
Bacteroidota	*Thermaurantimonas aggregans*	WP_124396826.1	WP_124397494.1	WP_160160588.1
Bacteroidota	*Thermaurantimonas sp.*	WP_409769462.1	WP_409771203.1	WP_409771132.1
Bacteroidota	*Thermoflavifilum aggregans*	WP_100313719.1	WP_100314561.1	WP_100314460.1
Bacteroidota	*Thermoflavifilum sp.*	WP_297042760.1	WP_297044056.1	WP_297043879.1
Bacteroidota	*Thermoflavifilum thermophilum*	WP_092457765.1	WP_092461028.1	WP_092460516.1
Bacteroidota	*Thermoflexibacter ruber*	WP_091545755.1	WP_091545253.1	WP_091542748.1
Bacteroidota	*Thermonema lapsum*	WP_166919763.1	WP_166920515.1	WP_166918106.1
Bacteroidota	*Thermonema rossianum*	WP_051632972.1	WP_038030079.1	WP_084147099.1
Bacteroidota	*Thermonema sp.*	WP_288005729.1	WP_288007082.1	WP_288004089.1

Bacteroidota	*Tunicatimonas pelagia*	WP_302205012.1	WP_302207186.1	WP_302204998.1
Bacteroidota	*Ulvibacter antarcticus*	WP_121906710.1	WP_121907624.1	WP_121907465.1
Bacteroidota	*Ulvibacter litoralis*	WP_093143547.1	WP_093144987.1	WP_093145223.1
Bacteroidota	*Ulvibacter sp. MAR_2010_11*	WP_100803909.1	WP_100804196.1	WP_100803434.1
Bacteroidota	*Ulvibacterium marinum*	WP_282162937.1	WP_120711029.1	WP_120713983.1
Bacteroidota	*Ulvibacterium sp.*	WP_425236708.1	WP_422080450.1	WP_422084048.1
Bacteroidota	*Urechidicola croceus*	WP_070235832.1	WP_070235596.1	WP_070236178.1
Bacteroidota	*Urechidicola vernalis*	WP_311591836.1	WP_311592154.1	WP_311594012.1
Bacteroidota	*Vaginella massiliensis*	WP_068598256.1	WP_068595181.1	WP_395090947.1
Bacteroidota	*Vicingus serpentipes*	WP_147101768.1	WP_147097973.1	WP_147100110.1
Bacteroidota	*Wandonia haliotis*	WP_343788488.1	WP_343787892.1	WP_343785738.1
Bacteroidota	*Wenyingzhuangia aestuarii*	WP_167898441.1	WP_167897819.1	WP_167896793.1
Bacteroidota	*Wenyingzhuangia fucanilytica*	WP_068826438.1	WP_068825555.1	WP_068826364.1
Bacteroidota	*Wenyingzhuangia gilva*	WP_302883866.1	WP_302885059.1	WP_302883913.1
Bacteroidota	*Wenyingzhuangia heitensis*	WP_167185859.1	WP_167187886.1	WP_167187308.1
Bacteroidota	*Wenyingzhuangia marina*	WP_073119375.1	WP_073118897.1	WP_073119261.1
Bacteroidota	*Wenyingzhuangia sp.*	WP_347182783.1	WP_347180434.1	WP_347181761.1
Bacteroidota	*Winogradskyella alexanderae*	WP_224530615.1	WP_224526489.1	WP_224526926.1
Bacteroidota	*Winogradskyella algicola*	WP_138433396.1	WP_138434160.1	WP_138434612.1
Bacteroidota	*Winogradskyella aquimaris*	WP_320556630.1	WP_320556356.1	WP_320555584.1
Bacteroidota	*Winogradskyella arenosi*	WP_114309489.1	WP_114309772.1	WP_114310057.1
Bacteroidota	*Winogradskyella aurantia*	WP_094968397.1	WP_094967377.1	WP_094968831.1
Bacteroidota	*Winogradskyella aurantiaca*	WP_115462847.1	WP_115463094.1	WP_317047361.1
Bacteroidota	*Winogradskyella bathintestinalis*	WP_290206804.1	WP_290206994.1	WP_290206180.1
Bacteroidota	*Winogradskyella costae*	WP_179335048.1	WP_179334623.1	WP_179335300.1
Bacteroidota	*Winogradskyella echinorum*	WP_186845557.1	WP_186845790.1	WP_186845877.1
Bacteroidota	*Winogradskyella eckloniae*	WP_173281061.1	WP_173280570.1	WP_173281378.1
Bacteroidota	*Winogradskyella endarachnes*	WP_157361762.1	WP_157363281.1	WP_157362488.1

Bacteroidota	*Winogradskyella epiphytica*	WP_110475170.1	WP_110476132.1	WP_110476237.1
Bacteroidota	*Winogradskyella eximia*	WP_282031934.1	WP_282032511.1	WP_115817452.1
Bacteroidota	*Winogradskyella flava*	WP_282042702.1	WP_185789937.1	WP_185789761.1
Bacteroidota	*Winogradskyella forsetii*	WP_179008254.1	WP_179006261.1	WP_179005818.1
Bacteroidota	*Winogradskyella haliclonae*	WP_188374348.1	WP_188374596.1	WP_188375109.1
Bacteroidota	*Winogradskyella helgolandensis*	WP_178985666.1	WP_218646038.1	WP_179319418.1
Bacteroidota	*Winogradskyella immobilis*	WP_227477848.1	WP_227476698.1	WP_227477668.1
Bacteroidota	*Winogradskyella jejuensis*	WP_073086886.1	WP_073087204.1	WP_073085722.1
Bacteroidota	*Winogradskyella litorisediminis*	WP_386127183.1	WP_386132737.1	WP_386132081.1
Bacteroidota	*Winogradskyella litoriviva*	WP_173300105.1	WP_173301905.1	WP_173300484.1
Bacteroidota	*Winogradskyella ludwigii*	WP_179338973.1	WP_179339464.1	WP_179338155.1
Bacteroidota	*Winogradskyella luteola*	WP_218544848.1	WP_218544234.1	WP_218544490.1
Bacteroidota	*Winogradskyella maritima*	WP_386101441.1	WP_386095869.1	WP_386099159.1
Bacteroidota	*Winogradskyella pacifica*	WP_179347853.1	WP_179349892.1	WP_179349795.1
Bacteroidota	*Winogradskyella pelagia*	WP_208154871.1	WP_208153767.1	WP_208154100.1
Bacteroidota	*Winogradskyella poriferorum*	WP_331809773.1	WP_331810245.1	WP_331809409.1
Bacteroidota	*Winogradskyella psychrotolerans*	WP_215925849.1	WP_215925180.1	WP_215936225.1
Bacteroidota	*Winogradskyella pulchriflava*	WP_386061894.1	WP_386061462.1	WP_386064116.1
Bacteroidota	*Winogradskyella rapida*	WP_386114648.1	WP_386113114.1	WP_386117398.1
Bacteroidota	*Winogradskyella schleiferi*	WP_178988830.1	WP_178988226.1	WP_178987854.1
Bacteroidota	*Winogradskyella sediminis*	WP_092443319.1	WP_115839024.1	WP_417875597.1
Bacteroidota	*Winogradskyella sp.*	WP_418638804.1	WP_417871518.1	WP_370000453.1
Bacteroidota	*Winogradskyella tangerina*	WP_111682739.1	WP_111684095.1	WP_111683804.1
Bacteroidota	*Winogradskyella thalassocola*	WP_092469884.1	WP_092467141.1	WP_092469187.1
Bacteroidota	*Winogradskyella undariae*	WP_179315410.1	WP_179317035.1	WP_173588138.1
Bacteroidota	*Winogradskyella ursingii*	WP_179344862.1	WP_179346499.1	WP_179346073.1
Bacteroidota	*Winogradskyella vidalii*	WP_179353704.1	WP_179352429.1	WP_179351812.1
Bacteroidota	*Winogradskyella vincentii*	WP_224478105.1	WP_224479179.1	WP_224478740.1

Bacteroidota	*Winogradskyella wandonensis*	WP_132703720.1	WP_132705881.1	WP_132702815.1
Bacteroidota	*Winogradskyella wichelsiae*	WP_179376959.1	WP_179376871.1	WP_179376516.1
Bacteroidota	*Wocania arenilitoris*	WP_237240459.1	WP_237239480.1	WP_237240506.1
Bacteroidota	*Wocania ichthyoenteri*	WP_034044660.1	WP_034040352.1	WP_034041167.1
Bacteroidota	*Xanthocytophaga agilis*	WP_314516216.1	WP_314508697.1	WP_314512272.1
Bacteroidota	*Xanthocytophaga flavus*	WP_313990804.1	WP_314031117.1	WP_313990763.1
Bacteroidota	*Xanthomarina gelatinilytica*	WP_417866332.1	WP_417858572.1	WP_417855412.1
Bacteroidota	*Xanthomarina sp.*	WP_324313051.1	WP_324313525.1	WP_324313434.1
Bacteroidota	*Xanthomarina spongicola*	WP_109681702.1	WP_109681507.1	WP_109682487.1
Bacteroidota	*Yeosuana aromativorans*	WP_188653250.1	WP_188654997.1	WP_188652436.1
Bacteroidota	*Yeosuana marina*	WP_339917917.1	WP_166963373.1	WP_339917623.1
Bacteroidota	*Zeaxanthinibacter enoshimensis*	WP_373074604.1	WP_373074286.1	WP_373072286.1
Bacteroidota	*Zeaxanthinibacter sp. PT1*	WP_272595066.1	WP_272593691.1	WP_272594309.1
Bacteroidota	*Zhouia amylolytica*	WP_038262980.1	WP_038262849.1	WP_255083157.1
Bacteroidota	*Zhouia sp. PK063*	WP_399473499.1	WP_399471047.1	WP_399471254.1
Bacteroidota	*Zhouia spongiae*	WP_242936813.1	WP_242936238.1	WP_242937265.1
Bacteroidota	*Zobellia alginiliquefaciens*	WP_276166266.1	WP_276166540.1	WP_276169207.1
Bacteroidota	*Zobellia amurskyensis*	WP_038233676.1	WP_155598589.1	WP_155600564.1
Bacteroidota	*Zobellia barbeyronii*	WP_214613186.1	WP_214610970.1	WP_214611862.1
Bacteroidota	*Zobellia nedashkovskayae*	WP_194534562.1	WP_194535239.1	WP_194534115.1
Bacteroidota	*Zobellia roscoffensis*	WP_194526843.1	WP_194530383.1	WP_194526398.1
Bacteroidota	*Zobellia russellii*	WP_400073611.1	WP_400073453.1	WP_400073937.1
Bacteroidota	*Zobellia sp. B3R18*	WP_215914274.1	WP_215912230.1	WP_215912435.1
Bacteroidota	*Zobellia uliginosa*	WP_215938390.1	WP_215939514.1	WP_303399556.1
Bacteroidota	*Zunongwangia atlantica*	WP_084840657.1	WP_084841855.1	WP_084841803.1
Bacteroidota	*Zunongwangia endophytica*	WP_290231854.1	WP_290233323.1	WP_290233428.1
Bacteroidota	*Zunongwangia mangrovi*	WP_092540965.1	WP_092542863.1	WP_092542653.1
Bacteroidota	*Zunongwangia profunda*	WP_013072155.1	WP_041578784.1	WP_228251206.1

Bacteroidota	*Zunongwangia sp.*	WP_417887496.1	WP_347406885.1	WP_417885621.1
Balneolota	*Aliifodinibius salipaludis*	WP_095605785.1	WP_095607636.1	WP_255233382.1
Balneolota	*Aliifodinibius sp. S!AR15-10*	WP_310684926.1	WP_310682093.1	WP_310685409.1
Balneolota	*Balneola sp. EhC07*	WP_066222314.1	WP_066220100.1	WP_066217088.1
Balneolota	*Balneola vulgaris*	WP_018127418.1	WP_018126226.1	WP_018126604.1
Balneolota	*Cyclonatronum proteinivorum*	WP_114985548.1	WP_114983795.1	WP_164682663.1
Balneolota	*Cyclonatronum sp.*	WP_291482712.1	WP_291482818.1	WP_291482004.1
Balneolota	*Fodinibius halophilus*	WP_165266187.1	WP_165267505.1	WP_165265239.1
Balneolota	*Fodinibius roseus*	WP_073061524.1	WP_073063141.1	WP_073059135.1
Balneolota	*Fodinibius salicampi*	WP_265790485.1	WP_265788309.1	WP_265787781.1
Balneolota	*Fodinibius salinus*	WP_148898505.1	WP_246138157.1	WP_170245550.1
Balneolota	*Fodinibius saliphilus*	WP_138430825.1	WP_138430056.1	WP_138429484.1
Balneolota	*Fodinibius salsisoli*	WP_265764074.1	WP_265766003.1	WP_265765528.1
Balneolota	*Fodinibius sediminis*	WP_142712524.1	WP_142714645.1	WP_185958215.1
Balneolota	*Fodinibius sp.*	WP_322574622.1	WP_322575165.1	WP_372638409.1
Balneolota	*Gracilimonas amylolytica*	WP_103666029.1	WP_103663667.1	WP_103663801.1
Balneolota	*Gracilimonas halophila*	WP_390297046.1	WP_390300200.1	WP_390300637.1
Balneolota	*Gracilimonas mengyeensis*	WP_142455253.1	WP_142455020.1	WP_142452685.1
Balneolota	*Gracilimonas sediminicola*	WP_255134448.1	WP_255135815.1	WP_255135726.1
Balneolota	*Gracilimonas sp.*	WP_395079183.1	WP_395069629.1	WP_395080504.1
Balneolota	*Gracilimonas tropica*	WP_020404354.1	WP_020402163.1	WP_020402845.1
Balneolota	*Halalkalibaculum roseum*	WP_165138370.1	WP_165140079.1	WP_165140357.1
Balneolota	*Halalkalibaculum sp. DA3122*	WP_395259678.1	WP_395259273.1	WP_395261429.1
Balneolota	*Natronogracilivirga saccharolytica*	WP_210513032.1	WP_210509515.1	WP_210511334.1
Balneolota	*Rhodohalobacter barkolensis*	WP_101073066.1	WP_101071599.1	WP_101071812.1
Balneolota	*Rhodohalobacter halophilus*	WP_069131108.1	WP_069130964.1	WP_069131403.1
Balneolota	*Rhodohalobacter mucosus*	WP_109647779.1	WP_109646781.1	WP_109645422.1
Balneolota	*Rhodohalobacter sp.*	WP_372902930.1	WP_322571692.1	WP_322571913.1

Balneolota	*Rhodohalobacter sulfatireducens*	WP_237852468.1	WP_237853641.1	WP_237855134.1
Calditrichaeota	*Caldithrix abyssi*	WP_006929106.1	WP_044281486.1	WP_006931009.1
Chlamydiota	*Criblamydia sequanensis*	WP_041016448.1	WP_053331647.1	WP_041017369.1
Chlamydiota	*Estrella lausannensis*	WP_239414312.1	WP_098037590.1	WP_098038239.1
Chlamydiota	*Waddlia chondrophila*	WP_041941457.1	WP_013181378.1	WP_013181809.1
Cyanobacteriota	*Leptolyngbya sp. 7M*	WP_211624753.1	WP_211627088.1	WP_211627697.1
Deinococcota	*Allomeiothermus silvanus*	WP_276958151.1	WP_013159191.1	WP_276956435.1
Deinococcota	*Calidithermus chliarophilus*	WP_027893954.1	WP_027892264.1	WP_027893633.1
Deinococcota	*Calidithermus roseus*	WP_119275938.1	WP_119278785.1	WP_119278386.1
Deinococcota	*Calidithermus terrae*	WP_119313774.1	WP_119316488.1	WP_119314295.1
Deinococcota	*Calidithermus timidus*	WP_018465601.1	WP_018466689.1	WP_018467417.1
Deinococcota	*Deinobacterium chartae*	WP_183983655.1	WP_183987798.1	WP_343058308.1
Deinococcota	*Deinococcus actinosclerus*	WP_231724576.1	WP_062159268.1	WP_062157899.1
Deinococcota	*Deinococcus aerius*	WP_103129980.1	WP_103130965.1	WP_103128680.1
Deinococcota	*Deinococcus aerolatus*	WP_188969226.1	WP_188974266.1	WP_188969069.1
Deinococcota	*Deinococcus aerophilus*	WP_188902948.1	WP_188905080.1	WP_188901788.1
Deinococcota	*Deinococcus aestuarii*	WP_216317724.1	WP_216323892.1	WP_216328636.1
Deinococcota	*Deinococcus aetherius*	WP_264774375.1	WP_264775877.1	WP_264774420.1
Deinococcota	*Deinococcus alpinitundrae*	WP_237724992.1	WP_161882051.1	WP_161882035.1
Deinococcota	*Deinococcus altitudinis*	WP_407570891.1	WP_407569033.1	WP_407570468.1
Deinococcota	*Deinococcus aluminii*	WP_345451717.1	WP_345458031.1	WP_345451657.1
Deinococcota	*Deinococcus antarcticus*	WP_380077259.1	WP_380079366.1	WP_380076048.1
Deinococcota	*Deinococcus apachensis*	WP_019585681.1	WP_026332545.1	WP_019585857.1
Deinococcota	*Deinococcus aquaedulcis*	WP_221088799.1	WP_328774622.1	WP_221089802.1
Deinococcota	*Deinococcus aquatilis*	WP_019008767.1	WP_019010936.1	WP_019009930.1
Deinococcota	*Deinococcus aquiradiocola*	WP_188960394.1	WP_188964532.1	WP_188960829.1
Deinococcota	*Deinococcus arcticus*	WP_199188265.1	WP_158263797.1	WP_107136680.1
Deinococcota	*Deinococcus arenae*	WP_229781077.1	WP_162621388.1	WP_110829477.1

Deinococcota	*Deinococcus arenicola*	WP_317639329.1	WP_317641462.1	WP_317640175.1
Deinococcota	*Deinococcus budaensis*	WP_184024512.1	WP_246363429.1	WP_184024438.1
Deinococcota	*Deinococcus caeni*	WP_345444445.1	WP_345441182.1	WP_345441909.1
Deinococcota	*Deinococcus carri*	WP_345461590.1	WP_345466657.1	WP_345461676.1
Deinococcota	*Deinococcus cavernae*	WP_119761767.1	WP_119764245.1	WP_119762871.1
Deinococcota	*Deinococcus cellulosilyticus*	WP_146882446.1	WP_146886834.1	WP_246130609.1
Deinococcota	*Deinococcus daejeonensis*	WP_189053633.1	WP_189055309.1	WP_189053065.1
Deinococcota	*Deinococcus depolymerans*	WP_343760772.1	WP_343758711.1	WP_343755778.1
Deinococcota	*Deinococcus deserti*	WP_012693197.1	WP_162485366.1	WP_012693436.1
Deinococcota	*Deinococcus detaillensis*	WP_143721285.1	WP_143719968.1	WP_143719870.1
Deinococcota	*Deinococcus ficus*	WP_027462627.1	WP_022801228.1	WP_027461688.1
Deinococcota	*Deinococcus fonticola*	WP_135227646.1	WP_135229883.1	WP_135230392.1
Deinococcota	*Deinococcus frigens*	WP_029476876.1	WP_029478215.1	WP_029477453.1
Deinococcota	*Deinococcus gobiensis*	WP_014685004.1	WP_043800282.1	WP_014684828.1
Deinococcota	*Deinococcus grandis*	WP_058976710.1	WP_058974927.1	WP_058976407.1
Deinococcota	*Deinococcus hohokamensis*	WP_380059839.1	WP_380062069.1	WP_380060145.1
Deinococcota	*Deinococcus hopiensis*	WP_084049044.1	WP_084050723.1	WP_084048923.1
Deinococcota	*Deinococcus humi*	WP_184129699.1	WP_184126902.1	WP_184129920.1
Deinococcota	*Deinococcus irradiatisoli*	WP_245896078.1	WP_109827479.1	WP_109827008.1
Deinococcota	*Deinococcus koreensis*	WP_103309064.1	WP_103313291.1	WP_103312787.1
Deinococcota	*Deinococcus kurensis*	WP_162393704.1	WP_235910409.1	WP_155297836.1
Deinococcota	*Deinococcus malanensis*	WP_189005511.1	WP_229780756.1	WP_189004304.1
Deinococcota	*Deinococcus maricopensis*	WP_013557152.1	WP_013555732.1	WP_013557142.1
Deinococcota	*Deinococcus marmoris*	WP_075831195.1	WP_075831896.1	WP_075835214.1
Deinococcota	*Deinococcus metalli*	WP_184109496.1	WP_184108687.1	WP_221275190.1
Deinococcota	*Deinococcus metallilatus*	WP_129119165.1	WP_129117670.1	WP_129119133.1
Deinococcota	*Deinococcus misasensis*	WP_034338289.1	WP_034339836.1	WP_034337961.1
Deinococcota	*Deinococcus multiflagellatus*	WP_224603931.1	WP_380056235.1	WP_224605968.1

Deinococcota	*Deinococcus navajonensis*	WP_380036978.1	WP_380041639.1	WP_380037409.1
Deinococcota	*Deinococcus oregonensis*	WP_380008879.1	WP_380013361.1	WP_380009835.1
Deinococcota	*Deinococcus peraridilitoris*	WP_041230988.1	WP_015234773.1	WP_015237366.1
Deinococcota	*Deinococcus petrolearius*	WP_380047487.1	WP_380048497.1	WP_380047843.1
Deinococcota	*Deinococcus phoenicis*	WP_034358017.1	WP_034354440.1	WP_034358065.1
Deinococcota	*Deinococcus piscis*	WP_189643464.1	WP_189641973.1	WP_189643702.1
Deinococcota	*Deinococcus planocerae*	WP_102125389.1	WP_102126075.1	WP_102125342.1
Deinococcota	*Deinococcus psychrotolerans*	WP_124872848.1	WP_124867313.1	WP_124867048.1
Deinococcota	*Deinococcus puniceus*	WP_157451107.1	WP_064013629.1	WP_064014463.1
Deinococcota	*Deinococcus radiodurans*	WP_051618804.1	WP_010888991.1	WP_027479890.1
Deinococcota	*Deinococcus radiomollis*	WP_407541532.1	WP_407540184.1	WP_407538780.1
Deinococcota	*Deinococcus radiophilus*	WP_126351677.1	WP_126352099.1	WP_229253139.1
Deinococcota	*Deinococcus radiopugnans*	WP_221265535.1	WP_039686665.1	WP_039683546.1
Deinococcota	*Deinococcus radiotolerans*	WP_189067161.1	WP_189068310.1	WP_189067021.1
Deinococcota	*Deinococcus reticulitermitis*	WP_092265124.1	WP_177183183.1	WP_092262682.1
Deinococcota	*Deinococcus roseus*	WP_229684599.1	WP_188999069.1	WP_229684699.1
Deinococcota	*Deinococcus rubellus*	WP_260561884.1	WP_260559421.1	WP_260559226.1
Deinococcota	*Deinococcus ruber*	WP_189087755.1	WP_189092038.1	WP_189092761.1
Deinococcota	*Deinococcus saxicola*	WP_415784007.1	WP_415785443.1	WP_415785112.1
Deinococcota	*Deinococcus soli (ex Cha et al. 2016)*	WP_046844773.1	WP_309855596.1	WP_309849145.1
Deinococcota	*Deinococcus sonorensis*	WP_350242827.1	WP_350244490.1	WP_350245292.1
Deinococcota	*Deinococcus sp.*	WP_303100937.1	WP_291426961.1	WP_291425733.1
Deinococcota	*Deinococcus taeanensis*	WP_225477265.1	WP_225475795.1	WP_225477183.1
Deinococcota	*Deinococcus taklimakanensis*	WP_386843827.1	WP_386843423.1	WP_386842091.1
Deinococcota	*Deinococcus terrestris*	WP_152868895.1	WP_152871367.1	WP_322618497.1
Deinococcota	*Deinococcus wulumuqiensis*	WP_114671372.1	WP_025568165.1	WP_278911727.1
Deinococcota	*Deinococcus xinjiangensis*	WP_353540710.1	WP_353541224.1	WP_353543416.1
Deinococcota	*Deinococcus yavapaiensis*	WP_110886036.1	WP_110886876.1	WP_110887518.1

Deinococcota	*Deinococcus yunweiensis*	WP_412029121.1	WP_412029680.1	WP_412029553.1
Deinococcota	*Marinithermus hydrothermalis*	WP_013703448.1	WP_013704541.1	WP_013702922.1
Deinococcota	*Meiothermus granaticius*	WP_119357307.1	WP_240631290.1	WP_119358206.1
Deinococcota	*Meiothermus sp.*	WP_337868470.1	WP_295397524.1	WP_314135743.1
Deinococcota	*Oceanithermus desulfurans*	WP_147148152.1	WP_147145011.1	WP_183677849.1
Deinococcota	*Oceanithermus profundus*	WP_013457204.1	WP_013458147.1	WP_013456947.1
Deinococcota	*Oceanithermus sp.*	WP_293171335.1	WP_287372649.1	WP_287408025.1
Deinococcota	*Thermus albus*	WP_243027396.1	WP_243027258.1	WP_243029035.1
Deinococcota	*Thermus altitudinis*	WP_243029601.1	WP_243029378.1	WP_243031917.1
Deinococcota	*Thermus amyloliquefaciens*	WP_038058845.1	WP_038056350.1	WP_038058292.1
Deinococcota	*Thermus antranikianii*	WP_028493307.1	WP_028493165.1	WP_028493524.1
Deinococcota	*Thermus aquaticus*	WP_053768318.1	WP_053767951.1	WP_003043996.1
Deinococcota	*Thermus arciformis*	WP_093007318.1	WP_093005597.1	WP_093004707.1
Deinococcota	*Thermus brockianus*	WP_071676276.1	WP_071677682.1	WP_071677286.1
Deinococcota	*Thermus caldifontis*	WP_205387548.1	WP_114312579.1	WP_114312959.1
Deinococcota	*Thermus calditerrae*	WP_038046263.1	WP_234553299.1	WP_234554877.1
Deinococcota	*Thermus composti*	WP_188847462.1	WP_188845202.1	WP_188847932.1
Deinococcota	*Thermus filiformis*	WP_038061065.1	WP_038060589.1	WP_038067687.1
Deinococcota	*Thermus igniterrae*	WP_018112247.1	WP_018112198.1	WP_018111336.1
Deinococcota	*Thermus islandicus*	WP_022798861.1	WP_022797977.1	WP_022797816.1
Deinococcota	*Thermus oshimai*	WP_018461480.1	WP_016329451.1	WP_016328891.1
Deinococcota	*Thermus parvatiensis*	WP_060384412.1	WP_060384356.1	WP_008633192.1
Deinococcota	*Thermus scotoductus*	WP_172955583.1	WP_038068734.1	WP_038070430.1
Deinococcota	*Thermus sediminis*	WP_117237037.1	WP_117236739.1	WP_117238527.1
Deinococcota	*Thermus sp.*	WP_026328956.1	WP_370533807.1	WP_347241700.1
Deinococcota	*Thermus tengchongensis*	WP_234557131.1	WP_234557249.1	WP_135343133.1
Deinococcota	*Thermus thermamylovorans*	WP_130839858.1	WP_130839531.1	WP_130841377.1
Deinococcota	*Thermus thermophilus*	WP_165738579.1	WP_203970898.1	WP_223903362.1

Deinococcota	*Truepera radiovictrix*	WP_013178337.1	WP_013177075.1	WP_013177872.1
Gemmatimonadota	*Candidatus Palauibacter irciniicola*	WP_419859026.1	WP_419856829.1	WP_419858894.1
Gemmatimonadota	*Candidatus Palauibacter polyketidifaciens*	WP_310783687.1	WP_310785236.1	WP_310785103.1
Gemmatimonadota	*Candidatus Palauibacter scopulicola*	WP_310777503.1	WP_310775338.1	WP_310778328.1
Gemmatimonadota	*Candidatus Palauibacter soopunensis*	WP_310758907.1	WP_310757617.1	WP_310759045.1
Gemmatimonadota	*Gemmatimonas aurantiaca*	WP_337170776.1	WP_012682624.1	WP_012683085.1
Gemmatimonadota	*Gemmatimonas groenlandica*	WP_171224594.1	WP_171225058.1	WP_171224533.1
Gemmatimonadota	*Gemmatimonas phototrophica*	WP_026850599.1	WP_026850308.1	WP_158514790.1
Gemmatimonadota	*Gemmatimonas sp.*	WP_396213143.1	WP_396214343.1	WP_309669755.1
Gemmatimonadota	*Gemmatirosa kalamazoonensis*	WP_025412160.1	WP_025411901.1	WP_025412232.1
Gemmatimonadota	*Longimicrobium sp.*	WP_331875729.1	WP_331053369.1	WP_331074245.1
Gemmatimonadota	*Longimicrobium terrae*	WP_170038756.1	WP_170035611.1	WP_170036976.1
Gemmatimonadota	*Pseudogemmatithrix spongiicola*	WP_367887761.1	WP_367887364.1	WP_367887824.1
Gemmatimonadota	*Roseisolibacter agri*	WP_284351024.1	WP_284350742.1	WP_284351645.1
Ignavibacteriota	*Ignavibacterium album*	WP_304142912.1	WP_014560830.1	WP_304130095.1
Ignavibacteriota	*Ignavibacterium sp.*	WP_297836878.1	WP_337867012.1	WP_337872978.1
Myxococcota	*Archangium lipolyticum*	WP_257455266.1	WP_257455265.1	WP_257455264.1
Myxococcota	*Archangium minus*	WP_395823165.1	WP_395823167.1	WP_395823169.1
Myxococcota	*Archangium primigenium*	WP_204487858.1	WP_204487860.1	WP_204487862.1
Myxococcota	*Archangium sp.*	WP_331119032.1	WP_331102339.1	WP_331102341.1
Myxococcota	*Archangium violaceum*	WP_204220669.1	WP_395843542.1	WP_239015428.1
Myxococcota	*Corallococcus aberystwythensis*	WP_120553254.1	WP_120553253.1	WP_208721103.1
Myxococcota	*Corallococcus coralloides*	WP_128795524.1	WP_014394710.1	WP_014394711.1
Myxococcota	*Corallococcus exercitus*	WP_375759901.1	WP_375759900.1	WP_375759899.1
Myxococcota	*Corallococcus exiguus*	WP_171810442.1	WP_206879771.1	WP_206879769.1
Myxococcota	*Corallococcus interemptor*	WP_427290402.1	WP_120546908.1	WP_120546907.1
Myxococcota	*Corallococcus macrosporus*	WP_207050954.1	WP_013938150.1	WP_193364466.1

Myxococcota	*Corallococcus sicarius*	WP_120623473.1	WP_120623474.1	WP_208718773.1
Myxococcota	*Corallococcus soli*	WP_193347660.1	WP_193347661.1	WP_193347662.1
Myxococcota	*Corallococcus sp. BB11-1*	WP_267556363.1	WP_267556362.1	WP_267556361.1
Myxococcota	*Hyalangium gracile*	WP_224246637.1	WP_224246631.1	WP_224246630.1
Myxococcota	*Hyalangium minutum*	WP_044181231.1	WP_044181229.1	WP_044181226.1
Myxococcota	*Hyalangium rubrum*	WP_321546729.1	WP_321546728.1	WP_321546727.1
Myxococcota	*Hyalangium sp.*	WP_324988804.1	WP_324988805.1	WP_422653662.1
Myxococcota	*Hyalangium versicolor*	WP_224364566.1	WP_224364567.1	WP_224364568.1
Myxococcota	*Melittangium boletus*	WP_095980233.1	WP_095980232.1	WP_095983038.1
Myxococcota	*Myxococcus dinghuensis*	WP_253972760.1	WP_253972761.1	WP_253972790.1
Myxococcota	*Myxococcus eversor*	WP_163870315.1	WP_163870316.1	WP_163870317.1
Myxococcota	*Myxococcus fulvus*	WP_408889278.1	WP_074951182.1	WP_408889280.1
Myxococcota	*Myxococcus guangdongensis*	WP_254042490.1	WP_254042489.1	WP_254042488.1
Myxococcota	*Myxococcus hansupus*	WP_002635932.1	WP_002635933.1	WP_002635934.1
Myxococcota	*Myxococcus landrumus*	WP_206714871.1	WP_206714872.1	WP_241757966.1
Myxococcota	*Myxococcus qinghaiensis*	WP_253996877.1	WP_253996878.1	WP_253996879.1
Myxococcota	*Myxococcus sp. AM010*	WP_176417252.1	WP_176417253.1	WP_176417254.1
Myxococcota	*Myxococcus stipitatus*	WP_338863836.1	WP_338863837.1	WP_338863838.1
Myxococcota	*Myxococcus vastator*	WP_163777287.1	WP_163777286.1	WP_176412746.1
Myxococcota	*Myxococcus xanthus*	WP_140795178.1	WP_140857059.1	WP_174259366.1
Myxococcota	*Pyxidicoccus caerfyrddinensis*	WP_164000549.1	WP_164000547.1	WP_164000545.1
Myxococcota	*Pyxidicoccus fallax*	WP_169350108.1	WP_169350107.1	WP_169350106.1
Myxococcota	*Pyxidicoccus parkwaysis*	WP_206724901.1	WP_206724902.1	WP_371877666.1
Myxococcota	*Pyxidicoccus sp. MSG2*	WP_267859784.1	WP_267859785.1	WP_267859786.1
Myxococcota	*Pyxidicoccus trucidator*	WP_164018915.1	WP_164018914.1	WP_164018913.1
Myxococcota	*Pyxidicoccus xibeiensis*	WP_253983710.1	WP_253983709.1	WP_253983708.1
Myxococcota	*Stigmatella ashevillena*	WP_272144062.1	WP_272144064.1	WP_272144065.1
Myxococcota	*Stigmatella aurantiaca*	WP_013374927.1	WP_002613833.1	WP_013374928.1

Myxococcota	*Stigmatella erecta*	WP_093519928.1	WP_093519930.1	WP_093519932.1
Myxococcota	*Stigmatella hybrida*	WP_225411804.1	WP_225411805.1	WP_225411806.1
Myxococcota	*Vulgatibacter sp.*	WP_373045413.1	WP_373045414.1	WP_373045415.1
Planctomycetota	*Engelhardtia mirabilis*	WP_145070674.1	WP_419191964.1	WP_419191963.1
Planctomycetota	*Saltatorellus ferox*	WP_145205850.1	WP_419190757.1	WP_145205827.1
Rhodothermota	*Longibacter salinarum*	WP_098074559.1	WP_098074888.1	WP_098074867.1
Rhodothermota	*Longimonas halophila*	WP_098061945.1	WP_098063067.1	WP_098060759.1
Rhodothermota	*Longimonas sp.*	WP_353562849.1	WP_353564194.1	WP_353562900.1
Rhodothermota	*Rhodocaloribacter litoris*	WP_166974234.1	WP_228350645.1	WP_166978060.1
Rhodothermota	*Rhodothermus bifroesti*	WP_210373907.1	WP_210375124.1	WP_210375121.1
Rhodothermota	*Rhodothermus marinus*	WP_029933186.1	WP_161540516.1	WP_012843179.1
Rhodothermota	*Rhodothermus profundi*	WP_072715349.1	WP_072715210.1	WP_072715214.1
Rhodothermota	*Rubricoccus marinus*	WP_094547598.1	WP_094548922.1	WP_094545390.1
Rhodothermota	*Rubrivirga marina*	WP_095512051.1	WP_095512152.1	WP_095511188.1
Rhodothermota	*Rubrivirga sp.*	WP_424519443.1	WP_420456587.1	WP_424520788.1
Rhodothermota	*Salinibacter altiplanensis*	WP_103029746.1	WP_103018943.1	WP_103020602.1
Rhodothermota	*Salinibacter grassmerensis*	WP_263786151.1	WP_263785401.1	WP_263786104.1
Rhodothermota	*Salinibacter ruber*	WP_259170794.1	WP_259083461.1	WP_251923059.1
Rhodothermota	*Salinibacter sp.*	WP_375800574.1	WP_332316875.1	WP_263834564.1
Rhodothermota	*Salisaeta longa*	WP_022835967.1	WP_022836238.1	WP_028567198.1
